# Low‐Density Lipoprotein Receptor‐Related Protein 6 Cell Surface Availability Regulates Fuel Metabolism in Astrocytes

**DOI:** 10.1002/advs.202004993

**Published:** 2021-06-27

**Authors:** Hei‐Man Chow, Jacquelyne Ka‐Li Sun, Ronald P. Hart, Kenneth King‐Yip Cheng, Clara H. L. Hung, Tsun‐Ming Lau, Kin‐Ming Kwan

**Affiliations:** ^1^ School of Life Sciences, Faculty of Science The Chinese University of Hong Kong 999077 Hong Kong; ^2^ Department of Cell Biology and Neuroscience Rutgers University Piscataway NJ 08854 USA; ^3^ Department of Health Technology and Informatics The Hong Kong Polytechnic University 999077 Hong Kong; ^4^ The University Research Facility in Life Sciences The Hong Kong Polytechnic University 999077 Hong Kong

**Keywords:** Alzheimer's disease, amino acid metabolism, astrocyte, metabolic reprogramming, Wnt signaling

## Abstract

Early changes in astrocyte energy metabolism are associated with late‐onset Alzheimer's disease (LOAD), but the underlying mechanism remains elusive. A previous study suggested an association between a synonymous SNP (rs1012672, C→T) in *LRP6* gene and LOAD; and that is indeed correlated with diminished *LRP6* gene expression in the frontal cortex region. The authors show that LRP6 is a unique Wnt coreceptor on astrocytes, serving as a bimodal switch that modulates their metabolic landscapes. The Wnt‐LRP6 mediated mTOR‐AKT axis is essential for sustaining glucose metabolism. In its absence, Wnt switches to activate the LRP6‐independent Ca^2+^‐PKC‐NFAT axis, resulting in a transcription network that favors glutamine and branched chain amino acids (BCAAs) catabolism over glucose metabolism. Exhaustion of these raw materials essential for neurotransmitter biosynthesis and recycling results in compromised synaptic, cognitive, and memory functions; priming for early changes that are frequently found in LOAD. The authors also highlight that intranasal supplementation of glutamine and BCAAs is effective in preserving neuronal integrity and brain functions, proposing a nutrient‐based method for delaying cognitive and memory decline when LRP6 cell surface levels and functions are suboptimal.

## Graphical abstract

Astrocytes are the major brain metabolic workhorses and altered energy metabolism is associated with late‐onset Alzheimer's disease. Differential Wnt downstream signaling modulates the metabolic landscape in these cells. Intranasal supplementation of enhanced demand on glutamine and branched‐chain amino acids may help to preserve neuronal integrity and brain functions; suggesting an alternative nutrient‐based method for delaying cognitive and memory decline.

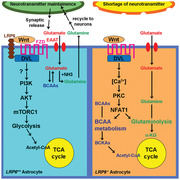

## 1 Introduction

Wnt signaling is well‐conserved throughout evolution,^[^ ^1^ ^]^ and is particularly important to the central nervous system. A strong relationship between the loss of Wnt signaling and neuronal damage in late‐onset Alzheimer's disease (LOAD) is noticed.^[^ ^2^ ^]^ Populational studies indicated that different metabolic‐related risk factors contribute to accelerated age‐related cognitive decline and even LOAD.^[^ ^3^ ^]^ Recent studies indicated that Wnt signaling may serve as a unifying coordinator of cellular metabolism;^[^ ^4^ ^]^ insulin signaling, and mitochondrial biogenesis.^[^ ^5^ ^]^ Mutations of Wnt signaling components are known risk factors of metabolic syndromes as well. An intron polymorphism of *β*‐catenin cofactor *TCF7L2* is related to increased risk of type II diabetes (T2DM).^[^ ^6^ ^]^ Gene polymorphisms of Wnt coreceptor *LRP6* are also associated with T2DM,^[^ ^7^ ^]^ hyperlipidemia;^[^ ^8^ ^]^ atherosclerosis,^[^ ^9^ ^]^ and even LOAD.^[^ ^10^ ^]^ These findings suggest that defective Wnt signaling in the brain may play an important link between altered fuel metabolism and early LOAD pathogenesis.

The energy requirements of the central nervous system are exceptionally high.^[^ ^11^ ^]^ Inside the brain, astrocytes are the major metabolic workhorses, supplying fuel metabolites and biosynthetic building blocks to the neighboring cells, mainly neurons.^[^ ^12^ ^]^ The brain Wnt signaling is well‐studied in neurons, with which primarily mediates the canonical *β*‐catenin pathway. Mature astrocytes are also exposed to the very same microenvironment of Wnt ligands and antagonists, but insights on the corresponding signaling axis are limited. In this report, we show that LRP6 is a unique Wnt co‐receptor expressed on astrocytes with its LOAD‐associated SNP (rs1012672) linked to reduced gene expression in the frontal cortex region. The mTOR‐AKT axis is the primary Wnt downstream signaling pathway mediated by the LRP6 coreceptor, which is essential for maintaining the astrocyte physiological glycolytic metabolic network and its auxiliary functions to neurons. Knockdown of *Lpr6* switches Wnt signal to the coreceptor‐independent calcium‐planar cell polarity (PCP)‐NFAT axis; thereby reprograms the cellular metabolic dependence on glucose to glutamate‐derived glutamine and branched chain amino acids (BCAAs). Exhaustion of these amino acids by astrocytes which are normally needed for synaptogenesis results in neuronal synaptic dysfunction, triggering both cognitive and memory deficits. Bridging these further to LOAD, exposure of human cultured astrocytes to APOE4 triggers cytosolic entrapping of the membrane LRP6 which reminiscences the effects observed when *Lrp6* expression is lost. By a nutrient‐based approach, we uncovered that the neurotoxic effect of LRP6 loss is predominantly metabolic, as intranasal supplementations of glutamine and BCAAs were beneficial in preserving physiological and functional integrity of neurons, suggesting an unconventional but feasible way for delaying cognitive and memory decline.

## 2 Results and Discussion

### 2.1 Differential Wnt Signal Component Profiles between Astrocytes and Neurons

**Figure 1:**
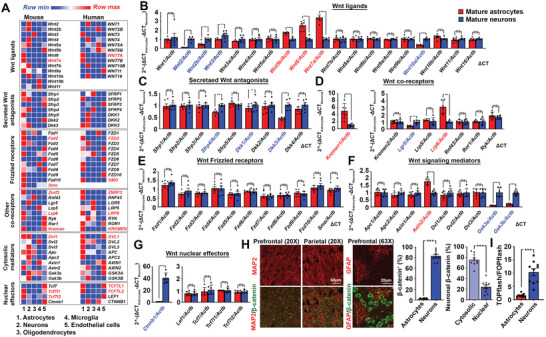
Wnt signaling network in the brain. A) Referencing the Brain RNA‐Seq portal, normalized fragments per kilobase million (FPKM) values of Wnt signaling components in both human and mouse mature brain cells are compared. Red highlights are genes commonly enriched in both human and mouse astrocytes. B–G) Quantitative PCR analyses of various categories of Wnt signaling components from messages harvested from mouse primary neurons and astrocytes. The expression of genes of interests is normalized against expression level of beta‐actin. Relative fold changes between astrocytes and neurons are calculated (*N* = 6, ***p <0.0001, **p <0.001, *p <0.01, ns = non‐significant, two‐tailed unpaired *t*‐test). Red highlights are genes significantly enriched in astrocytes whereas blue ones are those significantly enriched in neurons. H) Representative immunostaining images of *β*‐catenin signals in MAP‐positive neurons or GFAP‐positive astrocytes in the cortex regions. Quantification of relative proportion of *β*‐catenin‐positive cells types (*N* = 10, ***p <0.0001, unpaired *t*‐test); and the relative proportion of cytosolic versus nuclear *β*‐catenin signals in neurons are shown (*N* = 10, ***p <0.0001, two‐tailed unpaired *t*‐test). I) Nuclear *β*‐catenin activities in Wnt‐ligand exposed DIV14 primary neurons and cultured astrocytes (*N* = 10, ***p <0.0001, ns = non‐significant, two‐tailed unpaired *t*‐test). Values represent the Mean ± SEM.

Wnt signaling is initiated by ligand‐receptor interaction at the cell membrane.^[^ ^13^ ^]^ Referencing the Wnt database and the public‐deposited brain cell RNA‐sequencing transcriptome data;^[^ ^14^ ^]^ comparison of normalized fragments per kilobase million (FPKM) values of a list of major mammalian Wnt signaling components was performed. Among various mature brain cell types, multiple Wnt components were enriched in both neurons and astrocytes, suggesting they actively shape the Wnt signaling network in the brain (**Figure** **1**A; Figure S1A, Supporting Information). In agreement with previous reports, neuronal Wnt signaling components were primarily canonical;^[^ ^15^ ^]^ but freshly harvested, fully differentiated astrocytes expressed a different set of Wnt signaling components (Figure 1B–G). In mouse neurons, gene expression of canonical signaling components, including ligands *Wnt2, Wnt2b, Wnt3*, and *Wnt10a*; secreted Wnt antagonists *Sfrp4*, *Dkk1, and Dkk3*; coreceptor *Lgr5*; cytosolic mediators *Gsk3α* and *Gsk3β* (Figure 1B–F); as well as the downstream effector *Ctnnb1* (Figure 1G) along with its protein distribution and nuclear activities (Figure 1H,I; Figure S1B, Supporting Information, left panel) were found enriched. In contrast to neurons, gene expression of non‐canonical signaling components, including ligands *Wnt5b*, *Wnt6*, and *Wnt7a*; coreceptors *Lrp6* and *Kremen1*; as well as canonical Wnt signaling suppressor *Axin* were enriched in freshly harvested mature mouse astrocytes (Figure 1B–G; Figure S1B,C, Supporting Information). Moreover, immunohistochemistry and luciferase assays confirmed that freshly harvested mature astrocytes showed no signs of *β*‐catenin nuclear signals and activities (Figure 1H,I); indicating an alternative Wnt downstream network was activated in these cells.

### 2.2 Wnt Coreceptor LRP6 Selectively Controls the Metabolic Landscape in Astrocytes

**Figure 2:**
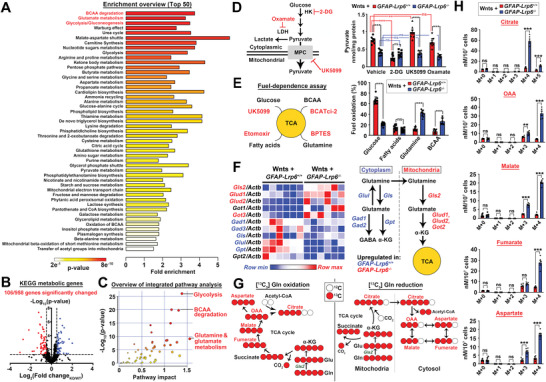
LRP6 shapes the metabolic landscape in astrocytes. A) Global metabolic profiling of freshly enriched *GFAP‐Lrp6^+/+^* and *GFAP‐Lrp6^–/–^* astrocytes followed by the Metabolite Set Enrichment Analysis (MESA) (*N* = 6). Pathways are ranked by significance values. B) qPCR analyses of 958 mouse KEGG metabolic‐related genes expression in freshly enriched *GFAP‐Lrp6^+/+^* and *GFAP‐Lrp6^–/–^* astrocytes (*N* = 6; horizontal dotted line *p* = 0.05; two‐tailed unpaired *t*‐test). C) Integrated metabolites and gene expression analyses were performed on MetaboAnalyst. Top three significant pathways with the greatest impact are labelled. D) Levels of intracellular pyruvate was evaluated in Wnt ligands (Wnt3a, 5a, 6, and 7a recombinant proteins; 100 ng mL^−1^ each) exposed cultured astrocytes after pharmacological inhibition against glucose utilization (50 mm 2‐deoxyglucose [2‐DG]); pyruvate entrance into mitochondria (2 µm UK5099) or lactate conversion (2.5 mm oxamate) for 4 h (*N* = 8, ***p <0.0001, **p <0.001, ns = non‐significant, One‐way ANOVA). E) Seahorse Mito Fuel Flex test on evaluating fuel dependent was performed with slight modification to include an inhibitor against BCAA catabolism (5 µm BCATci‐2) (*N* = 10, ***p <0.0001, ns = non‐significant; two‐tailed unpaired *t*‐test). F) Expression levels of genes in charge of both cytosolic (blue) and mitochondrial (red) glutaminolysis were analyzed in cultured astrocytes. All genes of interests are normalized against *Actb* internal control (*N* = 6). G) Schematic representation of oxidative and reductive reactions in glutamine‐glutamate‐*α*‐ketoglutarate flux. H) Mass isotopologue analysis of citrate; oxaloacetate (OAA); malate; fumarate; and aspartate in *GFAP‐Lrp6^+/+^* and *GFAP‐Lrp6^–/–^* cultured astrocytes exposed to [U‐^13^C]‐glutamine for 2 h (*N* = 5, ***p <0.0001, **p <0.001, ns = non‐significant, two‐tailed unpaired *t*‐test). Values represent the Mean ± SEM.

While Wnt ligands and frizzled receptors are expressed in multiple isoforms and sometimes deemed to be functionally redundant; LRP5 and LRP6 are the two unique classic mammalian Wnt coreceptors.^[^ ^16^ ^]^ Our data revealed that LRP6 was the isoform selectively enriched in mature astrocytes (Figure 1A,D; Figure S1C, Supporting Information). With the existing associations present between *LRP6* mutations and a spectrum of metabolic disorders, we speculated that LRP6‐mediated Wnt signaling modulates the metabolic landscape of these cells. With an inducible conditional knockout mouse model targeting *Lrp6* in astrocytes (Figure S2A,B, Supporting Information), astrocytes were enriched from cortical tissues using astrocyte cell surface antigen‐2 (ACSA‐2) based immunoaffinity method (Figure S2B–H, Supporting Information).^[^ ^17^ ^]^ With freshly enriched astrocytes, global metabolite profiling was immediately performed. Compared to cells enriched from wide type animals; *GFAP‐Lrp6^−/−^* astrocytes revealed enriched clusters of metabolites indicated in BCAAs degradation; glutamate metabolism and glycolysis/gluconeogenesis (**Figure** **2**A; Tables S1 and S2, Supporting Information). Further qPCR analysis of 958 KEGG metabolic‐related genes indicated 106 of them were altered in *GFAP‐Lrp6^−/−^* astrocytes (Figure 2B). By the integrated transcriptomic and metabolomic analysis; the same glutamine‐glutamate metabolism; glycolysis and BCAA degradation pathways were indicated as ones with the highest pathway impacts (Figure 2C; Table S3, Supporting Information). With Wnt‐exposed culture astrocytes subjected to acute knockdown of *Lrp6* (Figure S2I,J, Supporting Information), similar changes were found (Figure S2K–M, Supporting Information) as those suffered from chronic knockout of *Lrp6* harvested from the transgenic animals (Figure 2A–C); indicating that these metabolic changes were unlikely an outcome of any functional compensation effects.

Under physiological conditions, glucose is the predominant source of carbon feeding into the tricarboxylic (TCA) cycle. By brief in vitro culturing of the enriched cells in the presence of Wnt agonists followed by bioenergetic analyses, this revealed that glucose‐derived pyruvate was substantially lower in GFAP‐*Lrp6^−/−^* astrocytes. The same phenomenon was found even when the fate towards lactate (i.e., Oxamate treatment) or acetyl‐CoA (i.e., UK5099 treatment) formation was suppressed (Figure 2D). This implies that the Wnt‐induced glycolytic flux in *GFAP‐Lrp6^–/–^* cells was defective, as also confirmed by both diminished glucose‐induced and maximal glycolytic capacities revealed in the ECAR assay (Figure S3A, Supporting Information). Despite that, quantities of key TCA cycle intermediates remained indifferent among both types of cells (Figure S3B–D—vehicle, Supporting Information). But when carbon entry from glycolysis into the mitochondria was blocked by UK5099; only wildtype but not *GFAP‐Lrp6^–/–^* astrocytes revealed obvious reductions (Figure S3B–D—UK5099, Supporting Information); hinting the latter cells were running on fuels other than glucose‐derived pyruvate. By substrate‐dependence analysis, *GFAP*‐*Lrp6^−/−^* cells revealed elevated dependence on glutamine‐ and to a lesser extent BCAAs (Figure 2E). This phenomenon was also reflected by reduced intracellular levels of glutamine and glutamate in Wnt‐exposed cultures (Figure S3E, Supporting Information). Considered also the upregulated expression of genes involved in mitochondrial glutaminolysis (Figure 2F); hinting that glutamine and glutamate were supporting the fidelity of the TCA cycle. To further evaluate that, stable isotope tracing of metabolic fates of (^13^C_5_‐Glutamine) was performed. Oxidative glutamine metabolism would generate mainly (M+4) forms of TCA cycle intermediates. In contrast, reductive carboxylation would yield (M+5) form of citrate and (M+3) forms of oxaloacetate (OAA), aspartate, fumarate, and malate (Figure 2G). Isotopologue profiling revealed that Wnt‐exposed *GFAP‐Lrp6^−/−^* cells contained more labelled metabolites than wildtype cells; and with majority of them being M+4 labelled; affirming oxidative flux is the predominant fate (Figure 2H). Similarly, levels of all BCAAs were reduced as well but their corresponding downstream branched‐chain *α*‐ketoacids (BCKAs) were induced (Figure S3F, Supporting Information), hinting enhanced BCAA catabolism. Selective tracing of (^13^C_6;_^15^N‐Isoleucine) revealed that instead of taking part in the recycling reactions with *α*‐ketoglutarate and glutamate, which occurred predominantly in wildtype cells (Figure S3G‐Ile panel, Supporting Information); oxidative catabolism was enhanced in *GFAP‐Lrp6^−/−^* cells (Figure S3G‐citrate and *α*‐KG panels, Supporting Information). Together, the switching from glucose‐to glutamine and BCAAs‐dependences in Wnt‐exposed *GFAP‐Lrp6^−/−^* astrocytes suggested that the loss of *Lrp6‐*mediated Wnt signaling results in metabolic reprogramming in these cells.

### 2.3 LRP6‐Depedent and Independent Wnt Signaling Axes Regulate the Metabolic Network in Astrocytes

**Figure 3:**
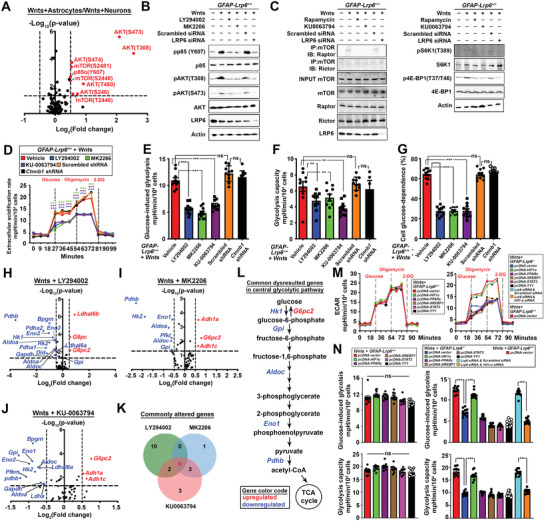
LRP6‐mediated Wnt signaling models the metabolic network in astrocytes. A) Normalized signal intensities of phosphorylated and total forms of each protein were determined, the ratio (phospho/total) of each protein was calculated between cultured astrocytes and neuron*s* exposed to Wnt ligands. Dotted line indicates *p* = 0.05. Red highlights are genes significantly enriched in astrocytes whereas blue ones are those significantly enriched in neurons (*N* = 6, two‐tailed unpaired *t*‐test). B,C) Representative Western blots of cultured *GFAP‐Lrp6^+/+^* astrocytes subjected to pharmacological inhibition against PI3K (10 µm LY294002), AKT (1 nm MK2206), mTOR (0.1 nm rapamycin/ 10 nm KU‐0063794) for 24 h; or having *Lrp6* acutely knockdown for 72 h prior harvesting. Quantifications are shown in *y* Figure S4A,B, Supporting Information. D) ECAR profile, E) glucose‐induced glycolysis, F) glycolysis capacities and glucose dependence of Wnt ligands exposed cultured *GFAP‐Lrp6^+/+^* astrocytes pre‐treated with 10 µm LY294002, 1 nm MK2206, 10 nm KU‐0063794 for 24 h; or having *Ctnnb1* knockdown for 72 h were analyzed (*N* = 10, ***p <0.0001; **p <0.01; One‐way ANOVA). H–J) Comparisons of 60 glycolysis pathway gene expression levels in Wnt ligands‐treated cultured *GFAP‐Lrp6^+/+^* astrocytes exposed to 10 µm LY294002, 1 nm MK2206, or 10 nm KU0063794 for 24 h. Comparisons were made against corresponding vehicle treatment controls (*N* = 6‐9; dotted line *p* = 0.05; two‐tailed unpaired *t*‐test). K) Venn diagram showing the number of commonly altered genes identified from Figure 3H–J; L) 6 out of 10 commonly altered genes were mapped to the central glycolysis pathway. Red highlights are genes commonly upregulated whereas blue highlights are those commonly downregulated. M) Glycolytic functions of Wnt ligand‐exposed *GFAP‐Lrp6^+/+^* and *GFAP‐Lrp6^–/–^* astrocyte cultures ectopically expressing (72 h) various known downstream modulators of mTOR were evaluated (*N* = 10, ***p <0.0001; two‐tailed unpaired *t*‐test at corresponding time points). N) Glucose‐induced and maximal glycolytic capacities were calculated from data in Figure 3M (*N* = 10, ***p <0.01; one‐way ANOVA). Values represent the Mean ± SEM.

With the above observations, but yet the canonical *β*‐catenin signaling is unlikely the downstream mechanism of Wnt‐LRP6‐mediated signaling (Figure 1H–I); potential Wnt network in astrocytes was therefore investigated by a high‐throughput ELISA‐based antibody assay which allows unbiased detection of phosphorylation events related. Relative to neurons, strong levels of phosphorylated PI3K p85, GSK3*β*, AKT, and mTOR were enriched while levels of various forms of phosphorylated *β*‐catenin were substantially lower in Wnt‐exposed astrocytes (**Figure** **3**A; Table S4, Supporting Information). The PI3K and AKT involvements were confirmed further by their respective pharmacological inhibitors (Figure 3B; Figure S4A, Supporting Information). Downstream to that, mTOR may serve as a core component of either complex 1 (mTORC1) or 2 (mTORC2). Co‐immunoprecipitation assay revealed that mTOR in astrocytes preferentially interacted with Raptor; and such combination also led to elevated phosphorylation levels of S6K1 and 4E‐BP1 proteins in these cells (Figure 3C; Figure S4B, Supporting Information). The upstream role of LRP6 on the PI3K‐AKT‐mTORC1 was also validated by siRNA knockdown assays (Figure 3B,C; Figure S4A,B, Supporting Information); and that these inter‐related regulated events were independent of *β*‐catenin (Figure S3H,I, Supporting Information).

Among various cellular functions of mTOR complexes, mTORC1 is by far being best characterized as the cellular metabolic coordinator.^[^ ^18^ ^]^ With wildtype astrocytes, pharmacological inhibition against PI3K, AKT, or mTOR resulted in impairments of glucose‐induced glycolysis and glycolytic capacity and dependence; and these changes were again *β*‐catenin independent (Figure 3D–G). Quantitative PCR analysis suggested these changes were at least in part attributed to the reduced expression of genes supporting the glycolytic pathway (Figure 3H–L). Previous studies suggested mTORC1 coordinates metabolism through regulating several transcription factors, including HIF1*α*, PPAR*γ*, STAT3, SREBP1, and YY1.^[^ ^18^ ^]^ Among them, HIF1*α* responded well to Wnt ligands stimulation and mTOR inhibition in astrocytes (Figure 3M,N; Figure S4C, Supporting Information). The involvement of HIF1*α* was further validated as its knockdown diminished the rescuing effect on ECAR mediated by LRP6 ectopic expression in *GFAP‐Lrp6^−/−^* astrocytes (Figure 3M,N). Intriguingly, the compensatory dependences on glutamine and BCAAs and the induction of corresponding pathway genes were not observed simply by direct inhibition of the PI3K‐AKT‐mTOR axis (Figure S4D–G, Supporting Information). Under these circumstances, we found that dependence on fatty acids was unexpectedly induced instead (Figure S4F, Supporting Information). Together, these implied that the PI3K‐AKT‐mTORC1 axis is involved only in regulating the glycolytic homeostasis, whereas cellular adaptions to glutamine and BCAAs when *Lrp6* is lost are likely regulated simultaneously by alternative mechanisms.

**Figure 4:**
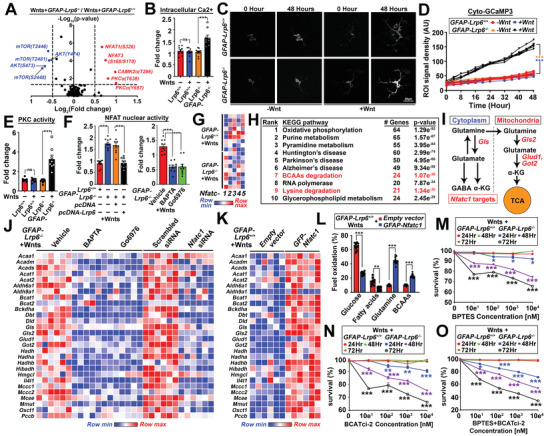
LRP6‐independent Wnt‐ Ca^2+^‐PKC‐NFAT pathway induces glutamate and BCAA‐dependence. A) Normalized signal intensities of phosphorylated and total forms of each protein were determined, the ratio (phospho/total) of each protein was calculated between *GFAP‐Lrp6^+/+^* and *GFAP‐Lrp6^–/–^* astrocytes exposed to Wnt ligands. The dotted line indicates *p* = 0.05. Red highlights are genes significantly enriched in *GFAP‐Lrp6^–/–^* astrocytes whereas blue highlights are those significantly enriched in *GFAP‐Lrp6^+/+^* astrocytes (*N* = 6, two‐tailed unpaired *t*‐test). B) Intracellular calcium level was evaluated in Wnt‐exposed cultured *GFAP‐Lrp6^+/+^* and *GFAP‐Lrp6^–/–^* astrocytes by colorimetric assay (*N* = 9, ***p <0.0001, two‐tailed unpaired *t*‐test). C,D) Representative fluorescent images taken at 0 or 48 h in cultured *GFAP‐Lrp6^+/+^* and *GFAP‐Lrp6^–/–^* astrocytes pre‐transfected with pcDNA3‐Cyto‐GCaMP3 reporter which the signal intensities reflect the cytosolic level of Ca^2+^ ions. Trajectory changes in signal intensities in regions of interests (ROIs) are shown (*N* = 10, ***p <0.0001; two‐tailed unpaired *t*‐test). E) PKC activities in *GFAP‐Lrp6^+/+^* and *GFAP‐Lrp6^–/–^* astrocyte cultures were evaluated by colorimetric assay (*N* = 10, ***p <0.0001; two‐tailed unpaired *t*‐test). F) Luciferase assays of NFAT nuclear activities in lysates harvested from Wnt‐exposed astrocyte cultures. *GFAP‐Lrp6^–/–^* astrocytes were subjected to various manipulations, including ectopic expression of *Lrp6* for 72 h; pharmacological treatment with calcium chelator 5 µm BAPTA‐AM or PKC inhibitor 5 nm Go6976 for 24 h (*N* = 10, ***p <0.0001; one‐way ANOVA). G) Quantitative PCR analyses of various *Nfat* family genes in Wnt ligands treated *GFAP‐Lrp6^+/+^* and *GFAP‐Lrp6^–/–^* astrocytes. Expression of genes of interests were normalized against expression level of beta‐actin (*N* = 9). H) Top ten enriched KEGG pathways are listed among the 371 *NFATC1* target genes with functional roles indicating in fuel metabolism. I) Schematic diagram showing cytoplasmic and mitochondrial glutaminolysis pathway. Red and blue highlights indicate *ENCODE NFATC1* targets. J,K) Relative expression levels of *NFATC1* targets implicated in glutamine and BCAAs metabolism were analyzed in J) Wnt ligands‐treated *GFAP‐Lrp6^–/–^* astrocytes subjected to 24 h 5 µm BAPTA‐AM or 5 nm Go6976 treatment; or having *Nfatc1* knockdown for 72 h (*N* = 6). K) The same analyses were performed in the same cells ectopically expressed *Nfatc1* (*N* = 6). All genes were normalized against the expression level of beta‐actin. L) Fuel dependence was evaluated in Wnt‐exposed *GFAP‐Lrp6^+/+^* astrocytes ectopically expressed *Nfatc1* for 72 h. The relative dependences on various fuels were calculated and compared with empty vector controls (*N* = 10, ***p <0.0001, **p <0.001; two‐tailed unpaired *t*‐test within each metabolite). M–O) Cell survival curves of *GFAP‐Lrp6^+/+^* and *GFAP‐Lrp6^–/–^* astrocytes treated with various dosages of M) BPTES, N) BCATci‐2, or (O) both for different time courses (*N* = 6, ***p <0.0001, **p <0.001, *p <0.01, Two‐way ANOVA). Values represent the Mean ± SEM.

LRP6 is a Wnt coreceptor. In its absence, Wnt may trigger coreceptor‐independent signaling pathways.^[^ ^19^ ^]^ With the Wnt antibody ELISA array, while reductions in phosphorylated PI3K p85*α* subunit; AKT and mTOR were re‐captured in Wnt‐exposed *GFAP‐Lrp6^−/−^* astrocytes; elevated phosphorylated levels of CaMKII*α*, CaMKII(*β*/*γ*/*δ*), PKC*α*, NFAT1, and NFAT3 were simultaneously found (**Figure** **4**A; Table S4, Supporting Information). With elevated levels of intracellular Ca^2+^ ions also (Figure 4B–D), activated PKC*α* (Figure 4E) and NFAT nuclear activities (Figure 4F) were found in in vitro cell‐based assays, these suggested that the non‐canonical Ca^2+^‐PKC‐NFAT axis was turned on as LRP6‐independent Wnt signaling pathway. The NFAT family of transcription factors is well‐indicated in modulating the immune cell metabolic landscape.^[^ ^20^ ^]^ With *Nfatc1* as the predominant isoform expressed in Wnt‐exposed *GFAP‐Lrp6^–/–^* astrocytes (Figure 4G); 6812 of its downstream targets were then identified from the *ENCODE* database (Table S5, Supporting Information). Among them, 387 were common to the 958 genes indicated in the KEGG metabolic network (Table S6, Supporting Information). By the gene set enrichment analysis, BCAAs metabolism‐related pathways, including “Valine, leucine and isoleucine degradation” and “lysine degradation” were found among the top ten ranked pathways (Figure 4H; Table S7, Supporting Information). Despite glutamate metabolism was not among the top pathways listed; genes involved in mitochondrial glutaminolysis were indeed bona fide *NFATC1* targets as well (Figure 4I). The induced expressions of these genes in Wnt‐exposed *GFAP‐Lrp6^–/–^* was abolished when the Ca^2+^‐PKC‐NFAT axis was pharmacologically inhibited or when the *Nfatc1* was silenced (Figure 4J). Consistently, a reversed trend of these gene expressions (Figure 4K) and the metabolic switching towards glutamine and BCAAs‐dependences (Figure 4L; Figure S5A–C, Supporting Information) were found when *Nfatc1* was ectopically expressed in WT astrocytes. These therefore confirmed the modulating role of Ca^2+^‐PKC‐NFAT axis in both glutamine and BCAAs metabolism. With increased demand in glutamine oxidation, glutamine‐derived glutathione was found reduced in Wnt‐exposed *GFAP‐Lrp6^–/–^* astrocytes (Figure S5D, Supporting Information). This observation, together with the slightly enhanced basal rate of oxygen consumption (Figure S5F, Supporting Information), resulted in elevated levels of reactive oxygen species in these cells (Figure S5E, Supporting Information). In light of these metabolic reprogramming events, we speculated that *Lrp6* loss reduced metabolic flexibility in astrocytes. By cell survival assays, while inhibition of mitochondrial pyruvate availability or fatty acid oxidation revealed no effects to both genotypes of astrocytes (Figure S5G, Supporting Information); cell death was however observed when either mitochondrial glutaminolysis (Figure 4M), BCAAs catabolism (Figure 4N), or both (Figure 4O) were inhibited in *GFAP‐Lrp6^–/–^* astrocytes. Together this revealed that LRP6‐loss mediated metabolic programming led to diminished metabolic flexibilities in astrocytes.

### 2.4 Loss of LRP6 in Astrocytes Results in Astrogliosis, Leading to Impaired Synaptogenesis, Neurodegeneration, Impaired Cognitive, and Memory Functions in Mice

**Figure 5:**
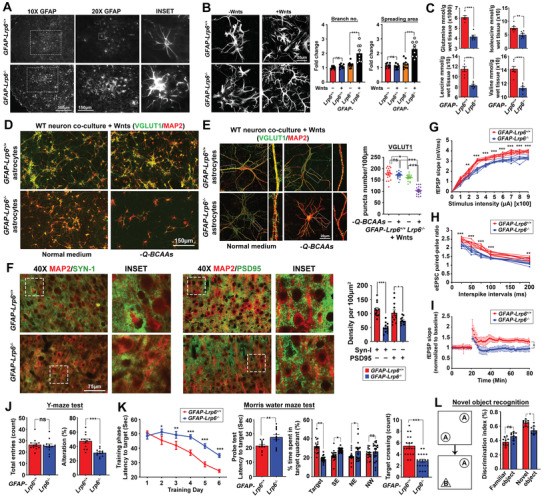
Loss of LRP6 in astrocytes impairs neuronal morphology and synaptic fidelity. A) Representative images of GFAP‐positive astrocytes in the frontal cortex regions (*N* = 6). B) Representative images of *GFAP‐Lrp6^+/+^* and *GFAP‐Lrp6^−/−^* astrocyte cultures. Quantification of branching number and spreading area are shown (*N* = 8; ***p <0.0001, **p <0.001, two‐tailed unpaired *t*‐test). C) Quantities of glutamine and BCAAs in cortical brain tissues were determined by CE‐TOFMS (*N* = 9, ***p <0.0001; **p <0.01; *p <0.01; unpaired *t*‐test). D,E) Representative D) low or E) high magnification images of MAP2‐positive dendrites and VGLUT1‐positive puncta of wildtype neurons co‐cultured with either Wnts‐exposed *GFAP‐Lrp6^+/+^* and *GFAP‐Lrp6^–/–^* astrocytes in the normal culturing medium or in one without supplementation of glutamine and BCAAs (‐Q‐BCAAs). Quantification of VGLUT1 puncta density are shown (*N* = 18, ***p <0.0001, *p <0.01, ns = non‐significant, One‐way ANOVA). F) Representative images of Synapsin‐I (SYN‐I) and PSD95 puncta densities in the frontal cortex regions. Quantification of their puncta densities are shown (*N* = 12, ***p <0.0001, *p <0.01, two‐tailed unpaired *t*‐test). G–I) Electrophysiology recordings in brain slices harvested from *GFAP‐Lrp6^+/+^* and *GFAP‐Lrp6^−/−^* mice. G) fEPSP input–output relationship was recorded in the hippocampal CA1 region upon stimulation at the superior colliculus region (*n* = 6, **p <0.001, *p <0.01, Two‐way ANOVA). H) Whole‐cell eEPSC pair‐pulsed ratio pattern recorded in hippocampal CA1 pyramidal neurons (*n* = 6, ***p <0.0001, **p <0.001, Two‐way ANOVA). I) Long‐term potential recording in superior colliculus after induction of a train of 100‐Hz stimuli. (*N* = 6 *p <0.01, ns = non‐significant, Two‐way ANOVA). J) Quantification of total entries and percentage alternations in a Y‐maze paradigm (*N* = 12, ***p <0.0001, ns = non‐significant, two‐tailed unpaired *t*‐test). K) Escape latency during the training phase (*N* = 12 ***p <0.0001, *p <0.01, Two‐way ANOVA) and probe trial (*N* = 12 **p <0.001, unpaired *t*‐test) of the Morris water maze test. Percentage of time spent in the target quadrant (*N* = 19, **p <0.001, *p <0.01, ns = non‐significant, One‐way ANOVA) and percentage of target crossing were also analyzed (*N* = 19, ***p <0.0001, two‐tailed unpaired *t*‐test). L) Novel object recognition analysis was performed. Frequencies of mice accessing familiar objects (object A) versus novel objects (object B) were recorded. Exploration time was analyzed and shown (*N* = 10, *p <0.01, ns = non‐significant, One‐way ANOVA). Values represent the Mean ± SEM.

Metabolic reprogramming is an adaptive mechanism facilitating phenotypic changes and functional properties of a cellular system.^[^ ^21^ ^]^ Combining the reduced expression of physiological (i.e., *Slc1a2*, *Slc1a3*) but elevated expression of reactive (i.e., *Gfap, Vim*) markers in enriched *GFAP‐Lrp6^–/–^* astrocytes (Figure S2F,G, Supporting Information); together with the morphological changes (i.e., increased branching number and spreading area) observed (**Figure** **5**A,B; Figure S6A, Supporting Information); these observations suggested a potential reactive astrogliosis event occurs upon LRP6‐deficiency. We observed that the changes in metabolic dependence of astrocytes reflected as reduced contents of glutamine and BCAAs at brain tissue level (Figure 5C); indicating their availability, as well as the ammonia byproduct of their oxidative catabolism may affect other cells in the brain. Amino acids are natural nitrogen carriers and their chronic catabolism can lead to ammonia production and toxicity; however our body could detoxify it as urea through the urea cycle.^[^ ^22^ ^]^ In both the in vitro (i.e., conditioned medium) and in vivo (i.e., blood plasma and cerebrospinal fluid [CSF]) systems; while no obvious differences were found in their ammonia levels; elevated urea was however observed in *GFAP‐Lrp6^–/–^* samples (Figure S6B, Supporting Information). Despite such general phenomenon, CSF urea remained unchanged as compared to blood plasma. Referencing the public available single cell transcriptome data,^[^ ^23^ ^]^ astrocytes are the only cell type in the brain expressing the urea transporter‐1 (*Slc14a1*) (Figure S6C, Supporting Information). Moreover, the unbiased metabolomic analysis (Figure 2A, Figure S2K, Supporting Information) and gene expression analysis of multiple urea cycle genes were all found upregulated in *GFAP‐Lrp6^–/–^* samples (Figure S6D, Supporting Information). Together these indicated that an adaptive urea‐producing mechanism was turned on in LRP6‐deficient conditions to cope with the aberrant amino acid catabolism program.

Under physiological conditions, these amino acids may serve as precursors for neurotransmitter recycling and biosynthesis;^[^ ^24^, ^25^ ^]^ therefore, their reduced availabilities from astrocyte to the neighboring neurons may affect their synaptic abundances and functions. To study so, a non‐contact co‐culture system was used^[^ ^26^ ^]^ where enriched astrocytes were seeded on a trans‐well insert and suspended above a monolayer of wildtype primary cortical neurons in a custom medium (Tables S8 and S9, Supporting Information) where the availability of specific metabolites‐of‐interests can be manipulated. In 72 h, wildtype neurons co‐cultured with Wnt‐exposed *Lrp6*‐deficient astrocytes revealed reduced VGLUT1, VGAT, Synapsin‐1, and neurite complexities (Figure 5D,E; Figure S6E–G, Supporting Information), particularly in culture conditions without glutamine and BCAAs supplementations. These changes were also captured in vivo (Figure 5F; Figure S6H–J, Supporting Information); and extended to deficits at tissue electrophysiology levels (Figure 5G–I). Further understanding on how these changes manifest to changes in animal behaviors was sought by a battery of behavioral analyses. Matching with the Allen Brain Atlas data which indicated that LRP6 is enriched predominantly in forebrain regions (Figure S6K, Supporting Information); functions related to these areas, including working, special, and recognition memories (Figure 5J–L) were compromised in *GFAP‐Lrp6^–/–^* mice (Figure S6L–N, Supporting Information).

### 2.5 Intranasal Administration of Glutamine and BCAA Improves Brain Physiology and Function in *GFAP‐Lrp6^–/–^* Mice

**Figure 6:**
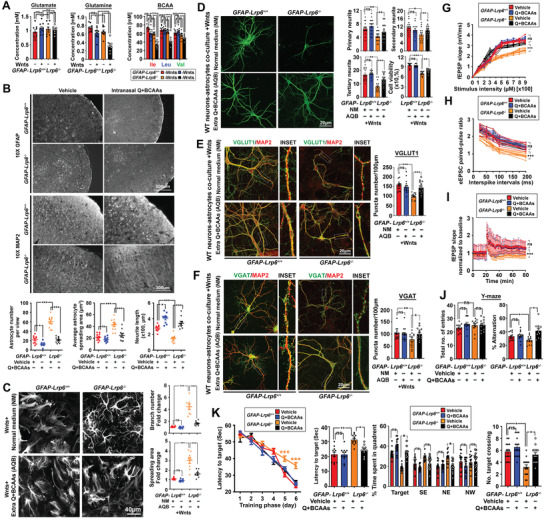
Glutamine and BCAAs supplementation improve neurite integrity; synaptic abundance; cognitive and memory function in *GFAP‐Lrp6^−/−^* mice. A) Extracellular levels of glutamate (left) and glutamine (middle) were measured by colorimetric assays in Wnts‐treated primary astrocyte cultures. Intracellular BCAAs were also determined by GC‐MS (*N* = 9, ***p <0.0001; ns = non‐significant; One‐way ANOVA). B) Representative images of GFAP‐positive astrocytes and MAP2‐positive dendrites at the frontal cortex region of *GFAP‐Lrp6^+/+^* and *GFAP‐Lrp6^−/−^* mice administered with a small bolus (≈15–20 µl) of 560 nmol/0.4 g average brain wet weight of glutamine and a mixture of BCAAs (each 10 nmol/0.4 g average brain wet weight) or equal volume of vehicle for 30 days (*N* = 6). C) Representative images GFAP‐positive astrocytes in Wnts‐exposed cultured astrocytes supplemented with routine culture medium or ones with additional supplementation of glutamine (Q) (340 µm) and BCAAs (Ile: 40 mm, Leu: 20 mm, and Val: 20 mm) (Q+BCAAs). Quantification of branching number and spreading area are shown on the right (*N* = 8, ***p <0.0001; ns = non‐significant; One‐way ANOVA). D) Representative images of MAP2‐positive neurites in wildtype mouse primary neurons co‐cultured with Wnts‐exposed mouse astrocytes in the routine culture medium or in one with extra supplementation of glutamine (Q) (340 µm) and BCAAs (Ile: 40 mm, Leu: 20 mm, and Val: 20 mm). Quantification of primary; secondary, and tertiary neurites and viability of neurons are shown (*N* = 16, ***p <0.0001, **p <0.001, *p <0.01, One‐way ANOVA). E,F) Representative images of E) VGLUT1/MAP2 and F) VGAT/MAP2 staining in wildtype neurons co‐cultured with Wnts‐treated *GFAP‐Lrp6^–/–^* astrocytes in the setting similar to (D). Quantification of puncta density are shown on the right (*N* = 15, ***p <0.0001, ns = non‐significant, One‐way ANOVA). G–I) Electrophysiology recordings in acute brain slices harvested from mice supplemented with vehicles or glutamine plus BCAA in manners mentioned in (B). G) fEPSP input‐output relationship was recorded in the hippocampal CA1 region upon stimulation at the superior colliculus region (*n* = 6, ***p <0.0001; ns = non‐significant, Two‐way ANOVA). H) Whole‐cell eEPSC pair‐pulsed ratio pattern recorded in hippocampal CA1 pyramidal neurons (*n* = 6, ***p <0.0001, ns = non‐significant; Two‐way ANOVA). I) Long‐term potential recording in superior colliculus after induction of a train of 100‐Hz stimuli. (*N* = 6, *p <0.01, ns = non‐significant; Two‐way ANOVA). J) Total entries and percentage alternations in a Y‐maze paradigm (*N* = 12, *p <0.01, ns = non‐significant, One‐way ANOVA). K) Escape latency during the training phase (*N* = 12, ***p <0.0001, Two‐way ANOVA) and probe trial (*N* = 12, **p <0.001, *p <0.01, ns = non‐significant, One‐way ANOVA); percentage of time spent in various quadrants (*N* = 15, *p <0.01, ns = non‐significant, One‐way ANOVA) and number of target crossing (*N* = 15, *p <0.01, ns = non‐significant, One‐way ANOVA) of Morris water maze (MWM) test. Values represent the Mean ± SEM.

The above data suggested that compromised brain functions were likely contributed by reduced glutamate‐derived glutamine and BCAAs availability to neurons. Utilizing the in vitro system where glutamine in the custom medium was replaced with 2 mm glutamate; both types of astrocytes revealed similar extents of glutamate utilization; yet the amount of glutamine released to the medium was significantly diminished in Wnts‐exposed *GFAP‐Lrp6^−/−^* astrocyte culture (**Figure** **6**A). Similarly, levels of BCAAs remained in the custom medium were also reduced, indicating their enhanced usages as well (Figure 6A). With these, we speculated that the exhaustion of these synaptic building blocks was the major contributor of reduced synaptic densities and functions. Targeted supplementation of these metabolites in quantities equivalent to the differences found between *GFAP‐Lrp6^+/+^* and *GFAP‐Lrp6^−/−^* brain samples (Figure 5C) was performed through the intranasal route which allowed direct brain delivery and avoidance of any unexpected degradation in the body system^[^ ^27^ ^]^ (Figure S7A, Supporting Information). Upon 30 days of supplementation, brain tissues of *GFAP‐Lrp6^−/−^* mice revealed alleviated astrogliosis with better neuronal dendritic morphologies and synaptic densities (Figure 6B; Figure S7B,C, Supporting Information). Simultaneously, brain glutamine and BCAAs levels (Figure S7H, Supporting Information), as well as ROS and glutathione levels (Figure S7E, Supporting Information) were also improved in these brain tissues as well. Similar improvements in physiological astroglial (Figure 6C) and neuronal morphologies (Figure 6D; Figure S7D, Supporting Information), neuronal survival (Figure 6D), and synaptic densities (Figure 6E,F) were also found in in vitro assays upon additional glutamine and BCAAs supplementation for 72 h. Functionally, both brain electrophysiology (Figure 6G–I) and memory‐related functions (Figure 6J,K) were substantially improved in *GFAP‐Lrp6^−/−^* mice administered with supplements over vehicle controls. We again failed to find any obvious accumulation of toxic ammonia (Figure S7E, Supporting Information); yet elevated levels of urea were observed in both the conditioned medium from in vitro assays, so as the blood plasma samples from in vivo assays but again not in the CSF (Figure S7G, Supporting Information). Together, these indicated that insufficient glutamine and BCAAs availability due to aberrant astrocytic metabolic consumption could prime synaptic dysfunction and neurodegeneration; but that could be prevented by direct supplementation of these metabolites to the brain system.

### 2.6 Reduced LRP6 Surface Abundances by LOAD Risk Factors APOE4 may Play Roles in Altering Wnt‐Governed Metabolic Network in Human Astrocytes

**Figure 7:**
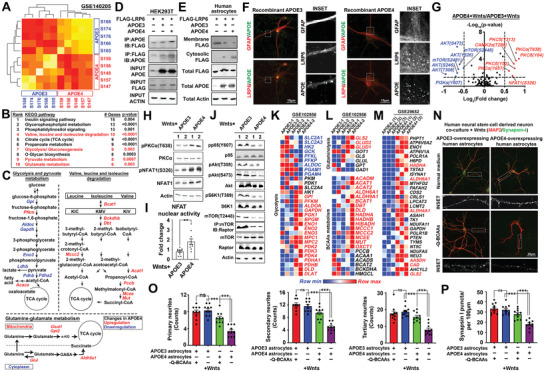
APOE4 interacts with and reduces LRP6 surface abundances; alters Wnt‐governed metabolic network in human astrocytes. A) Heatmap showing the similarity and differences among transcriptome profiles of APOE3 and APOE4 mouse hippocampal samples from the GSE140205 dataset. B) Differential gene expression analyses of 958 KEGG metabolic‐related genes between APOE3 and APOE4 groups. All significantly changed (FDR <0.05) genes were enriched and subjected to GSEA analysis. Top ten enriched KEGG pathways are listed. Red highlights indicate relevant metabolic‐related pathways. C) Significantly altered genes found in the APOE4 group which were involved in “glycolysis and pyruvate metabolism;” “valine, leucine and isoleucine degradation” and “glutamine‐glutamate metabolism” were laid out. Red highlights indicate upregulated genes and blue ones are downregulated. D,E) Representative immunoblots revealing D) stronger interactions between LRP6 and APOE4 over that with APOE3 performed in HEK293T cells (*N* = 6); and E) reduced surface availability of LRP6 when APOE4 was ectopically expressed in wildtype human astrocytes (*N* = 6). F) Representative images showing the cellular distribution of LRP6 along with exogenous recombinant APOE3 or APOE4 proteins in wildtype human astrocytes (*N* = 10). G) Normalized intensities of phosphorylated and total forms of each protein were determined. Ratio (phospho/total) of each protein was calculated between Wnts‐treated APOE4‐and APOE3‐overexpressing human astrocytes. Horizontal dotted line indicates *p* = 0.05 (*N* = 6, two tailed unpaired *t*‐test). H) Representative immunoblots of PKC*α*‐NFAT1 signaling components (*N* = 6) and I) and luciferase assay for NFAT1 nuclear activities (*N* = 9, **p <0.001, two‐tailed unpaired *t*‐test) in Wnts‐treated human astrocytes ectopically expressing either APOE3 or APOE4. J) Representative immunoblots revealed loss of activating phosphorylation on PI3K‐AKT‐mTOR signaling components in Wnts‐treated human astrocytes ectopically expressing APOE4 (*N* = 6). K,L) Referencing the transcriptome data of isogenic *APOE3* and *APOE4* human iPSC‐derived astrocytes (GSE102956); key genes involved in K) glycolysis; L) glutaminolysis and BCAA metabolism were dysregulated. Red indicates upregulated genes and blue ones are downregulated. M) Comparing the transcriptome data of APOE3 and APOE4 astrocytes harvested from human LOAD brains of Braak stages I‐II (GSE29652); significantly changes genes that are also *NFATC1* targets with roles in fuel metabolic pathways were enriched. Red highlights are ones indicated in glutamine and BCAA metabolism. N) Representative images showing neuritic morphology and pre‐synaptic puncta density in human neural stem‐cell derived neurons co‐cultured with Wnts‐treated APOE3 or APOE4‐overexpressing human astrocytes in the normal culturing medium or in one without any glutamine and BCAAs supplementation (‐Q‐BCAAs) (*N* = 10). O) Quantification of primary, secondary, and tertiary neurites together with P) Synapsin‐I puncta density are shown (*N* = 10, ***p <0.0001, ns = non‐significant, One‐way ANOVA). Values represent the Mean ± SEM.

Apart from being a Wnt coreceptor, LRP6 is also a classic member of LDL receptor (LDLR) family (Figure S8A, Supporting Information). Apolipoprotein E is therefore a potential ligand which variants of the more frequent *APOE3* allel*e* to *APOE4* is the greatest genetic risk factor for LOAD.^[^ ^28^ ^]^ Analyses of the 958 KEGG metabolic genes in the published transcriptome datasets of hippocampus tissues harvested from homozygous human APOE3‐ and APOE4 knock‐in mice revealed similar impairments in glycolysis while glutamine and BCAAs metabolisms were induced (**Figure** **7**A,C). This metabolic pattern was indeed similar to the case observed in the LRP6‐deficient model, therefore we speculated if presence of APOE4 alters the function of LRP6 in astrocytes. We first evaluated the binding affinities between LRP6 and the two APOE isoforms. In HEK293T cells where endogenous APOE and LRP6 levels were low (Figure S8B, Supporting Information), overexpression of naturally secreted APOE‐3 and ‐4 isoforms and FLAG‐tagged LRP6; followed by co‐immunoprecipitation analysis revealed that LRP6 showed higher binding affinity towards APOE4 over APOE3 (Figure 7D). Based on known interacting mode between APOE proteins and LRP family members; we predicted that the LDLR type A (LA) domain on LRP6 is likely taking part in APOE binding; yet the true 3D structure remains unsolved. Therefore by auto‐simulating its structure on Phyre^2^ based on its protein amino acids sequence (Figure S8C, Supporting Information);^[^ ^29^ ^]^ the predicted structure indeed well‐resembled to the LDL receptor‐like module on other LRP family members (Figure S8D, Supporting Information). Previous studies suggested that the interaction between APOE and ligand‐binding repeats of LDLR family likely involving electrostatic interactions;^[^ ^30^ ^]^ we then docked the predicted LRP6 LA‐domain to the N‐terminal LDL‐receptor binding region of APOE3 (PDB: INFN, Chain A) or APOE4 (PDB: 1B68, Chain A) using the electrostatic‐favored mode. By comparing the free energy score of each cluster center;^[^ ^31^ ^]^ LRP6 was predicted to favorably bind towards APOE4 (−845.7 kcal mol^−1^) over APOE3 (−799.1 kcal mol^−1^) (Figure S8E, Supporting Information). These findings are important, as the presence of one *APOE4* allele is sufficient to outcompete LRP6:APOE3 interactions in the more common heterozygote conditions.

Previous studies revealed that the double arginine residues on APOE4 render its isoelectric point closer to the pH in the early endosomes (pH = 6.5) (Figure S8E, Supporting Information); hence hampering the associated endosomal recycling.^[^ ^32^ ^]^ In agreement with the findings observed in the ectopic APOE isoforms expression experiment (Figure 7E); addition of recombinant human APOE4 but not the APOE3 proteins directly reduced the membrane availability of LRP6 in human astrocytes; proving that APOE triggered such phenomenon from extracellular origin (Figure 7F). Indeed, we found that human astrocytes behaved as if they had lost LRP6 expression under the influences of APOE4. At signaling level, elevated protein phosphorylation levels that activates the Ca^2+^‐PKC‐NFAT axis were observed whereas many of those indicated in activated PI3K‐AKT‐mTOR pathway were reduced (Figure 7G–J). With the public datasets (GSE102956), expression profiles of key metabolic genes in isogenic *APOE3* and *APOE4* human iPSC‐derived astrocytes were compared (Figure 7K,L). In *APOE4* astrocytes, gene expression levels of glycolysis facilitators like glucose transporters and hexokinases were reduced while key pathway inhibitors like pyruvate dehydrogenase kinases were elevated, indicating diminished glycolysis (Figure 7K). In contrast, key drivers of mitochondrial glutaminolysis (Figure 7L‐top) and that of BCAAs were upregulated (Figure 7L‐bottom). Together, these indicated that human *APOE4* astrocytes acquired a metabolic network resembling that in LRP6‐deficient mouse astrocytes; linking to the changes observed at tissue level (Figure 7A–C). With *APOE4* being the strongest genetic risk factor for LOAD;^[^ ^33^ ^]^ transcriptome profiles of *APOE4‐* and *APOE4+* human astrocytes within the same Braak Stages (I‐II, III‐IV and V‐VI) were also compared (GSE29652). By the GEO2R analyses, 3499; 2278 and 822 significantly changed genes were respectively found in Stages I–II, III–IV, and V–VI (Figure S8F, Supporting Information); hinting that maximal effects of APOE4 on astrocytes might have occurred at the preclinical Braak I–II stages when synaptic deficits are generally initiated.^[^ ^34^ ^]^ By focusing on the 3499 hits identified from Braak I‐II samples; 707 putative *ENCODE* NFATC1 targets were identified (Figure S8F, Supporting Information). Among which, key genes promoting the mitochondrial glutaminolysis and BCAA lysine degradation were found (Figure 7M); hinting that APOE4 astrocytes at these Braak stages are depending more on these amino acids. By the in vitro co‐culture system; human neural stem cell‐derived neurons became more sensitive to glutamine and BCAAs withdrawal in the medium when they were co‐cultured with APOE4‐overexpressing human astrocytes (Figure 7N–P). Together, the entrapping actions of APOE4 on LRP6 from the cell surface alter astrocyte Wnt downstream signalling and its metabolic network, this reminiscent to the changes and systemic effects induced by astrocyte LRP6 deficiencies.

## 3 Discussion

Our work demonstrates how the coreceptor LRP6 serves as a bimodal switch that regulates Wnt downstream signaling and metabolic network in astrocytes. Wnt‐LRP6 triggers the activation of PI3K‐AKT‐mTOR signaling which governs the glycolytic homeostasis that physiologically offers metabolic support to neurons. In its absence, however, Wnt alternatively induces the Ca^2+^‐PKC*α*‐NFAT1 axis which not only favors the acquisition of a reactive phenotype, but also remodels their original metabolic framework to one that favors glutamate and BCAAs catabolism. The net result of such metabolic remodeling is the exhaustion of these key amino acids in the microenvironment that are normally needed for maintaining the synaptic homeostasis and function. This study also has important implications towards the understanding of early pathogenesis of LOAD. First, significant association between a synonymous SNP in exon 18 (rs1012672, C→T) of the *LRP6* gene and LOAD was reported.^[^ ^10^ ^]^ With reference to the *BRAINEC*, reduction in LRP6 was uniquely found in the frontal cortex region in the presence of rs1012672 (Figure S8G, Supporting Information). Combining these with the experimental findings, these hinted that the enhanced risk of rs1012672 to LOAD is at least partially contributed by reduced astrocyte LRP6 and the associated metabolic reprogramming events in the region. Similar to the loss in expression, reductions in LRP6 cell surface abundance also yielded similar effects. We have demonstrated APOE4 interacts with LRP6 and dampens its cell surface abundance. Indeed, previous studies indicated that APOE4 interacts with a number of LRP family members which interferes their functions in both Wnt‐dependent and independent manners;^[^ ^35^ ^]^ whether this applies to LRP6 as well would warrant future investigations. As a Wnt co‐receptor, we speculated that other factors like Wnt antagonist Dickkopf‐1 (DKK1)—as induced by amyloid‐*β* ^[^ ^36^ ^]^—could also interfere LRP6 cell surface availability by triggering its aberrant endocytosis.^[^ ^37^ ^]^ In the aggregate, the astrocytic Wnt‐LRP6 governed metabolic framework in the aging brain may subject to different sources of challenges. While previous studies reported that neuronal deletion of LRP6 may cause synaptic and plasticity deficits;^[^ ^38^ ^]^ here we extended the understanding of LRP6 biology to astrocytes. Our data focused on the metabolic reprogramming events occurred in these cells; highlighting the switch from glucose to glutamine and BCAAs dependence may chronically exhausts key amino acids needed for maintaining the synaptic homeostasis. Indeed, compromised astrocytic glycolytic activities may also contribute to the impaired brain glucose utilization frequently reported in early stages of LOAD.^[^ ^39^ ^]^ While brain contents of BCAAs and its association with LOAD have not been reported yet; their importance to the brain are reflected by the fact that their uptake at the blood brain barrier exceeds that of all other amino acids and that the brain oxidizes BCAAs at much higher rates than peripheral muscles.^[^ ^40^ ^]^

Under physiological conditions, the majority of glutamine re‐synthesized from glutamate in astrocytes is released out to the neurons, serving as a precursor for neurotransmitter biosynthesis via the “glutamate‐glutamine cycle”.^[^ ^41^ ^]^ A previous study indicated that reduction in astrocyte‐dependent glutamine supply to neurons results in reduced synaptic efficacy and that could be reversed by exogenous glutamine supplementation.^[^ ^41^ ^]^ On the other hand, BCAAs are also precursors for synaptic glutamate synthesis via the “glutamate‐BCAAs cycle”, and this pathway is particularly important during brain injuries.^[^ ^25^ ^]^ In these “cycling” events, intermediates like glutamine, glutamate, and BCAAs are reformed and therefore their levels shall be less fluctuating in the brain.^[^ ^42^ ^]^ Our data revealed that chronic insufficiencies of LRP6 compromises glycolytic activities in astrocytes. This implies that pyruvate derived from glucose which normally supplies carbon for acetyl‐CoA and oxaloacetate productions becomes limited.^[^ ^43^ ^]^ The fuel‐dependence as well as the isotopologue tracing tests indicated that glutamine may serve as an immediate surrogate fueling the TCA cycle; where its carbons enter the cycle as alpha‐ketoglutarate and are oxidized to carbon dioxide to form oxaloacetate. Similarly, BCAAs—in particular isoleucine and leucine—supported the TCA cycle alternatively as acetyl‐CoA. Therefore, glutamine‐derived oxaloacetate and BCAAs‐derived acetyl‐CoA eventually condense as citrate in the first step of the TCA cycle and fuel the subsequent oxidative reactions. Because of that, we speculated the repartitioning effects of these amino acids from the recycling reactions (i.e., “glutamate‐glutamine cycle” and “glutamate‐BCAAs cycle” between astrocytes and neurons) towards enhanced oxidative fates (i.e., formation of TCA cycle intermediates and carbon dioxide in astrocytes) resulted in their diminished contents in the brain; leading to diminished neurotransmitter pools and hence the synaptic functions in the brain.

Based on these metabolic findings, we then demonstrated that the altered brain metabolic balance can potentially be circumvented by short‐term intranasal glutamine and BCAAs supplementation, even if altered cellular metabolism could be secondary to pre‐existing genetic or other events during the pathogenesis of LOAD. While BCAAs are essential amino acids and could be limiting already under most circumstances; questions on glutamine supplementation may exist as potential feedback mechanisms from the peripheral may exist to sustain the brain glutamine homeostasis. However, consider a previous report indicated that sharp differences (i.e., 5–10 folds) in levels of amino acids including glutamine exist between the mouse brain interstitial fluid and plasma fluids;^[^ ^44^ ^]^ so as artificial augmentation of plasma glutamine availability by intraperitoneal injection failed to increase the total brain interstitial fluid glutamine in freely moving mice.^[^ ^44^ ^]^ These together suggested that there are no obvious associations between the central and peripheral glutamine levels and reserves;^[^ ^45^ ^]^ and that the proposed feedback mechanism is not as straightforward as thought. These signs also supported the adaption of the intra‐nasal delivery approach over system‐delivery approaches (e.g., via food, water, or intraperitoneal injection, etc.) in our study; which allows direct brain delivery and avoids questions on absorption and unexpected degradation in other body systems.

Amino acids are natural nitrogen carriers, glutamine in particular.^[^ ^46^ ^]^ The metabolic adaption to amino acid catabolism may result in ammonia released as a byproduct, leading to unexpected cellular toxicity. Our data suggested that the induction of ureagenesis in LRP6‐deficient astrocytes is likely a cellular feedback mechanism in coping with the increased ammonia stress resulted from enhanced glutamine and BCAAs metabolism; when the amino acid cycling reactions (as mentioned above) which “locks” ammonia are diminished in these cells. Indeed, astrocyte is the unique cell species in the brain expressing the major urea transporter that facilitates transmembrane urea transport even under physiological conditions (Figure S6C, Supporting Information); suggesting it is their intrinsic property to maintain the local nitrogen homeostasis. Our data suggested that activated urea cycle in the LRP6‐deficient astrocytes is associated with elevated urea excretion to the peripheral circulation. Intriguingly, previous studies also indicated that positive associations exist between elevated brain urea with dementia;^[^ ^47^ ^]^ though it remains unclear whether this relationship is solely attributed to elevated urea production alone, or also due to compromised removal from the brain‐CSF system, or both. Nonetheless, our data reveal a potential mechanism on the potential cause of elevated brain urea production. This sign, together with reduced brain contents of glutamine and BCAAs, as well as compromised brain glycolytic activity, may serve as a unified group of biomarkers useful for early diagnosis.

Studies on specific modulation of amino acids and other metabolites in the diet has indeed shown promise for improving markers of aging and longevity;^[^ ^48^ ^]^ with our findings, this could be potentially adapted for pathological brain aging and early LOAD management.^[^ ^49^ ^]^ We revealed that intranasal glutamine and BCAAs supplementation deemed beneficial when astrocytes were metabolically reprogrammed to consume these amino acids. Although brain‐specific application is yet to be available, glutamine is supplemented clinically to patients with critical illness as it could become “conditionally essential” under these circumstances.^[^ ^50^ ^]^ On the other hand, all BCAAs are by default essential amino acids; and that oral supplements have been used with good tolerance in treatments of several neurologic diseases, including bipolar disorder, tardive dyskinesia, amyotrophic lateral sclerosis, and spinocerebellar degeneration.^[^ ^48^ ^]^ These clinical applications indicated that both glutamine and BCAAs can be consumed in considerable amounts by humans without adverse effects, and in many cases, with significant benefits. Together, our study revealed new insights on the metabolic roles of brain Wnt signalling, with important implications in the early pathogenesis of LOAD. Our work also suggests that direct brain supplementations of glutamine and BCAAs are beneficial for preserving the function and neuronal integrity in carriers of *APOE‐e4* alleles or even *LRP6* point mutations. This offers evidence to support nutrient‐based alternative medicine approach for managing neurodegenerative disorders.

## 4 Experimental Section

Unless otherwise specified, all chemicals and reagents were purchased from Sigma. Details of antibodies and special reagents are listed in Table S10, Supporting Information. Sequence‐based reagents, including primers for real‐time PCR and sub‐cloning are listed in Table S11, Supporting Information.

### Mouse and Human Microarray Data Mining

The brain RNA‐seq transcriptome databases of glia, neurons, and vascular cells of the cerebral cortex from (https://web.stanford.edu/group/barres_lab/brain_rnaseq.html) and (http://celltypes.org/brain/) were quarried with a list of mammalian Wnt‐related genes built from the Wnt homepage (http://web.stanford.edu/group/nusselab/cgi‐bin/wnt/). Normalized FPKM values of different genes from mature astrocyte, neuron, myelinating oligodendrocytes, microglia, and endothelial cells were obtained, categorized, and plotted as heatmaps using the online Morpheus platform (https://software.broadinstitute.org/morpheus/). K‐means clustering (number of clusters = 2) was performed to genes with similar expression pattern using the one minus Pearson Correlation metric with Morpheus. Public datasets GSE140205, GSE102956, and GSE29562 were accessed from GEO Omnibus. Analyses and data extraction were performed by EdgeR and GEO2R, respectively.

### Animal Maintenance, Cortical Brain Tissue Isolation, Collection of Blood Plasma and Cerebrospinal Fluid, Mouse Primary or Human Neuronal and Astrocyte Cultures

*C57BL/6J*, *GFAP‐Cre* [B6.Cg‐Tg(GFAP‐cre/ERT2)505Fmv/J], *Lrp6‐flox* [B6; 129S‐Lrp6^tm1.1Vari^/J] mice obtained from Jackson Laboratory. Mouse colonies were maintained and bred in the Animal and Plant Care Facility of The Hong Kong University of Science and Technology (HKUST) and the Laboratory Animal Services Centre of The Chinese University of Hong Kong (CUHK). All animal experimental protocols were approved both by the Animal Ethics Committees at both HKUST and CUHK; and their care was in accord with the institutional and Hong Kong guidelines.

Brain tissues were isolated by anesthetizing adult mice with intraperitoneal administration of 1.25% (vol/vol) Avertin at a dosage of 30 mL kg^−1^ body weight. The heart of each mouse was then surgically exposed, the left chamber was catheterized, and the right atrium was opened. Chilled physiological saline was perfused trans‐cardially for 3 min to remove blood from the body. After perfusion, the cranial bones were opened; cortex and cerebellum were harvested, snap‐frozen in liquid nitrogen, and stored at −80 °C until use. For intranasal administration of targeted supplements, the process was performed on lightly anesthetized mice. Each mouse was placed on a sterile surgical pad and lightly stretched out to better hold the scruff. With a firm grip on the scruff, the mouse was turned on its back while allowing the mouse to breathe and be comfortable. With the neck and chin flat and parallel to the pad, the tip of the pipettor containing the sample was placed near the left nostril of the mouse at a 45° angle, and about 5 µL of sample was administered to the nostril with a 2–3 s interval in between for a total of 10 µL/nostril. The mouse was held in this position for 5 s or until it regained consciousness, then the administration step was repeated for the other nostril for a total of 20 µL/mouse. After the mouse had received all drops, the animals were kept restrained on its back until the material disappeared into the nares and then returned back to its cage until the entire treatment plan was finished.

For blood plasma collection, blood samples were taken from the tail vein prior sacrifice of the animals; and subjected to centrifugation at 12 000 revolutions per minute for 15 min at 4 °C. Plasma were obtained from supernatant fraction. Cerebrospinal fluid was collected from the cisterna magna prior sacrifice using a previously established method.^[^ ^51^ ^]^ Glass capillary tubes (Sutter Instrument; borosilicate glass B100‐75‐10) were prepared on a Sutter P‐87 flaming micropipette puller (heat box set at 300 and the pressure index set at 330) and trimmed so that the inner diameter of tapered tips was about 0.5 mm. Mice were anesthetized by intraperitoneal administration of 1.25% (vol/vol) Avertin at a dosage of 30 milliliters per kilogram body weight. For each individual mouse, the skin near the neck was first shaved, and then the body was placed prone on the stereotaxic instrument with direct contact to a heating pad. Once the head was secured with the head adaptors, the surgical site was swabbed with 10% povidone iodine, followed by 70% ethanol, and a sagittal incision of the skin was made inferior to the occiput. Under a dissection microscope, subcutaneous tissue and muscles (m. biventer cervicis and m. rectus capitis dorsalis major) were separated by blunt dissection with forceps. Then, the mouse was laid down so that the head was at a nearly 135° angle to the body. Under the dissection microscope, the dura mater of the cisterna was blotted dry with a sterile cotton swab and penetrated with a capillary tube to reach the cisterna magna. When a notable change in the resistance to the capillary tube occurred following insertion, the CSF was collected into the capillary tube. The capillary tube was carefully removed, and CSF was ejected from the capillary tube with a syringe into a 1.5‐mL tube, and frozen immediately on dry ice until further assays.

### Human Neural‐Progenitor Cells Derived Neurons and Mouse Primary Neuronal Culture

Human ReNcell CX Human Progenitor Cell line derived from the cortical region of the human fetal brain were obtained from Sigma, and cultured following manufacturer's instructions. Neural progenitor cells (NPC) were plated on sterile coverslips in 6‐ or 12‐well plates and coated with poly‐L‐ornithine for 1 h at room temperature. Coated coverslips were washed three times with sterile water and dried for 30 min. Subsequently, a 100 µL drop of laminin solution (50 µg mL^−1^ in water) was placed in the middle of each coverslip, incubated for 15–30 min at 37 °C/5% CO_2_ and then placed with 100 µL drop of DMEM until plating of NPS. Immediately before plating NPCs were washed with Dulbecco's phosphate‐buffered saline and dissociated with collagenase (100 U mL^−1^). One fully confluent 10 cm dish of NPCs was divided over a 12‐well plate. A 100 µL drop of NPC cell suspension was placed on the lamin‐coated spot for 1 h to allow for attachment of NPCs on coverslips in neural differentiation medium (Neurobasal medium, 1% N2 supplement, 2% B27‐RA supplement, 1% minimum essential medium/non‐essential amino acid, 20 ng mL^−1^ brain‐derived neurotrophic factor (ProSpec Bio), 20 ng mL^−1^ glial cell‐derived neurotrophic factor (ProSpec Bio), 1 µm dibutyryl cyclic adenosine monophosphate (Sigma), 200 µm ascorbic acid (Sigma), 2 µg mL^−1^ laminin, and 1% penicillin/streptomycin). After 1 h, 900 µL of neural differentiation medium was added to each well. Cells were refreshed with medium three times per week. During weeks 1–4, the medium was fully refreshed. After 4 weeks of neural differentiation, only half of the volume of medium per well was refreshed. Cells were maintained for 8–10 weeks after plating of NPC. Differentiation into neurons was performed in Neurobasal medium supplemented with B27 Serum‐free supplement and GlutaMax‐1 supplement for 45 days.

Mouse embryonic cortical neurons were isolated by standard procedures.^[^ ^52^ ^]^ Gravid females were killed on the 16th day of gestation and E16.5 embryos collected in ice‐cold PBS‐glucose. The cortical lobes were removed, following which the meninges were removed and the cortices were placed in 1× trypsin solution for 10 min, with manual shaking for 5 min. After digestion, an equal volume of DMEM with 10% (vol/vol) FBS was added to inactivate the trypsin. Samples were then centrifuged at 2500 rpm for 5 min. Supernatant was removed, followed by transferring the pellet to fresh Neurobasal medium supplemented with B‐27, penicillin/streptomycin (1×) and L‐glutamine (2 mm; GlutaMAX, Invitrogen) prior to gentle resuspension. Tissue was triturated ten times through a 5 mL pipette and allowed to settle to the bottom of a 15‐mL conical tube. Dissociated cells in solution above the pellet were removed. Surviving cells were identified by trypan blue exclusion and counted before plating on poly‐L‐lysine‐coated (0.05 mg mL^−1^) glass coverslips. Unless otherwise specified, cells were plated in 24‐well plates at 50 000 cells per well and allowed to mature for over 7–10 days in vitro (DIV) before transfection or lentiviral transduction. For other experiments, cells were grown for a minimum of 14 days in vitro (DIV14) before any drug‐treatment experiments. Every 3 days half of the culture medium was replaced with an equal volume of fresh medium to sustain the culture.

### Human Neural‐Progenitor Cells Derived Astrocytes and Mouse Primary Astrocytes

Human ReNcell CX Human Progenitor Cell line derived from the cortical region of the human fetal brain were obtained from Sigma, and cultured following manufacturer's instructions. NPS were plated at 50–60% confluence on poly‐ornithine‐laminin‐coated dishes in NSC medium which consist of neural medium containing B27 supplement (1×), nonessential amino acids (1×), GlutaMAX (1×), Anti‐Anti(1×) (or 50 µg mL^−1^ Penn‐Strep) (all from Invitrogen), and 20 ng mL^−1^ of basic fibroblast growth factor (bFGF; Sigma). The next day, cells were switched to fresh NSC medium containing 5 ng mL^−1^ CNTF, 10 ng mL^−1^ BMP (PEPROTECH), and 8 ng mL^−1^ bFGF in presence or absence of 1% fetal bovine serum (FBS), or to StemPro medium with supplement containing 10 ng mL^−1^ of Activin A, 10 ng mL^−1^ of Heregulin 1*β*, and 200 ng mL^−1^ of IGF analog (all from Invitrogen). The medium was changed every other day and cells were passaged at least five to six times during the differentiation process.

In mouse primary astrocyte culture, we have adopted the culturing protocol developed by the Barres lab,^[^ ^53^ ^]^ which differs from the traditional protocol which is restricted to the usage of neonatal mouse brains as the source and results in flattened, fibroblast‐like “astrocytes” (also known as “astrocyte progenitor cells”). For mouse cultures, purification of ultrapure brain cortex astrocytes from adult animals, the experimental step contains three major steps, including tissue dissociation, additional myelin removal, and astrocyte isolation as previously reported.^[^ ^54^ ^]^ For tissue dissociation, both adult male and female mice were used. Brain cortical tissue was dissociated using the Neural Tissue Dissociation Kit P (papain) (Miltenyi Biotec.). Following enzymatic digestion, cells were released using mechanical trituration with 10 mL serological pipettes (three rounds of ten strokes each). Freshly dissociated cells were then passed through a 20‐µm Nitex filter to remove remaining tissue clumps. Myelin removal using equilibrium density centrifugation was then performed. With 90% Percoll PLUS in 1 × Hank's balanced salt solution (HBSS) with calcium and magnesium added to the cell suspension, a final Percoll concentration of 24% was achieved. DNase I was then added (1250 units per 10 mL of suspension), and the suspension was mixed and then spun down at 300 × g for 11 min at room temperature in a Hettich 320R universal centrifuge. The cell‐containing pellet was resuspended in 0.5% BSA in PBS without calcium and magnesium (Thermo Fisher Scientific). Additional myelin removal step was performed with Myelin Removal Beads II (Miltenyi Biotec.) according to the manufacturer's instructions along with the LS magnetic column (Miltenyi Biotec.). The flow‐through was subsequently collected and made to proceed to astrocyte isolation with the ACSA‐2 kit (Miltenyi Biotec.) according to the standard protocol, using two runs of enrichment on consecutive MS columns (Miltenyi Biotec.). Portion of enriched astrocytes were subjected purity testing, short‐term 24–72‐h culture or directly used for various assays.

### Neuron‐Astrocytes Co‐Culture System

Both human‐ and mouse‐specific neuron‐astrocyte co‐culture systems were set up as described previously. For these experiments, astrocytes were plated on the top side of the poly‐L‐lysine‐coated polycarbonate membranes of the Transwell inserts (2 × 10^4^ per insert) and cultured in detail as mentioned above. At 24 h before combining the cultures; the original culturing medium was switched to that of neurons (of the same species) prior combining the cultures. Transwell inserts with attached astrocytes were transferred into 12‐well plates and positioned 2 mm above the neuronal monolayer growing on the bottom of the well. Pores in the polycarbonate membranes of the Transwell insert allow for free passage of astrocyte‐derived molecules. The co‐culture was maintained together in respective custom neuronal culturing medium with various conditions of glutamine and BCAAs supplementation (details see different assays) for 72 h before commencing experiments.

### Lentivirus Production and Transduction

Lentivirus stocks were produced as previously described with slight modifications. Human embryonic kidney 293FT cells (Invitrogen) were transfected using Lipofectamine 2000 (Thermo Fisher) with the expression of two helper plasmids: psPAX2 (Addgene #12260) and pMD2.G (Addgene #12259) Ten micrograms of the transfer vector, 5 µg psPAX2 and 5 µg pMD2.G of DNA were used per 10‐cm plate. Forty‐eight hours after transfection, the supernatants of four plates were pooled, centrifuged at 780 × g for 5 min, filtered through a 0.45‐µm pour size filter, and further centrifuged at 24 000 rpm for 2 h. The resulting pellet was re‐suspended in 100 µL of PBS. Lentivirus titration was performed on DIV5‐6 at titer range 10^7^ IU/mL. In most experiments, cultures were infected overnight, rinsed twice with virus‐free medium the next morning and incubated in normal culturing medium for another 48–72 h prior assay.

### Ectopic APOE Expression

Lentiviral vectors expressing APOE3 or APOE4 (Morris et al., in preparation) were constructed by cloning cDNA from induced human neurons by PCR with primers specific for NM_00041. The E3 sequences was mutagenized to E4 using the NEBaseChanger Kit (New England Biolabs). Coding sequences were inserted into FUGW lentiviral backbone, along with a T2A‐linked mCherry gene, using Gibson assembly (HiFi DNA Assembly Kit, New England Biolabs).

### Colorimetric Assay

Cellular contents of pyruvate (K609), succinate (K649), fumarate (K633), glutamine (K556), glutamate (K629), and acetyl‐CoA (K317) were measured using kits purchased from BioVision. Intracellular calcium levels were measured using kits purchased from BioVision (K380). Sample PKC activities were measured using kits purchased from ENZO (ADI‐EKS‐420A). Ammonia (AA0100), Urea (MAK006), and glutathione (CS0260) assay kits were purchased from Sigma Aldrich. ROS/Superoxide detection assay kit was purchased from Abcam. All assays were performed according to the manufacturer's instructions.

### Live Imaging of Cytosolic Calcium Ions

Primary mouse astrocytes cultured on coated 35‐mm glass‐bottom dishes was transfected with 1 µg of plasmid DNA of pcDNA3‐Cyto‐GCaMP3 (Addgene #64853) and 4 µL of lipofectamine LTX and 0.4 µL Plus reagent (Life Technologies) for 4 h. After transfection, cells were allowed to recover for 24 h, then prior to imaging, culture medium was changes to HBSS with 25 mH HEPES. Live imaging was performed on an inverted Leica Sp8 confocal microscope for 48 h, which was equipped with Leica HyD hybrid detector and visualized through a HC PL APO CS2 63× (1.40) NA oil‐immersion objective. Image acquisition was controlled by the LAS X, with the sequential scanning program. A filter set at 420 nm (excitation), 525 nm (emission) and 495 (dichroic) was used. Signal intensity in the region of interest (ROI) along time was analyzed on the LAS X software.

### Metabolic Fuel Flux Assays

Glycolytic Stress and Mito Fuel Flex Tests were performed on XFe24 Bioanalyzer (Agilent). All assays were performed following manufacturer's protocols except with slight modifications as mentioned below. In brief, the test inhibits the import of four major metabolic substrates (pyruvate, fatty acids, glutamine and/or BCAAs) with mitochondrial pyruvate carrier inhibitor UK5099 (2 µm); carnitine palmitoyl transferase 1A inhibitor etomoxir (4 µm); glutaminase inhibitor BPTES (3 µm); or cytosolic branched chain amino acid transferase inhibitor 2 (BCATci‐2; 5 µm). This test determines cellular dependence and capacities on each of the metabolites to fuel mitochondrial metabolism by inhibiting the individual substrate import into the metabolic network. Baseline OCR was monitored for 18 min followed by sequential inhibitor injections (i.e., Treatment 1 or 2) with OCR reading for 1 h following each treatment. The inhibitor treatments and calculation are shown below:

**Table:** Fuel Dependence

Metabolite test	Treatment 1	Treatment 2
Glucose/pyruvate dependence	UK5099	Etomoxir + BPTES + BCATci‐2
Glutamine dependence	BPTES	Etomoxir + UK5099 + BCATci‐2
Fatty acid dependence	Etomoxir	BPTES + UK5099 + BCATci‐2
BCAA dependence	BCATci‐2	Etomoxir + UK5099 + BPTES

Dependency (%) = [(Baseline OCR‐target inhibitor OCR) / (Baseline OCR‐all inhibitors OCR)] × 100%

**Table:** Fuel Capacities

Metabolite test	Treatment 1	Treatment 2
Glucose/pyruvate capacity	Etomoxir + BPTES + BCATci‐2	UK5099
Glutamine capacity	Etomoxir + UK5099 + BCATci‐2	BPTES
Fatty acid capacity	BPTES + UK5099 + BCATci‐2	Etomoxir
BCAA capacity	Etomoxir + UK5099 + BPTES	BCATci‐2

Capacity (%) = [1 − (Baseline OCR‐other 2 inhibitors OCR) / (Baseline OCR‐all inhibitors OCR)] × 100%

### Glycolysis Stress Test

The XF Glycolysis Stress Test (Seahorse Bioscience, Agilent) was used to assess glycolysis function in cells, which was conducted using the XFe24 Analyzer. By directly measuring ECAR, the kit provided a standard method to assess the following key parameters of glycolysis flux: glycolysis, glycolytic capacity, and glycolytic reserve, in addition to non‐glycolytic acidification. Cells were seeded at a density of 45 000 cells/well. Cells were first incubated in pyruvate‐free glycolytic assay medium for 1 h prior to the first injection of a saturated concentration of glucose (final concentration: 10 mm). The cells catabolize glucose into pyruvate via the glycolysis pathway, producing ATP, nicotinamide‐adenine dinucleotide (reduced form), water, and protons. The discharge of protons into surrounding medium leads to a sudden increase in ECAR, which was used to define the basal glycolytic capacity. The second injection was oligomycin (final concentration: 1 µm), which may divert energy production to glycolysis by restricting mitochondrial ATP production. Consequently, the sharp increase in ECAR indicates the level of glycolytic capacity. The final injection was 2‐deoxy‐glucose (2‐DG; final concentration: 50 mm), which is a glucose analog that inhibits glycolysis through competitive binding with glucose hexokinase; the first enzyme in the glycolytic pathway. The resulting decrease in ECAR confirmed that the ECAR produced in the experiment was caused by glycolysis. The gap between glycolytic capacity and glycolysis was defined as the glycolytic reserve. The ECAR prior to glucose injection is referred to as non‐glycolytic acidification and may occur due to additional processes in the cell.

### Assessment of Mitochondrial Function

The mitochondrial OCR in both primary neurons and human fibroblasts were assessed using a Seahorse Bioscience XFe24 analyzer (Agilent) in combination with the Seahorse Bioscience XF Cell Mito Stress Test assay kit. In this assay, sequential additions of the ATP synthase inhibitor, oligomycin, the mitochondrial uncoupler, FCCP, and the complex I + II inhibitors rotenone + antimycin A provide insight into different aspects of mitochondrial function, as described in the text. Experiments were performed on intact adherent cells.

### Quantitative RT‐PCR (qPCR) Analysis

Total cellular RNA was purified from brain tissues or cultured cells using RNease kit (Qiagen) following the manufacturer's protocol. For quantitative real‐time PCR (qPCR), RNA was reverse‐transcribed using the High‐Capacity cDNA Reverse Transcription Kit (Applied Biosystems) according to the manufacturer's instructions. The resulting cDNA was analyzed by qRT‐PCR using SYBR Green PCR Master Mix (Applied Biosystems). All reactions were performed in a Roche LightCycler (LC) 480 instrument using the following protocol: pre‐incubation at 95 °C for 15 min (1 cycle), denaturation at 94 °C for 15 s, annealing and extension at 55 °C for 30 s (40 cycles), and melting at 95 °C for 5 s, 65 °C for 60 s and 95 °C continued (1 cycle) followed by cooling at 40 °C for 30 s. The specificity of the primers was confirmed by observing a single melting point peak. qPCR efficiency was calculated from the slope between 95 and 105% with co‐efficiency of reaction *R* ^2^ = 0.98–0.99. A total of 7–9 biological replicates × at least 3 technical replicates were performed for each treatment group. Data was analyzed using the comparative Ct method (ΔΔCt method).

### Wnt Pathway‐Related Phosphoprotein Profiling

Assay was performed according to the manufacturer's protocol and as that published previously.^[^ ^55^ ^]^ The Wnt pathway phospho‐ antibody array (PNT277) from Full Moon Biosystems Inc. (Sunnyvale, CA) contains 227 antibodies spotted in six replicates onto 3D polymer‐coated glass slide. The antibody array experiment was performed following manufacturer's directions. In brief, cell lysates obtained from mature neurons or astrocytes were biotinylated with the antibody array assay kit (Full Moon Biosystems, Inc.). The antibody microarray slides were first blocked in a blocking solution (Full Moon Biosystems, Inc.) for 30 min at room temperature, rinsed with Milli‐Q grade water for 3–5 min, and dried by centrifugation. The slides were then incubated with the biotin‐labelled cell lysate (50 µg of protein) in coupling solution (Full Moon Biosystems, Inc.) at room temperature for 2 h. The array slides were washed four to five times with 1× Wash Solution (Full Moon Biosystems, Inc.) and rinsed extensively with Milli‐Q grade water before detection of bound biotinylated proteins using Cy3‐conjugated streptavidin. Slides were scanned on a GenePix 4000B scanner, and the images were analyzed with GenePix Pro6.0 (Molecular Devices, Sunnyvale, CA). The fluorescence signal of each antibody was obtained from the intensity of antibody spot after normalized with the blank signal (spot without antibody). Only hits simultaneously fulfill Log_2_(Fold change) ±0.5 and *p*‐values <0.05 are considered significant.

### Immunocytochemistry and Immunohistochemistry

For immunocytochemistry, primary neuronal and astrocyte cultures were grown on 13‐mm coverslips (Marienfeld) in 24‐well plates, whereas for immunohistochemistry, 10 µm cryo‐sections of frozen mouse brains were used. Samples were fixed with fresh 4% (wt/vol) paraformaldehyde (Sigma‐Aldrich) for 10 min, washed and followed by permeabilization with 0.3% Triton‐X100 in PBS for 10 min. After blocking with 5% (wt/vol) BSA in PBS for 1 h, primary antibodies were added and incubated overnight at 4 °C. The following day, coverslips were washed three times (10 min each) with PBS. After rinsing, secondary antibodies were applied for 1 h at room temperature followed by three additional washes with PBS. The coverslips were then inverted and mounted on glass slides with ProLong Gold Antifade Reagent (Life Technologies). Immuno‐florescence was analyzed, and Z‐stack maximum projected images were photographed using TCS SP8 confocal microscope (Leica Microsystems Inc.)

### Co‐Immunoprecipitation and SDS‐PAGE‐Western Blotting

Isolated brain tissues or cell pellets were homogenized in RIPA buffer (Millipore) with 1× complete protease inhibitor mixture (Roche) and 1× PhosSTOP phosphatase inhibitor mixture (Roche) on ice then centrifuged for 10 min at 18 400 × g to remove large debris. The protein concentration of the supernatant was determined by Bradford Assay (Bio‐Rad). For co‐immunoprecipitation, 1 mg of the total cell lysates was first incubated with control IgG (Santa Cruz Biotechnology) for 30 min, pre‐cleared with 50 µL Dynabeads Protein G (Invitrogen), and then incubated with various antibodies overnight at 4 °C, using the suggested dilutions from the product datasheets. Beads bound with immune complexes were collected by DynaMag‐2 (Life Technologies) and washed three times before elution in 90 µL if buffer containing 0.2 m Glycine‐HCl, pH 2.5, which was neutralized with 10 µL of neutralization buffer (1 m Tris‐HCl [pH 9.0]). The eluates were subjected to 9–15% SDS‐PAGE and Western blot analysis.

For SDS‐PAGE (polyacrylamide gel electrophoresis), 100 µg of proteins derived from cell or tissue lysates were prepared in 5× sample buffer (10% w/v SDS; 10 mm beta‐mercaptoethanol; 20% v/v glycerol; 0.2 m Tris‐HCl, pH6.8; 0.05% Bromophenol Blue). With a Bio‐Rad system, separating gels of different acrylamide percentages (6%‐15%) were prepared with the following components in double distilled water: acrylamide/bis‐acrylamide (30%/0.8% w/v), 1.5 m Tris (pH = 8.8), 10% (w/v) SDS, 10% (w/v) ammonium persulfate and TEMED) and a 5% stacking gel (5 mL prep: 2.975 mL Water; 1.25 mL 0.5 m Tris‐HCl, pH6.8; 0.05 mL 10% (w/v) SDS, 0.67 mL acrylamide/bis‐acrylamide (30%/0.8% w/v), 0.05 mL 10% (w/v) ammonium persulfate, and 0.005 mL TEMED) were prepared. Samples were run in SDS‐containing running buffer (25 mm Tris‐HCl; 200 mm glycine and 0.1% [w/v] SDS) until the dye front and the protein marker reached the foot of the glass plate. Standard immune‐blotting procedures were used, which include protein transfer to polyvinylidene difluoride (PVDF) membranes, blocking with non‐fat milk, and incubation with primary and secondary antibody, followed by visualization with the SuperSignal West Dura/Femto Chemiluminescent Substrate (ThermoFisher Scientific).

### Luciferase Assay for Nuclear Activities of Transcription Factor

Nuclear activities of endogenous *β*‐catenin were analyzed by the M50 Super 8× TOPflash (Addgene #12456)/FOPflash (Addgene #12457) reporter system; whereas that of NFAT was analyzed by the pGL3‐NFAT luciferase reporter (Addgene #17870); HIF1a by HRE‐luciferase reporter (Addgene #26731); SREBP by pSynSRE‐T‐Luc (Addgene #60444)/pSynSRE‐Mut‐T‐Luc (Addgene #60490) luciferase reporter system; PPAR by PPRE X 3‐TK‐Luc (Addgene #1015) reporter; STAT3 by the STAT3 Reporter Kit (BPS Bioscience, #79730); and YY1 by the YY1 Luciferase reporter vector (Panomics, LR0090). To normalize transfection efficiency in the reporter assays, cells were co‐transfected with pRL‐TK plasmid, which contains a functional *Renilla* luciferase gene cloned downstream of a herpes simplex virus thymidine kinase promoter (Promega). The assay was carried out as described above with the FOPflash reporter as a negative control. Luminescence was measured using a Bright‐GloTM Luciferase Assay System (Promega) on a luminometer (Berthold Technologies), and normalized to control *Renilla* Luciferase signal. Luciferase activity was calculated against the negative control signals and fold differences were compared among groups in separate assays.

### Untargeted Metabolome Analysis by Capillary Electrophoresis Time‐Of‐Flight Mass Spectrometry (CE‐TOFMS) and Liquid Chromatography (LC)‐TOFMS

Metabolome analyses were performed in mouse frontal cortex tissue or astrocytes using CE‐TOFMS for both cationic and anionic metabolites on the basis of Human Metabolome Technologies’ (HMT) standard library. Samples were sent to HMT where their weights were first measured. For CE‐TOFMS preparation, samples were mixed with 1500 µL of 50% acetonitrile in water (v/v) containing internal standards (10 µm) and homogenized by a homogenizer (1500 rpm, 120 s × 1 times). The supernatant (400 µL) was then filtrated through 5‐kDa cut off filter (ULTRAFREE‐MC‐PLHCC, HMT) to remove macromolecules. The filtrate was centrifugally concentrated and resuspended in 50 µL of ultrapure water immediately before measurement. Whereas for LC‐TOFMS preparation, weighted samples were mixed with 300 µL of 1% formic acid in acetonitrile (v/v) containing internal standards (10 µm) and homogenized by a homogenizer (1500 rpm, 120 s × 2 times). The mixture was yet again homogenized after adding 100 µL of Milli‐Q water and then centrifuged (2300 × g, 4 °C, 5 min). After the supernatant was collected, 300 µL of 1% formic acid in acetonitrile (v/v) and 100 µL of MilliQ‐water were added to the precipitation. The homogenization and centrifugation were performed as described previously, and the supernatant was mixed with previously collected one. The mixed supernatant was filtrated through 3‐kDA cut‐off filter (NANOCEP 3K OMEGA, PALL Corporation, Michigan, USA) to remove proteins and far filtrated through column (Hybrid SPE phospholipid 55261‐U, Supelco, Bellefonte, PA, USA) to remove phospholipids. The filtrate was desiccated and resuspended in 200 µL of 50% isopropanol in Milli‐Q water (v/v) immediately before the measurement.

### CE‐TOFMS Measurement

The compounds were measured in the Cation and Anion modes of CE‐TOFMS based metabolome analysis in the following conditions. Samples were diluted in 2 folds for measurement, to improve analysis qualities of the CE‐MS analysis.

**Table:** Cationic Metabolites (Cation Mode) Parameters

Device	
Agilent CE‐TOFMS System (Agilent Technologies Inc.) Machine No.12	
Capillary: fused silica capillary i.d. 50 µm × 80 cm	
**Analytical condition**	
Run buffer	Cation buffer solution (p/n: H3301‐1001)
Rinse buffer	Cation buffer solution (p/n: H3301‐1001)
Sample injection	Pressure injection 50 mbar, 5 s
CE voltage	Positive, 30 kV
MS ionization	ESI Positive
MS capillary voltage	4000 V
MS scan range	m/z 50–1000
Sheath liquid	HMT sheath liquid (p/n: H3301‐1020)

**Table:** Anionic Metabolites (Anion Mode) Parameters

Device	
Agilent CE‐TOFMS system (Agilent Technologies Inc.) machine No. 5	
Capillary: fused silica capillary i.d. 50 µm × 80 cm	
**Analytical condition**	
Run buffer	Anion buffer solution (p/n: I3302‐1023)
Rinse buffer	Anion buffer solution (p/n: I3302‐1023)
Sample injection	Pressure injection 50 mbar, 22 s
CE voltage	Positive, 30 kV
MS ionization	ESI negative
MS capillary voltage	3500 V
MS scan range	m/z 50–1000
Sheath liquid	HMT sheath liquid (p/n: H3301‐1020)

### LC‐TOFMS Measurement

The compounds were measured in the Positive and Negative modes of LC‐TOFMS based metabolome analysis in the following conditions. Samples were diluted in onefold for measurement, to improve analysis qualities of the CE‐MS analysis.

**Table:** Cationic Metabolites (Positive Mode) Parameters

Device	
LC system: Agilent 1200 series RRLC system SL (Agilent Technologies Inc.)	
Column: ODS column 2 × 50 mm, 2 µm	
MS system: Agilent LS/MSD TOF (Agilent Technologies Inc.) machine No. 9	
**Analytical condition**	
Column Temp:	40 °C
Mobile phase A	H2O/0.1% HCOOH
Mobile phase B	Isopropanol: Acetonitrile: H2O (65:30:5)/0.1% HCOOH, 2 mm HCOONH_4_
Flow rate	0.3 mL min^−1^
Run time	20 min
Post time	7.5 min
Gradient condition	0–0.5 min: B 1%, 0.5–13.5 min: B 1–100%, 13.5–20 min: B 100%
MS ionization mode	ESI Positive
MS Nebulizer pressure	40 psi
MS dry gas flow	10 L min^−1^
MS dry gas temp	350 °C
MS capillary voltage	4000 V
MS scan range	m/z 100–1700
Sample injection	1 µL

**Table:** Anionic Metabolites (Negative Mode) Parameters

Device	
LC system: Agilent 1200 series RRLC system SL (Agilent Technologies Inc.)	
Column: ODS column 2 × 50 mm, 2 µm	
MS system: Agilent LS/MSD TOF (Agilent Technologies Inc.) machine No. 9	
**Analytical condition**	
Column Temp:	40 °C
Mobile phase A	H2O/0.1% HCOOH
Mobile phase B	Isopropanol: Acetonitrile: H2O (65:30:5)/0.1% HCOOH, 2 mm HCOONH_4_
Flow rate	0.3 mL min^−1^
Run time	20 min
Post time	7.5 min
Gradient condition	0–0.5 min: B 1%, 0.5–13.5 min: B 1–100%, 13.5–20 min: B 100%
MS ionization mode	ESI Negative
MS Nebulizer pressure	40 psi
MS dry gas flow	10 L min^−1^
MS dry gas temp	350 °C
MS capillary voltage	3500 V
MS scan range	m/z 100–1700
Sample injection	1 µL

Peaks detected in both CE‐TOFMS and LC‐TOFMS were extracted using automatic integration software (MasterHands ver. 2.17.1.11 developed at Keio University) in order to obtain peak information including m/z, migration time (MT) in CE, retention time (RT) in LC, and peak area. The peak area was then converted to relative peak area by the following equation. The peak detection limited was determined based on signal‐noise ratio = 3

(1) $$\begin{array}{ccc} \mathrm{Relative}\;\mathrm{Peak}\;\mathrm{Area} & = & \mathrm{Metabolite}\;\mathrm{Peak}\;\mathrm{Area}/\mathrm{Internal}\;\mathrm{Standard}\;\mathrm{Peak}\;\mathrm{Area}\\ & & \times \;\mathrm{Sample}\;\mathrm{Amount} \end{array}$$

Putative metabolites were then assigned from HMT's standard library and Known–Unknown peak library on the basis of m/z and MT or RT. The tolerance was ±0.5 min in MT and ±0.3 min in RT, ±10 ppm (CE‐TOFMS) and ±25 ppm (LC‐TOFMS) in m/z. If several peaks were assigned the same candidate, the candidate was given the branch number.

(2) $$\begin{array}{ccc} \mathrm{Mass}\;\mathrm{error}\;\left(\mathrm{ppm}\right) & = & \left(\mathrm{Measured}\;\mathrm{Value}-\mathrm{Theoretical}\;\mathrm{Value}\right)/\\ & & \mathrm{Measured}\;\mathrm{Value}\times 10^{6} \end{array}$$

Subsequent absolute quantification was performed in target metabolites. All the metabolite concentrations were calculated by normalizing the peak area of each metabolite with respect to the area of the internal standard and by using standard curves, which were obtained by single‐point (100 or 50 µm) calibrations. Significantly changed metabolites (with Log_2_FC ± 0.5; *p* <0.05) were enriched and analyzed by the Metabolite Set Enrichment Analysis (MESA) or the Joint Pathway Analysis module (with KEGG metabolic gene expression data) on MetaboAnalyst (https://www.metaboanalyst.ca/MetaboAnalyst/ModuleView.xhtml).

### Stable‐Isotope Labelled Leucine and Glutamine Metabolite Tracing

Metabolic fate and catabolic flux of isoleucine (Ile) and glutamine (Gln) were performed as previously reported.^[^ ^56^ ^]^ In primary astrocytes, [^13^C_6_, ^15^N_1_] Ile, or [^13^C_5_] Gln tracing followed by capillary electrophoresis‐time of flight mass spectrometer (CE‐TOF/MS) (Agilent Technologies) were performed. Freshly harvested astrocytes were incubated in the BCAA‐free medium supplemented with 2 mm [^13^C_5_, ^15^N_1_] Ile (608068, Sigma‐Aldrich); or glutamine‐free medium supplemented with 2 mm [^13^C_5_] Gln (605166, Sigma‐Aldrich) and collected at 2 h‐post incubation, cells were washed twice with 10 mL of 5% mannitol aqueous solution, and subsequently incubated with 1 mL of methanol containing 25 µm internal standards (methionine sulfone, 2‐(*N*‐morpholino)‐ethanesulfonic acid (MES) and D‐camphor‐10‐sulfonic acid) for 10 min. Four hundred microliters of the extracts were mixed with 200 µL Milli‐Q water and 400 µL chloroform and centrifuged at 10 000 g for 3 min at 4 °C. Subsequently, 400 µL of the aqueous solution was centrifugally filtered through a 5‐kDa cut‐off filter to remove proteins. The filtrate was centrifugally concentrated and dissolved in 50 µL of Milli‐Q water that contained reference compounds (200 µm each of 3‐aminopyrrolidine and trimesate) immediately before metabolome analysis.

The relative concentrations of all the charged metabolites in samples were measured by CE‐TOFMS, following the methods as previously reported.^[^ ^57^ ^]^ In brief, a fused silica capillary (50 µm internal diameter × 100 cm) was used with 1 m formic acid as the electrolyte. Methanol: water (50% v/v) containing 0.1 µm hexakis (2,2‐difluoroethoxy) phosphazene was delivered as the sheath liquid at 10 µL min^−1^. Electrospray ionization (ESI)‐TOFMS was performed in positive‐ion mode, and the capillary voltage was set to 4 kV. Automatic recalibration of each acquired spectrum was achieved using the masses of the reference standards. [(^13^C isotopic ion of a protonated methanol dimer (2 MeOH + H)]^+^, m/z 66.0632) and ([hexakis (2,2‐difluoroethoxy) phosphazene + H]^+^, m/z 622.0290). Quantification was performed by comparing peak areas to calibration curves generated using internal standardization techniques with methionine sulfone. The other conditions were identical to those described previously.^[^ ^57^ ^]^ To analyze anionic metabolites, a commercially available COSMO(+) (chemically coated with cationic polymer) capillary (50 µm internal diameter × 105 cm) (Nacalai Tesque) was used with a 50 mm ammonium acetate solution (pH 8.5) as the electrolyte. Methanol; 5 mm ammonium acetate (50% v/v) containing 0.1 µm hexakis (2,2‐difluoroethoxy) phosphazene was delivered as the sheath liquid at 10 µL min−1. ESI‐TOFMS was performed in negative ion mode, and the capillary voltage was set to 3.5 kV. For anion analysis, trimesate and CAS were used as the reference and the internal standards, respectively. The other conditions were identical to those described previously.^[^ ^58^ ^]^ MPE of isotopes, an index of isotopic enrichment of metabolites, was calculated as the percent of all atoms within the metabolite pool that are labelled according to the established formula.^[^ ^59^ ^]^

### LIVE/DEAD Cell Viability Assays

The LIVE/DEAD viability/cytotoxicity kit for mammalian cells was purchased from ThermoFisher. The method relies on two probes: calcein AM and ethidium homodimer (EthD‐1). Cultures were washed three times with 500 volumes of Dulbecco's PBS (D‐PBS). Two hundred microliters of the calcein/EthD‐1 working solution (1 and 2 µm, respectively in d‐PBS) were applied to the surface of a coverslip or 35‐mm disposable Petri dish and incubated for 45 min at room temperature in the dark. Imaging was performed on a Leica TCS SP8 confocal laser scanning platform, equipped with Leica HyD hybrid detector and visualized through a HC PL APO CS2 63× (1.40) NA oil‐immersion objective. Image acquisition was controlled by the LAS X, with the sequential scanning program. Calcein was excited at 485 ± 15 nm, and emission was measured at 530 ± 15 nm; EthD‐1 was excited at 530 ± 15 nm with emission measured at 645 ± 15 nm. Quantification of live (green) and dead (red) cells was performed randomly on ten fields from each of three to four blinded samples. The percentage of dead cells was calculated as (number of dead cells/total number of live and dead cells) × 100%.

### Animal Behavioral Tests

All tests were performed as previously described with slight modifications. All mice were housed in groups of five per cage. All behavioral tests were performed during the light phase of the circadian cycle between 09:00 and 17:00. All behavioral testing began by allowing mice to habituate to the testing rooms before tests. Experiments were performed in blind to the genotype when behavioral tests were carried out. Experiments were performed in blind to the genotype or treatments when behavioral tests were carried out. With an overhead camera and the Freeze Frame software (Actimetrics), learning and memory performances were assessed using the Morris water maze, Y‐maze, and novel object recognition test. Followed by these, experimental mice were also subjected to open field test, forced swim test, and rotarod test.

### Morris Water Maze Test

The Morris water maze test was conducted as described previously. Briefly, a blue circular tank (90 cm (diameter)*35 cm (high)) was filled with water (≈22 °C), and a platform (10 cm in diameter) was submerged 1 cm beneath the surface of the water in a target quadrant. The walls surrounding the tank contained bright and contrasting shapes that served as reference cues. Training was conducted over seven consecutive days with four trials/day using an inter‐trial interval of 1–1.5 min. Mice were placed randomly into each of four starting locations for each of four daily training trials. In each trial, mice swam until they found the hidden platform or were gently guided to it by the experimenter if not found within 60 s. Mice remained on the platform for 15 s before returning to the home cage. Daily data were averaged across the four trials. On day 7, a probe trial was conducted, and the hidden platform was removed, mice were placed in the pool and allowed to swim for 60 s. The time spent in each of the four quadrants, and the number of target (platform) area crossings, were recorded.

### Novel Object Recognition Test

The novel‐object recognition test was based on the innate tendency of rodents to differentially explore novel objects over familiar ones. The test was performed as previously described. On the first day, mice were placed into a Plexiglas rectangular cage (40 cm (L) × 40 cm (W) × 40 cm (H)) for 10 min. On the second day, mice were presented with two of the same objects, indicated as A and B. Twenty‐four hours later, the mice were exposed to two different objects: A and C, with C indicating a novel object. All objects were rinsed with ethanol and allowed to dry between trials and before the first trial. All testing and training sessions were videotaped and analyzed in blind to the treatment group of the animals. Object exploration was defined as each instance in which a mouse's nose touched the object or was oriented toward and within 2 cm of the object. Exploratory activity within the experimental arena was measured over a 5‐min trial with a computer timer.

### Open‐Field Test

Mice were placed in the center of an open‐field arena (40 cm (L) × 40 cm (W) × 40 cm (H)) for 10 min. During the experiments, the entire open field was video recorded. Total travel distance and time spent in the center areas of the maze were computed by a Smart 3.0 video tracking system (Panlab, Harvard Apparatus).

### Rotarod Test

Mice were placed on a stationary rotarod (IITC Life Sciences) in a well‐lit room that was then activated and accelerated from 0 to 45 revolutions per minute over 5 min. The latency of mice to fall off the rod or taking one revolution was measured. Trials were repeated four times with intertribal intervals of 30 min over a single day.

### For Forced Swim Test

The forced swim test was used for evaluation of the depressive‐like behaviors. The tanks (15 cm (D) × 30 cm (H)) were filled with tap water set at 22 °C. Mice were placed in the water and their escape‐related mobility behavior was measured for 8 min.

### In Silico Protein Structure Modelling and Docking

With the protein sequence of the human LRP6 protein obtained from the NCBI‐Gene database, the LDLR type A (LA) repeats domain structure (a.a. 1326–1360) was used to predict the corresponding 3D protein structure on the Phyre^2^ engine, using the intensive modelling mode. The predicted structure was compared with other LDL receptor related proteins to confirm its confidence was >99%, followed by docking with APOE3 and APOE4 LA‐interacting domains on ClusPro 2.0 (electrostatic intensive mode) (https://cluspro.bu.edu/login.php). Predicted form of interactions and scores of the first ranked cluster conformation were obtained.

### Electrophysiological Recording

Hippocampal slices were prepared from mice aged 2–4 postnatal weeks. The mice were sacrificed by decapitation after initial anesthesia. Brains were removed from the skulls and placed in ice‐cold artificial cerebrospinal fluid (ACSF) composed of (in mmol L^−1^): NaCl 124, KCl 3.0, CaCl_2_ 2, MgCl_2_ 6, NaH_2_PO_4_ 1.25, NaHCO_3_ 26, and glucose 10 in 95% O_2_ and 5% CO_2_. Transverse hippocampal slices (400 µm) were cut using a Vibroslice (VT 1000S; Leica), transferred to a holding chamber with 95% O_2_ and 5% CO_2_, and stabilized at room temperature for at least 1 h. For recordings, the slices were transferred to a recording chamber, where it was perfused with perfusing ACSF (2 mL min^−1^) at 30–32 °C. The perfusing ACSF solution for standard recording contained (in millimole per liter): NaCl 119, KCl 2.5, CaCl_2_ 2, MgCl_2_ 2, NaHCO_3_ 26, NaH_2_PO_4_ 1 and glucose 10, gassed with 95% O_2_ and 5% CO_2_.

Paired‐pulse stimulation (PPS, pulse intervals of 40 ms, repeated at 0.1 Hz) was delivered with 0.1 ms negative constant current pulses (20–60 µA), via a monopolar tungsten electrode at the Schaffer collaterals. The stimulus intensity of the first pulse was adjusted to induce fEPSP amplitude at about 50% of maximal fEPSP. The fEPSPs were recorded with an Axopatch 200B amplifier in the CA1 apical dendritic layer using a glass micropipette filled with perfusing ACSF or NaCl (1–2 mol L^−1^), with the pipette resistance of 2–5 MΩ. LTP was induced by two successive HFS trains (100 Hz, 100 impulses each, an interval of 10 s). The strength of synaptic transmission was determined by measuring the initial (10–60% rising phase) slope of fEPSPs. LTP in the CA1 area was induced by one train of 100‐Hz stimuli with the same intensity of the test stimulus, and a cut was made between CA1 and CA3 in hippocampal slices to prevent the propagation of epileptiform activity. For recording of eEPSC, a custom‐made bipolar stimulation electrode was position 150–220 µm subjacent to a recorded neuron. Current pulses (50–120 µA in amplitude and 100 µs in pulse duration), generated and isolated by Master‐9 (AMP Instruments Ltd) were used as stimuli and delivered at 0.033 Hz. In most cases, we used a stimulation intensity that could evoke an eEPSC of ≈100–150 pA.

### Quantification Procedures and Statistical Analyses

For each experiment, no statistical methods were used to predetermine sample sizes, but our sample sizes were similar to those reported in recent publications. Data distribution was assumed to be normal, but this was not formally tested. All samples were analyzed, and data collected blinded to the experimental conditions. All experiments were performed in at least three independent occasions. Quantification of cellular morphology parameters was performed in a blinded manner. Analyses of the qPCR data were performed on Prism 8.0 for generating the volcano plot. Differences between groups were analyzed using two‐tailed unpaired Student's *t*‐test (for two groups) or one‐way ANOVA (for more than three groups) for normally distributed data or using a Wilcoxon signed‐rank test for skewed data. Two‐way ANOVA was used to determine the effect of two nominal predictor variables. Gene Ontology analysis was performed using Gene Annotation Tool to Help Explain Relationships (GATHER) platform at UT Health Science Center at Houston (https://changlab.uth.tmc.edu/gather/gather.py) and Gene Set Enrichment Analysis (GSEA) platform at Broad Institute (https://www.gsea‐msigdb.org/gsea/index.jsp). Heatmap was plotted on Morpheus at Broad Institute (https://software.broadinstitute.org/morpheus/). SNP effect on specific gene expression in different brain region analysis was performed on BRAINEAC (http://www.braineac.org). All statistical analyses were performed using GraphPad Prism 8.0 and SigmaStat statistics software package. Log_2_FC ± 0.5 together with *p* <0.05 were considered to indicate statistical significance.

## Conflict of Interest

The authors declare no conflict of interest.

## Author Contributions

H.‐M.C. and R.P.H. designed the research. H.‐M.C., J. K.‐L. S., R.P.H., and T.‐M.L. performed research. H.‐M.C., R.P.H, K.K‐Y C., C.H‐L.H., and K.‐M.K. contributed new reagents and analytic tools. H.‐M.C., J. K.‐L. S., and R.P.H. analyzed the data. H.‐M.C. and R.P.H. wrote the paper.

## Supporting information

Supporting Information

## Data Availability

The data that support the findings of this study are available from the corresponding author upon reasonable request.
