# Supplementary material for: Low‐Density Lipoprotein Receptor‐Related Protein 6 Cell Surface Availability Regulates Fuel Metabolism in Astrocytes
Source: Adv Sci (Weinh). 2021 Jun 27;8(16):2004993. doi: 10.1002/advs.202004993 (PMC8373092; doi:10.1002/advs.202004993)
Supplement: Supplementary file 1 — Supporting Information [file ADVS-8-2004993-s001.pdf]

© 2021 Wiley-VCH GmbH

ADVANCED SCIENCE
Open Access

Supporting Information

for *Adv. Sci.*, DOI: 10.1002/advs.202004993

Low-density lipoprotein receptor-related protein 6 cell surface availability regulates fuel metabolism in astrocytes

Hei-Man CHOW,* Jacquelyne Ka-Li SUN, Ronald P. HART, Kenneth King-Yip CHENG, Clara H.L. HUNG, Tsun-Ming LAU, Kin-Ming KWAN

Supporting Information

Low-density lipoprotein receptor-related protein 6 cell surface availability regulates fuel metabolism in astrocytes

Hei-Man CHOW[1,*]
Jacquelyne Ka-Li SUN[1]
Ronald P. HART[2]
Kenneth King-Yip CHENG[3]
Clara H.L. HUNG[4]
Tsun-Ming LAU[1]
Kin-Ming KWAN[1]

[1]School of Life Sciences, Faculty of Science, The Chinese University of Hong Kong, Hong Kong.
[2]Department of Cell Biology and Neuroscience, Rutgers University, Piscataway, New Jersey, USA
[3]Department of Health Technology and Informatics, The Hong Kong Polytechnic University, Hong Kong
[4]The University Research Facility in Life Sciences, The Hong Kong Polytechnic University, Hong Kong

* To whom correspondence should be addressed:
Hei-Man CHOW
Email: heimanchow@cuhk.edu.hk
Phone: (+852)3943-1530

Contents of supporting information

Supplementary Figure: 1-8
Supplementary Table: 1-11

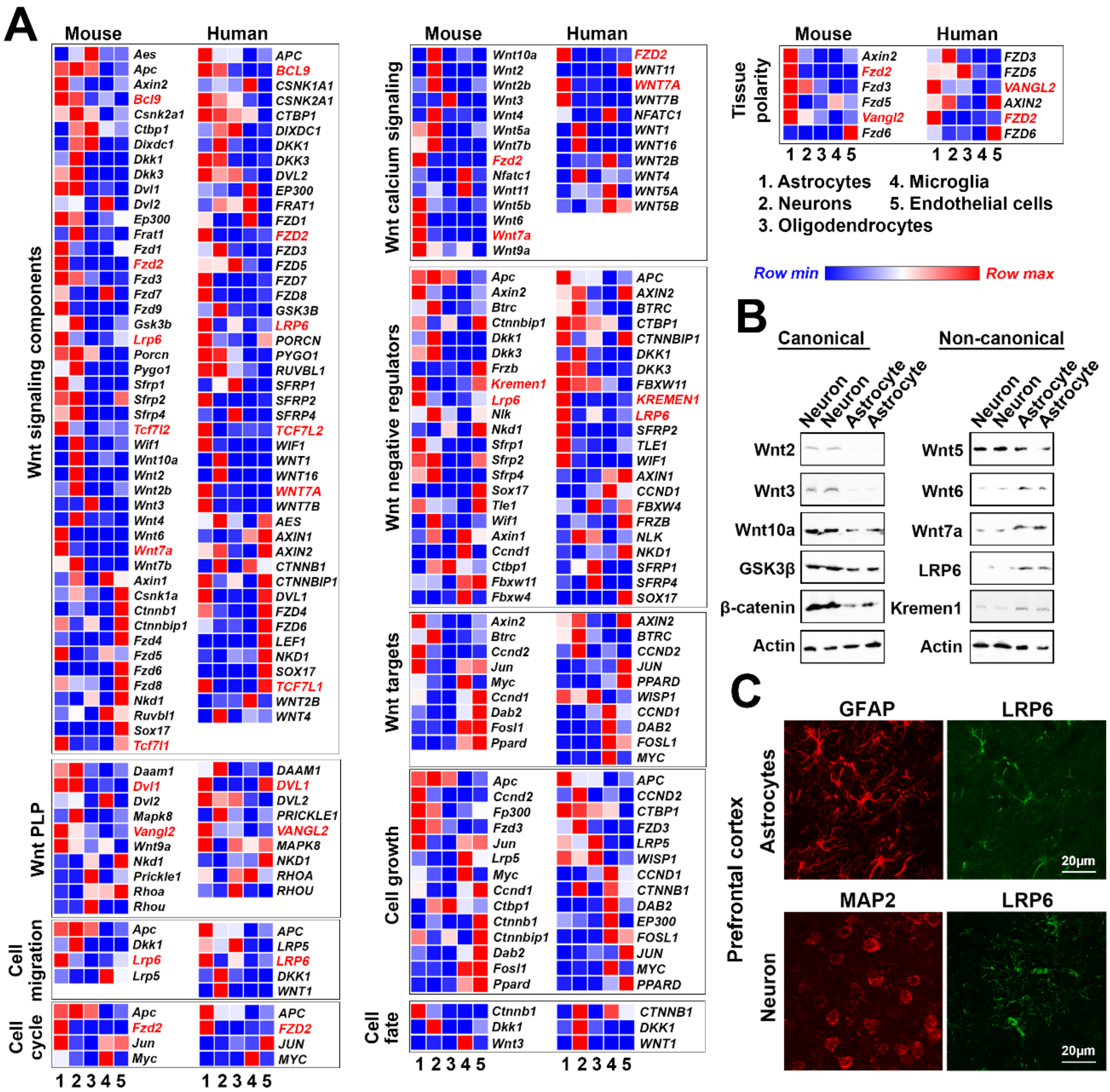

Supplementary Figure 1. Wnt signaling network in the brain.

(A) With reference to the data available on Brain RNA-Seq portal (https://www.brainrnaseq.org), relative expression levels of the complete lists of Wnt signaling components, regulators and downstream targets were compared among various mature brain cell types. Red highlights are genes commonly enriched in both human and mouse astrocytes. (B) Representative immunoblots of the canonical and non-canonical Wnt signaling components in mouse primary cortical neurons and freshly enriched astrocytes (N=6). (C) Representative immunohistochemistry images of the mouse frontal cortex regions showing LRP6 signals co-stained with either GFAP-astrocyte or MAP2-neuronal markers (N=6).

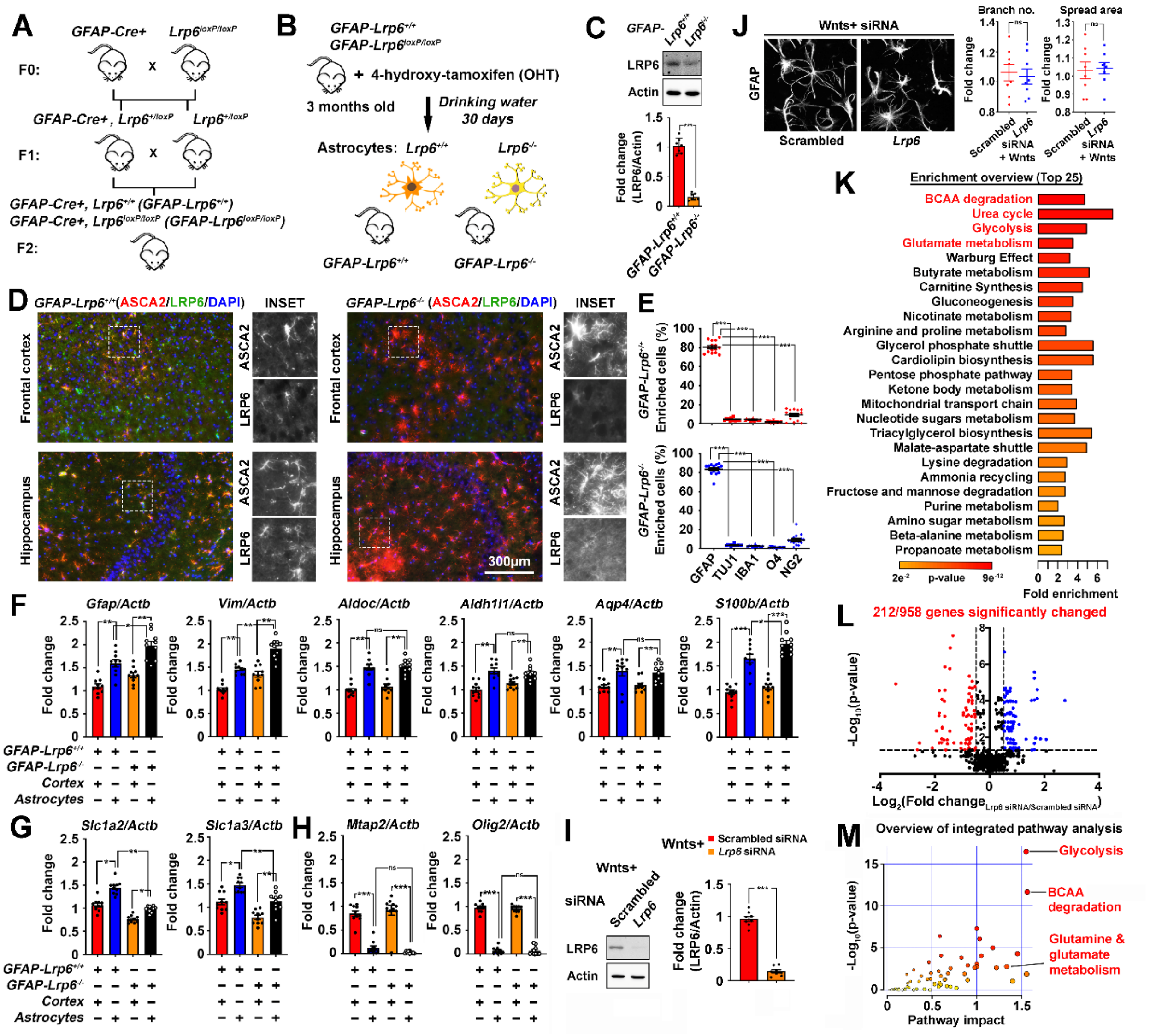

Supplementary Figure 2. Workflows of adult astrocyte-specific *Lrp6*-knockdown and enrichment of primary astrocytes.

(A-B) Schematic diagrams showing (A) the breeding scheme which F0 transgenic mice harboring two-floxed *Lrp6* allele were crossed with mice that express Cre-recombinase under the control of *GFAP* promoter. The double heterozygous (*GFAP-Cre+, Lrp6$^{+/loxP}$*) F1 progeny was then crossbred with the *Lrp6* single-floxed mice (*Lrp6$^{+/loxP}$*) of the same progeny to generate pups of *GFAP-Cre+, Lrp6$^{+/+}$* (*GFAP-Lrp6$^{+/+}$*) and *GFAP-Cre+, Lrp6$^{loxP/loxP}$* (*GFAP-Lrp6$^{loxP/loxP}$*) genotypes. (B) By three months of age, induction of *GFAP*-promoter driven Cre-recombinase was performed through oral administration of 25 μg/ml 4-hydroxy-tamoxifen (OHT) for 30 consecutive days in drinking water, which allows sufficient time to induce the knockout out of *Lrp6* in astrocytes. (C) Representative immunoblots of LRP6 protein level in both cortical tissue of *GFAP-Lrp6$^{+/+}$* and *GFAP-Lrp6$^{-/-}$* mice. Quantification shown on the right (N=7, P<0.0001, two-tailed unpaired t-test). (D) Representative immunohistochemistry images showing the effect of 30 days OHT administration on LRP6 protein expression in astrocytes located at both the frontal cortex and hippocampus regions. (E) Quantification of immunopanning enriched cells immune-labelled

with various brain cell markers (Astrocyte—GFAP; neuron—TUJ1; microglia—IBA1; oligodendrocytes—O4; pericytes—NG2) in enriched astrocyte cultures (N=14; ***P<0.0001; one-way ANOVA). (F-H) Quantitative PCR was performed to characterize various markers of (F) astrocyte cytoskeleton and cytoplasm; (G) astrocyte glutamine-glutamate transporter system; as well as (H) neurons and oligodendrocytes in both cortex tissues and enriched astrocytes harvested from *GFAP-Lrp6*$^{+/+}$ and *GFAP-Lrp6*$^{-/-}$ mice (N=10, ***P<0.0001; **P<0.001; *P<0.01; ns=non-significant; one-way ANOVA). (I) Representative immunoblots of LRP6 protein level in Wnts-treated astrocytes pre-overexpressing Lrp6-specific siRNA for 72 hours. Quantification shown on the right (N=8, P<0.0001, two-tailed unpaired t-test). (J) representative immunocytochemistry images showing the morphologies of Wnts-treated astrocytes pre-overexpressing Lrp6-specific siRNA for 72 hours. Quantification of branch number and spreading area are shown on the right (N=8, P<0.0001, two-tailed unpaired t-test). (K) Global metabolic profiling of Wnts-treated astrocytes pre-overexpressing Lrp6-specific siRNA for 72 hours, followed by the Metabolite Set Enrichment Analysis (MESA) (N=6). Pathways are ranked by significance values. (L) qPCR analyses of 958 mouse KEGG metabolic-related genes expression in the same cells mentioned in (K) (N=6; horizontal dotted line P=0.05; two-tailed unpaired t-test). (M) Integrated metabolites and gene expression analyses was performed on MetaboAnalyst. Top 3 significant pathways with the greatest impact are labelled. Values represent the Mean ± SEM.

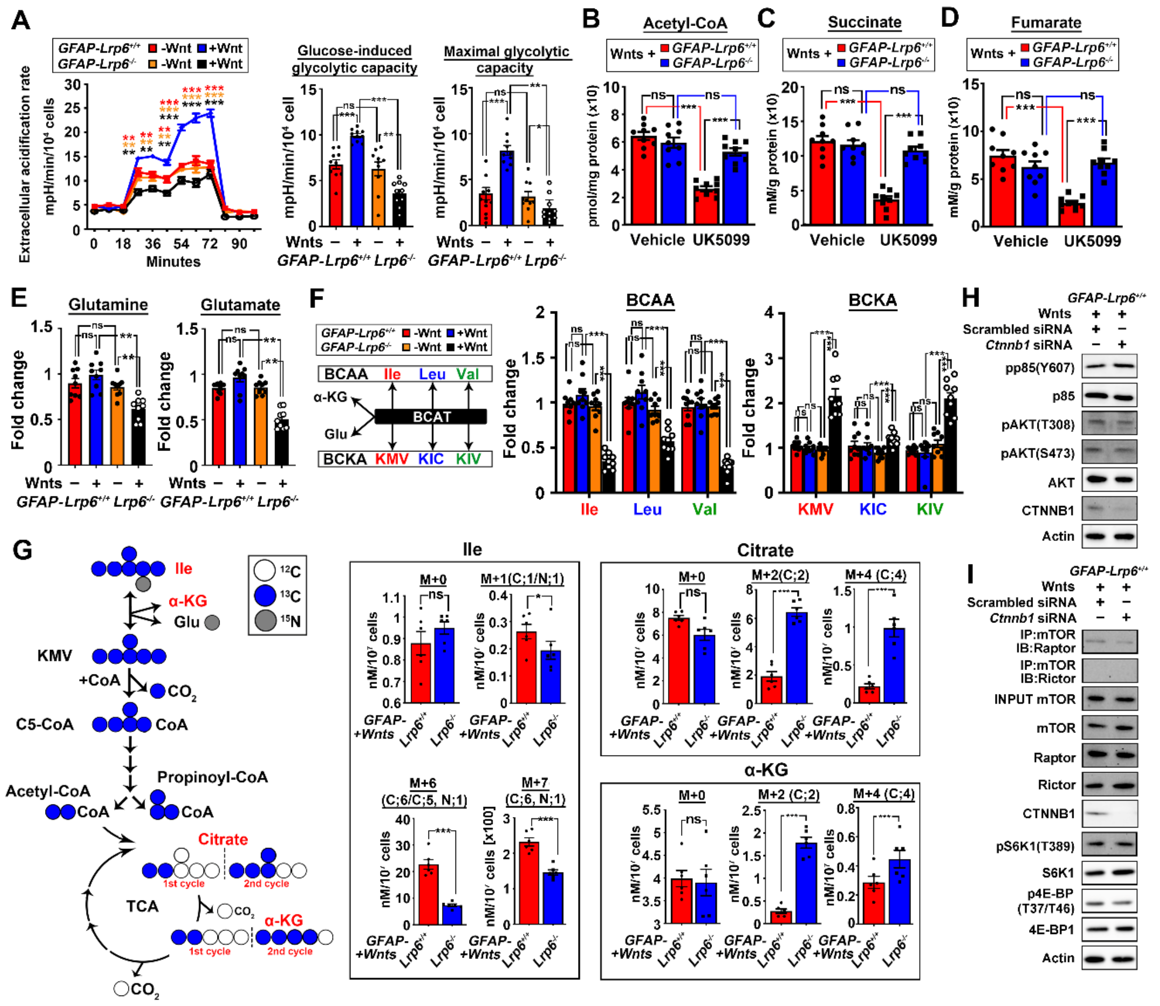

Supplementary Figure 3. Metabolic profiling of Wnt-stimulated *GFAP-Lrp6+/+* and *GFAP-Lrp6-/-* astrocytes

(A) Glycolytic functions of cultured astrocytes were analyzed (N=10, ***P<0.0001; *P<0.01; ns=non-significant; One-way ANOVA). (B-E) Intracellular levels of (B) acetyl-CoA, (C) succinate, (D) fumarate and (E) glutamine and glutamate in Wnt-exposed astrocyte cultures were measured by colorimetric assays [N=9, ***P<0.0001; **P<0.01; ns=non-significant; One-way ANOVA for (B-D); two-tailed unpaired t-test for (E)]. (F) Quantities of intracellular BCAAs and BCKAs in astrocyte cultures were determined by GC-MS. Relative fold differences are shown (N=6, ***P<0.0001; ns=non-significant; One-way ANOVA). (G) Schematic representation of isoleucine oxidative reactions linking towards the TCA cycle. Filled blue circles represent ^{13}C atoms; grey ones represent the ^{15}N atom derived from [$^{13}C_{6;}$ ^{15}N-Isoleucine] whereas white circles represent ^{12}C atoms derived from non-labelled metabolites in the system. On the right are mass isotopologue analyses of isoleucine (Ile); citrate and α-ketoglutarate (α-KG) in Wnts-treated astrocytes exposed to [$^{13}C_{6;}$ ^{15}N-Isoleucine] for 2 hours (N=6, ***P<0.0001, *P<0.01, ns=non-significant, two-tailed unpaired t-test). (H-I) Representative immunoblots of Wnts-treated *GFAP-Lrp6+/+* astrocyte cultures having *Ctnnb1* knockdown for 72 hours prior harvesting. Values represent the Mean ± SEM.

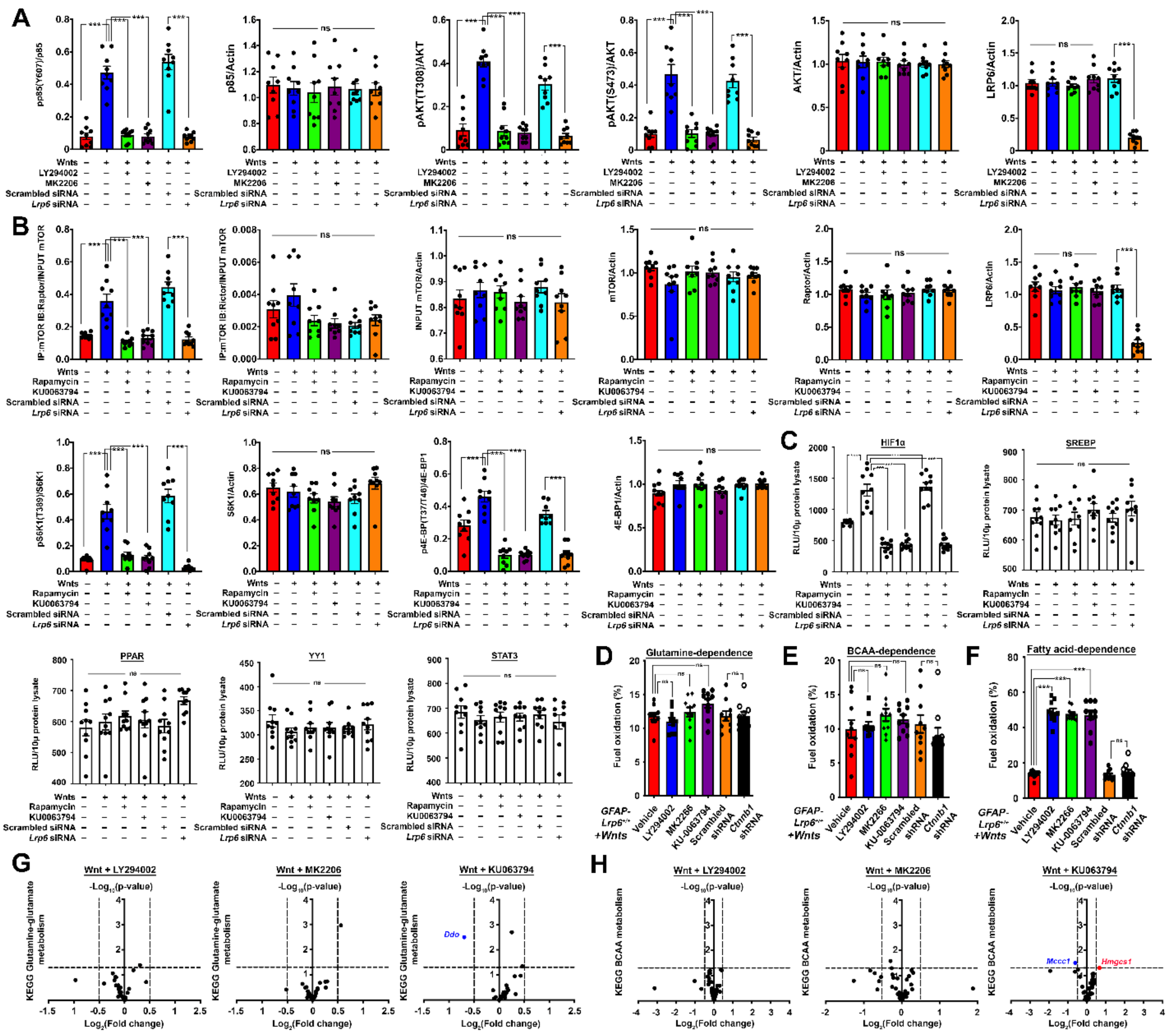

Supplementary Figure 4. Immunoblot quantifications and metabolic reprogramming of astrocytes when the Wnt-LRP6 mediated PI3K-AKT-mTOR axis is blocked

(A-B) Quantification of immunoblots in (A) Figure 3B and (B) 3C; each was normalized against the corresponding internal controls (N=8; ***P<0.0001; ns=non-significant; one-way ANOVA). (C) Luciferase reporter assays were performed in Wnt ligands or vehicle exposed *GFAP-Lrp6*$^{+/+}$ astrocytes pre-transfected with various transcription factor (i.e. HIF1α; SREBP; PPAR and YY1) nuclear activities reporters for 24 hours, followed by mTOR pharmacological inhibitor treatment (Lanes 1-4) for another 24 hours. Lanes 5-6 corresponded to cells co-transfected with scrambled or *Lrp6* siRNA with various transcription factor nuclear reporters indicated prior harvesting (N=10, ***P<0.0001; ns=non-significant; one-way ANOVA). (D-F) Fuel dependence was evaluated in Wnts-exposed *GFAP-Lrp6*$^{+/+}$ astrocytes pre-treated with 10µM LY294002; 1nM MK2206; 10nM KU-0063794 for 24 hours; or having *Ctnnb1* knockdown for 72 hours were analysed. The relative (D) glutamine; (E) BCAA and (F) fatty acid-dependence were evaluated (N=10, ***P<0.0001; ns=non-significant; One-way ANOVA). (G-H) Volcano plot showing the comparisons of (G) 32 KEGG glutamine-glutamate metabolic pathway genes and (H) 44 KEGG

BCAA degradation pathways gene expression levels obtained from qPCR analysis in Wnt-exposed *GFAP-Lrp6*$^{+/+}$ astrocytes pre-treated with 10μM LY294002; 1nM MK2206; 10nM KU-0063794 for 24 hours (N=6; horizontal dotted line p=0.05; two-tailed unpaired t-test). Red highlights indicate upregulated and blue ones indicated downregulated genes. Values represent the Mean ± SEM.

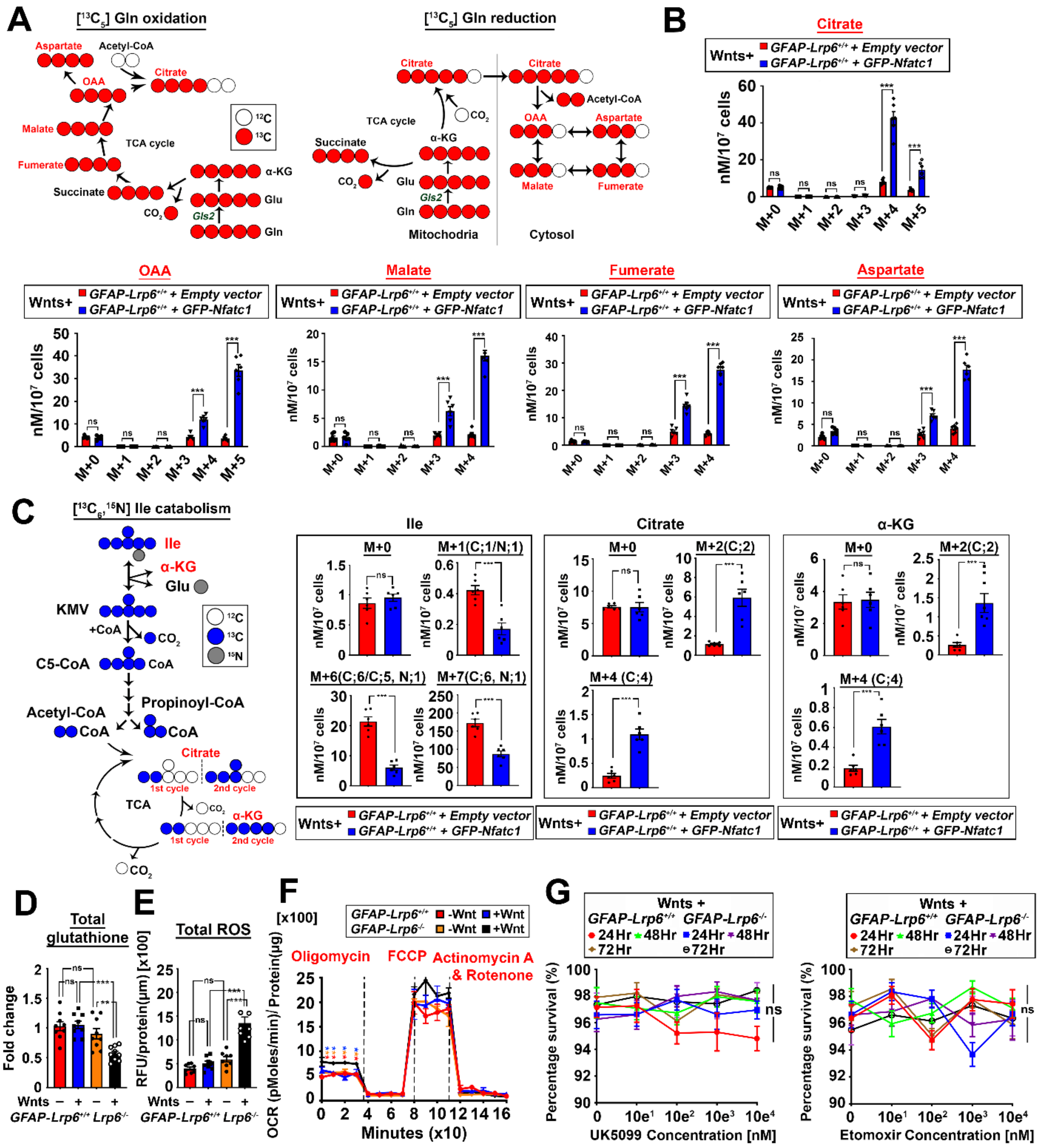

Supplementary Figure 5. Activation of NFAT1 drives metabolic adaptions to glutamine and BCAAs metabolisms in astrocytes

(A) Schematic representation of oxidative (Left) and reductive (right) reactions of glutamine-glutamate-α-ketoglutarate flux. Filled red circles represent ^{13}C atoms derived from [$^{13}C_5$]-glutamine; white circles represent ^{12}C atoms derived from non-labelled metabolites within the system. (B) Mass isotopologue analysis of citrate; oxaloacetate (OAA); malate; fumarate and aspartate in Wnts-treated *GFAP-Lrp6*$^{+/+}$ astrocytes with or without ectopic expression of GFP-tagged *Nfact1* for 48 hours, [U-^{13}C]-glutamine was then incubated for 2 hours prior harvesting (N=6, ***P<0.0001, ns=non-significant, two-tailed unpaired t-test). (C) Schematic

representation of isoleucine oxidative reactions linking to the TCA cycle. Filled blue circles represent ^{13}C atoms and grey ones represent ^{15}N atom derived from [$^{13}C_{6;}$ ^{15}N-Isoleucine]; white circles represent ^{12}C atoms derived from non-labelled metabolites in the system. On the right are mass isotopologue analyses of isoleucine (Ile); citrate and α-ketoglutarate (α-KG) in Wnt ligands exposed *GFAP-Lrp6*$^{+/+}$ astrocytes with or without ectopic expression of GFP-tagged *Nfatc1* for 48 hours. [$^{13}C_{6;}$ ^{15}N-Isoleucine] was incubated for 2 hours prior harvesting (N=6, ***P<0.0001, ns=non-significant, two-tailed unpaired t-test). (D) Total glutathione and (E) ROS levels in astrocyte cultures were determined (N=6, ***P<0.0001, **P<0.001, ns=non-significant, one-way ANOVA). (F) Oxygen consumption rates of astrocyte cultures were determined by Seahorse Mito Stress Test (N=8; ***P<0.01, coloured asterisk represent comparison between black datapoint with corresponding coloured datapoint, one-way ANOVA at corresponding time point). (G) Cell survival curves of Wnts-treated astrocyte cultures treated with various dosages of mitochondrial pyruvate carrier inhibitor UK5099 or fatty acid oxidation inhibitor Etomoxir for 24, 48 and 72 hours (N=6, ns=non-significant, Two-way ANOVA). Values represent the Mean ± SEM.

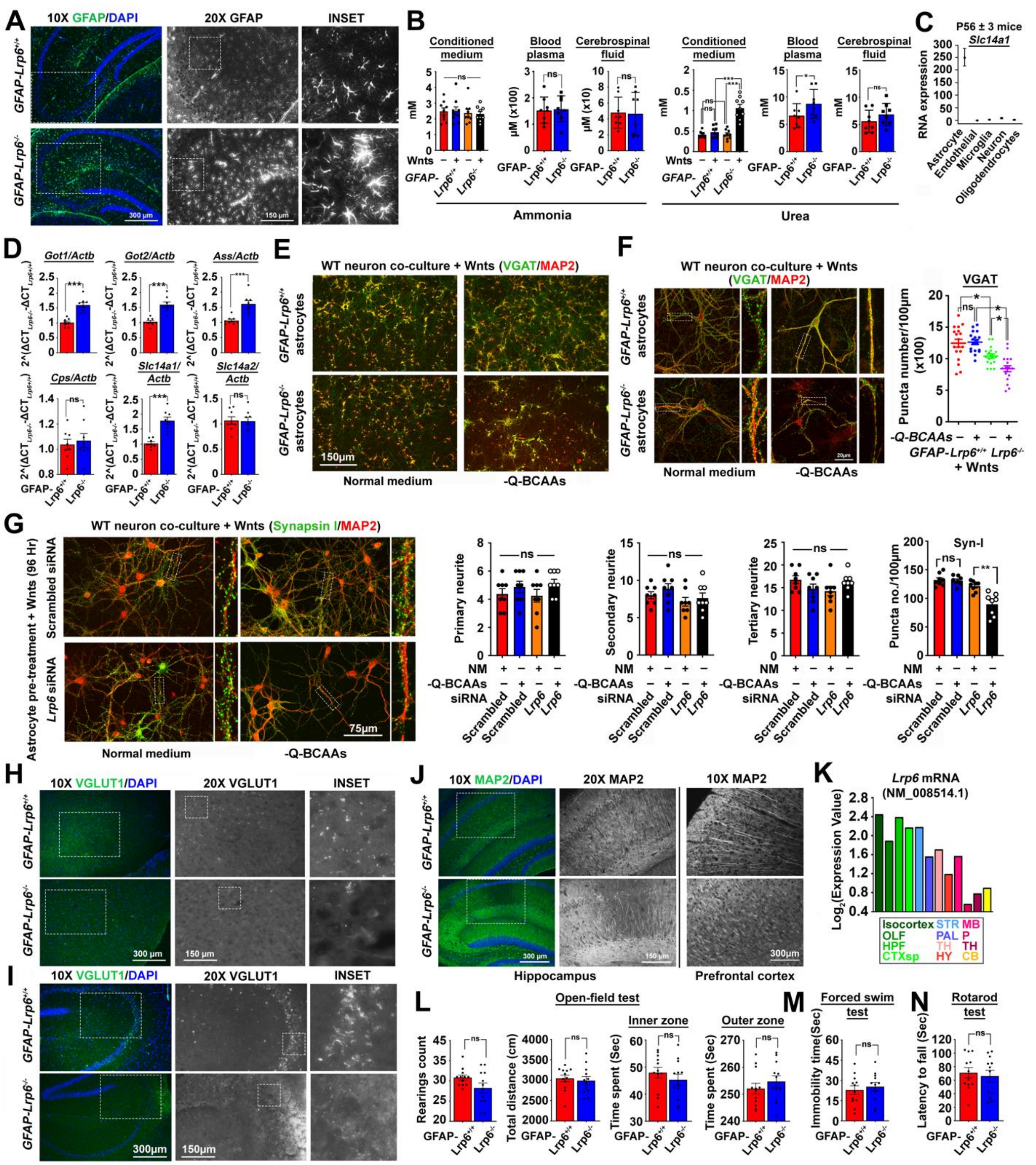

Supplementary Figure 6. Loss of LRP6 triggers reactive phenotype, compromising neurite integrity and synaptic abundances of neighbouring neurons.

(A) Representative immunohistochemistry images showing the effect of *Lrp6* knockout on astrocyte distribution and morphologies at hippocampal CA3 region. (B) Quantification of extracellular ammonia and urea in conditioned medium harvested *in vitro* after astrocyte culturing for 72 hours; blood plasma and cerebrospinal fluid *in vivo* (N=8, ***P<0.001, *P<0.01, ns=non-significant; one-way ANOVA and two-tailed unpaired t-test). (C) Referencing the public single cell transcriptome data of the adult (Post-natal day 56 ± 3) mouse brains, gene

expression level of *Slc14a1* in various mature brain cells were compared. (D) Quantitative PCR was performed to characterize key genes involved in the urea cycle (N=8, ***P<0.0001, ns=non-significant, two-tailed unpaired t-test). (E-F) Representative (E) low or (F) high magnification images of VGAT-positive puncta of wildtype neurons co-cultured with either Wnts-exposed *GFAP-Lrp6*$^{+/+}$ and *GFAP-Lrp6*$^{-/-}$ astrocytes in normal culturing medium or in one without supplementation of glutamine and BCAAs (-Q-BCAAs). Quantifications of VGAT puncta density are shown (N=18, ***P<0.0001, *P<0.01, ns=non-significant, One-way ANOVA). (G) Representative images of MAP2-positive dendrites and synapsin-I (SYN-I) puncta of wildtype neurons co-cultured with either Wnts-exposed astrocytes pre-expressed with scrambled or *Lrp6* siRNA for 72 hours in normal culturing medium or in one without supplementation of glutamine and BCAAs (-Q-BCAAs). Quantifications of primary, secondary and tertiary neurite number together with SYN-1 puncta density are shown (N=8, **P<0.001, ns=non-significant, One-way ANOVA). (H-I) Representative immunohistochemistry images showing the effect of *Lrp6* knockout in astrocytes on VGLUT1 density at the (H) prefrontal cortex and the (I) hippocampal CA3 regions. (J) Representative immunohistochemistry images showing the effect of *Lrp6* knockout in astrocytes on neurite integrity at the prefrontal cortex and the hippocampal CA2 regions (N=6). (K) Referencing the Allen Mouse Brain Atlas, expression level of *Lrp6* transcript was quantified in various mouse brain regions. (Forebrain regions: Isocortex; OLF and CTXsp. Between Fore-and-midbrain: STR and PAL. Midbrain regions: TH; HY and MB. Brainstem area: P and MY. Hindbrain: CB). (L) Number of rearing; total distance travelled; time-spent in the inner and outer zones were quantified in an open-field test paradigm (N=12, ns=non-significant, two-tailed unpaired t-test). (M) Quantification of immobility time recorded in the forced swim test paradigm (N=12, ns=non-significant, two-tailed unpaired t-test). (N) Quantification of time latency to fall in the rotarod test paradigm (N=12, ns=non-significant, two-tailed unpaired t-test). Values represent the Mean ± SEM.

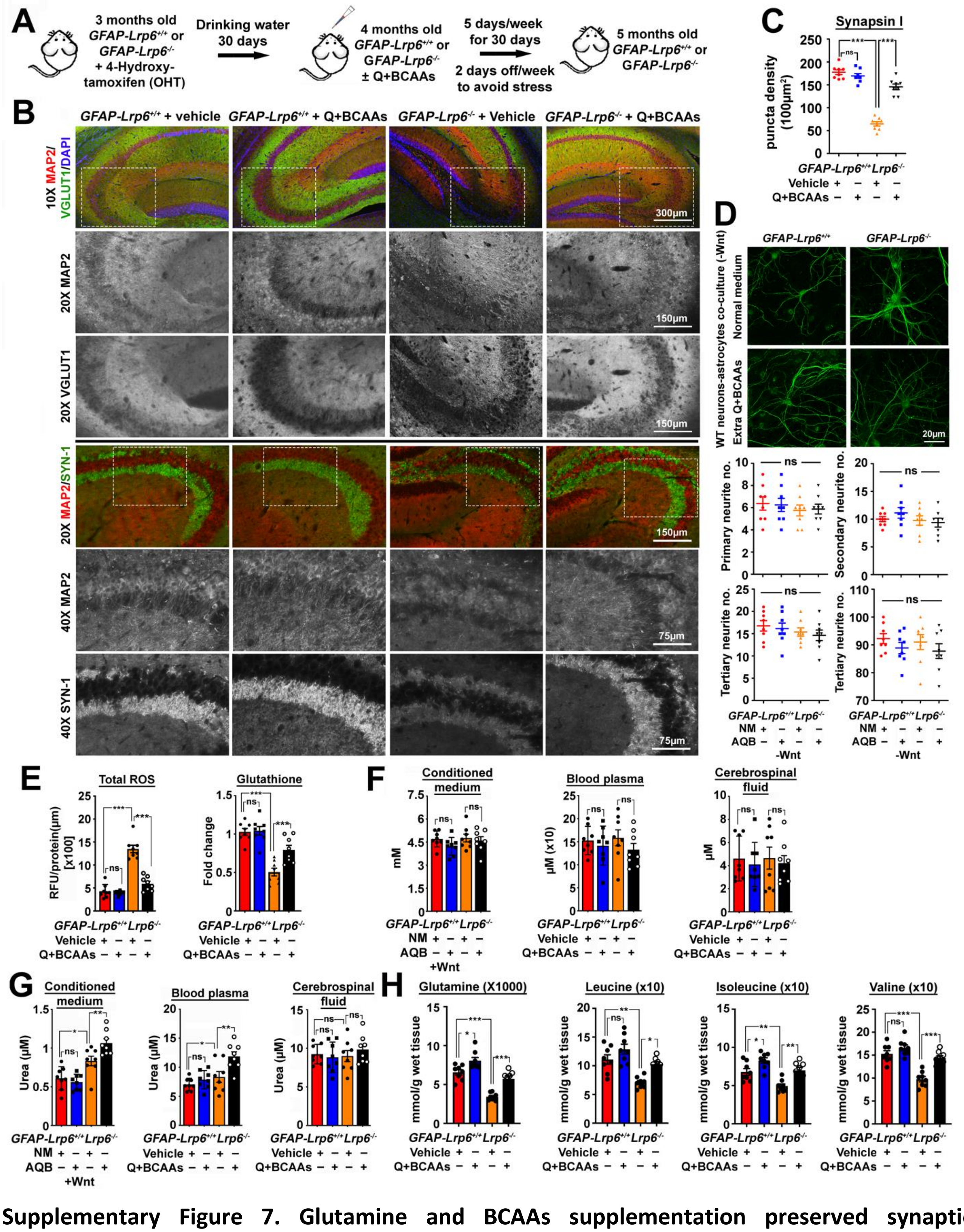

Supplementary Figure 7. Glutamine and BCAAs supplementation preserved synaptic abundance and neurite integrity of neurons

(A) Schematic diagram of the treatment scheme involving the intranasal delivery of a small bolus (~15-20μl) of 560 nmol/0.4 g average brain wet weight of glutamine (Q) and a mixture of

BCAAs (each 10 nmol/0.4 g average brain wet weight) or equal volume of vehicle into both the *GFAP-Lrp6*$^{+/+}$ and *GFAP-Lrp6*$^{-/-}$ mice for 30 days. (B) Representative immunohistochemistry images showing the effect of Q+BCAAs supplementation on VGLUT1 and Synapsin-I (SYN-1) signals as well as neurite integrity in the mouse hippocampal CA3 regions (N=8). (C) Quantification of Synapsin-I puncta density in (B) (N=8, ***P<0.0001, ns=non-significant, one-way ANOVA). (D) Representative images of MAP2-positive neurites in wildtype mouse primary neurons co-cultured with mouse astrocytes without Wnt ligands exposure in the routine culture medium or in one with extra supplementation of glutamine (Q) (340µM) and BCAAs (Ile: 40 mM; Leu: 20mM and Val: 20mM). Quantification of primary; secondary, tertiary neurites and viability of neurons are shown (N=8, ns=non-significant, One-way ANOVA). (E) Quantification of ROS and glutathione levels in cortical brain tissues harvested from mice subjected to Q+BCAAs supplementation or vehicle controls (N=8; ***P<0.0001, ns=non=significant, one-way ANOVA). (F-G) Quantification of extracellular ammonia and urea in conditioned medium harvested *in vitro*; blood plasma and cerebrospinal fluid *in vivo* (N=8, ***P<0.0001, **P<0.001, *P<0.01, ns=non-significant; one-way ANOVA). (H) Quantification of glutamine and BCAA levels in brain tissues of *GFAP-Lrp6*$^{+/+}$ and *GFAP-Lrp6*$^{-/-}$ mice subjected to either vehicle or Q+BCAAs supplementation (N=8, ***P<0.0001, **P<0.001, *P<0.01, ns=non=significant, one-way ANOVA). Values represent the Mean ± SEM.

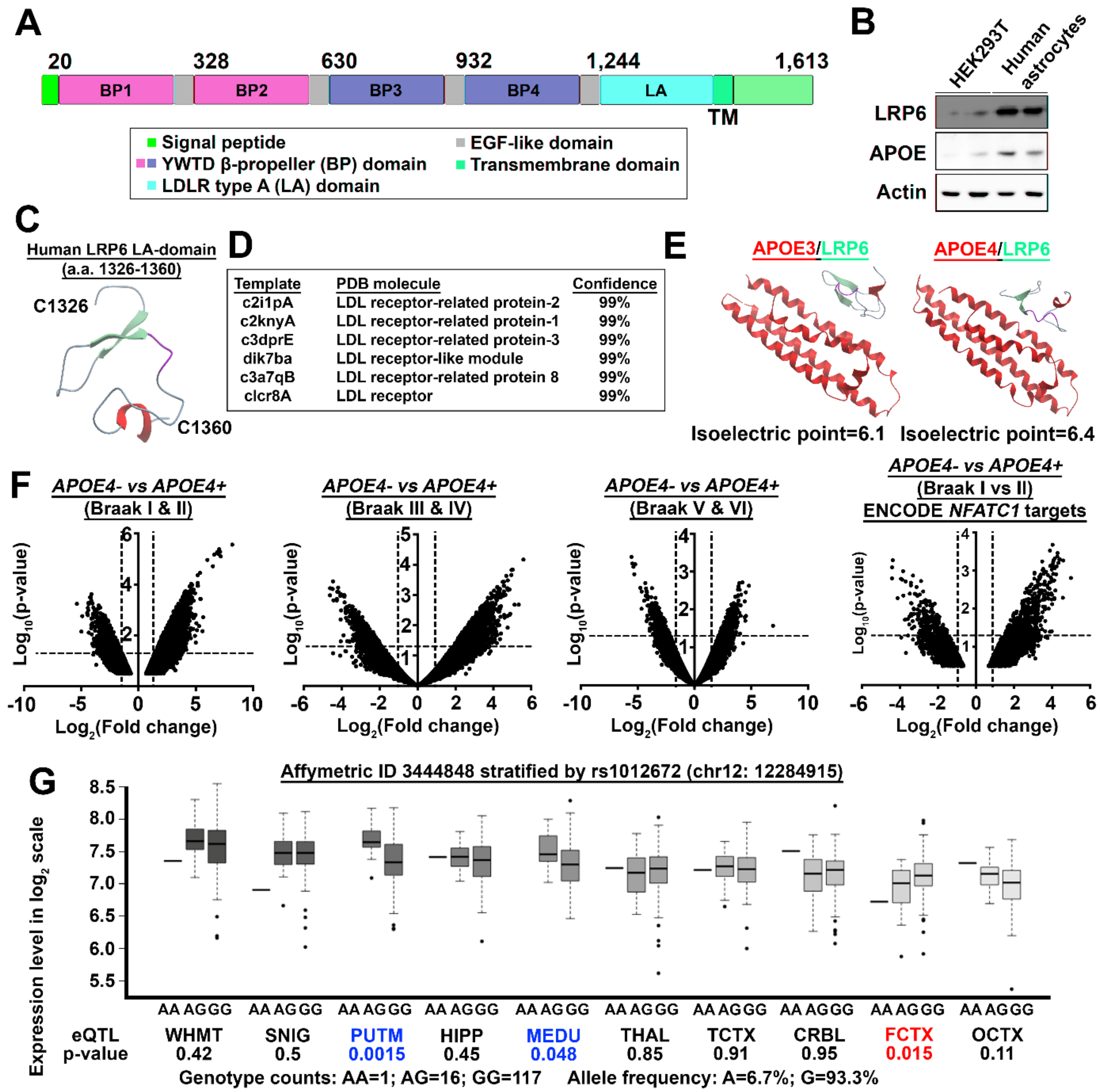

Supplementary Figure 8. APOE4 mediates LRP6 loss effect in astrocytes and contributes to enhanced risk of LOAD.

(A) A schematic diagram showing the domain organization of human LRP6 protein. 'BP' stands for YWTD β-propeller domain. The first and second YWTD domains are shown in pink and the third and fourth ones in blue. EGF-like domains, LDLR type A repeats and transmembrane region are shown in gray, cyan and green, respectively. (B) Representative immunoblots revealing endogenous expressions of LRP6 and APOE were almost absent in human HEK293T cells as compared to human astrocytes (N=6). (C) The predicted LRP6 LA domain structure by Phyre[2] adopts a (D) LDL-receptor like domain with 99% confidences. (E) *In silico* docking was performed between LRP6 with (E) APOE3 or (F) APOE4 proteins. Predicted forms of interaction as well as the protein isoelectric points are shown[33]. (F) Left 3 plots: Volcano plots showing comparisons of gene expression profiles between APOE4+ and APOE4- astrocytes laser-dissected out from post-mortem human brains of AD patients of various Braak Stages (N=3

samples per group). Far right: Volcano plot showing the comparison of all ENCODE *NFATC1* target gene expressions in APOE4+ and APOE4- astrocytes laser-dissected out from post-mortem human brains of AD patients within Braak Stages I and II (N=3 samples per group). Horizontal otted line indicated P=0.05. (G) Association of SNP rs1012672 with *LRP6* gene expression level in 10 brain regions (Affymetrix ID 3444848). Data were extracted from the *BRAINEAC* database (http://www.braineac.org). Abbreviations: eQTL, expression quantitative trait locus; SING, substantia nigra; THAL, thalamus; MEDU, inferior olivary nucleus; PUTM, putamen; HIPP, hippocampus; TCTX, temporal cortex; WHMT, intralobular white matter; FCTX, frontal cortex; OCTX, occipital cortex; CRBL, cerebellar cortex. Red indicates significant downregulation and blue as significant upregulation. On the forward strand: AA= homozygous G->A mutation; AG=heterozygous G->A mutation; GG=homozygous wildtype. Genotype counts and allele frequencies are labelled.

Supplementary Table 1. Full list of metabolites identified from *GFAP-LRP6*$^{+/+}$ and *GRAP-LRP6*$^{-/-}$ astrocytes using the CE-TOFMS.

Compound name	HMDB ID	GFAP-Lrp6+/+		GFAP-Lrp6-/-		GFAP-Lrp6-/-/GFAP-LRP6+/+	
		Mean	S.D.	Mean	S.D.	FC	*p-value*
3-Hydroxy-3-methylglutaryl-CoA	HMDB0001375	1.5E-02	3.7E-03	4.4E-02	8.8E-03	2.8	0.000
3-Hydroxyisovaleryl-CoA	HMDB0006870	8.7E-05	1.4E-05	1.2E-04	1.9E-05	1.4	0.007
(S)-3-Hydroxyisobutyric acid	HMDB0000023	2.1E-02	1.1E-03	4.2E-02	5.4E-03	2.0	0.000
(S)-Methylmalonate semialdehyde	HMDB0002217	7.0E-03	6.1E-04	9.1E-03	6.9E-04	1.3	0.000
1-Methyl-4-imidazoleacetic acid	HMDB0002820	5.4E-04	1.3E-04	5.1E-04	8.1E-05	0.9	0.585
1-Methyladenosine	HMDB0003331	2.2E-04	3.6E-05	2.0E-04	2.4E-05	0.9	0.352
1-Methylhistamine	HMDB0000898	2.7E-04	5.9E-05	3.0E-04	8.4E-06	1.1	0.231
1-Methylhistidine<br>3-Methylhistidine	HMDB0000001<br>HMDB0000479	6.4E-04	1.3E-04	5.0E-04	1.2E-04	0.8	0.081
1-Methylnicotinamide	HMDB0000699	1.6E-04	N.D.	N.D.	N.D.	N.D.	N.D.
2,3-Diphosphoglyceric acid	HMDB0001294	3.6E-05	9.9E-06	1.2E-04	8.0E-05	3.3	0.047
2-Aminoisobutyric acid<br>2-Aminobutyric acid	HMDB0001906<br>HMDB0000452	8.3E-04	N.D.	N.D.	N.D.	N.D.	N.D.
2'-Deoxycytidine	HMDB0000014	6.4E-05	N.D.	N.D.	N.D.	N.D.	N.D.
2-Hydroxyglutaric acid	HMDB0000606,HMDB0000694	1.7E-03	2.2E-04	1.9E-03	1.5E-04	1.1	0.186
2-Hydroxyvaleric acid	HMDB0001863	N.D.	N.D.	N.D.	N.D.	N.D.	N.D.
2-Methyl-3-hydroxybutyryl-CoA	HMDB0001356	1.5E-02	3.3E-03	3.4E-02	8.6E-03	2.3	0.001
2-Methylbutyryl-CoA	HMDB0001041	1.5E-02	3.9E-03	3.5E-02	5.1E-03	2.3	0.000
3'-Dephospho CoA	HMDB0001373	2.1E-04	2.9E-05	2.3E-04	3.8E-05	1.1	0.244
3-Hydroxy-3-methylglutaric acid	HMDB0000355	5.1E-04	1.1E-04	6.5E-04	5.5E-05	1.3	0.017
3-Hydroxybutyric acid	HMDB0000011,HMDB0000357,HMDB0000442	6.7E-04	1.9E-04	8.5E-04	1.6E-04	1.3	0.104

3-Methyl-2-oxovaleric acid	HMDB0000491	1.6E-02	4.4E-03	4.3E-02	1.4E-02	2.7	0.001
3-Methylbutanoyl-CoA	HMDB0001113	7.7E-05	5.0E-05	1.3E-04	1.8E-05	1.7	0.031
3-Methylbutanoyl-CoA	HMDB0001113	3.1E-03	1.5E-03	4.5E-03	1.1E-03	1.5	0.096
3-Methylcrotonyl-CoA	HMDB0001493	6.2E-04	2.4E-04	9.5E-04	2.2E-04	1.5	0.033
3-Methylglutaconyl-CoA	HMDB0000522	1.6E-04	2.4E-05	3.0E-04	2.1E-05	1.9	0.000
3-Phosphoglyceric acid	HMDB0000807	1.0E-04	3.9E-06	1.1E-04	1.5E-05	1.1	0.350
4-Guanidinobutyric acid	HMDB0003464	4.8E-04	6.2E-05	7.8E-04	2.2E-05	1.6	0.000
5-Aminoimidazole-4-carboxamide ribotide	HMDB0001517	4.2E-05	3.3E-06	4.1E-05	8.6E-06	1.0	0.965
5'-Deoxy-5'-methylthioadenosine	HMDB0001173	N.D.	N.D.	N.D.	N.D.	N.D.	N.D.
5-Hydroxyindoleacetic acid	HMDB0000763	6.0E-05	2.2E-05	6.1E-05	1.1E-05	1.0	0.928
5-Oxoproline	HMDB0000267	1.3E-03	6.4E-04	1.3E-03	5.0E-04	1.0	0.910
6-Phosphogluconic acid	HMDB0001316	3.7E-04	9.6E-05	2.6E-04	4.0E-05	0.7	0.020
Acetoacetate	HMDB0000060	8.5E-05	1.4E-05	1.2E-04	1.4E-05	1.5	0.002
Acetoacetyl-CoA	HMDB0001484	1.5E-04	2.6E-05	4.3E-04	2.9E-04	2.9	0.037
Acetyl CoA_divalent	HMDB0001206	2.3E-04	2.2E-05	2.4E-04	4.4E-05	1.1	0.560
Acetylcholine	HMDB0000895	2.3E-03	2.9E-04	1.1E-03	2.2E-04	0.5	0.000
Adenine	HMDB0000034	7.2E-04	9.5E-05	7.9E-04	8.8E-05	1.1	0.198
Adenosine	HMDB0000050	4.1E-02	7.5E-03	4.2E-02	3.5E-03	1.0	0.817
Adenylosuccinic acid	HMDB0000536	1.6E-03	1.5E-04	1.6E-03	4.7E-04	1.0	0.839
ADP	HMDB0001341	1.3E-02	1.3E-03	1.1E-02	1.2E-03	0.8	0.018
ADP-glucose GDP-fucose	HMDB0006557 HMDB0001095	4.2E-04	2.3E-05	4.2E-04	4.1E-05	1.0	0.843
ADP-ribose	HMDB0001178	2.0E-03	2.7E-04	3.1E-03	5.8E-04	1.6	0.001
Ala	HMDB0000161,HMDB0001310	6.9E-02	4.2E-03	3.6E-02	8.0E-03	0.5	0.000
allo-Threonine	HMDB0004041	N.D.	N.D.	N.D.	N.D.	N.D.	N.D.

alpha-Ketoisovaleric acid	HMDB0000019	1.2E-02	2.6E-03	1.9E-02	2.9E-03	1.6	0.001
AMP	HMDB0000045	3.7E-02	4.4E-03	3.1E-02	3.1E-03	0.8	0.022
Anserine_divalent		2.4E-04	4.4E-05	3.3E-04	8.3E-05	1.4	0.036
Arg	HMDB0000517,HMDB0003416	1.3E-02	1.4E-03	1.4E-02	1.0E-03	1.0	0.592
Argininosuccinic acid	HMDB0000052	3.7E-04	3.6E-05	4.0E-04	1.8E-04	1.1	0.653
Ascorbate 2-glucoside		N.D.	N.D.	N.D.	N.D.	N.D.	N.D.
Ascorbic acid	HMDB0000044	3.9E-02	5.9E-03	3.2E-02	9.0E-03	0.8	0.158
Asn	HMDB0000168,HMDB0033780	5.4E-03	3.8E-04	4.8E-03	1.2E-03	0.9	0.340
Asp	HMDB0000191,HMDB0006483	1.0E-02	7.2E-04	1.9E-02	7.9E-03	1.9	0.018
ATP	HMDB0000538	7.9E-03	1.5E-03	6.9E-03	1.6E-03	0.9	0.276
Betaine	HMDB0000043	2.9E-03	5.0E-04	4.3E-03	2.7E-03	1.5	0.227
Butyrylcarnitine	HMDB0002013	N.D.	N.D.	N.D.	N.D.	N.D.	N.D.
cAMP	HMDB0000058	1.2E-04	2.4E-05	2.2E-04	3.3E-04	1.8	0.499
Carboxymethyllysine		4.9E-04	2.8E-04	2.3E-04	3.5E-05	0.5	0.046
Carnitine	HMDB0000062	1.7E-02	1.5E-03	2.4E-02	1.3E-03	1.4	0.000
Carnosine	HMDB0000033	2.9E-03	5.9E-04	3.5E-03	1.0E-03	1.2	0.307
cCMP<br>2',3'-cCMP	 HMDB0011691	N.D.	N.D.	1.8E-05	6.1E-06	N.D.	N.D.
CDP	HMDB0001546	1.3E-04	1.9E-05	1.1E-04	1.8E-05	0.8	0.112
CDP-choline	HMDB0001413	7.0E-04	5.6E-05	6.0E-04	2.6E-05	0.9	0.003
Choline	HMDB0000097	3.8E-02	5.5E-03	4.4E-02	5.0E-03	1.2	0.085
cis-Aconitic acid	HMDB0000072	4.2E-04	4.6E-05	3.8E-04	1.5E-05	0.9	0.115
Citraconic acid	HMDB0000634	7.0E-05	1.5E-05	7.5E-05	1.8E-05	1.1	0.771
Citric acid	HMDB0000094	9.0E-03	4.0E-03	7.8E-03	2.0E-03	0.9	0.526
Citrulline	HMDB0000904	1.4E-03	7.2E-05	2.6E-03	3.0E-04	1.8	0.000
CMP	HMDB0000095	7.1E-04	1.1E-04	5.0E-04	5.0E-05	0.7	0.002
CMP-*N*-	HMDB0001176	7.7E-04	4.0E	7.6E-04	5.6E	1.0	0.82

acetylneuraminate			-05		-05		2
CoA_divalent		1.1E-03	8.4E-05	1.3E-03	9.8E-05	1.3	0.000
Creatine	HMDB0000064	3.4E-02	1.3E-03	3.9E-02	3.0E-03	1.1	0.011
Creatinine	HMDB0000562	1.3E-03	5.9E-05	1.3E-03	8.2E-05	1.0	0.267
CTP	HMDB0000082	5.4E-05	1.3E-05	5.0E-05	1.5E-05	0.9	0.594
Cys	HMDB0000574,HMDB0003417	1.8E-04	3.4E-05	6.0E-04	1.9E-04	3.2	0.000
Cystathionine	HMDB0000099	1.5E-03	5.5E-04	3.9E-03	1.3E-03	2.5	0.002
Cysteine glutathione disulfide	HMDB0000656	4.7E-04	9.9E-05	5.5E-04	1.2E-04	1.2	0.265
Cytidine	HMDB0000089	2.0E-03	2.1E-04	1.8E-03	3.1E-04	0.9	0.360
Diethylaminomalonic acid		1.2E-03	5.1E-04	7.7E-04	2.9E-04	0.6	0.097
Dihydroxyacetone phosphate	HMDB0001473	2.3E-03	7.4E-04	8.7E-04	1.7E-04	0.4	0.001
Disulfiram		N.D.	N.D.	N.D.	N.D.	N.D.	N.D.
dTDP	HMDB0001274	N.D.	N.D.	N.D.	N.D.	N.D.	N.D.
dTDP-glucose	HMDB0001328	N.D.	N.D.	N.D.	N.D.	N.D.	N.D.
dTMP	HMDB0001227	N.D.	N.D.	N.D.	N.D.	N.D.	N.D.
Ethanolamine	HMDB0000149	6.8E-03	1.0E-03	4.7E-03	5.8E-04	0.7	0.001
Ethanolamine phosphate	HMDB0000224	1.7E-02	1.8E-03	9.0E-03	1.0E-03	0.5	0.000
FAD_divalent		1.0E-04	9.1E-06	1.1E-04	7.7E-06	1.1	0.111
Fructose 1,6-diphosphate	HMDB0001058	1.5E-03	8.4E-04	3.4E-04	4.9E-05	0.2	0.007
Fructose 6-phosphate	HMDB0000124	4.7E-04	1.8E-04	1.4E-04	1.6E-05	0.3	0.001
Fumaric acid	HMDB0000134	2.2E-03	2.0E-04	2.1E-03	1.5E-04	1.0	0.401
GABA	HMDB0000112	9.0E-03	1.0E-03	6.3E-03	2.3E-03	0.7	0.024
GDP	HMDB0001201	5.0E-03	4.4E-04	3.8E-03	9.4E-05	0.8	0.000
GDP-glucose GDP-mannose GDP-galactose	HMDB0003351 HMDB0001163	8.2E-04	8.5E-05	7.3E-04	6.8E-05	0.9	0.061
Gln	HMDB0000641,HMDB0003423	2.4E-02	4.3E-03	1.3E-02	7.0E-03	0.6	0.009

Glu	HMDB0000148,HMDB0003339	3.1E-02	1.3E-03	1.9E-02	4.8E-03	0.6	0.000
Gluconic acid	HMDB0000625	1.7E-04	1.5E-05	1.6E-04	1.7E-05	0.9	0.242
Glucose 1-phosphate	HMDB0001586	5.8E-04	1.4E-04	2.3E-04	3.1E-05	0.4	0.000
Glucose 6-phosphate	HMDB0001401	1.6E-03	6.2E-04	4.1E-04	4.7E-05	0.3	0.001
Glu-Glu		6.2E-05	1.7E-05	5.5E-05	5.0E-06	0.9	0.612
Glutathione (GSH)	HMDB0000125	9.0E-02	6.7E-03	6.3E-02	2.6E-02	0.7	0.031
Glutathione (GSSG)_divalent		1.8E-02	1.8E-03	9.9E-03	5.7E-03	0.6	0.010
Gly	HMDB0000123	1.4E-03	5.4E-04	2.4E-03	4.7E-04	1.7	0.006
Glyceraldehyde 3-phosphate	HMDB0001112	5.2E-05	1.4E-05	N.D.	N.D.	N.D.	N.D.
Glycerol	HMDB0000131	1.0E-02	2.7E-03	1.3E-02	3.2E-03	1.2	0.200
Glycerol 3-phosphate	HMDB0000126	8.2E-03	1.4E-03	1.6E-02	3.1E-03	1.9	0.000
Glycerophosphocholine	HMDB0000086	6.4E-02	1.1E-02	7.5E-02	5.2E-03	1.2	0.053
Gly-Gly	HMDB0011733	2.8E-04	3.9E-05	2.5E-04	1.2E-05	0.9	0.368
GMP	HMDB0001397	7.6E-03	6.0E-04	5.9E-03	4.2E-04	0.8	0.000
GTP	HMDB0001273	2.2E-03	2.5E-04	1.8E-03	3.1E-04	0.8	0.020
Guanosine	HMDB0000133	4.0E-03	5.2E-04	4.6E-03	5.1E-04	1.1	0.104
Hexanoic acid	HMDB0000535	4.8E-05	3.1E-06	5.1E-05	3.9E-06	1.0	0.328
His	HMDB0000177	1.0E-02	2.5E-03	7.3E-03	1.9E-03	0.7	0.051
Histamine	HMDB0000870	4.9E-05	1.0E-06	3.9E-05	N.D.	0.8	N.D.
Homocarnosine	HMDB0000745	6.2E-03	7.8E-04	1.1E-02	7.7E-04	1.8	0.000
Homoserine	HMDB0000719	6.1E-04	4.5E-05	5.5E-04	5.2E-05	0.9	0.054
Homovanillic acid	HMDB0000118	2.0E-04	7.0E-05	2.2E-04	N.D.	1.1	N.D.
Hydroxyproline	HMDB0000725	7.6E-04	5.3E-05	7.6E-04	3.5E-05	1.0	0.990
Hypotaurine	HMDB0000965	9.6E-04	3.1E-04	5.8E-04	2.6E-04	0.6	0.054
Hypoxanthine	HMDB0000157	5.8E-03	1.1E-03	6.3E-03	1.1E-03	1.1	0.462
Ile	HMDB0000172	1.7E-02	5.4E	5.8E-03	2.4E	0.3	0.00

			-03		-03		1
IMP	HMDB0000175	6.7E-03	9.2E-04	6.5E-03	1.9E-03	1.0	0.774
Inosine	HMDB0000195	2.4E-02	4.3E-03	2.3E-02	3.3E-03	1.0	0.786
Isethionic acid	HMDB0003903	9.2E-04	1.4E-04	1.0E-03	7.0E-05	1.1	0.153
Isobutyryl-CoA	HMDB0001243	2.0E-05	3.9E-06	4.2E-05	1.2E-05	2.1	0.002
Isovaleric acid<br>Valeric acid	HMDB0000718<br>HMDB0000892	6.1E-05	N.D.	N.D.	N.D.	N.D.	N.D.
Ketoleucine	HMDB0000695	2.7E-02	9.5E-03	4.1E-02	2.3E-02	1.5	0.192
L-3-Aminoisobutanoate	HMDB0003911	2.0E-03	5.5E-04	3.2E-03	8.7E-04	1.6	0.017
Lactic acid	HMDB0000190,HMDB0001311	1.8E-01	9.8E-03	1.9E-01	4.0E-02	1.0	0.870
Leu	HMDB0000687	1.6E-02	3.6E-03	9.2E-03	6.0E-03	0.6	0.030
Lys	HMDB0000182,HMDB0003405	3.5E-02	9.5E-03	1.8E-02	1.5E-03	0.5	0.002
Malic acid	HMDB0000156,HMDB0000744	1.9E-02	1.4E-03	1.6E-02	9.4E-04	0.9	0.003
Met	HMDB0000696	5.0E-03	8.7E-04	3.9E-03	1.3E-03	0.8	0.126
Methylmalonyl-CoA	HMDB0001269	6.3E-02	2.6E-02	7.0E-02	7.4E-02	1.1	0.834
Mucic acid	HMDB0000639	4.2E-05	7.9E-06	4.7E-05	3.7E-06	1.1	0.362
myo-Inositol 1-phosphate<br>*myo*-Inositol 3-phosphate	HMDB0000213<br>HMDB0006814	1.8E-03	2.1E-04	1.4E-03	1.5E-04	0.8	0.005
myo-Inositol 2-phosphate	HMDB0002985	8.0E-04	9.0E-05	5.0E-04	4.5E-05	0.6	0.000
N^2-Acetylaminoadipic acid	HMDB0062715	8.0E-05	2.2E-05	1.8E-04	4.5E-05	2.2	0.001
N^2-Succinylornithine	HMDB0001199	2.4E-04	4.7E-05	6.2E-04	1.3E-04	2.6	0.000
N^5-Ethylglutamine		6.7E-04	2.0E-04	5.0E-04	1.3E-04	0.7	0.109
N^6,N^6,N^6-Trimethyllysine	HMDB0001325	7.2E-04	7.8E-05	3.4E-04	2.7E-05	0.5	0.000
N^6-Methyllysine	HMDB0002038	4.5E-04	5.5E-05	3.9E-04	6.4E-05	0.9	0.134
N^8-Acetylspermidine	HMDB0002189	4.9E-05	N.D.	5.8E-05	5.2E-06	1.2	N.D.
N-Acetylaspartic acid	HMDB0000812	1.5E-01	8.9E-03	1.3E-01	1.4E-02	0.8	0.003

N-Acetylgalactosamine<br>*N*-Acetylmannosamine<br>*N*-Acetylglucosamine	HMDB0000853<br>HMDB0001129<br>HMDB0000215	4.2E-04	N.D.	2.5E-04	N.D.	0.6	N.D.
N-Acetylglucosamine 1-phosphate	HMDB0001367	2.7E-04	3.9E-05	1.8E-04	2.0E-05	0.6	0.000
N-Acetylglucosamine 6-phosphate	HMDB0001062	1.3E-04	2.3E-05	9.3E-05	1.7E-05	0.7	0.007
N-Acetylglutamic acid	HMDB0001138	1.2E-03	8.6E-05	1.1E-03	5.9E-05	0.9	0.013
N-Acetylglutamine	HMDB0006029	2.5E-04	1.0E-04	2.1E-04	7.9E-05	0.8	0.474
N-Acetyllysine	HMDB0000446	2.1E-04	2.3E-05	2.0E-04	1.5E-05	1.0	0.760
N-Acetylmethionine	HMDB0011745	4.8E-05	5.9E-06	3.9E-05	5.7E-06	0.8	0.019
N-Acetylneuraminic acid	HMDB0000230	2.6E-03	1.6E-04	1.9E-03	1.3E-04	0.7	0.000
N-Acetylphenylalanine	HMDB0000512	2.5E-05	2.2E-06	1.7E-05	4.1E-06	0.7	0.126
N-Acetyl-β-alanine	HMDB0061880	7.3E-05	4.9E-06	7.0E-05	7.8E-06	1.0	0.375
NAD^+	HMDB0000902	7.4E-03	1.3E-03	5.0E-03	1.3E-03	0.7	0.008
NADH	HMDB0001487	2.1E-04	6.6E-05	1.4E-04	4.1E-05	0.6	0.043
$NADP^+$	HMDB0000217	4.6E-04	7.0E-05	4.1E-04	6.1E-05	0.9	0.213
NADPH_divalent		3.4E-05	1.1E-05	N.D.	N.D.	N.D.	N.D.
N-Glycolylneuraminic acid	HMDB0000833	3.9E-05	1.0E-05	4.5E-05	5.8E-06	1.1	0.295
Nicotinamide	HMDB0001406	2.3E-02	1.1E-02	1.2E-02	3.7E-03	0.5	0.037
Nicotinic acid	HMDB0001488	9.4E-05	N.D.	N.D.	N.D.	N.D.	N.D.
NMN	HMDB0000229	1.2E-04	2.3E-05	1.3E-04	2.1E-05	1.0	0.874
Noradrenaline<br>6-Hydroxydopamine	HMDB0000216<br>HMDB0001537	1.6E-04	3.9E-05	1.6E-04	3.6E-05	1.0	0.859
O-Acetylcarnitine	HMDB0000201	1.5E-02	1.4E-03	2.4E-02	1.8E-03	1.6	0.000

O-Acetylhomoserine 2-Aminoadipic acid	HMDB0000510	3.1E-03	1.3E-03	3.9E-03	7.5E-04	1.3	0.219
Octopamine Dopamine	HMDB0004825 HMDB0000073	2.3E-04	6.9E-05	N.D.	N.D.	N.D.	N.D.
Ornithine	HMDB0000214,HMDB0003374	4.2E-04	7.8E-05	3.3E-04	4.7E-05	0.8	0.038
O-Succinylhomoserine		3.6E-05	9.3E-06	2.8E-05	6.4E-06	0.8	0.146
Oxoglutaric acid	HMDB0000208	5.3E-02	1.4E-02	6.2E-02	7.9E-03	1.2	0.029
Pantothenic acid	HMDB0000210	1.1E-03	1.6E-04	1.4E-03	1.9E-04	1.3	0.019
Pelargonic acid	HMDB0000847	1.0E-04	2.5E-05	9.7E-05	6.1E-06	1.0	0.703
Phe	HMDB0000159	1.3E-02	4.0E-03	1.0E-02	3.3E-03	0.8	0.277
Phosphocreatine	HMDB0001511	1.1E-04	1.6E-05	1.6E-04	2.9E-05	1.5	0.002
Phosphoenolpyruvic acid	HMDB0000263	N.D.	N.D.	4.7E-05	5.9E-06	N.D.	N.D.
Phosphorylcholine	HMDB0001565	4.6E-02	3.9E-03	3.5E-02	2.9E-03	0.8	0.000
Phytic acid_divalent		2.6E-04	2.3E-05	2.4E-04	2.1E-05	0.9	0.093
Pipecolic acid	HMDB0000070,HMDB0000716,HMDB0005960	4.0E-04	8.7E-05	4.9E-04	9.6E-05	1.2	0.117
Pro	HMDB0000162,HMDB0003411	1.3E-02	9.7E-04	9.8E-03	8.7E-04	0.7	0.000
Propionyl-CoA	HMDB0001275	7.4E-05	8.1E-06	1.0E-04	7.0E-06	1.4	0.000
Prostaglandin E_2	HMDB0001220	6.8E-05	1.7E-05	2.0E-05	4.1E-06	0.3	0.001
Prostaglandin $F_{2\alpha}$	HMDB0001139	3.1E-05	8.3E-06	N.D.	N.D.	N.D.	N.D.
PRPP	HMDB0000280	3.2E-04	7.8E-05	2.6E-04	5.2E-05	0.8	0.138
Putrescine	HMDB0001414	1.4E-04	2.1E-05	1.4E-04	2.6E-05	1.0	0.885
Pyridoxamine 5'-phosphate	HMDB0001555	2.6E-04	2.2E-05	3.0E-04	2.6E-05	1.2	0.008
Pyrophosphate	HMDB0000250	1.6E-03	1.3E-04	1.5E-03	1.2E-04	1.0	0.468
Quinic acid	HMDB0003072	N.D.	N.D.	8.0E-05	4.2E-05	N.D.	N.D.
Rhein		8.7E-05	8.3E-06	7.8E-05	9.5E-06	0.9	0.089
Ribose 5-phosphate	HMDB0001548	2.5E-04	5.0E-05	1.5E-04	4.6E-05	0.6	0.007
Ribulose 1,5-diphosphate		1.6E-04	7.9E-05	6.0E-05	5.0E-05	0.4	0.026

Ribulose 5-phosphate	HMDB0000618	2.8E-03	3.0E-04	2.8E-03	3.4E-04	1.0	0.719
Saccharopine	HMDB0000279	6.1E-04	1.9E-04	1.5E-03	2.1E-04	2.5	0.000
S-Adenosylhomocysteine	HMDB0000939	1.1E-04	5.3E-06	1.1E-04	2.1E-05	1.0	0.882
S-Adenosylmethionine	HMDB0001185	1.0E-03	8.9E-05	1.1E-03	1.2E-04	1.1	0.272
Sedoheptulose 7-phosphate	HMDB0001068	2.2E-04	3.7E-05	1.8E-04	1.5E-05	0.8	0.034
Serotonin	HMDB0000259	2.8E-04	5.5E-05	2.4E-04	8.3E-05	0.9	0.341
S-Lactoylglutathione	HMDB0001066	2.7E-04	8.2E-05	7.7E-05	1.6E-05	0.3	0.000
S-Methylmalonyl-CoA	HMDB0001269	2.4E-04	3.5E-05	2.9E-04	2.8E-05	1.2	0.022
Sorbitol 6-phosphate	HMDB0005831	5.1E-05	4.2E-06	3.5E-05	4.1E-06	0.7	0.000
Spermidine	HMDB0001257	3.7E-04	2.5E-05	4.4E-04	2.8E-05	1.2	0.002
Spermine	HMDB0001256	2.8E-04	6.3E-05	2.1E-04	2.3E-05	0.8	0.035
Stachydrine	HMDB0004827	1.7E-04	2.5E-06	1.9E-04	3.9E-05	1.1	0.527
Succinic acid	HMDB0000254	1.2E-02	1.1E-03	1.2E-02	5.6E-04	1.0	0.597
Succinyl-CoA	HMDB0001022	2.4E-04	2.3E-04	5.2E-04	1.7E-04	2.2	0.034
Taurine	HMDB0000251	1.3E-01	1.7E-02	9.0E-02	9.6E-03	0.7	0.000
Taurocholic acid	HMDB0000036	2.2E-05	2.1E-06	3.6E-05	N.D.	1.6	N.D.
Terephthalic acid	HMDB0002428	2.1E-04	1.7E-05	1.9E-04	2.4E-05	0.9	0.213
Thiamine	HMDB0000235	8.7E-05	1.3E-05	6.7E-05	1.2E-05	0.8	0.023
Thiamine diphosphate	HMDB0001372	1.7E-04	2.0E-05	1.7E-04	1.6E-05	1.0	0.632
Thiamine phosphate	HMDB0002666	6.8E-05	1.2E-05	8.8E-05	5.5E-06	1.3	0.003
Thiaproline		3.9E-04	8.0E-05	8.2E-04	1.7E-04	2.1	0.001
Thr	HMDB0000167	2.3E-02	1.8E-03	1.9E-02	3.1E-03	0.8	0.041
Threonic acid	HMDB0000943	1.1E-03	1.5E-04	9.1E-04	7.8E-05	0.8	0.013
threo-β-Methylaspartic acid		N.D.	N.D.	3.5E-04	5.1E-05	N.D.	N.D.
trans-Glutaconic acid	HMDB0000620	5.9E-04	6.6E-05	6.1E-04	4.4E-05	1.0	0.748

Trehalose 6-phosphate	HMDB0001124	2.5E-05	4.4E-06	2.7E-05	2.7E-06	1.1	0.497
Trigonelline	HMDB0000875	2.4E-04	7.1E-05	4.2E-04	8.4E-05	1.8	0.002
Trimethylamine	HMDB0000906	2.5E-04	N.D.	N.D.	N.D.	N.D.	N.D.
Trimethylamine *N*-oxide	HMDB0000925	3.8E-04	8.1E-05	4.1E-04	7.0E-05	1.1	0.592
Trp	HMDB0000929	3.1E-03	1.7E-03	2.3E-03	1.3E-03	0.8	0.418
Tyr	HMDB0000158	8.3E-03	3.5E-03	6.2E-03	2.8E-03	0.7	0.281
UDP	HMDB0000295	5.3E-04	6.7E-05	5.1E-04	1.1E-04	1.0	0.771
UDP-glucose<br>UDP-galactose	HMDB0000286<br>HMDB0000302	5.1E-03	1.1E-03	1.0E-02	5.2E-03	2.0	0.039
UDP-glucuronic acid	HMDB0000935	4.0E-04	2.1E-05	1.0E-03	5.2E-04	2.5	0.016
UDP-N-acetylgalactosamine-1<br>UDP-N-acetylglucosamine-1	HMDB0000304<br>HMDB0000290	4.8E-05	1.0E-05	1.1E-04	5.3E-05	2.2	0.027
UDP-N-acetylgalactosamine-2<br>UDP-N-acetylglucosamine-2	HMDB0000304<br>HMDB0000290	4.7E-03	3.6E-04	1.1E-02	5.4E-03	2.3	0.020
UMP	HMDB0000288	2.5E-03	3.6E-04	1.9E-03	2.1E-04	0.8	0.006
Uracil	HMDB0000300	5.0E-04	N.D.	3.4E-04	N.D.	0.7	N.D.
Urea	HMDB0000294	1.9E-01	3.9E-02	2.8E-01	2.7E-02	1.5	0.03
Uric acid	HMDB0000289	6.7E-05	1.1E-05	6.9E-05	9.6E-06	1.0	0.766
Uridine	HMDB0000296	3.9E-03	4.0E-04	5.2E-03	4.2E-04	1.3	0.000
UTP	HMDB0000285	2.8E-04	5.4E-05	2.8E-04	8.3E-05	1.0	0.869
Val	HMDB0000883	1.6E-02	6.4E-03	7.6E-03	2.3E-03	0.5	0.009
Xanthine	HMDB0000292	8.6E-04	1.1E-04	6.3E-04	1.4E-04	0.7	0.039
XC0016		7.3E-03	1.8E-03	3.2E-03	6.7E-04	0.4	0.000
XC0071		N.D.	N.D.	N.D.	N.D.	N.D.	N.D.
XC0132		1.7E-04	1.2E-05	1.6E-04	7.7E-06	1.0	0.451

β-Ala	HMDB0000056	4.7E-03	7.9E-04	4.6E-03	7.7E-04	1.0	0.836
β-Ala-Lys		N.D.	N.D.	N.D.	N.D.	N.D.	N.D.
γ-Butyrobetaine	HMDB0001161	2.6E-03	4.5E-04	1.4E-03	1.9E-04	0.5	0.000
γ-Glu-2-aminobutyric acid		3.0E-04	5.6E-05	2.6E-04	5.6E-05	0.8	0.193
γ-Glu-Cys	HMDB0001049	2.0E-04	3.8E-05	2.0E-04	1.8E-05	1.0	0.821

Supplementary Table 2. Full list and details of MSEA enriched pathways.

	total	expected	hits	Raw p	Holm p	FDR
Valine, Leucine and Isoleucine	60	6.45	24	7.53E-10	7.38E-08	7.38E-08

Degradation						
Glutamate Metabolism	49	5.26	17	3.57E-06	0.000346	0.000175
Gluconeogenesis	35	3.76	13	2.46E-05	0.00236	0.000804
Warburg Effect	58	6.23	17	4.69E-05	0.00445	0.00115
Urea Cycle	29	3.12	11	8.82E-05	0.00829	0.00173
Malate-Aspartate Shuttle	10	1.07	6	0.000198	0.0184	0.0029
Carnitine Synthesis	22	2.36	9	0.000207	0.0191	0.0029
Nucleotide Sugars Metabolism	20	2.15	8	0.000578	0.0526	0.00701
Glycolysis	25	2.69	9	0.000643	0.0579	0.00701
Arginine and Proline Metabolism	53	5.69	14	0.000783	0.0697	0.00767
Ketone Body Metabolism	13	1.4	6	0.00123	0.108	0.011
Pentose Phosphate Pathway	29	3.12	9	0.00217	0.189	0.0161
Butyrate Metabolism	19	2.04	7	0.00231	0.198	0.0161
Glycine and Serine Metabolism	59	6.34	14	0.00246	0.209	0.0161
Aspartate Metabolism	35	3.76	10	0.00246	0.209	0.0161
Propanoate Metabolism	42	4.51	11	0.00321	0.267	0.0197
Cardiolipin Biosynthesis	11	1.18	5	0.00356	0.292	0.0205
Ammonia Recycling	32	3.44	9	0.0046	0.372	0.025
Alanine Metabolism	17	1.83	6	0.00617	0.494	0.0318
Glucose-Alanine Cycle	13	1.4	5	0.00831	0.656	0.0399
Phospholipid Biosynthesis	29	3.12	8	0.00856	0.667	0.0399
Thiamine Metabolism	9	0.967	4	0.0104	0.799	0.0434
De Novo Triacylglycerol Biosynthesis	9	0.967	4	0.0104	0.799	0.0434
Lysine Degradation	30	3.22	8	0.0106	0.799	0.0434
Phosphatidylcholine Biosynthesis	14	1.5	5	0.0118	0.876	0.0464
Threonine and 2-Oxobutanoate Degradation	20	2.15	6	0.0148	1	0.0556
Cysteine Metabolism	26	2.79	7	0.0159	1	0.0557
Citric Acid Cycle	32	3.44	8	0.0159	1	0.0557
Glutathione Metabolism	21	2.26	6	0.0189	1	0.0626
Amino Sugar Metabolism	33	3.54	8	0.0192	1	0.0626
Purine Metabolism	74	7.95	14	0.0205	1	0.0648
Glycerol Phosphate Shuttle	11	1.18	4	0.0229	1	0.0702
Pyruvate Metabolism	48	5.16	10	0.0261	1	0.0775
Phosphatidylethanolamine Biosynthesis	12	1.29	4	0.0316	1	0.091
Nicotinate and Nicotinamide Metabolism	37	3.97	8	0.0369	1	0.103
Starch and Sucrose Metabolism	31	3.33	7	0.0403	1	0.11
Mitochondrial Electron Transport Chain	19	2.04	5	0.0444	1	0.118
Fructose and Mannose Degradation	32	3.44	7	0.0472	1	0.122

Phytanic Acid Peroxisomal Oxidation	26	2.79	6	0.0513	1	0.129
Lactose Synthesis	20	2.15	5	0.0542	1	0.133
Pantothenate and CoA Biosynthesis	21	2.26	5	0.0653	1	0.156
Galactose Metabolism	38	4.08	7	0.103	1	0.241
Glycerolipid Metabolism	25	2.69	5	0.121	1	0.276
Oxidation of Branched Chain Fatty Acids	26	2.79	5	0.138	1	0.293
Inositol Phosphate Metabolism	26	2.79	5	0.138	1	0.293
Plasmalogen Synthesis	26	2.79	5	0.138	1	0.293
Beta-Alanine Metabolism	34	3.65	6	0.149	1	0.31
Mitochondrial Beta-Oxidation of Short Chain Saturated Fatty Acids	27	2.9	5	0.155	1	0.317
Methionine Metabolism	43	4.62	7	0.169	1	0.339
Transfer of Acetyl Groups into Mitochondria	22	2.36	4	0.204	1	0.4
Biotin Metabolism	8	0.859	2	0.209	1	0.401
Phenylacetate Metabolism	9	0.967	2	0.251	1	0.463
Homocysteine Degradation	9	0.967	2	0.251	1	0.463
Beta Oxidation of Very Long Chain Fatty Acids	17	1.83	3	0.273	1	0.492
Inositol Metabolism	33	3.54	5	0.276	1	0.492
Pyruvaldehyde Degradation	10	1.07	2	0.293	1	0.512
Spermidine and Spermine Biosynthesis	18	1.93	3	0.304	1	0.522
Ethanol Degradation	19	2.04	3	0.335	1	0.565
Phenylalanine and Tyrosine Metabolism	28	3.01	4	0.354	1	0.569
Selenoamino Acid Metabolism	28	3.01	4	0.354	1	0.569
Mitochondrial Beta-Oxidation of Long Chain Saturated Fatty Acids	28	3.01	4	0.354	1	0.569
Taurine and Hypotaurine Metabolism	12	1.29	2	0.375	1	0.591
Folate Metabolism	29	3.12	4	0.38	1	0.591
Porphyrin Metabolism	40	4.3	5	0.433	1	0.663
Androstenedione Metabolism	24	2.58	3	0.486	1	0.719
Estrone Metabolism	24	2.58	3	0.486	1	0.719
Histidine Metabolism	43	4.62	5	0.499	1	0.719
Fatty acid Metabolism	43	4.62	5	0.499	1	0.719
Phosphatidylinositol Phosphate Metabolism	17	1.83	2	0.561	1	0.796
Mitochondrial Beta-Oxidation of Medium Chain Saturated Fatty Acids	27	2.9	3	0.569	1	0.796
Pterine Biosynthesis	29	3.12	3	0.619	1	0.85
Pyrimidine Metabolism	59	6.34	6	0.624	1	0.85

Sphingolipid Metabolism	40	4.3	4	0.64	1	0.85
Lactose Degradation	9	0.967	1	0.642	1	0.85
Riboflavin Metabolism	20	2.15	2	0.652	1	0.852
Betaine Metabolism	21	2.26	2	0.679	1	0.875
Androgen and Estrogen Metabolism	33	3.54	3	0.708	1	0.882
D-Arginine and D-Ornithine Metabolism	11	1.18	1	0.715	1	0.882
Trehalose Degradation	11	1.18	1	0.715	1	0.882
Bile Acid Biosynthesis	65	6.98	6	0.72	1	0.882
Caffeine Metabolism	24	2.58	2	0.749	1	0.907
Tryptophan Metabolism	60	6.45	5	0.794	1	0.949
Tyrosine Metabolism	72	7.73	6	0.809	1	0.955
Vitamin B6 Metabolism	20	2.15	1	0.899	1	1
Fatty Acid Elongation In Mitochondria	35	3.76	2	0.906	1	1
Steroid Biosynthesis	48	5.16	3	0.907	1	1
Sulfate/Sulfite Metabolism	22	2.36	1	0.92	1	1
Retinol Metabolism	37	3.97	2	0.922	1	1
Steroidogenesis	43	4.62	2	0.957	1	1
Fatty Acid Biosynthesis	35	3.76	1	0.983	1	1
Arachidonic Acid Metabolism	69	7.41	3	0.986	1	1

Supplementary Table 3. Complete list of metabolic pathways identified from MetaboAnalyst.

	Total	Expected	Hits	Raw p	Holm adjust	FDR	Impact
**Glycolysis or Gluconeogenesis**	61	5.875	26	5.84E-12	4.90E-10	4.90E-10	1.3833
**Arginine biosynthesis**	27	2.6004	15	2.25E-09	1.87E-07	8.33E-08	1.1538
**Valine, leucine and isoleucine degradation**	88	8.4754	28	2.98E-09	2.44E-07	8.33E-08	1.3103

**Alanine, aspartate and glutamate metabolism**	61	5.875	20	3.65E-07	2.95E-05	7.66E-06	1.1333
**Inositol phosphate metabolism**	69	6.6455	21	7.57E-07	6.05E-05	1.27E-05	0.69118
**Phosphatidylinositol signaling system**	74	7.127	20	1.08E-05	0.00085557	0.00015162	0.71233
**D-Glutamine and D-glutamate metabolism**	10	0.96311	6	0.00011306	0.0088184	0.0013567	1.5556
**Galactose metabolism**	51	4.9119	14	0.00020066	0.015451	0.0020407	1.06
**Butanoate metabolism**	29	2.793	10	0.00021865	0.016617	0.0020407	1.0357
**Taurine and hypotaurine metabolism**	16	1.541	7	0.00037661	0.028246	0.0031635	0.73333
**Valine, leucine and isoleucine biosynthesis**	12	1.1557	6	0.00042096	0.031151	0.0032146	1.2727
**Lysine degradation**	49	4.7193	13	0.00049066	0.035818	0.0034346	0.72917
**Linoleic acid metabolism**	17	1.6373	7	0.00058829	0.042357	0.0038012	1
**Sphingolipid metabolism**	58	5.5861	14	0.00084852	0.060245	0.0050911	0.33333
**Pentose phosphate pathway**	47	4.5266	12	0.0011739	0.082174	0.0065739	1.1304
**Nitrogen metabolism**	10	0.96311	5	0.0013358	0.09217	0.0066304	1
**Arachidonic acid metabolism**	81	7.8012	17	0.0013419	0.09217	0.0066304	0.6125
**Glutathione metabolism**	56	5.3934	13	0.0019082	0.12785	0.008905	0.78182
**Amino sugar and nucleotide sugar metabolism**	79	7.6086	16	0.0027389	0.18077	0.012109	0.78205
**Cysteine and methionine metabolism**	71	6.8381	14	0.0064153	0.41699	0.026944	0.61429
**beta-Alanine metabolism**	44	4.2377	10	0.0073889	0.47289	0.029555	0.4186
**Fructose and mannose metabolism**	40	3.8525	9	0.011632	0.73281	0.044413	0.71795
**Propanoate metabolism**	48	4.623	10	0.013814	0.85645	0.05045	0.89362
**Glyoxylate and dicarboxylate metabolism**	56	5.3934	11	0.015507	0.94596	0.053762	0.36364
**Citrate cycle (TCA cycle)**	42	4.0451	9	0.016001	0.96003	0.053762	1.1707
**Aminoacyl-tRNA biosynthesis**	74	7.127	13	0.021926	1	0.070838	0.19178
**Arginine and proline metabolism**	78	7.5123	13	0.0326	1	0.1015	0.4935

				5		8	1
**Pantothenate and CoA biosynthesis**	34	3.2746	7	0.039377	1	0.11813	0.48485
**Pyruvate metabolism**	45	4.334	8	0.061704	1	0.17808	0.68182
**Synthesis and degradation of ketone bodies**	10	0.96311	3	0.063601	1	0.17808	1.3333
**Glycine, serine and threonine metabolism**	68	6.5492	10	0.11279	1	0.28955	0.56716
**Starch and sucrose metabolism**	43	4.1414	7	0.11332	1	0.28955	0.47619
**Glycerolipid metabolism**	35	3.3709	6	0.11375	1	0.28955	0.47059
**Ascorbate and aldarate metabolism**	13	1.252	3	0.12253	1	0.30272	0.58333
**Phenylalanine metabolism**	21	2.0225	4	0.1367	1	0.32808	0.4
**Thiamine metabolism**	14	1.3484	3	0.14539	1	0.33925	0.53846
**Retinol metabolism**	47	4.5266	7	0.16044	1	0.36425	0.21739
**Histidine metabolism**	32	3.082	5	0.18913	1	0.40737	0.22581
**Pentose and glucuronate interconversions**	32	3.082	5	0.18913	1	0.40737	0.58065
**Glycerophospholipid metabolism**	86	8.2828	11	0.20011	1	0.42024	0.45882
**Phenylalanine, tyrosine and tryptophan biosynthesis**	11	1.0594	2	0.2869	1	0.58779	0.8
**Tyrosine metabolism**	88	8.4754	10	0.33861	1	0.67722	0.37931
**Pyrimidine metabolism**	99	9.5348	11	0.35434	1	0.6922	0.80612
**Tryptophan metabolism**	84	8.0902	9	0.42054	1	0.80286	0.36145
**Drug metabolism - cytochrome P450**	98	9.4385	10	0.47399	1	0.88478	0.19588
**Vitamin B6 metabolism**	21	2.0225	2	0.61504	1	1	0.35
**alpha-Linolenic acid metabolism**	22	2.1189	2	0.64085	1	1	0.095238
**Purine metabolism**	166	15.988	15	0.64734	1	1	0.77576
**Terpenoid backbone biosynthesis**	36	3.4672	3	0.68896	1	1	0.2
**Fatty acid degradation**	102	9.8238	8	0.78266	1	1	0.69307

**Nicotinate and nicotinamide metabolism**	42	4.0451	3	0.78559	1	1	0.5122
**Metabolism of xenobiotics by cytochrome P450**	145	13.965	11	0.84325	1	1	0.38889
**Seleno compound metabolism**	35	3.3709	2	0.86527	1	1	0.14706
**Biotin metabolism**	21	2.0225	1	0.88186	1	1	0.05
**Ether lipid metabolism**	39	3.7561	2	0.90251	1	1	0.10526
**Drug metabolism - other enzymes**	70	6.7418	4	0.91785	1	1	0.10145
**Folate biosynthesis**	61	5.875	3	0.94347	1	1	0.16667
**Porphyrin and chlorophyll metabolism**	53	5.1045	2	0.97021	1	1	0.038462
**Various types of N-glycan biosynthesis**	36	3.4672	1	0.97461	1	1	0.057143
**Biosynthesis of unsaturated fatty acids**	47	4.5266	1	0.99183	1	1	0.47826
**Fatty acid elongation**	75	7.2234	2	0.99588	1	1	0.2973
**Primary bile acid biosynthesis**	92	8.8607	2	0.99916	1	1	0.043956
**N-Glycan biosynthesis**	77	7.416	1	0.99964	1	1	0.026316
**Glycosphingolipid biosynthesis - lacto and neolacto series**	121	11.654	1	1	1	1	0.033333
**Fatty acid biosynthesis**	129	12.424	1	1	1	1	0.046875
**Steroid hormone biosynthesis**	199	19.166	3	1	1	1	0.060606

Supplementary Table 4. List of signalling molecules whose phosphorylation increased or decreased in astrocytes.
For Figure 3A
Upregulated phosphoprotein

**Protein**	**Phosphorylation site**	**Effect**
AKT	S246	Activation
AKT	T308	Activation
AKT	T450	Activation
AKT	S473	Activation

AKT1	S246	Activation
AKT2	S474	Activation
GSK3β	S9	Inhibition
MEK2	S295	Inhibition
mTOR	T2446	Activation
mTOR	S2448	Activation
mTOR	S2481	Activation
p85α	Y607	Activation
p85α	Y467/Y199	Activation

Downregulated phosphoprotein

**Protein**	**Phosphorylation site**	**Effect**
β-catenin	S33	Inhibition
β-catenin	S37	Inhibition
β-catenin	Y489	Activation
β-catenin	Y654	Activation
ERK8	T175/T177	Activation
MAP3K7	T187	Activation

For Figure 4A
Upregulated phosphoprotein

**Protein**	**Phosphorylation site**	**Effect**
CamkIIα	T286	Activation
CamkIIβ/γ/δ	T287	Activation
NFAT1	S326	Activation
NFAT3	S168/S170	Activation
PKCα	T638	Activation
PKCα	Y657	Activation

Downregulated phosphoprotein

**Protein**	**Phosphorylation site**	**Effect**
AKT	T308	Activation
AKT	S473	Activation
AKT	Y474	Activation
AKT2	S474	Activation
mTOR	T2446	Activation
mTOR	S2448	Activation
mTOR	S2481	Activation
p85α	Y607	Activation
p85α	Y467/Y199	Activation

Supplementary Table 5. List of 6,812 NFATC1 targets from the ENCODE database.

Symbol	Name
AAAS	achalasia, adrenocortical insufficiency, alacrimia
AAGAB	alpha- and gamma-adaptin binding protein
AAK1	AP2 associated kinase 1
AAMDC	adipogenesis associated, Mth938 domain containing
AAMP	angio-associated, migratory cell protein
AAR2	AAR2 splicing factor homolog (S. cerevisiae)
AARS	alanyl-tRNA synthetase

**AARS2**	**alanyl-tRNA synthetase 2, mitochondrial**
**AASDH**	**aminoadipate-semialdehyde dehydrogenase**
**AATF**	**apoptosis antagonizing transcription factor**
**ABCA11P**	**ATP-binding cassette, sub-family A (ABC1), member 11, pseudogene**
**ABCA12**	**ATP-binding cassette, sub-family A (ABC1), member 12**
**ABCA17P**	**ATP-binding cassette, sub-family A (ABC1), member 17, pseudogene**
**ABCA3**	**ATP-binding cassette, sub-family A (ABC1), member 3**
**ABCA7**	**ATP-binding cassette, sub-family A (ABC1), member 7**
**ABCB10**	**ATP-binding cassette, sub-family B (MDR/TAP), member 10**
**ABCB4**	**ATP-binding cassette, sub-family B (MDR/TAP), member 4**
**ABCB6**	**ATP-binding cassette, sub-family B (MDR/TAP), member 6 (Langereis blood group)**
**ABCC10**	**ATP-binding cassette, sub-family C (CFTR/MRP), member 10**
**ABCC12**	**ATP-binding cassette, sub-family C (CFTR/MRP), member 12**
**ABCC4**	**ATP-binding cassette, sub-family C (CFTR/MRP), member 4**
**ABCD2**	**ATP-binding cassette, sub-family D (ALD), member 2**
**ABCD4**	**ATP-binding cassette, sub-family D (ALD), member 4**
**ABCE1**	**ATP-binding cassette, sub-family E (OABP), member 1**
**ABCF2**	**ATP-binding cassette, sub-family F (GCN20), member 2**
**ABCF3**	**ATP-binding cassette, sub-family F (GCN20), member 3**
**ABHD14A**	**abhydrolase domain containing 14A**
**ABHD14A-ACY1**	**ABHD14A-ACY1 readthrough (NMD candidate)**
**ABHD14B**	**abhydrolase domain containing 14B**
**ABHD15**	**abhydrolase domain containing 15**
**ABHD17B**	**abhydrolase domain containing 17B**
**ABHD5**	**abhydrolase domain containing 5**
**ABI1**	**abl-interactor 1**
**ABI2**	**abl-interactor 2**
**ABI3**	**ABI family, member 3**
**ABL1**	**ABL proto-oncogene 1, non-receptor tyrosine kinase**
**ABRACL**	**ABRA C-terminal like**
**ABT1**	**activator of basal transcription 1**
**ABTB1**	**ankyrin repeat and BTB (POZ) domain containing 1**
**ACAA1**	**acetyl-CoA acyltransferase 1**
**ACACA**	**acetyl-CoA carboxylase alpha**
**ACAD10**	**acyl-CoA dehydrogenase family, member 10**
**ACAD11**	**acyl-CoA dehydrogenase family, member 11**
**ACAD9**	**acyl-CoA dehydrogenase family, member 9**
**ACADM**	**acyl-CoA dehydrogenase, C-4 to C-12 straight chain**
**ACADS**	**acyl-CoA dehydrogenase, C-2 to C-3 short chain**
**ACADVL**	**acyl-CoA dehydrogenase, very long chain**
**ACAP3**	**ArfGAP with coiled-coil, ankyrin repeat and PH domains 3**

**ACAT1**	**acetyl-CoA acetyltransferase 1**
**ACAT2**	**acetyl-CoA acetyltransferase 2**
**ACBD3**	**acyl-CoA binding domain containing 3**
**ACBD5**	**acyl-CoA binding domain containing 5**
**ACBD6**	**acyl-CoA binding domain containing 6**
**ACCS**	**1-aminocyclopropane-1-carboxylate synthase homolog (Arabidopsis)(non-functional)**
**ACD**	**adrenocortical dysplasia homolog (mouse)**
**ACIN1**	**apoptotic chromatin condensation inducer 1**
**ACKR3**	**atypical chemokine receptor 3**
**ACO2**	**aconitase 2, mitochondrial**
**ACOT13**	**acyl-CoA thioesterase 13**
**ACOT2**	**acyl-CoA thioesterase 2**
**ACOT8**	**acyl-CoA thioesterase 8**
**ACOX1**	**acyl-CoA oxidase 1, palmitoyl**
**ACOX3**	**acyl-CoA oxidase 3, pristanoyl**
**ACP2**	**acid phosphatase 2, lysosomal**
**ACPT**	**acid phosphatase, testicular**
**ACSL1**	**acyl-CoA synthetase long-chain family member 1**
**ACTA2**	**actin, alpha 2, smooth muscle, aorta**
**ACTB**	**actin, beta**
**ACTG1**	**actin gamma 1**
**ACTL10**	**actin-like 10**
**ACTL6A**	**actin-like 6A**
**ACTN4**	**actinin, alpha 4**
**ACTR1B**	**ARP1 actin-related protein 1 homolog B, centractin beta (yeast)**
**ACTR2**	**ARP2 actin-related protein 2 homolog (yeast)**
**ACTR3**	**ARP3 actin-related protein 3 homolog (yeast)**
**ACTR3C**	**ARP3 actin-related protein 3 homolog C (yeast)**
**ACTR5**	**ARP5 actin-related protein 5 homolog (yeast)**
**ACTR6**	**ARP6 actin-related protein 6 homolog (yeast)**
**ACTR8**	**ARP8 actin-related protein 8 homolog (yeast)**
**ACY3**	**aminoacylase 3**
**ACYP2**	**acylphosphatase 2, muscle type**
**ADAL**	**adenosine deaminase-like**
**ADAM15**	**ADAM metallopeptidase domain 15**
**ADAM17**	**ADAM metallopeptidase domain 17**
**ADAM22**	**ADAM metallopeptidase domain 22**
**ADAM8**	**ADAM metallopeptidase domain 8**
**ADAR**	**adenosine deaminase, RNA-specific**
**ADARB2-AS1**	**ADARB2 antisense RNA 1**
**ADAT1**	**adenosine deaminase, tRNA-specific 1**

**ADAT2**	**adenosine deaminase, tRNA-specific 2**
**ADAT3**	**adenosine deaminase, tRNA-specific 3**
**ADCK1**	**aarF domain containing kinase 1**
**ADCK3**	**aarF domain containing kinase 3**
**ADCK4**	**aarF domain containing kinase 4**
**ADCK5**	**aarF domain containing kinase 5**
**ADD1**	**adducin 1 (alpha)**
**ADGRE2**	**adhesion G protein-coupled receptor E2**
**ADGRE5**	**adhesion G protein-coupled receptor E5**
**ADGRF3**	**adhesion G protein-coupled receptor F3**
**ADGRG5**	**adhesion G protein-coupled receptor G5**
**ADH5**	**alcohol dehydrogenase 5 (class III), chi polypeptide**
**ADHFE1**	**alcohol dehydrogenase, iron containing, 1**
**ADI1**	**acireductone dioxygenase 1**
**ADIG**	**adipogenin**
**ADIPOR1**	**adiponectin receptor 1**
**ADM5**	**adrenomedullin 5 (putative)**
**ADNP**	**activity-dependent neuroprotector homeobox**
**ADO**	**2-aminoethanethiol (cysteamine) dioxygenase**
**ADPGK**	**ADP-dependent glucokinase**
**ADPRH**	**ADP-ribosylarginine hydrolase**
**ADPRHL2**	**ADP-ribosylhydrolase like 2**
**ADRBK1**	**adrenergic, beta, receptor kinase 1**
**ADRM1**	**adhesion regulating molecule 1**
**ADSL**	**adenylosuccinate lyase**
**ADSS**	**adenylosuccinate synthase**
**AEN**	**apoptosis enhancing nuclease**
**AFF1**	**AF4/FMR2 family, member 1**
**AFF3**	**AF4/FMR2 family, member 3**
**AFF4**	**AF4/FMR2 family, member 4**
**AFG3L1P**	**AFG3-like AAA ATPase 1, pseudogene**
**AFMID**	**arylformamidase**
**AFTPH**	**aftiphilin**
**AGA**	**aspartylglucosaminidase**
**AGBL2**	**ATP/GTP binding protein-like 2**
**AGBL5**	**ATP/GTP binding protein-like 5**
**AGER**	**advanced glycosylation end product-specific receptor**
**AGFG2**	**ArfGAP with FG repeats 2**
**AGGF1**	**angiogenic factor with G patch and FHA domains 1**
**AGL**	**amylo-alpha-1, 6-glucosidase, 4-alpha-glucanotransferase**
**AGO1**	**argonaute RISC catalytic component 1**

**AGO2**	**argonaute RISC catalytic component 2**
**AGO3**	**argonaute RISC catalytic component 3**
**AGPAT3**	**1-acylglycerol-3-phosphate O-acyltransferase 3**
**AGPAT5**	**1-acylglycerol-3-phosphate O-acyltransferase 5**
**AGPS**	**alkylglycerone phosphate synthase**
**AHCYL2**	**adenosylhomocysteinase-like 2**
**AICDA**	**activation-induced cytidine deaminase**
**AIDA**	**axin interactor, dorsalization associated**
**AIM1**	**absent in melanoma 1**
**AIM2**	**absent in melanoma 2**
**AIMP1**	**aminoacyl tRNA synthetase complex-interacting multifunctional protein 1**
**AIMP2**	**aminoacyl tRNA synthetase complex-interacting multifunctional protein 2**
**AIP**	**aryl hydrocarbon receptor interacting protein**
**AK2**	**adenylate kinase 2**
**AKAP1**	**A kinase (PRKA) anchor protein 1**
**AKAP10**	**A kinase (PRKA) anchor protein 10**
**AKAP5**	**A kinase (PRKA) anchor protein 5**
**AKAP8**	**A kinase (PRKA) anchor protein 8**
**AKAP8L**	**A kinase (PRKA) anchor protein 8-like**
**AKAP9**	**A kinase (PRKA) anchor protein 9**
**AKIP1**	**A kinase (PRKA) interacting protein 1**
**AKIRIN2**	**akirin 2**
**AKR1A1**	**aldo-keto reductase family 1, member A1 (aldehyde reductase)**
**AKR1B15**	**aldo-keto reductase family 1, member B15**
**AKT1S1**	**AKT1 substrate 1 (proline-rich)**
**AKT2**	**v-akt murine thymoma viral oncogene homolog 2**
**AKTIP**	**AKT interacting protein**
**ALAS1**	**5'-aminolevulinate synthase 1**
**ALCAM**	**activated leukocyte cell adhesion molecule**
**ALDH16A1**	**aldehyde dehydrogenase 16 family, member A1**
**ALDH18A1**	**aldehyde dehydrogenase 18 family, member A1**
**ALDH3A1**	**aldehyde dehydrogenase 3 family, member A1**
**ALDH4A1**	**aldehyde dehydrogenase 4 family, member A1**
**ALDH6A1**	**aldehyde dehydrogenase 6 family, member A1**
**ALDH9A1**	**aldehyde dehydrogenase 9 family, member A1**
**ALDOA**	**aldolase A, fructose-bisphosphate**
**ALG10B**	**ALG10B, alpha-1,2-glucosyltransferase**
**ALG14**	**ALG14, UDP-N-acetylglucosaminyltransferase subunit**
**ALG2**	**ALG2, alpha-1,3/1,6-mannosyltransferase**
**ALG3**	**ALG3, alpha-1,3- mannosyltransferase**
**ALG5**	**ALG5, dolichyl-phosphate beta-glucosyltransferase**

ALKBH1	alkB, alkylation repair homolog 1 (E. coli)
ALKBH2	alkB, alkylation repair homolog 2 (E. coli)
ALKBH3	alkB, alkylation repair homolog 3 (E. coli)
ALKBH4	alkB, alkylation repair homolog 4 (E. coli)
ALKBH5	AlkB family member 5, RNA demethylase
ALKBH7	alkB, alkylation repair homolog 7 (E. coli)
ALOXE3	arachidonate lipoxygenase 3
ALYREF	Aly/REF export factor
AMACR	alpha-methylacyl-CoA racemase
AMD1	adenosylmethionine decarboxylase 1
AMDHD2	amidohydrolase domain containing 2
AMH	anti-Mullerian hormone
AMIGO3	adhesion molecule with Ig-like domain 3
AMN1	antagonist of mitotic exit network 1 homolog (S. cerevisiae)
AMPD2	adenosine monophosphate deaminase 2
AMT	aminomethyltransferase
AMZ2	archaelysin family metallopeptidase 2
AMZ2P1	archaelysin family metallopeptidase 2 pseudogene 1
ANAPC1	anaphase promoting complex subunit 1
ANAPC10	anaphase promoting complex subunit 10
ANAPC11	anaphase promoting complex subunit 11
ANAPC13	anaphase promoting complex subunit 13
ANAPC15	anaphase promoting complex subunit 15
ANAPC16	anaphase promoting complex subunit 16
ANAPC2	anaphase promoting complex subunit 2
ANAPC4	anaphase promoting complex subunit 4
ANAPC7	anaphase promoting complex subunit 7
ANG	angiogenin, ribonuclease, RNase A family, 5
ANGPTL6	angiopoietin-like 6
ANKAR	ankyrin and armadillo repeat containing
ANKFY1	ankyrin repeat and FYVE domain containing 1
ANKHD1	ankyrin repeat and KH domain containing 1
ANKHD1-EIF4EBP3	ANKHD1-EIF4EBP3 readthrough
ANKLE1	ankyrin repeat and LEM domain containing 1
ANKLE2	ankyrin repeat and LEM domain containing 2
ANKMY2	ankyrin repeat and MYND domain containing 2
ANKRD10	ankyrin repeat domain 10
ANKRD11	ankyrin repeat domain 11
ANKRD12	ankyrin repeat domain 12
ANKRD13A	ankyrin repeat domain 13A
ANKRD13B	ankyrin repeat domain 13B

ANKRD13C	ankyrin repeat domain 13C
ANKRD13D	ankyrin repeat domain 13 family, member D
ANKRD16	ankyrin repeat domain 16
ANKRD17	ankyrin repeat domain 17
ANKRD26	ankyrin repeat domain 26
ANKRD32	ankyrin repeat domain 32
ANKRD33B	ankyrin repeat domain 33B
ANKRD37	ankyrin repeat domain 37
ANKRD39	ankyrin repeat domain 39
ANKRD40	ankyrin repeat domain 40
ANKRD42	ankyrin repeat domain 42
ANKRD46	ankyrin repeat domain 46
ANKRD54	ankyrin repeat domain 54
ANKS1A	ankyrin repeat and sterile alpha motif domain containing 1A
ANKZF1	ankyrin repeat and zinc finger domain containing 1
ANO6	anoctamin 6
ANO8	anoctamin 8
ANP32A	acidic (leucine-rich) nuclear phosphoprotein 32 family, member A
ANP32B	acidic (leucine-rich) nuclear phosphoprotein 32 family, member B
ANP32E	acidic (leucine-rich) nuclear phosphoprotein 32 family, member E
ANXA2	annexin A2
ANXA2R	annexin A2 receptor
ANXA4	annexin A4
ANXA6	annexin A6
ANXA7	annexin A7
AOC2	amine oxidase, copper containing 2 (retina-specific)
AP1AR	adaptor-related protein complex 1 associated regulatory protein
AP1G1	adaptor-related protein complex 1, gamma 1 subunit
AP1G2	adaptor-related protein complex 1, gamma 2 subunit
AP1S2	adaptor-related protein complex 1, sigma 2 subunit
AP1S3	adaptor-related protein complex 1, sigma 3 subunit
AP2B1	adaptor-related protein complex 2, beta 1 subunit
AP2M1	adaptor-related protein complex 2, mu 1 subunit
AP2S1	adaptor-related protein complex 2, sigma 1 subunit
AP3B1	adaptor-related protein complex 3, beta 1 subunit
AP3M2	adaptor-related protein complex 3, mu 2 subunit
AP3S1	adaptor-related protein complex 3, sigma 1 subunit
AP3S2	adaptor-related protein complex 3, sigma 2 subunit
AP4B1	adaptor-related protein complex 4, beta 1 subunit
AP4E1	adaptor-related protein complex 4, epsilon 1 subunit
AP4M1	adaptor-related protein complex 4, mu 1 subunit

**AP5S1**	**adaptor-related protein complex 5, sigma 1 subunit**
**AP5Z1**	**adaptor-related protein complex 5, zeta 1 subunit**
**APAF1**	**apoptotic peptidase activating factor 1**
**APBA3**	**amyloid beta (A4) precursor protein-binding, family A, member 3**
**APBB1IP**	**amyloid beta (A4) precursor protein-binding, family B, member 1 interacting protein**
**APBB2**	**amyloid beta (A4) precursor protein-binding, family B, member 2**
**APBB3**	**amyloid beta (A4) precursor protein-binding, family B, member 3**
**APEH**	**acylaminoacyl-peptide hydrolase**
**APEX1**	**APEX nuclease (multifunctional DNA repair enzyme) 1**
**API5**	**apoptosis inhibitor 5**
**APITD1**	**apoptosis-inducing, TAF9-like domain 1**
**APITD1-CORT**	**APITD1-CORT readthrough**
**APOA1BP**	**apolipoprotein A-I binding protein**
**APOBEC3H**	**apolipoprotein B mRNA editing enzyme, catalytic polypeptide-like 3H**
**APOBR**	**apolipoprotein B receptor**
**APOC4**	**apolipoprotein C-IV**
**APOC4-APOC2**	**APOC4-APOC2 readthrough (NMD candidate)**
**APOE**	**apolipoprotein E**
**APOL2**	**apolipoprotein L, 2**
**APOLD1**	**apolipoprotein L domain containing 1**
**APOM**	**apolipoprotein M**
**APOO**	**apolipoprotein O**
**APPL1**	**adaptor protein, phosphotyrosine interaction, PH domain and leucine zipper containing 1**
**APRT**	**adenine phosphoribosyltransferase**
**APTX**	**aprataxin**
**AQP1**	**aquaporin 1 (Colton blood group)**
**AQP11**	**aquaporin 11**
**AQP3**	**aquaporin 3 (Gill blood group)**
**AQR**	**aquarius intron-binding spliceosomal factor**
**ARAP2**	**ArfGAP with RhoGAP domain, ankyrin repeat and PH domain 2**
**ARF1**	**ADP-ribosylation factor 1**
**ARF3**	**ADP-ribosylation factor 3**
**ARF4**	**ADP-ribosylation factor 4**
**ARF6**	**ADP-ribosylation factor 6**
**ARFGAP2**	**ADP-ribosylation factor GTPase activating protein 2**
**ARFGAP3**	**ADP-ribosylation factor GTPase activating protein 3**
**ARFIP1**	**ADP-ribosylation factor interacting protein 1**
**ARFIP2**	**ADP-ribosylation factor interacting protein 2**
**ARHGAP1**	**Rho GTPase activating protein 1**
**ARHGAP10**	**Rho GTPase activating protein 10**

**ARHGAP15**	**Rho GTPase activating protein 15**
**ARHGAP17**	**Rho GTPase activating protein 17**
**ARHGAP19**	**Rho GTPase activating protein 19**
**ARHGAP19-SLIT1**	**ARHGAP19-SLIT1 readthrough (NMD candidate)**
**ARHGAP21**	**Rho GTPase activating protein 21**
**ARHGAP22**	**Rho GTPase activating protein 22**
**ARHGAP30**	**Rho GTPase activating protein 30**
**ARHGAP31**	**Rho GTPase activating protein 31**
**ARHGAP4**	**Rho GTPase activating protein 4**
**ARHGAP9**	**Rho GTPase activating protein 9**
**ARHGDIA**	**Rho GDP dissociation inhibitor (GDI) alpha**
**ARHGDIB**	**Rho GDP dissociation inhibitor (GDI) beta**
**ARHGEF1**	**Rho guanine nucleotide exchange factor (GEF) 1**
**ARHGEF12**	**Rho guanine nucleotide exchange factor (GEF) 12**
**ARHGEF2**	**Rho/Rac guanine nucleotide exchange factor (GEF) 2**
**ARID1A**	**AT rich interactive domain 1A (SWI-like)**
**ARID1B**	**AT rich interactive domain 1B (SWI1-like)**
**ARID2**	**AT rich interactive domain 2 (ARID, RFX-like)**
**ARID3A**	**AT rich interactive domain 3A (BRIGHT-like)**
**ARID3B**	**AT rich interactive domain 3B (BRIGHT-like)**
**ARID4A**	**AT rich interactive domain 4A (RBP1-like)**
**ARID4B**	**AT rich interactive domain 4B (RBP1-like)**
**ARID5A**	**AT rich interactive domain 5A (MRF1-like)**
**ARID5B**	**AT rich interactive domain 5B (MRF1-like)**
**ARIH1**	**ariadne RBR E3 ubiquitin protein ligase 1**
**ARIH2**	**ariadne RBR E3 ubiquitin protein ligase 2**
**ARIH2OS**	**ariadne homolog 2 opposite strand**
**ARL1**	**ADP-ribosylation factor-like 1**
**ARL11**	**ADP-ribosylation factor-like 11**
**ARL14EP**	**ADP-ribosylation factor-like 14 effector protein**
**ARL15**	**ADP-ribosylation factor-like 15**
**ARL2**	**ADP-ribosylation factor-like 2**
**ARL2-SNX15**	**ARL2-SNX15 readthrough (NMD candidate)**
**ARL2BP**	**ADP-ribosylation factor-like 2 binding protein**
**ARL3**	**ADP-ribosylation factor-like 3**
**ARL5A**	**ADP-ribosylation factor-like 5A**
**ARL5B**	**ADP-ribosylation factor-like 5B**
**ARL6IP1**	**ADP-ribosylation factor-like 6 interacting protein 1**
**ARL6IP4**	**ADP-ribosylation factor-like 6 interacting protein 4**
**ARL6IP5**	**ADP-ribosylation factor-like 6 interacting protein 5**
**ARL6IP6**	**ADP-ribosylation factor-like 6 interacting protein 6**

**ARL8A**	**ADP-ribosylation factor-like 8A**
**ARL8B**	**ADP-ribosylation factor-like 8B**
**ARMC1**	**armadillo repeat containing 1**
**ARMC5**	**armadillo repeat containing 5**
**ARMC6**	**armadillo repeat containing 6**
**ARMC7**	**armadillo repeat containing 7**
**ARMC8**	**armadillo repeat containing 8**
**ARMC9**	**armadillo repeat containing 9**
**ARMT1**	**acidic residue methyltransferase 1**
**ARPC1A**	**actin related protein 2/3 complex, subunit 1A, 41kDa**
**ARPC1B**	**actin related protein 2/3 complex, subunit 1B, 41kDa**
**ARPC2**	**actin related protein 2/3 complex, subunit 2, 34kDa**
**ARPC3**	**actin related protein 2/3 complex, subunit 3, 21kDa**
**ARPC4**	**actin related protein 2/3 complex, subunit 4, 20kDa**
**ARPC4-TTLL3**	**ARPC4-TTLL3 readthrough**
**ARPC5**	**actin related protein 2/3 complex, subunit 5, 16kDa**
**ARPC5L**	**actin related protein 2/3 complex, subunit 5-like**
**ARRDC1-AS1**	**ARRDC1 antisense RNA 1**
**ARRDC3**	**arrestin domain containing 3**
**ARSB**	**arylsulfatase B**
**ARSK**	**arylsulfatase family, member K**
**ART3**	**ADP-ribosyltransferase 3**
**ASAH1**	**N-acylsphingosine amidohydrolase (acid ceramidase) 1**
**ASAP1**	**ArfGAP with SH3 domain, ankyrin repeat and PH domain 1**
**ASB1**	**ankyrin repeat and SOCS box containing 1**
**ASB13**	**ankyrin repeat and SOCS box containing 13**
**ASB16-AS1**	**ASB16 antisense RNA 1**
**ASB2**	**ankyrin repeat and SOCS box containing 2**
**ASB3**	**ankyrin repeat and SOCS box containing 3**
**ASB6**	**ankyrin repeat and SOCS box containing 6**
**ASB8**	**ankyrin repeat and SOCS box containing 8**
**ASCC1**	**activating signal cointegrator 1 complex subunit 1**
**ASCC3**	**activating signal cointegrator 1 complex subunit 3**
**ASF1A**	**anti-silencing function 1A histone chaperone**
**ASH1L**	**ash1 (absent, small, or homeotic)-like (Drosophila)**
**ASH2L**	**ash2 (absent, small, or homeotic)-like (Drosophila)**
**ASMTL-AS1**	**ASMTL antisense RNA 1**
**ASNSD1**	**asparagine synthetase domain containing 1**
**ASPH**	**aspartate beta-hydroxylase**
**ASPSCR1**	**alveolar soft part sarcoma chromosome region, candidate 1**
**ASTL**	**astacin-like metallo-endopeptidase (M12 family)**

**ASUN**	**asunder spermatogenesis regulator**
**ASXL1**	**additional sex combs like transcriptional regulator 1**
**ASXL2**	**additional sex combs like transcriptional regulator 2**
**ATAD1**	**ATPase family, AAA domain containing 1**
**ATAD2**	**ATPase family, AAA domain containing 2**
**ATAD2B**	**ATPase family, AAA domain containing 2B**
**ATAD5**	**ATPase family, AAA domain containing 5**
**ATF1**	**activating transcription factor 1**
**ATF2**	**activating transcription factor 2**
**ATF3**	**activating transcription factor 3**
**ATF4**	**activating transcription factor 4**
**ATF5**	**activating transcription factor 5**
**ATF6**	**activating transcription factor 6**
**ATF7**	**activating transcription factor 7**
**ATF7IP**	**activating transcription factor 7 interacting protein**
**ATG101**	**autophagy related 101**
**ATG12**	**autophagy related 12**
**ATG16L1**	**autophagy related 16-like 1 (S. cerevisiae)**
**ATG16L2**	**autophagy related 16-like 2 (S. cerevisiae)**
**ATG3**	**autophagy related 3**
**ATG4C**	**autophagy related 4C, cysteine peptidase**
**ATG5**	**autophagy related 5**
**ATG9A**	**autophagy related 9A**
**ATIC**	**5-aminoimidazole-4-carboxamide ribonucleotide formyltransferase/IMP cyclohydrolase**
**ATL2**	**atlastin GTPase 2**
**ATL3**	**atlastin GTPase 3**
**ATM**	**ATM serine/threonine kinase**
**ATN1**	**atrophin 1**
**ATOX1**	**antioxidant 1 copper chaperone**
**ATP10D**	**ATPase, class V, type 10D**
**ATP13A1**	**ATPase type 13A1**
**ATP1A1-AS1**	**ATP1A1 antisense RNA 1**
**ATP1A3**	**ATPase, Na+/K+ transporting, alpha 3 polypeptide**
**ATP2A3**	**ATPase, Ca++ transporting, ubiquitous**
**ATP2C1**	**ATPase, Ca++ transporting, type 2C, member 1**
**ATP5B**	**ATP synthase, H+ transporting, mitochondrial F1 complex, beta polypeptide**
**ATP5E**	**ATP synthase, H+ transporting, mitochondrial F1 complex, epsilon subunit**
**ATP5G1**	**ATP synthase, H+ transporting, mitochondrial Fo complex, subunit C1 (subunit 9)**
**ATP5G2**	**ATP synthase, H+ transporting, mitochondrial Fo complex, subunit C2 (subunit 9)**
**ATP5G3**	**ATP synthase, H+ transporting, mitochondrial Fo complex, subunit C3 (subunit 9)**
**ATP5I**	**ATP synthase, H+ transporting, mitochondrial Fo complex, subunit E**

**ATP5J**	**ATP synthase, H+ transporting, mitochondrial Fo complex, subunit F6**
**ATP5L**	**ATP synthase, H+ transporting, mitochondrial Fo complex, subunit G**
**ATP5SL**	**ATP5S-like**
**ATP6V0A2**	**ATPase, H+ transporting, lysosomal V0 subunit a2**
**ATP6V0B**	**ATPase, H+ transporting, lysosomal 21kDa, V0 subunit b**
**ATP6V0D1**	**ATPase, H+ transporting, lysosomal 38kDa, V0 subunit d1**
**ATP6V1A**	**ATPase, H+ transporting, lysosomal 70kDa, V1 subunit A**
**ATP6V1C1**	**ATPase, H+ transporting, lysosomal 42kDa, V1 subunit C1**
**ATP6V1D**	**ATPase, H+ transporting, lysosomal 34kDa, V1 subunit D**
**ATP6V1G1**	**ATPase, H+ transporting, lysosomal 13kDa, V1 subunit G1**
**ATP6V1G2**	**ATPase, H+ transporting, lysosomal 13kDa, V1 subunit G2**
**ATP6V1H**	**ATPase, H+ transporting, lysosomal 50/57kDa, V1 subunit H**
**ATP8A1**	**ATPase, aminophospholipid transporter (APLT), class I, type 8A, member 1**
**ATP8B3**	**ATPase, aminophospholipid transporter, class I, type 8B, member 3**
**ATPAF2**	**ATP synthase mitochondrial F1 complex assembly factor 2**
**ATPIF1**	**ATPase inhibitory factor 1**
**ATR**	**ATR serine/threonine kinase**
**ATRAID**	**all-trans retinoic acid-induced differentiation factor**
**ATRIP**	**ATR interacting protein**
**ATXN1**	**ataxin 1**
**ATXN1L**	**ataxin 1-like**
**ATXN2**	**ataxin 2**
**ATXN2L**	**ataxin 2-like**
**ATXN7**	**ataxin 7**
**ATXN7L2**	**ataxin 7-like 2**
**ATXN7L3**	**ataxin 7-like 3**
**AUNIP**	**aurora kinase A and ninein interacting protein**
**AUP1**	**ancient ubiquitous protein 1**
**AURKAIP1**	**aurora kinase A interacting protein 1**
**AVPR2**	**arginine vasopressin receptor 2**
**AXDND1**	**axonemal dynein light chain domain containing 1**
**AZIN1**	**antizyme inhibitor 1**
**B2M**	**beta-2-microglobulin**
**B3GALNT2**	**beta-1,3-N-acetylgalactosaminyltransferase 2**
**B3GALT4**	**UDP-Gal:betaGlcNAc beta 1,3-galactosyltransferase, polypeptide 4**
**B3GALT6**	**UDP-Gal:betaGal beta 1,3-galactosyltransferase polypeptide 6**
**B3GAT3**	**beta-1,3-glucuronyltransferase 3**
**B3GNT1**	
**B3GNT7**	**UDP-GlcNAc:betaGal beta-1,3-N-acetylglucosaminyltransferase 7**
**B3GNT9**	**UDP-GlcNAc:betaGal beta-1,3-N-acetylglucosaminyltransferase 9**
**B4GALT1**	**UDP-Gal:betaGlcNAc beta 1,4- galactosyltransferase, polypeptide 1**

**B4GALT3**	**UDP-Gal:betaGlcNAc beta 1,4- galactosyltransferase, polypeptide 3**
**B4GALT4**	**UDP-Gal:betaGlcNAc beta 1,4- galactosyltransferase, polypeptide 4**
**B4GALT6**	**UDP-Gal:betaGlcNAc beta 1,4- galactosyltransferase, polypeptide 6**
**B4GALT7**	**xylosylprotein beta 1,4-galactosyltransferase, polypeptide 7**
**B9D1**	**B9 protein domain 1**
**B9D2**	**B9 protein domain 2**
**BAD**	**BCL2-associated agonist of cell death**
**BAG2**	**BCL2-associated athanogene 2**
**BAG3**	**BCL2-associated athanogene 3**
**BAG4**	**BCL2-associated athanogene 4**
**BAIAP2L1**	**BAI1-associated protein 2-like 1**
**BANF1**	**barrier to autointegration factor 1**
**BANK1**	**B-cell scaffold protein with ankyrin repeats 1**
**BANP**	**BTG3 associated nuclear protein**
**BARD1**	**BRCA1 associated RING domain 1**
**BASP1**	**brain abundant, membrane attached signal protein 1**
**BATF**	**basic leucine zipper transcription factor, ATF-like**
**BAX**	**BCL2-associated X protein**
**BAZ1A**	**bromodomain adjacent to zinc finger domain, 1A**
**BAZ1B**	**bromodomain adjacent to zinc finger domain, 1B**
**BAZ2A**	**bromodomain adjacent to zinc finger domain, 2A**
**BBC3**	**BCL2 binding component 3**
**BBIP1**	**BBSome interacting protein 1**
**BBS1**	**Bardet-Biedl syndrome 1**
**BBS10**	**Bardet-Biedl syndrome 10**
**BCAP29**	**B-cell receptor-associated protein 29**
**BCAS2**	**breast carcinoma amplified sequence 2**
**BCAS3**	**breast carcinoma amplified sequence 3**
**BCAT1**	**branched chain amino-acid transaminase 1, cytosolic**
**BCAT2**	**branched chain amino-acid transaminase 2, mitochondrial**
**BCCIP**	**BRCA2 and CDKN1A interacting protein**
**BCDIN3D**	**BCDIN3 domain containing**
**BCKDHA**	**branched chain keto acid dehydrogenase E1, alpha polypeptide**
**BCKDK**	**branched chain ketoacid dehydrogenase kinase**
**BCL10**	**B-cell CLL/lymphoma 10**
**BCL11A**	**B-cell CLL/lymphoma 11A (zinc finger protein)**
**BCL2**	**B-cell CLL/lymphoma 2**
**BCL2A1**	**BCL2-related protein A1**
**BCL2L1**	**BCL2-like 1**
**BCL2L12**	**BCL2-like 12 (proline rich)**
**BCL2L13**	**BCL2-like 13 (apoptosis facilitator)**

**BCL7C**	**B-cell CLL/lymphoma 7C**
**BCLAF1**	**BCL2-associated transcription factor 1**
**BCOR**	**BCL6 corepressor**
**BCRP3**	**breakpoint cluster region pseudogene 3**
**BCS1L**	**BC1 (ubiquinol-cytochrome c reductase) synthesis-like**
**BDNF-AS**	**BDNF antisense RNA**
**BECN1**	**beclin 1, autophagy related**
**BEND6**	**BEN domain containing 6**
**BEST4**	**bestrophin 4**
**BET1L**	**Bet1 golgi vesicular membrane trafficking protein-like**
**BFAR**	**bifunctional apoptosis regulator**
**BHLHE40**	**basic helix-loop-helix family, member e40**
**BICD2**	**bicaudal D homolog 2 (Drosophila)**
**BIN2**	**bridging integrator 2**
**BIRC2**	**baculoviral IAP repeat containing 2**
**BIRC3**	**baculoviral IAP repeat containing 3**
**BIRC6**	**baculoviral IAP repeat containing 6**
**BIVM**	**basic, immunoglobulin-like variable motif containing**
**BLCAP**	**bladder cancer associated protein**
**BLK**	**BLK proto-oncogene, Src family tyrosine kinase**
**BLM**	**Bloom syndrome, RecQ helicase-like**
**BLNK**	**B-cell linker**
**BLOC1S1**	**biogenesis of lysosomal organelles complex-1, subunit 1**
**BLOC1S1-RDH5**	**BLOC1S1-RDH5 readthrough**
**BLOC1S2**	**biogenesis of lysosomal organelles complex-1, subunit 2**
**BLOC1S5**	**biogenesis of lysosomal organelles complex-1, subunit 5, muted**
**BLOC1S5-TXNDC5**	**BLOC1S5-TXNDC5 readthrough (NMD candidate)**
**BLOC1S6**	**biogenesis of lysosomal organelles complex-1, subunit 6, pallidin**
**BLVRA**	**biliverdin reductase A**
**BLVRB**	**biliverdin reductase B**
**BMF**	**Bcl2 modifying factor**
**BMP2K**	**BMP2 inducible kinase**
**BMPR2**	**bone morphogenetic protein receptor, type II (serine/threonine kinase)**
**BMS1**	**BMS1 ribosome biogenesis factor**
**BMS1P4**	**BMS1 ribosome biogenesis factor pseudogene 4**
**BNIP1**	**BCL2/adenovirus E1B 19kDa interacting protein 1**
**BOD1L1**	**biorientation of chromosomes in cell division 1-like 1**
**BOLA3**	**bolA family member 3**
**BOLA3-AS1**	**BOLA3 antisense RNA 1 (head to head)**
**BOP1**	**block of proliferation 1**
**BORA**	**bora, aurora kinase A activator**

**BPNT1**	**3'(2'), 5'-bisphosphate nucleotidase 1**
**BPTF**	**bromodomain PHD finger transcription factor**
**BRAF**	**B-Raf proto-oncogene, serine/threonine kinase**
**BRAP**	**BRCA1 associated protein**
**BRCA1**	**breast cancer 1, early onset**
**BRCA2**	**breast cancer 2, early onset**
**BRD8**	**bromodomain containing 8**
**BRE**	**brain and reproductive organ-expressed (TNFRSF1A modulator)**
**BREA2**	**breast cancer estrogen-induced apoptosis 2**
**BRF2**	**BRF2, RNA polymerase III transcription initiation factor 50 kDa subunit**
**BRI3BP**	**BRI3 binding protein**
**BRICD5**	**BRICHOS domain containing 5**
**BRIP1**	**BRCA1 interacting protein C-terminal helicase 1**
**BRIX1**	**BRX1, biogenesis of ribosomes**
**BRK1**	**BRICK1, SCAR/WAVE actin-nucleating complex subunit**
**BROX**	**BRO1 domain and CAAX motif containing**
**BSCL2**	**Berardinelli-Seip congenital lipodystrophy 2 (seipin)**
**BSDC1**	**BSD domain containing 1**
**BSG**	**basigin (Ok blood group)**
**BSPRY**	**B-box and SPRY domain containing**
**BST2**	**bone marrow stromal cell antigen 2**
**BTAF1**	**BTAF1 RNA polymerase II, B-TFIID transcription factor-associated, 170kDa**
**BTBD19**	**BTB (POZ) domain containing 19**
**BTBD6**	**BTB (POZ) domain containing 6**
**BTD**	**biotinidase**
**BTF3**	**basic transcription factor 3**
**BTF3L4**	**basic transcription factor 3-like 4**
**BTG1**	**B-cell translocation gene 1, anti-proliferative**
**BTG2**	**BTG family, member 2**
**BTLA**	**B and T lymphocyte associated**
**BTRC**	**beta-transducin repeat containing E3 ubiquitin protein ligase**
**BUB1**	**BUB1 mitotic checkpoint serine/threonine kinase**
**BUD13**	**BUD13 homolog (S. cerevisiae)**
**BUD31**	**BUD31 homolog (S. cerevisiae)**
**BYSL**	**bystin-like**
**BZRAP1**	**benzodiazepine receptor (peripheral) associated protein 1**
**BZW1**	**basic leucine zipper and W2 domains 1**
**BZW2**	**basic leucine zipper and W2 domains 2**
**C10ORF10**	**chromosome 10 open reading frame 10**
**C10ORF111**	**chromosome 10 open reading frame 111**
**C10ORF131**	**chromosome 10 open reading frame 131**

**C10ORF76**	**chromosome 10 open reading frame 76**
**C10ORF95**	**chromosome 10 open reading frame 95**
**C11ORF1**	**chromosome 11 open reading frame 1**
**C11ORF24**	**chromosome 11 open reading frame 24**
**C11ORF30**	**chromosome 11 open reading frame 30**
**C11ORF31**	**chromosome 11 open reading frame 31**
**C11ORF48**	**chromosome 11 open reading frame 48**
**C11ORF54**	**chromosome 11 open reading frame 54**
**C11ORF58**	**chromosome 11 open reading frame 58**
**C11ORF65**	**chromosome 11 open reading frame 65**
**C11ORF68**	**chromosome 11 open reading frame 68**
**C11ORF71**	**chromosome 11 open reading frame 71**
**C11ORF73**	**chromosome 11 open reading frame 73**
**C11ORF91**	**chromosome 11 open reading frame 91**
**C11ORF94**	**chromosome 11 open reading frame 94**
**C12ORF10**	**chromosome 12 open reading frame 10**
**C12ORF29**	**chromosome 12 open reading frame 29**
**C12ORF4**	**chromosome 12 open reading frame 4**
**C12ORF57**	**chromosome 12 open reading frame 57**
**C12ORF65**	**chromosome 12 open reading frame 65**
**C12ORF73**	**chromosome 12 open reading frame 73**
**C12ORF77**	**chromosome 12 open reading frame 77**
**C14ORF1**	**chromosome 14 open reading frame 1**
**C14ORF119**	**chromosome 14 open reading frame 119**
**C14ORF159**	**chromosome 14 open reading frame 159**
**C14ORF169**	**chromosome 14 open reading frame 169**
**C14ORF2**	**chromosome 14 open reading frame 2**
**C14ORF28**	**chromosome 14 open reading frame 28**
**C14ORF80**	**chromosome 14 open reading frame 80**
**C15ORF39**	**chromosome 15 open reading frame 39**
**C15ORF40**	**chromosome 15 open reading frame 40**
**C15ORF62**	**chromosome 15 open reading frame 62**
**C16ORF13**	**chromosome 16 open reading frame 13**
**C16ORF46**	**chromosome 16 open reading frame 46**
**C16ORF54**	**chromosome 16 open reading frame 54**
**C16ORF62**	**chromosome 16 open reading frame 62**
**C16ORF70**	**chromosome 16 open reading frame 70**
**C16ORF72**	**chromosome 16 open reading frame 72**
**C16ORF86**	**chromosome 16 open reading frame 86**
**C16ORF87**	**chromosome 16 open reading frame 87**
**C16ORF95**	**chromosome 16 open reading frame 95**

**C17ORF100**	**chromosome 17 open reading frame 100**
**C17ORF49**	**chromosome 17 open reading frame 49**
**C17ORF53**	**chromosome 17 open reading frame 53**
**C17ORF62**	**chromosome 17 open reading frame 62**
**C17ORF70**	**chromosome 17 open reading frame 70**
**C17ORF75**	**chromosome 17 open reading frame 75**
**C17ORF80**	**chromosome 17 open reading frame 80**
**C17ORF85**	**chromosome 17 open reading frame 85**
**C17ORF89**	**chromosome 17 open reading frame 89**
**C17ORF99**	**chromosome 17 open reading frame 99**
**C18ORF21**	**chromosome 18 open reading frame 21**
**C18ORF25**	**chromosome 18 open reading frame 25**
**C18ORF32**	**chromosome 18 open reading frame 32**
**C18ORF8**	**chromosome 18 open reading frame 8**
**C19ORF24**	**chromosome 19 open reading frame 24**
**C19ORF25**	**chromosome 19 open reading frame 25**
**C19ORF43**	**chromosome 19 open reading frame 43**
**C19ORF47**	**chromosome 19 open reading frame 47**
**C19ORF48**	**chromosome 19 open reading frame 48**
**C19ORF52**	**chromosome 19 open reading frame 52**
**C19ORF53**	**chromosome 19 open reading frame 53**
**C19ORF54**	**chromosome 19 open reading frame 54**
**C19ORF60**	**chromosome 19 open reading frame 60**
**C19ORF66**	**chromosome 19 open reading frame 66**
**C19ORF70**	**chromosome 19 open reading frame 70**
**C19ORF71**	**chromosome 19 open reading frame 71**
**C19ORF73**	**chromosome 19 open reading frame 73**
**C1D**	**C1D nuclear receptor corepressor**
**C1GALT1C1**	**C1GALT1-specific chaperone 1**
**C1ORF101**	**chromosome 1 open reading frame 101**
**C1ORF112**	**chromosome 1 open reading frame 112**
**C1ORF123**	**chromosome 1 open reading frame 123**
**C1ORF131**	**chromosome 1 open reading frame 131**
**C1ORF186**	**chromosome 1 open reading frame 186**
**C1ORF216**	**chromosome 1 open reading frame 216**
**C1ORF27**	**chromosome 1 open reading frame 27**
**C1ORF35**	**chromosome 1 open reading frame 35**
**C1ORF43**	**chromosome 1 open reading frame 43**
**C1ORF50**	**chromosome 1 open reading frame 50**
**C1ORF52**	**chromosome 1 open reading frame 52**
**C1ORF56**	**chromosome 1 open reading frame 56**

C1ORF74	chromosome 1 open reading frame 74
C1QBP	complement component 1, q subcomponent binding protein
C1R	complement component 1, r subcomponent
C1RL	complement component 1, r subcomponent-like
C1RL-AS1	C1RL antisense RNA 1
C20ORF144	chromosome 20 open reading frame 144
C20ORF195	chromosome 20 open reading frame 195
C20ORF24	chromosome 20 open reading frame 24
C21ORF2	chromosome 21 open reading frame 2
C21ORF33	chromosome 21 open reading frame 33
C21ORF58	chromosome 21 open reading frame 58
C21ORF59	chromosome 21 open reading frame 59
C21ORF62-AS1	C21orf62 antisense RNA 1
C21ORF91	chromosome 21 open reading frame 91
C22ORF23	chromosome 22 open reading frame 23
C22ORF29	chromosome 22 open reading frame 29
C22ORF39	chromosome 22 open reading frame 39
C22ORF46	chromosome 22 open reading frame 46
C2CD2	C2 calcium-dependent domain containing 2
C2CD3	C2 calcium-dependent domain containing 3
C2CD5	C2 calcium-dependent domain containing 5
C2ORF15	chromosome 2 open reading frame 15
C2ORF42	chromosome 2 open reading frame 42
C2ORF47	chromosome 2 open reading frame 47
C2ORF54	chromosome 2 open reading frame 54
C2ORF61	chromosome 2 open reading frame 61
C2ORF68	chromosome 2 open reading frame 68
C2ORF69	chromosome 2 open reading frame 69
C2ORF70	chromosome 2 open reading frame 70
C2ORF74	chromosome 2 open reading frame 74
C2ORF76	chromosome 2 open reading frame 76
C3ORF17	chromosome 3 open reading frame 17
C3ORF18	chromosome 3 open reading frame 18
C3ORF22	chromosome 3 open reading frame 22
C3ORF38	chromosome 3 open reading frame 38
C3ORF58	chromosome 3 open reading frame 58
C3ORF62	chromosome 3 open reading frame 62
C4ORF29	chromosome 4 open reading frame 29
C4ORF46	chromosome 4 open reading frame 46
C5ORF15	chromosome 5 open reading frame 15
C5ORF22	chromosome 5 open reading frame 22

C5ORF24	chromosome 5 open reading frame 24
C5ORF28	chromosome 5 open reading frame 28
C5ORF30	chromosome 5 open reading frame 30
C5ORF34	chromosome 5 open reading frame 34
C5ORF45	chromosome 5 open reading frame 45
C5ORF51	chromosome 5 open reading frame 51
C5ORF63	chromosome 5 open reading frame 63
C6ORF1	chromosome 6 open reading frame 1
C6ORF120	chromosome 6 open reading frame 120
C6ORF226	chromosome 6 open reading frame 226
C6ORF48	chromosome 6 open reading frame 48
C6ORF52	chromosome 6 open reading frame 52
C6ORF62	chromosome 6 open reading frame 62
C7ORF13	chromosome 7 open reading frame 13
C7ORF25	chromosome 7 open reading frame 25
C7ORF26	chromosome 7 open reading frame 26
C7ORF43	chromosome 7 open reading frame 43
C7ORF49	chromosome 7 open reading frame 49
C7ORF50	chromosome 7 open reading frame 50
C7ORF55	chromosome 7 open reading frame 55
C7ORF71	chromosome 7 open reading frame 71
C7ORF73	chromosome 7 open reading frame 73
C8G	complement component 8, gamma polypeptide
C8ORF33	chromosome 8 open reading frame 33
C8ORF37	chromosome 8 open reading frame 37
C8ORF44	chromosome 8 open reading frame 44
C8ORF44-SGK3	C8orf44-SGK3 readthrough
C8ORF59	chromosome 8 open reading frame 59
C8ORF76	chromosome 8 open reading frame 76
C8ORF82	chromosome 8 open reading frame 82
C8ORF86	chromosome 8 open reading frame 86
C9ORF114	chromosome 9 open reading frame 114
C9ORF156	chromosome 9 open reading frame 156
C9ORF16	chromosome 9 open reading frame 16
C9ORF163	chromosome 9 open reading frame 163
C9ORF172	chromosome 9 open reading frame 172
C9ORF3	chromosome 9 open reading frame 3
C9ORF41	chromosome 9 open reading frame 41
C9ORF43	chromosome 9 open reading frame 43
C9ORF69	chromosome 9 open reading frame 69
C9ORF72	chromosome 9 open reading frame 72

**C9ORF85**	**chromosome 9 open reading frame 85**
**C9ORF89**	**chromosome 9 open reading frame 89**
**C9ORF91**	**chromosome 9 open reading frame 91**
**CA5BP1**	**carbonic anhydrase VB pseudogene 1**
**CAAP1**	**caspase activity and apoptosis inhibitor 1**
**CAB39**	**calcium binding protein 39**
**CAB39L**	**calcium binding protein 39-like**
**CABIN1**	**calcineurin binding protein 1**
**CABYR**	**calcium binding tyrosine-(Y)-phosphorylation regulated**
**CACNA1E**	**calcium channel, voltage-dependent, R type, alpha 1E subunit**
**CACNB3**	**calcium channel, voltage-dependent, beta 3 subunit**
**CACTIN**	**cactin, spliceosome C complex subunit**
**CACUL1**	**CDK2-associated, cullin domain 1**
**CACYBP**	**calcyclin binding protein**
**CAD**	**carbamoyl-phosphate synthetase 2, aspartate transcarbamylase, and dihydroorotase**
**CAGE1**	**cancer antigen 1**
**CAHM**	**colon adenocarcinoma hypermethylated (non-protein coding)**
**CALCOCO1**	**calcium binding and coiled-coil domain 1**
**CALCOCO2**	**calcium binding and coiled-coil domain 2**
**CALHM2**	**calcium homeostasis modulator 2**
**CALM1**	**calmodulin 1 (phosphorylase kinase, delta)**
**CALM2**	**calmodulin 2 (phosphorylase kinase, delta)**
**CALM3**	**calmodulin 3 (phosphorylase kinase, delta)**
**CALN1**	**calneuron 1**
**CALR**	**calreticulin**
**CAMK1**	**calcium/calmodulin-dependent protein kinase I**
**CAMK2D**	**calcium/calmodulin-dependent protein kinase II delta**
**CAMP**	**cathelicidin antimicrobial peptide**
**CAMSAP2**	**calmodulin regulated spectrin-associated protein family, member 2**
**CAMTA2**	**calmodulin binding transcription activator 2**
**CAND1**	**cullin-associated and neddylation-dissociated 1**
**CANX**	**calnexin**
**CAP1**	**CAP, adenylate cyclase-associated protein 1 (yeast)**
**CAPN10**	**calpain 10**
**CAPN15**	**calpain 15**
**CAPNS1**	**calpain, small subunit 1**
**CAPRIN1**	**cell cycle associated protein 1**
**CAPS2**	**calcyphosine 2**
**CAPZA1**	**capping protein (actin filament) muscle Z-line, alpha 1**
**CARD11**	**caspase recruitment domain family, member 11**
**CARD8**	**caspase recruitment domain family, member 8**

**CARM1**	**coactivator-associated arginine methyltransferase 1**
**CASC1**	**cancer susceptibility candidate 1**
**CASC3**	**cancer susceptibility candidate 3**
**CASC5**	**cancer susceptibility candidate 5**
**CASD1**	**CAS1 domain containing 1**
**CASP10**	**caspase 10, apoptosis-related cysteine peptidase**
**CASP3**	**caspase 3, apoptosis-related cysteine peptidase**
**CASP6**	**caspase 6, apoptosis-related cysteine peptidase**
**CATSPERD**	**catsper channel auxiliary subunit delta**
**CBFA2T3**	**core-binding factor, runt domain, alpha subunit 2; translocated to, 3**
**CBFB**	**core-binding factor, beta subunit**
**CBLB**	**Cbl proto-oncogene B, E3 ubiquitin protein ligase**
**CBLL1**	**Cbl proto-oncogene-like 1, E3 ubiquitin protein ligase**
**CBLN3**	**cerebellin 3 precursor**
**CBR4**	**carbonyl reductase 4**
**CBX1**	**chromobox homolog 1**
**CBX3**	**chromobox homolog 3**
**CBX3P2**	**chromobox homolog 3 pseudogene 2**
**CBX5**	**chromobox homolog 5**
**CBX6**	**chromobox homolog 6**
**CBX7**	**chromobox homolog 7**
**CBY3**	**chibby homolog 3 (Drosophila)**
**CC2D1B**	**coiled-coil and C2 domain containing 1B**
**CCAR1**	**cell division cycle and apoptosis regulator 1**
**CCAR2**	**cell cycle and apoptosis regulator 2**
**CCBL2**	**cysteine conjugate-beta lyase 2**
**CCDC101**	**coiled-coil domain containing 101**
**CCDC102A**	**coiled-coil domain containing 102A**
**CCDC102B**	**coiled-coil domain containing 102B**
**CCDC103**	**coiled-coil domain containing 103**
**CCDC107**	**coiled-coil domain containing 107**
**CCDC109B**	**coiled-coil domain containing 109B**
**CCDC110**	**coiled-coil domain containing 110**
**CCDC113**	**coiled-coil domain containing 113**
**CCDC115**	**coiled-coil domain containing 115**
**CCDC12**	**coiled-coil domain containing 12**
**CCDC121**	**coiled-coil domain containing 121**
**CCDC124**	**coiled-coil domain containing 124**
**CCDC13**	**coiled-coil domain containing 13**
**CCDC130**	**coiled-coil domain containing 130**
**CCDC134**	**coiled-coil domain containing 134**

**CCDC137**	**coiled-coil domain containing 137**
**CCDC138**	**coiled-coil domain containing 138**
**CCDC142**	**coiled-coil domain containing 142**
**CCDC144B**	**coiled-coil domain containing 144B (pseudogene)**
**CCDC146**	**coiled-coil domain containing 146**
**CCDC151**	**coiled-coil domain containing 151**
**CCDC157**	**coiled-coil domain containing 157**
**CCDC171**	**coiled-coil domain containing 171**
**CCDC174**	**coiled-coil domain containing 174**
**CCDC18**	**coiled-coil domain containing 18**
**CCDC24**	**coiled-coil domain containing 24**
**CCDC25**	**coiled-coil domain containing 25**
**CCDC28A**	**coiled-coil domain containing 28A**
**CCDC28B**	**coiled-coil domain containing 28B**
**CCDC43**	**coiled-coil domain containing 43**
**CCDC47**	**coiled-coil domain containing 47**
**CCDC50**	**coiled-coil domain containing 50**
**CCDC51**	**coiled-coil domain containing 51**
**CCDC53**	**coiled-coil domain containing 53**
**CCDC57**	**coiled-coil domain containing 57**
**CCDC58**	**coiled-coil domain containing 58**
**CCDC59**	**coiled-coil domain containing 59**
**CCDC6**	**coiled-coil domain containing 6**
**CCDC62**	**coiled-coil domain containing 62**
**CCDC66**	**coiled-coil domain containing 66**
**CCDC69**	**coiled-coil domain containing 69**
**CCDC7**	**coiled-coil domain containing 7**
**CCDC77**	**coiled-coil domain containing 77**
**CCDC78**	**coiled-coil domain containing 78**
**CCDC85B**	**coiled-coil domain containing 85B**
**CCDC86**	**coiled-coil domain containing 86**
**CCDC88A**	**coiled-coil domain containing 88A**
**CCDC88B**	**coiled-coil domain containing 88B**
**CCDC88C**	**coiled-coil domain containing 88C**
**CCDC9**	**coiled-coil domain containing 9**
**CCDC90B**	**coiled-coil domain containing 90B**
**CCDC94**	**coiled-coil domain containing 94**
**CCDC97**	**coiled-coil domain containing 97**
**CCL15**	**chemokine (C-C motif) ligand 15**
**CCL22**	**chemokine (C-C motif) ligand 22**
**CCL3**	**chemokine (C-C motif) ligand 3**

**CCL5**	**chemokine (C-C motif) ligand 5**
**CCM2**	**cerebral cavernous malformation 2**
**CCNB1IP1**	**cyclin B1 interacting protein 1, E3 ubiquitin protein ligase**
**CCNC**	**cyclin C**
**CCND2**	**cyclin D2**
**CCND3**	**cyclin D3**
**CCNDBP1**	**cyclin D-type binding-protein 1**
**CCNG2**	**cyclin G2**
**CCNH**	**cyclin H**
**CCNI**	**cyclin I**
**CCNJ**	**cyclin J**
**CCNL1**	**cyclin L1**
**CCNL2**	**cyclin L2**
**CCNT1**	**cyclin T1**
**CCNT2**	**cyclin T2**
**CCNYL1**	**cyclin Y-like 1**
**CCR1**	**chemokine (C-C motif) receptor 1**
**CCR5**	**chemokine (C-C motif) receptor 5 (gene/pseudogene)**
**CCR6**	**chemokine (C-C motif) receptor 6**
**CCR7**	**chemokine (C-C motif) receptor 7**
**CCR8**	**chemokine (C-C motif) receptor 8**
**CCR9**	**chemokine (C-C motif) receptor 9**
**CCRN4L**	**CCR4 carbon catabolite repression 4-like (S. cerevisiae)**
**CCT2**	**chaperonin containing TCP1, subunit 2 (beta)**
**CCT3**	**chaperonin containing TCP1, subunit 3 (gamma)**
**CCT4**	**chaperonin containing TCP1, subunit 4 (delta)**
**CCT5**	**chaperonin containing TCP1, subunit 5 (epsilon)**
**CCT6A**	**chaperonin containing TCP1, subunit 6A (zeta 1)**
**CCT6B**	**chaperonin containing TCP1, subunit 6B (zeta 2)**
**CCT7**	**chaperonin containing TCP1, subunit 7 (eta)**
**CCT8**	**chaperonin containing TCP1, subunit 8 (theta)**
**CD101**	**CD101 molecule**
**CD164**	**CD164 molecule, sialomucin**
**CD200R1**	**CD200 receptor 1**
**CD22**	**CD22 molecule**
**CD226**	**CD226 molecule**
**CD27**	**CD27 molecule**
**CD274**	**CD274 molecule**
**CD28**	**CD28 molecule**
**CD2AP**	**CD2-associated protein**
**CD2BP2**	**CD2 (cytoplasmic tail) binding protein 2**

CD300A	CD300a molecule
CD300C	CD300c molecule
CD320	CD320 molecule
CD37	CD37 molecule
CD38	CD38 molecule
CD40	CD40 molecule, TNF receptor superfamily member 5
CD46	CD46 molecule, complement regulatory protein
CD47	CD47 molecule
CD48	CD48 molecule
CD53	CD53 molecule
CD55	CD55 molecule, decay accelerating factor for complement (Cromer blood group)
CD58	CD58 molecule
CD59	CD59 molecule, complement regulatory protein
CD5L	CD5 molecule-like
CD63	CD63 molecule
CD68	CD68 molecule
CD69	CD69 molecule
CD70	CD70 molecule
CD72	CD72 molecule
CD74	CD74 molecule, major histocompatibility complex, class II invariant chain
CD79A	CD79a molecule, immunoglobulin-associated alpha
CD80	CD80 molecule
CD83	CD83 molecule
CDC123	cell division cycle 123
CDC14A	cell division cycle 14A
CDC20	cell division cycle 20
CDC26	cell division cycle 26
CDC27	cell division cycle 27
CDC37	cell division cycle 37
CDC37L1	cell division cycle 37-like 1
CDC42	cell division cycle 42
CDC42BPG	CDC42 binding protein kinase gamma (DMPK-like)
CDC42EP3	CDC42 effector protein (Rho GTPase binding) 3
CDC42SE1	CDC42 small effector 1
CDC42SE2	CDC42 small effector 2
CDC5L	cell division cycle 5-like
CDC6	cell division cycle 6
CDC7	cell division cycle 7
CDC73	cell division cycle 73
CDCA2	cell division cycle associated 2
CDCA3	cell division cycle associated 3

**CDCA4**	**cell division cycle associated 4**
**CDCA5**	**cell division cycle associated 5**
**CDH17**	**cadherin 17, LI cadherin (liver-intestine)**
**CDH24**	**cadherin 24, type 2**
**CDIP1**	**cell death-inducing p53 target 1**
**CDIPT**	**CDP-diacylglycerol--inositol 3-phosphatidyltransferase**
**CDK10**	**cyclin-dependent kinase 10**
**CDK11A**	**cyclin-dependent kinase 11A**
**CDK11B**	**cyclin-dependent kinase 11B**
**CDK12**	**cyclin-dependent kinase 12**
**CDK13**	**cyclin-dependent kinase 13**
**CDK14**	**cyclin-dependent kinase 14**
**CDK16**	**cyclin-dependent kinase 16**
**CDK17**	**cyclin-dependent kinase 17**
**CDK19**	**cyclin-dependent kinase 19**
**CDK2AP1**	**cyclin-dependent kinase 2 associated protein 1**
**CDK4**	**cyclin-dependent kinase 4**
**CDK5RAP1**	**CDK5 regulatory subunit associated protein 1**
**CDK5RAP3**	**CDK5 regulatory subunit associated protein 3**
**CDK6**	**cyclin-dependent kinase 6**
**CDK7**	**cyclin-dependent kinase 7**
**CDK9**	**cyclin-dependent kinase 9**
**CDKAL1**	**CDK5 regulatory subunit associated protein 1-like 1**
**CDKL3**	**cyclin-dependent kinase-like 3**
**CDKN1B**	**cyclin-dependent kinase inhibitor 1B (p27, Kip1)**
**CDKN2AIP**	**CDKN2A interacting protein**
**CDKN2AIPNL**	**CDKN2A interacting protein N-terminal like**
**CDKN2C**	**cyclin-dependent kinase inhibitor 2C (p18, inhibits CDK4)**
**CDR2**	**cerebellar degeneration-related protein 2, 62kDa**
**CDS2**	**CDP-diacylglycerol synthase (phosphatidate cytidylyltransferase) 2**
**CDV3**	**CDV3 homolog (mouse)**
**CEBPZ**	**CCAAT/enhancer binding protein (C/EBP), zeta**
**CELF1**	**CUGBP, Elav-like family member 1**
**CELSR3**	**cadherin, EGF LAG seven-pass G-type receptor 3**
**CEMP1**	**cementum protein 1**
**CEND1**	**cell cycle exit and neuronal differentiation 1**
**CENPA**	**centromere protein A**
**CENPBD1**	**CENPB DNA-binding domains containing 1**
**CENPH**	**centromere protein H**
**CENPJ**	**centromere protein J**
**CENPL**	**centromere protein L**

**CENPN**	**centromere protein N**
**CENPO**	**centromere protein O**
**CENPP**	**centromere protein P**
**CENPQ**	**centromere protein Q**
**CENPT**	**centromere protein T**
**CENPU**	**centromere protein U**
**CENPW**	**centromere protein W**
**CEP120**	**centrosomal protein 120kDa**
**CEP135**	**centrosomal protein 135kDa**
**CEP170**	**centrosomal protein 170kDa**
**CEP19**	**centrosomal protein 19kDa**
**CEP192**	**centrosomal protein 192kDa**
**CEP250**	**centrosomal protein 250kDa**
**CEP350**	**centrosomal protein 350kDa**
**CEP44**	**centrosomal protein 44kDa**
**CEP57**	**centrosomal protein 57kDa**
**CEP57L1**	**centrosomal protein 57kDa-like 1**
**CEP63**	**centrosomal protein 63kDa**
**CEP68**	**centrosomal protein 68kDa**
**CEP72**	**centrosomal protein 72kDa**
**CEP76**	**centrosomal protein 76kDa**
**CEP83**	**centrosomal protein 83kDa**
**CEP95**	**centrosomal protein 95kDa**
**CEPT1**	**choline/ethanolamine phosphotransferase 1**
**CERCAM**	**cerebral endothelial cell adhesion molecule**
**CERS6**	**ceramide synthase 6**
**CES2**	**carboxylesterase 2**
**CETN3**	**centrin, EF-hand protein, 3**
**CFAP36**	**cilia and flagella associated protein 36**
**CFAP43**	**cilia and flagella associated protein 43**
**CFAP53**	**cilia and flagella associated protein 53**
**CFAP57**	**cilia and flagella associated protein 57**
**CFAP69**	**cilia and flagella associated protein 69**
**CFAP70**	**cilia and flagella associated protein 70**
**CFAP97**	**cilia and flagella associated protein 97**
**CFDP1**	**craniofacial development protein 1**
**CFL1**	**cofilin 1 (non-muscle)**
**CFL1P1**	**cofilin 1 (non-muscle) pseudogene 1**
**CFL2**	**cofilin 2 (muscle)**
**CFLAR**	**CASP8 and FADD-like apoptosis regulator**
**CFLAR-AS1**	**CFLAR antisense RNA 1**

**CFP**	**complement factor properdin**
**CGGBP1**	**CGG triplet repeat binding protein 1**
**CGREF1**	**cell growth regulator with EF-hand domain 1**
**CHAC2**	**ChaC, cation transport regulator homolog 2 (E. coli)**
**CHAF1A**	**chromatin assembly factor 1, subunit A (p150)**
**CHAMP1**	**chromosome alignment maintaining phosphoprotein 1**
**CHCHD1**	**coiled-coil-helix-coiled-coil-helix domain containing 1**
**CHCHD2**	**coiled-coil-helix-coiled-coil-helix domain containing 2**
**CHCHD4**	**coiled-coil-helix-coiled-coil-helix domain containing 4**
**CHCHD5**	**coiled-coil-helix-coiled-coil-helix domain containing 5**
**CHCHD6**	**coiled-coil-helix-coiled-coil-helix domain containing 6**
**CHCHD7**	**coiled-coil-helix-coiled-coil-helix domain containing 7**
**CHD1**	**chromodomain helicase DNA binding protein 1**
**CHD1L**	**chromodomain helicase DNA binding protein 1-like**
**CHD2**	**chromodomain helicase DNA binding protein 2**
**CHD8**	**chromodomain helicase DNA binding protein 8**
**CHD9**	**chromodomain helicase DNA binding protein 9**
**CHEK1**	**checkpoint kinase 1**
**CHERP**	**calcium homeostasis endoplasmic reticulum protein**
**CHMP1A**	**charged multivesicular body protein 1A**
**CHMP1B**	**charged multivesicular body protein 1B**
**CHMP2A**	**charged multivesicular body protein 2A**
**CHMP3**	**charged multivesicular body protein 3**
**CHMP4A**	**charged multivesicular body protein 4A**
**CHMP6**	**charged multivesicular body protein 6**
**CHORDC1**	**cysteine and histidine-rich domain (CHORD) containing 1**
**CHP1**	**calcineurin-like EF-hand protein 1**
**CHPF2**	**chondroitin polymerizing factor 2**
**CHRAC1**	**chromatin accessibility complex 1**
**CHRNA6**	**cholinergic receptor, nicotinic, alpha 6 (neuronal)**
**CHST11**	**carbohydrate (chondroitin 4) sulfotransferase 11**
**CHST12**	**carbohydrate (chondroitin 4) sulfotransferase 12**
**CHST14**	**carbohydrate (N-acetylgalactosamine 4-0) sulfotransferase 14**
**CHST2**	**carbohydrate (N-acetylglucosamine-6-O) sulfotransferase 2**
**CHTF8**	**CTF8, chromosome transmission fidelity factor 8 homolog (S. cerevisiae)**
**CHTOP**	**chromatin target of PRMT1**
**CHUK**	**conserved helix-loop-helix ubiquitous kinase**
**CHURC1**	**churchill domain containing 1**
**CHURC1-FNTB**	**CHURC1-FNTB readthrough**
**CIAO1**	**cytosolic iron-sulfur assembly component 1**
**CIAPIN1**	**cytokine induced apoptosis inhibitor 1**

**CIC**	**capicua transcriptional repressor**
**CIDEB**	**cell death-inducing DFFA-like effector b**
**CIDECP**	**cell death-inducing DFFA-like effector c pseudogene**
**CIITA**	**class II, major histocompatibility complex, transactivator**
**CIPC**	**CLOCK-interacting pacemaker**
**CIRBP**	**cold inducible RNA binding protein**
**CIRBP-AS1**	**CIRBP antisense RNA 1**
**CIRH1A**	**cirrhosis, autosomal recessive 1A (cirhin)**
**CISD1**	**CDGSH iron sulfur domain 1**
**CISD2**	**CDGSH iron sulfur domain 2**
**CISD3**	**CDGSH iron sulfur domain 3**
**CITED2**	**Cbp/p300-interacting transactivator, with Glu/Asp-rich carboxy-terminal domain, 2**
**CKAP2**	**cytoskeleton associated protein 2**
**CKLF**	**chemokine-like factor**
**CKLF-CMTM1**	**CKLF-CMTM1 readthrough**
**CKS1B**	**CDC28 protein kinase regulatory subunit 1B**
**CKS2**	**CDC28 protein kinase regulatory subunit 2**
**CLASP1**	**cytoplasmic linker associated protein 1**
**CLASP2**	**cytoplasmic linker associated protein 2**
**CLASRP**	**CLK4-associating serine/arginine rich protein**
**CLCC1**	**chloride channel CLIC-like 1**
**CLCN2**	**chloride channel, voltage-sensitive 2**
**CLCN3**	**chloride channel, voltage-sensitive 3**
**CLDN1**	**claudin 1**
**CLDN12**	**claudin 12**
**CLDND1**	**claudin domain containing 1**
**CLDND2**	**claudin domain containing 2**
**CLEC16A**	**C-type lectin domain family 16, member A**
**CLEC17A**	**C-type lectin domain family 17, member A**
**CLEC2D**	**C-type lectin domain family 2, member D**
**CLEC7A**	**C-type lectin domain family 7, member A**
**CLECL1**	**C-type lectin-like 1**
**CLHC1**	**clathrin heavy chain linker domain containing 1**
**CLIC4**	**chloride intracellular channel 4**
**CLIP2**	**CAP-GLY domain containing linker protein 2**
**CLIP4**	**CAP-GLY domain containing linker protein family, member 4**
**CLK1**	**CDC-like kinase 1**
**CLK2**	**CDC-like kinase 2**
**CLN3**	**ceroid-lipofuscinosis, neuronal 3**
**CLN5**	**ceroid-lipofuscinosis, neuronal 5**
**CLNS1A**	**chloride channel, nucleotide-sensitive, 1A**

**CLOCK**	**clock circadian regulator**
**CLP1**	**cleavage and polyadenylation factor I subunit 1**
**CLPB**	**ClpB homolog, mitochondrial AAA ATPase chaperonin**
**CLPTM1**	**cleft lip and palate associated transmembrane protein 1**
**CLSTN3**	**calsyntenin 3**
**CLTC**	**clathrin, heavy chain (Hc)**
**CLUAP1**	**clusterin associated protein 1**
**CLUH**	**clustered mitochondria (cluA/CLU1) homolog**
**CMAHP**	**cytidine monophospho-N-acetylneuraminic acid hydroxylase, pseudogene**
**CMC1**	**C-x(9)-C motif containing 1**
**CMC2**	**C-x(9)-C motif containing 2**
**CMPK1**	**cytidine monophosphate (UMP-CMP) kinase 1, cytosolic**
**CMTR1**	**cap methyltransferase 1**
**CNBP**	**CCHC-type zinc finger, nucleic acid binding protein**
**CNIH1**	**cornichon family AMPA receptor auxiliary protein 1**
**CNIH2**	**cornichon family AMPA receptor auxiliary protein 2**
**CNIH4**	**cornichon family AMPA receptor auxiliary protein 4**
**CNN2**	**calponin 2**
**CNNM2**	**cyclin and CBS domain divalent metal cation transport mediator 2**
**CNOT1**	**CCR4-NOT transcription complex, subunit 1**
**CNOT10**	**CCR4-NOT transcription complex, subunit 10**
**CNOT4**	**CCR4-NOT transcription complex, subunit 4**
**CNOT6**	**CCR4-NOT transcription complex, subunit 6**
**CNOT7**	**CCR4-NOT transcription complex, subunit 7**
**CNOT8**	**CCR4-NOT transcription complex, subunit 8**
**CNP**	**2',3'-cyclic nucleotide 3' phosphodiesterase**
**CNPPD1**	**cyclin Pas1/PHO80 domain containing 1**
**CNPY2**	**canopy FGF signaling regulator 2**
**CNPY3**	**canopy FGF signaling regulator 3**
**CNPY4**	**canopy FGF signaling regulator 4**
**CNR1**	**cannabinoid receptor 1 (brain)**
**CNR2**	**cannabinoid receptor 2 (macrophage)**
**CNST**	**consortin, connexin sorting protein**
**CNTD2**	**cyclin N-terminal domain containing 2**
**COA1**	**cytochrome c oxidase assembly factor 1 homolog (S. cerevisiae)**
**COA4**	**cytochrome c oxidase assembly factor 4 homolog (S. cerevisiae)**
**COA5**	**cytochrome c oxidase assembly factor 5**
**COA6**	**cytochrome c oxidase assembly factor 6**
**COASY**	**CoA synthase**
**COG2**	**component of oligomeric golgi complex 2**
**COG3**	**component of oligomeric golgi complex 3**

**COG4**	**component of oligomeric golgi complex 4**
**COG8**	**component of oligomeric golgi complex 8**
**COIL**	**coilin**
**COL19A1**	**collagen, type XIX, alpha 1**
**COL20A1**	**collagen, type XX, alpha 1**
**COL5A3**	**collagen, type V, alpha 3**
**COL9A2**	**collagen, type IX, alpha 2**
**COL9A3**	**collagen, type IX, alpha 3**
**COLEC11**	**collectin sub-family member 11**
**COLGALT1**	**collagen beta(1-O)galactosyltransferase 1**
**COMMD10**	**COMM domain containing 10**
**COMMD2**	**COMM domain containing 2**
**COMMD3**	**COMM domain containing 3**
**COMMD3-BMI1**	**COMMD3-BMI1 readthrough**
**COMMD4**	**COMM domain containing 4**
**COMMD5**	**COMM domain containing 5**
**COMMD6**	**COMM domain containing 6**
**COMTD1**	**catechol-O-methyltransferase domain containing 1**
**COPA**	**coatomer protein complex, subunit alpha**
**COPB1**	**coatomer protein complex, subunit beta 1**
**COPE**	**coatomer protein complex, subunit epsilon**
**COPG1**	**coatomer protein complex, subunit gamma 1**
**COPS2**	**COP9 signalosome subunit 2**
**COPS3**	**COP9 signalosome subunit 3**
**COPS4**	**COP9 signalosome subunit 4**
**COPS5**	**COP9 signalosome subunit 5**
**COPS6**	**COP9 signalosome subunit 6**
**COPS7A**	**COP9 signalosome subunit 7A**
**COPS7B**	**COP9 signalosome subunit 7B**
**COPS8**	**COP9 signalosome subunit 8**
**COPZ1**	**coatomer protein complex, subunit zeta 1**
**COQ10A**	**coenzyme Q10 homolog A (S. cerevisiae)**
**COQ10B**	**coenzyme Q10 homolog B (S. cerevisiae)**
**COQ3**	**coenzyme Q3 methyltransferase**
**COQ4**	**coenzyme Q4**
**COQ6**	**coenzyme Q6 monooxygenase**
**COQ9**	**coenzyme Q9**
**CORIN**	**corin, serine peptidase**
**CORO1A**	**coronin, actin binding protein, 1A**
**CORO1B**	**coronin, actin binding protein, 1B**
**CORO1C**	**coronin, actin binding protein, 1C**

**CORO7**	**coronin 7**
**CORO7-PAM16**	**CORO7-PAM16 readthrough**
**COX11**	**COX11 cytochrome c oxidase copper chaperone**
**COX14**	**COX14 cytochrome c oxidase assembly factor**
**COX15**	**cytochrome c oxidase assembly homolog 15 (yeast)**
**COX16**	**COX16 cytochrome c oxidase assembly homolog (S. cerevisiae)**
**COX17**	**COX17 cytochrome c oxidase copper chaperone**
**COX18**	**COX18 cytochrome c oxidase assembly factor**
**COX19**	**COX19 cytochrome c oxidase assembly factor**
**COX20**	**COX20 cytochrome c oxidase assembly factor**
**COX5A**	**cytochrome c oxidase subunit Va**
**COX5B**	**cytochrome c oxidase subunit Vb**
**COX6C**	**cytochrome c oxidase subunit VIc**
**COX7A2**	**cytochrome c oxidase subunit VIIa polypeptide 2 (liver)**
**COX7A2L**	**cytochrome c oxidase subunit VIIa polypeptide 2 like**
**COX7B**	**cytochrome c oxidase subunit VIIb**
**COX7C**	**cytochrome c oxidase subunit VIIc**
**COX8A**	**cytochrome c oxidase subunit VIIIA (ubiquitous)**
**CPEB2**	**cytoplasmic polyadenylation element binding protein 2**
**CPEB4**	**cytoplasmic polyadenylation element binding protein 4**
**CPNE1**	**copine I**
**CPNE6**	**copine VI (neuronal)**
**CPNE8**	**copine VIII**
**CPOX**	**coproporphyrinogen oxidase**
**CPPED1**	**calcineurin-like phosphoesterase domain containing 1**
**CPSF1**	**cleavage and polyadenylation specific factor 1, 160kDa**
**CPSF2**	**cleavage and polyadenylation specific factor 2, 100kDa**
**CPSF3**	**cleavage and polyadenylation specific factor 3, 73kDa**
**CPSF7**	**cleavage and polyadenylation specific factor 7, 59kDa**
**CPT1C**	**carnitine palmitoyltransferase 1C**
**CR2**	**complement component (3d/Epstein Barr virus) receptor 2**
**CRABP2**	**cellular retinoic acid binding protein 2**
**CRACR2A**	**calcium release activated channel regulator 2A**
**CRADD**	**CASP2 and RIPK1 domain containing adaptor with death domain**
**CRAT**	**carnitine O-acetyltransferase**
**CRBN**	**cereblon**
**CRCP**	**CGRP receptor component**
**CREB1**	**cAMP responsive element binding protein 1**
**CREB3**	**cAMP responsive element binding protein 3**
**CREB3L2**	**cAMP responsive element binding protein 3-like 2**
**CREB3L4**	**cAMP responsive element binding protein 3-like 4**

**CREBL2**	**cAMP responsive element binding protein-like 2**
**CREBRF**	**CREB3 regulatory factor**
**CREBZF**	**CREB/ATF bZIP transcription factor**
**CRELD1**	**cysteine-rich with EGF-like domains 1**
**CREM**	**cAMP responsive element modulator**
**CRK**	**v-crk avian sarcoma virus CT10 oncogene homolog**
**CRLF2**	**cytokine receptor-like factor 2**
**CRLF3**	**cytokine receptor-like factor 3**
**CRLS1**	**cardiolipin synthase 1**
**CRMP1**	**collapsin response mediator protein 1**
**CRTC1**	**CREB regulated transcription coactivator 1**
**CRTC2**	**CREB regulated transcription coactivator 2**
**CRYZ**	**crystallin, zeta (quinone reductase)**
**CS**	**citrate synthase**
**CSAD**	**cysteine sulfinic acid decarboxylase**
**CSDE1**	**cold shock domain containing E1, RNA-binding**
**CSE1L**	**CSE1 chromosome segregation 1-like (yeast)**
**CSK**	**c-src tyrosine kinase**
**CSNK1A1**	**casein kinase 1, alpha 1**
**CSNK1D**	**casein kinase 1, delta**
**CSNK1G1**	**casein kinase 1, gamma 1**
**CSNK1G2**	**casein kinase 1, gamma 2**
**CSNK1G3**	**casein kinase 1, gamma 3**
**CSPP1**	**centrosome and spindle pole associated protein 1**
**CSRNP1**	**cysteine-serine-rich nuclear protein 1**
**CSRNP2**	**cysteine-serine-rich nuclear protein 2**
**CSTF2T**	**cleavage stimulation factor, 3' pre-RNA, subunit 2, 64kDa, tau variant**
**CSTF3**	**cleavage stimulation factor, 3' pre-RNA, subunit 3, 77kDa**
**CTAGE5**	**CTAGE family, member 5**
**CTBS**	**chitobiase, di-N-acetyl-**
**CTC1**	**CTS telomere maintenance complex component 1**
**CTCF**	**CCCTC-binding factor (zinc finger protein)**
**CTDNEP1**	**CTD nuclear envelope phosphatase 1**
**CTDP1**	**CTD (carboxy-terminal domain, RNA polymerase II, polypeptide A) phosphatase, subunit 1**
**CTDSP2**	**CTD (carboxy-terminal domain, RNA polymerase II, polypeptide A) small phosphatase 2**
**CTDSPL2**	**CTD (carboxy-terminal domain, RNA polymerase II, polypeptide A) small phosphatase like 2**
**CTF1**	**cardiotrophin 1**
**CTH**	**cystathionine gamma-lyase**
**CTNNAL1**	**catenin (cadherin-associated protein), alpha-like 1**

**CTNNB1**	**catenin (cadherin-associated protein), beta 1, 88kDa**
**CTNNBL1**	**catenin, beta like 1**
**CTNS**	**cystinosin, lysosomal cystine transporter**
**CTR9**	**CTR9, Paf1/RNA polymerase II complex component**
**CTRB1**	**chymotrypsinogen B1**
**CTRL**	**chymotrypsin-like**
**CTSA**	**cathepsin A**
**CTSC**	**cathepsin C**
**CTSO**	**cathepsin O**
**CTSZ**	**cathepsin Z**
**CTTN**	**cortactin**
**CUEDC1**	**CUE domain containing 1**
**CUEDC2**	**CUE domain containing 2**
**CUL2**	**cullin 2**
**CUL4A**	**cullin 4A**
**CUL5**	**cullin 5**
**CUL7**	**cullin 7**
**CUL9**	**cullin 9**
**CUTA**	**cutA divalent cation tolerance homolog (E. coli)**
**CUTC**	**cutC copper transporter**
**CWC25**	**CWC25 spliceosome-associated protein homolog (S. cerevisiae)**
**CWF19L1**	**CWF19-like 1, cell cycle control (S. pombe)**
**CWF19L2**	**CWF19-like 2, cell cycle control (S. pombe)**
**CXCR3**	**chemokine (C-X-C motif) receptor 3**
**CXCR4**	**chemokine (C-X-C motif) receptor 4**
**CXCR5**	**chemokine (C-X-C motif) receptor 5**
**CXORF21**	**chromosome X open reading frame 21**
**CXORF38**	**chromosome X open reading frame 38**
**CXORF40A**	**chromosome X open reading frame 40A**
**CXORF40B**	**chromosome X open reading frame 40B**
**CXORF58**	**chromosome X open reading frame 58**
**CXORF65**	**chromosome X open reading frame 65**
**CXXC1**	**CXXC finger protein 1**
**CXXC4**	**CXXC finger protein 4**
**CYB561**	**cytochrome b561**
**CYB561A3**	**cytochrome b561 family, member A3**
**CYB561D1**	**cytochrome b561 family, member D1**
**CYB561D2**	**cytochrome b561 family, member D2**
**CYB5B**	**cytochrome b5 type B (outer mitochondrial membrane)**
**CYB5D1**	**cytochrome b5 domain containing 1**
**CYB5D2**	**cytochrome b5 domain containing 2**

CYB5R2	cytochrome b5 reductase 2
CYB5R4	cytochrome b5 reductase 4
CYBA	cytochrome b-245, alpha polypeptide
CYC1	cytochrome c-1
CYCS	cytochrome c, somatic
CYP1B1	cytochrome P450, family 1, subfamily B, polypeptide 1
CYP20A1	cytochrome P450, family 20, subfamily A, polypeptide 1
CYP2R1	cytochrome P450, family 2, subfamily R, polypeptide 1
CYP4V2	cytochrome P450, family 4, subfamily V, polypeptide 2
CYP51A1	cytochrome P450, family 51, subfamily A, polypeptide 1
CYS1	cystin 1
CYSRT1	cysteine-rich tail protein 1
CYSTM1	cysteine-rich transmembrane module containing 1
CYTH1	cytohesin 1
CYTH2	cytohesin 2
CYTIP	cytohesin 1 interacting protein
D2HGDH	D-2-hydroxyglutarate dehydrogenase
DAD1	defender against cell death 1
DAGLB	diacylglycerol lipase, beta
DALRD3	DALR anticodon binding domain containing 3
DANCR	differentiation antagonizing non-protein coding RNA
DAP3	death associated protein 3
DAPK1	death-associated protein kinase 1
DAPK3	death-associated protein kinase 3
DAPP1	dual adaptor of phosphotyrosine and 3-phosphoinositides
DARS	aspartyl-tRNA synthetase
DARS2	aspartyl-tRNA synthetase 2, mitochondrial
DAZAP1	DAZ associated protein 1
DAZAP2	DAZ associated protein 2
DBF4	DBF4 zinc finger
DBI	diazepam binding inhibitor (GABA receptor modulator, acyl-CoA binding protein)
DBNL	drebrin-like
DBP	D site of albumin promoter (albumin D-box) binding protein
DBT	dihydrolipoamide branched chain transacylase E2
DCAF10	DDB1 and CUL4 associated factor 10
DCAF11	DDB1 and CUL4 associated factor 11
DCAF13	DDB1 and CUL4 associated factor 13
DCAF16	DDB1 and CUL4 associated factor 16
DCAF17	DDB1 and CUL4 associated factor 17
DCAF6	DDB1 and CUL4 associated factor 6
DCAF7	DDB1 and CUL4 associated factor 7

**DCAF8**	**DDB1 and CUL4 associated factor 8**
**DCAKD**	**dephospho-CoA kinase domain containing**
**DCDC1**	**doublecortin domain containing 1**
**DCHS1**	**dachsous cadherin-related 1**
**DCK**	**deoxycytidine kinase**
**DCLRE1A**	**DNA cross-link repair 1A**
**DCLRE1B**	**DNA cross-link repair 1B**
**DCLRE1C**	**DNA cross-link repair 1C**
**DCP1A**	**decapping mRNA 1A**
**DCPS**	**decapping enzyme, scavenger**
**DCTD**	**dCMP deaminase**
**DCTN1**	**dynactin 1**
**DCTN4**	**dynactin 4 (p62)**
**DCTN5**	**dynactin 5 (p25)**
**DCTPP1**	**dCTP pyrophosphatase 1**
**DCUN1D2**	**DCN1, defective in cullin neddylation 1, domain containing 2**
**DCUN1D3**	**DCN1, defective in cullin neddylation 1, domain containing 3**
**DCUN1D5**	**DCN1, defective in cullin neddylation 1, domain containing 5**
**DCXR**	**dicarbonyl/L-xylulose reductase**
**DDAH2**	**dimethylarginine dimethylaminohydrolase 2**
**DDB2**	**damage-specific DNA binding protein 2, 48kDa**
**DDHD1**	**DDHD domain containing 1**
**DDHD2**	**DDHD domain containing 2**
**DDIAS**	**DNA damage-induced apoptosis suppressor**
**DDIT3**	**DNA-damage-inducible transcript 3**
**DDIT4**	**DNA-damage-inducible transcript 4**
**DDN**	**dendrin**
**DDOST**	**dolichyl-diphosphooligosaccharide--protein glycosyltransferase subunit (non-catalytic)**
**DDR2**	**discoidin domain receptor tyrosine kinase 2**
**DDRGK1**	**DDRGK domain containing 1**
**DDX1**	**DEAD (Asp-Glu-Ala-Asp) box helicase 1**
**DDX11L2**	**DEAD/H (Asp-Glu-Ala-Asp/His) box helicase 11 like 2**
**DDX18**	**DEAD (Asp-Glu-Ala-Asp) box polypeptide 18**
**DDX19A**	**DEAD (Asp-Glu-Ala-Asp) box polypeptide 19A**
**DDX20**	**DEAD (Asp-Glu-Ala-Asp) box polypeptide 20**
**DDX21**	**DEAD (Asp-Glu-Ala-Asp) box helicase 21**
**DDX25**	**DEAD (Asp-Glu-Ala-Asp) box helicase 25**
**DDX27**	**DEAD (Asp-Glu-Ala-Asp) box polypeptide 27**
**DDX28**	**DEAD (Asp-Glu-Ala-Asp) box polypeptide 28**
**DDX31**	**DEAD (Asp-Glu-Ala-Asp) box polypeptide 31**
**DDX39A**	**DEAD (Asp-Glu-Ala-Asp) box polypeptide 39A**

**DDX39B**	**DEAD (Asp-Glu-Ala-Asp) box polypeptide 39B**
**DDX3X**	**DEAD (Asp-Glu-Ala-Asp) box helicase 3, X-linked**
**DDX41**	**DEAD (Asp-Glu-Ala-Asp) box polypeptide 41**
**DDX42**	**DEAD (Asp-Glu-Ala-Asp) box helicase 42**
**DDX46**	**DEAD (Asp-Glu-Ala-Asp) box polypeptide 46**
**DDX47**	**DEAD (Asp-Glu-Ala-Asp) box polypeptide 47**
**DDX49**	**DEAD (Asp-Glu-Ala-Asp) box polypeptide 49**
**DDX5**	**DEAD (Asp-Glu-Ala-Asp) box helicase 5**
**DDX50**	**DEAD (Asp-Glu-Ala-Asp) box polypeptide 50**
**DDX51**	**DEAD (Asp-Glu-Ala-Asp) box polypeptide 51**
**DDX54**	**DEAD (Asp-Glu-Ala-Asp) box polypeptide 54**
**DDX55**	**DEAD (Asp-Glu-Ala-Asp) box polypeptide 55**
**DDX56**	**DEAD (Asp-Glu-Ala-Asp) box helicase 56**
**DDX59**	**DEAD (Asp-Glu-Ala-Asp) box polypeptide 59**
**DDX6**	**DEAD (Asp-Glu-Ala-Asp) box helicase 6**
**DEAF1**	**DEAF1 transcription factor**
**DECR1**	**2,4-dienoyl CoA reductase 1, mitochondrial**
**DECR2**	**2,4-dienoyl CoA reductase 2, peroxisomal**
**DEF6**	**differentially expressed in FDCP 6 homolog (mouse)**
**DEGS1**	**delta(4)-desaturase, sphingolipid 1**
**DEK**	**DEK proto-oncogene**
**DENND1C**	**DENN/MADD domain containing 1C**
**DENND2D**	**DENN/MADD domain containing 2D**
**DENND3**	**DENN/MADD domain containing 3**
**DENND4A**	**DENN/MADD domain containing 4A**
**DENND4B**	**DENN/MADD domain containing 4B**
**DENND5A**	**DENN/MADD domain containing 5A**
**DENND6A**	**DENN/MADD domain containing 6A**
**DENR**	**density-regulated protein**
**DEPDC5**	**DEP domain containing 5**
**DERL1**	**derlin 1**
**DERL2**	**derlin 2**
**DERL3**	**derlin 3**
**DESI1**	**desumoylating isopeptidase 1**
**DFFA**	**DNA fragmentation factor, 45kDa, alpha polypeptide**
**DGAT1**	**diacylglycerol O-acyltransferase 1**
**DGKA**	**diacylglycerol kinase, alpha 80kDa**
**DGKH**	**diacylglycerol kinase, eta**
**DGUOK**	**deoxyguanosine kinase**
**DHDDS**	**dehydrodolichyl diphosphate synthase**
**DHFR**	**dihydrofolate reductase**

DHFRL1	dihydrofolate reductase-like 1
DHODH	dihydroorotate dehydrogenase (quinone)
DHPS	deoxyhypusine synthase
DHRS1	dehydrogenase/reductase (SDR family) member 1
DHRS11	dehydrogenase/reductase (SDR family) member 11
DHRS13	dehydrogenase/reductase (SDR family) member 13
DHRS7	dehydrogenase/reductase (SDR family) member 7
DHRS7C	dehydrogenase/reductase (SDR family) member 7C
DHTKD1	dehydrogenase E1 and transketolase domain containing 1
DHX15	DEAH (Asp-Glu-Ala-His) box helicase 15
DHX29	DEAH (Asp-Glu-Ala-His) box polypeptide 29
DHX33	DEAH (Asp-Glu-Ala-His) box polypeptide 33
DHX8	DEAH (Asp-Glu-Ala-His) box polypeptide 8
DHX9	DEAH (Asp-Glu-Ala-His) box helicase 9
DIABLO	diablo, IAP-binding mitochondrial protein
DIAPH1	diaphanous-related formin 1
DIAPH3	diaphanous-related formin 3
DIEXF	digestive organ expansion factor homolog (zebrafish)
DIMT1	DIM1 dimethyladenosine transferase 1 homolog (S. cerevisiae)
DIO3OS	DIO3 opposite strand/antisense RNA (head to head)
DIP2A	DIP2 disco-interacting protein 2 homolog A (Drosophila)
DIRC2	disrupted in renal carcinoma 2
DIS3	DIS3 exosome endoribonuclease and 3'-5' exoribonuclease
DIS3L	DIS3 like exosome 3'-5' exoribonuclease
DIS3L2	DIS3 like 3'-5' exoribonuclease 2
DKFZP434I0714	uncharacterized protein DKFZP434I0714
DKFZP434K028	uncharacterized LOC26070
DLAT	dihydrolipoamide S-acetyltransferase
DLD	dihydrolipoamide dehydrogenase
DLEU1	deleted in lymphocytic leukemia 1 (non-protein coding)
DLG1	discs, large homolog 1 (Drosophila)
DLG2	discs, large homolog 2 (Drosophila)
DLG4	discs, large homolog 4 (Drosophila)
DLST	dihydrolipoamide S-succinyltransferase (E2 component of 2-oxo-glutarate complex)
DMAP1	DNA methyltransferase 1 associated protein 1
DMPK	dystrophia myotonica-protein kinase
DMTF1	cyclin D binding myb-like transcription factor 1
DMWD	dystrophia myotonica, WD repeat containing
DMXL1	Dmx-like 1
DNA2	DNA replication helicase/nuclease 2
DNAAF2	dynein, axonemal, assembly factor 2

**DNAH10**	**dynein, axonemal, heavy chain 10**
**DNAJA1**	**DnaJ (Hsp40) homolog, subfamily A, member 1**
**DNAJA2**	**DnaJ (Hsp40) homolog, subfamily A, member 2**
**DNAJA3**	**DnaJ (Hsp40) homolog, subfamily A, member 3**
**DNAJB1**	**DnaJ (Hsp40) homolog, subfamily B, member 1**
**DNAJB12**	**DnaJ (Hsp40) homolog, subfamily B, member 12**
**DNAJB14**	**DnaJ (Hsp40) homolog, subfamily B, member 14**
**DNAJB2**	**DnaJ (Hsp40) homolog, subfamily B, member 2**
**DNAJB4**	**DnaJ (Hsp40) homolog, subfamily B, member 4**
**DNAJB9**	**DnaJ (Hsp40) homolog, subfamily B, member 9**
**DNAJC10**	**DnaJ (Hsp40) homolog, subfamily C, member 10**
**DNAJC11**	**DnaJ (Hsp40) homolog, subfamily C, member 11**
**DNAJC14**	**DnaJ (Hsp40) homolog, subfamily C, member 14**
**DNAJC15**	**DnaJ (Hsp40) homolog, subfamily C, member 15**
**DNAJC18**	**DnaJ (Hsp40) homolog, subfamily C, member 18**
**DNAJC2**	**DnaJ (Hsp40) homolog, subfamily C, member 2**
**DNAJC24**	**DnaJ (Hsp40) homolog, subfamily C, member 24**
**DNAJC25**	**DnaJ (Hsp40) homolog, subfamily C , member 25**
**DNAJC25-GNG10**	**DNAJC25-GNG10 readthrough**
**DNAJC27**	**DnaJ (Hsp40) homolog, subfamily C, member 27**
**DNAJC27-AS1**	**DNAJC27 antisense RNA 1**
**DNAJC30**	**DnaJ (Hsp40) homolog, subfamily C, member 30**
**DNAJC4**	**DnaJ (Hsp40) homolog, subfamily C, member 4**
**DNAJC7**	**DnaJ (Hsp40) homolog, subfamily C, member 7**
**DNAJC8**	**DnaJ (Hsp40) homolog, subfamily C, member 8**
**DNAJC9**	**DnaJ (Hsp40) homolog, subfamily C, member 9**
**DNAJC9-AS1**	**DNAJC9 antisense RNA 1**
**DNASE1L2**	**deoxyribonuclease I-like 2**
**DNASE1L3**	**deoxyribonuclease I-like 3**
**DNASE2**	**deoxyribonuclease II, lysosomal**
**DND1**	**DND microRNA-mediated repression inhibitor 1**
**DNLZ**	**DNL-type zinc finger**
**DNM2**	**dynamin 2**
**DNMT1**	**DNA (cytosine-5-)-methyltransferase 1**
**DNMT3A**	**DNA (cytosine-5-)-methyltransferase 3 alpha**
**DNTTIP1**	**deoxynucleotidyltransferase, terminal, interacting protein 1**
**DNTTIP2**	**deoxynucleotidyltransferase, terminal, interacting protein 2**
**DOC2GP**	**double C2-like domains, gamma, pseudogene**
**DOCK10**	**dedicator of cytokinesis 10**
**DOCK11**	**dedicator of cytokinesis 11**
**DOHH**	**deoxyhypusine hydroxylase/monooxygenase**

**DOK1**	**docking protein 1, 62kDa (downstream of tyrosine kinase 1)**
**DOK2**	**docking protein 2, 56kDa**
**DOK3**	**docking protein 3**
**DOLK**	**dolichol kinase**
**DONSON**	**downstream neighbor of SON**
**DOPEY1**	**dopey family member 1**
**DOT1L**	**DOT1-like histone H3K79 methyltransferase**
**DPAGT1**	**dolichyl-phosphate (UDP-N-acetylglucosamine) N-acetylglucosaminephosphotransferase 1 (GlcNAc-1-P transferase)**
**DPF2**	**D4, zinc and double PHD fingers family 2**
**DPH1**	**diphthamide biosynthesis 1**
**DPH3**	**diphthamide biosynthesis 3**
**DPH5**	**diphthamide biosynthesis 5**
**DPH6**	**diphthamine biosynthesis 6**
**DPH7**	**diphthamide biosynthesis 7**
**DPM2**	**dolichyl-phosphate mannosyltransferase polypeptide 2, regulatory subunit**
**DPM3**	**dolichyl-phosphate mannosyltransferase polypeptide 3**
**DPP3**	**dipeptidyl-peptidase 3**
**DPP8**	**dipeptidyl-peptidase 8**
**DPP9**	**dipeptidyl-peptidase 9**
**DPY19L3**	**dpy-19-like 3 (C. elegans)**
**DPY19L4**	**dpy-19-like 4 (C. elegans)**
**DQX1**	**DEAQ box RNA-dependent ATPase 1**
**DR1**	**down-regulator of transcription 1, TBP-binding (negative cofactor 2)**
**DRAM1**	**DNA-damage regulated autophagy modulator 1**
**DRAM2**	**DNA-damage regulated autophagy modulator 2**
**DRAP1**	**DR1-associated protein 1 (negative cofactor 2 alpha)**
**DRG1**	**developmentally regulated GTP binding protein 1**
**DRG2**	**developmentally regulated GTP binding protein 2**
**DROSHA**	**drosha, ribonuclease type III**
**DSCC1**	**DNA replication and sister chromatid cohesion 1**
**DSCR3**	**Down syndrome critical region 3**
**DSE**	**dermatan sulfate epimerase**
**DSTYK**	**dual serine/threonine and tyrosine protein kinase**
**DTD1**	**D-tyrosyl-tRNA deacylase 1**
**DTL**	**denticleless E3 ubiquitin protein ligase homolog (Drosophila)**
**DTWD1**	**DTW domain containing 1**
**DTX3L**	**deltex 3 like, E3 ubiquitin ligase**
**DTX4**	**deltex 4, E3 ubiquitin ligase**
**DUS1L**	**dihydrouridine synthase 1-like (S. cerevisiae)**
**DUS2**	**dihydrouridine synthase 2**
**DUS3L**	**dihydrouridine synthase 3-like (S. cerevisiae)**

**DUSP1**	**dual specificity phosphatase 1**
**DUSP10**	**dual specificity phosphatase 10**
**DUSP12**	**dual specificity phosphatase 12**
**DUSP16**	**dual specificity phosphatase 16**
**DUSP2**	**dual specificity phosphatase 2**
**DUSP22**	**dual specificity phosphatase 22**
**DUSP28**	**dual specificity phosphatase 28**
**DUSP5**	**dual specificity phosphatase 5**
**DUSP7**	**dual specificity phosphatase 7**
**DUT**	**deoxyuridine triphosphatase**
**DVL2**	**dishevelled segment polarity protein 2**
**DXO**	**decapping exoribonuclease**
**DYNC1LI1**	**dynein, cytoplasmic 1, light intermediate chain 1**
**DYNC1LI2**	**dynein, cytoplasmic 1, light intermediate chain 2**
**DYNC2H1**	**dynein, cytoplasmic 2, heavy chain 1**
**DYNLL1**	**dynein, light chain, LC8-type 1**
**DYNLRB1**	**dynein, light chain, roadblock-type 1**
**DYNLRB2**	**dynein, light chain, roadblock-type 2**
**DYRK1B**	**dual-specificity tyrosine-(Y)-phosphorylation regulated kinase 1B**
**DYRK3**	**dual-specificity tyrosine-(Y)-phosphorylation regulated kinase 3**
**DZIP3**	**DAZ interacting zinc finger protein 3**
**E2F1**	**E2F transcription factor 1**
**E2F3**	**E2F transcription factor 3**
**E2F4**	**E2F transcription factor 4, p107/p130-binding**
**E2F5**	**E2F transcription factor 5, p130-binding**
**E2F6**	**E2F transcription factor 6**
**E2F7**	**E2F transcription factor 7**
**E4F1**	**E4F transcription factor 1**
**EAF1**	**ELL associated factor 1**
**EAF2**	**ELL associated factor 2**
**EAPP**	**E2F-associated phosphoprotein**
**EARS2**	**glutamyl-tRNA synthetase 2, mitochondrial**
**EBAG9**	**estrogen receptor binding site associated, antigen, 9**
**EBI3**	**Epstein-Barr virus induced 3**
**EBNA1BP2**	**EBNA1 binding protein 2**
**EBP**	**emopamil binding protein (sterol isomerase)**
**ECD**	**ecdysoneless homolog (Drosophila)**
**ECE1**	**endothelin converting enzyme 1**
**ECE2**	**endothelin converting enzyme 2**
**ECH1**	**enoyl CoA hydratase 1, peroxisomal**
**ECHDC1**	**ethylmalonyl-CoA decarboxylase 1**

**ECI1**	**enoyl-CoA delta isomerase 1**
**ECSIT**	**ECSIT signalling integrator**
**EDC4**	**enhancer of mRNA decapping 4**
**EDEM1**	**ER degradation enhancer, mannosidase alpha-like 1**
**EDEM3**	**ER degradation enhancer, mannosidase alpha-like 3**
**EDRF1**	**erythroid differentiation regulatory factor 1**
**EEA1**	**early endosome antigen 1**
**EED**	**embryonic ectoderm development**
**EEF1A1**	**eukaryotic translation elongation factor 1 alpha 1**
**EEF1B2**	**eukaryotic translation elongation factor 1 beta 2**
**EEF1D**	**eukaryotic translation elongation factor 1 delta (guanine nucleotide exchange protein)**
**EEF1G**	**eukaryotic translation elongation factor 1 gamma**
**EEF2**	**eukaryotic translation elongation factor 2**
**EEF2K**	**eukaryotic elongation factor 2 kinase**
**EEFSEC**	**eukaryotic elongation factor, selenocysteine-tRNA-specific**
**EFCAB12**	**EF-hand calcium binding domain 12**
**EFCAB13**	**EF-hand calcium binding domain 13**
**EFCAB2**	**EF-hand calcium binding domain 2**
**EFCAB7**	**EF-hand calcium binding domain 7**
**EFNA4**	**ephrin-A4**
**EFNB2**	**ephrin-B2**
**EFTUD2**	**elongation factor Tu GTP binding domain containing 2**
**EGFEM1P**	**EGF-like and EMI domain containing 1, pseudogene**
**EGLN2**	**egl-9 family hypoxia-inducible factor 2**
**EGOT**	**eosinophil granule ontogeny transcript (non-protein coding)**
**EGR1**	**early growth response 1**
**EGR2**	**early growth response 2**
**EGR3**	**early growth response 3**
**EHBP1L1**	**EH domain binding protein 1-like 1**
**EHD1**	**EH-domain containing 1**
**EHD4**	**EH-domain containing 4**
**EHMT1**	**euchromatic histone-lysine N-methyltransferase 1**
**EID1**	**EP300 interacting inhibitor of differentiation 1**
**EID2B**	**EP300 interacting inhibitor of differentiation 2B**
**EIF1**	**eukaryotic translation initiation factor 1**
**EIF1AD**	**eukaryotic translation initiation factor 1A domain containing**
**EIF1B**	**eukaryotic translation initiation factor 1B**
**EIF2A**	**eukaryotic translation initiation factor 2A, 65kDa**
**EIF2AK3**	**eukaryotic translation initiation factor 2-alpha kinase 3**
**EIF2B1**	**eukaryotic translation initiation factor 2B, subunit 1 alpha, 26kDa**
**EIF2B3**	**eukaryotic translation initiation factor 2B, subunit 3 gamma, 58kDa**

EIF2B4	eukaryotic translation initiation factor 2B, subunit 4 delta, 67kDa
EIF2B5	eukaryotic translation initiation factor 2B, subunit 5 epsilon, 82kDa
EIF2D	eukaryotic translation initiation factor 2D
EIF2S1	eukaryotic translation initiation factor 2, subunit 1 alpha, 35kDa
EIF2S2	eukaryotic translation initiation factor 2, subunit 2 beta, 38kDa
EIF2S3	eukaryotic translation initiation factor 2, subunit 3 gamma, 52kDa
EIF3A	eukaryotic translation initiation factor 3, subunit A
EIF3D	eukaryotic translation initiation factor 3, subunit D
EIF3E	eukaryotic translation initiation factor 3, subunit E
EIF3F	eukaryotic translation initiation factor 3, subunit F
EIF3G	eukaryotic translation initiation factor 3, subunit G
EIF3H	eukaryotic translation initiation factor 3, subunit H
EIF3J	eukaryotic translation initiation factor 3, subunit J
EIF3K	eukaryotic translation initiation factor 3, subunit K
EIF3M	eukaryotic translation initiation factor 3, subunit M
EIF4A1	eukaryotic translation initiation factor 4A1
EIF4A2	eukaryotic translation initiation factor 4A2
EIF4A3	eukaryotic translation initiation factor 4A3
EIF4B	eukaryotic translation initiation factor 4B
EIF4E	eukaryotic translation initiation factor 4E
EIF4E2	eukaryotic translation initiation factor 4E family member 2
EIF4EBP3	eukaryotic translation initiation factor 4E binding protein 3
EIF4ENIF1	eukaryotic translation initiation factor 4E nuclear import factor 1
EIF4G2	eukaryotic translation initiation factor 4 gamma, 2
EIF4H	eukaryotic translation initiation factor 4H
EIF5	eukaryotic translation initiation factor 5
EIF5A	eukaryotic translation initiation factor 5A
EIF5B	eukaryotic translation initiation factor 5B
ELAVL1	ELAV like RNA binding protein 1
ELAVL3	ELAV like neuron-specific RNA binding protein 3
ELF1	E74-like factor 1 (ets domain transcription factor)
ELF4	E74-like factor 4 (ets domain transcription factor)
ELK1	ELK1, member of ETS oncogene family
ELK3	ELK3, ETS-domain protein (SRF accessory protein 2)
ELK4	ELK4, ETS-domain protein (SRF accessory protein 1)
ELL2	elongation factor, RNA polymerase II, 2
ELL3	elongation factor RNA polymerase II-like 3
ELMOD3	ELMO/CED-12 domain containing 3
ELMSAN1	ELM2 and Myb/SANT-like domain containing 1
ELOF1	elongation factor 1 homolog (S. cerevisiae)
ELOVL1	ELOVL fatty acid elongase 1

ELOVL5	ELOVL fatty acid elongase 5
ELP2	elongator acetyltransferase complex subunit 2
ELP3	elongator acetyltransferase complex subunit 3
ELP4	elongator acetyltransferase complex subunit 4
ELP5	elongator acetyltransferase complex subunit 5
ELP6	elongator acetyltransferase complex subunit 6
EMC1	ER membrane protein complex subunit 1
EMC3	ER membrane protein complex subunit 3
EMC4	ER membrane protein complex subunit 4
EMC6	ER membrane protein complex subunit 6
EMC7	ER membrane protein complex subunit 7
EMC9	ER membrane protein complex subunit 9
EME1	essential meiotic structure-specific endonuclease 1
EME2	essential meiotic structure-specific endonuclease subunit 2
EMG1	EMG1 N1-specific pseudouridine methyltransferase
EMP1	epithelial membrane protein 1
ENC1	ectodermal-neural cortex 1 (with BTB domain)
ENDOG	endonuclease G
ENKD1	enkurin domain containing 1
ENKUR	enkurin, TRPC channel interacting protein
ENO1	enolase 1, (alpha)
ENO1-AS1	ENO1 antisense RNA 1
ENO2	enolase 2 (gamma, neuronal)
ENOPH1	enolase-phosphatase 1
ENOSF1	enolase superfamily member 1
ENSA	endosulfine alpha
ENTHD2	ENTH domain containing 2
ENY2	enhancer of yellow 2 homolog (Drosophila)
EOGT	EGF domain-specific O-linked N-acetylglucosamine (GlcNAc) transferase
EP300	E1A binding protein p300
EP400	E1A binding protein p400
EP400NL	EP400 N-terminal like
EPB41L2	erythrocyte membrane protein band 4.1-like 2
EPB41L4A-AS1	EPB41L4A antisense RNA 1
EPC1	enhancer of polycomb homolog 1 (Drosophila)
EPC2	enhancer of polycomb homolog 2 (Drosophila)
EPHX3	epoxide hydrolase 3
EPM2A	epilepsy, progressive myoclonus type 2A, Lafora disease (laforin)
EPM2AIP1	EPM2A (laforin) interacting protein 1
EPN1	epsin 1
EPRS	glutamyl-prolyl-tRNA synthetase

**EPS15**	**epidermal growth factor receptor pathway substrate 15**
**EPSTI1**	**epithelial stromal interaction 1 (breast)**
**EPT1**	**ethanolaminephosphotransferase 1**
**ERAL1**	**Era-like 12S mitochondrial rRNA chaperone 1**
**ERAP1**	**endoplasmic reticulum aminopeptidase 1**
**ERBB2IP**	**erbb2 interacting protein**
**ERCC1**	**excision repair cross-complementation group 1**
**ERCC3**	**excision repair cross-complementation group 3**
**ERCC5**	**excision repair cross-complementation group 5**
**ERCC6**	**excision repair cross-complementation group 6**
**ERCC6L2**	**excision repair cross-complementation group 6-like 2**
**ERH**	**enhancer of rudimentary homolog (Drosophila)**
**ERI2**	**ERI1 exoribonuclease family member 2**
**ERLEC1**	**endoplasmic reticulum lectin 1**
**ERLIN1**	**ER lipid raft associated 1**
**ERLIN2**	**ER lipid raft associated 2**
**ERMARD**	**ER membrane-associated RNA degradation**
**ERMN**	**ermin, ERM-like protein**
**ERMP1**	**endoplasmic reticulum metallopeptidase 1**
**ERN1**	**endoplasmic reticulum to nucleus signaling 1**
**ERP27**	**endoplasmic reticulum protein 27**
**ERP29**	**endoplasmic reticulum protein 29**
**ESAM**	**endothelial cell adhesion molecule**
**ESCO1**	**establishment of sister chromatid cohesion N-acetyltransferase 1**
**ESCO2**	**establishment of sister chromatid cohesion N-acetyltransferase 2**
**ESD**	**esterase D**
**ESF1**	**ESF1, nucleolar pre-rRNA processing protein, homolog (S. cerevisiae)**
**ESPL1**	**extra spindle pole bodies homolog 1 (S. cerevisiae)**
**ESR2**	**estrogen receptor 2 (ER beta)**
**ESYT1**	**extended synaptotagmin-like protein 1**
**ESYT2**	**extended synaptotagmin-like protein 2**
**ETF1**	**eukaryotic translation termination factor 1**
**ETFA**	**electron-transfer-flavoprotein, alpha polypeptide**
**ETFB**	**electron-transfer-flavoprotein, beta polypeptide**
**ETFDH**	**electron-transferring-flavoprotein dehydrogenase**
**ETV2**	**ets variant 2**
**ETV3**	**ets variant 3**
**ETV5**	**ets variant 5**
**ETV6**	**ets variant 6**
**EVC**	**Ellis van Creveld protein**
**EVI2A**	**ecotropic viral integration site 2A**

**EVL**	**Enah/Vasp-like**
**EWSR1**	**EWS RNA-binding protein 1**
**EXD1**	**exonuclease 3'-5' domain containing 1**
**EXD3**	**exonuclease 3'-5' domain containing 3**
**EXO1**	**exonuclease 1**
**EXOC2**	**exocyst complex component 2**
**EXOC3L1**	**exocyst complex component 3-like 1**
**EXOC8**	**exocyst complex component 8**
**EXOG**	**endo/exonuclease (5'-3'), endonuclease G-like**
**EXOSC1**	**exosome component 1**
**EXOSC3**	**exosome component 3**
**EXOSC4**	**exosome component 4**
**EXOSC5**	**exosome component 5**
**EXOSC6**	**exosome component 6**
**EXOSC7**	**exosome component 7**
**EXOSC8**	**exosome component 8**
**EXOSC9**	**exosome component 9**
**EXT1**	**exostosin glycosyltransferase 1**
**EYA3**	**EYA transcriptional coactivator and phosphatase 3**
**EZH1**	**enhancer of zeste 1 polycomb repressive complex 2 subunit**
**EZH2**	**enhancer of zeste 2 polycomb repressive complex 2 subunit**
**EZR**	**ezrin**
**F10**	**coagulation factor X**
**F11R**	**F11 receptor**
**F2RL3**	**coagulation factor II (thrombin) receptor-like 3**
**FABP5P3**	**fatty acid binding protein 5 pseudogene 3**
**FADD**	**Fas (TNFRSF6)-associated via death domain**
**FADS1**	**fatty acid desaturase 1**
**FADS2**	**fatty acid desaturase 2**
**FAF1**	**Fas (TNFRSF6) associated factor 1**
**FAF2**	**Fas associated factor family member 2**
**FAHD1**	**fumarylacetoacetate hydrolase domain containing 1**
**FAM102B**	**family with sequence similarity 102, member B**
**FAM104A**	**family with sequence similarity 104, member A**
**FAM109A**	**family with sequence similarity 109, member A**
**FAM111A**	**family with sequence similarity 111, member A**
**FAM111B**	**family with sequence similarity 111, member B**
**FAM114A2**	**family with sequence similarity 114, member A2**
**FAM118B**	**family with sequence similarity 118, member B**
**FAM120A**	**family with sequence similarity 120A**
**FAM120AOS**	**family with sequence similarity 120A opposite strand**

**FAM122A**	**family with sequence similarity 122A**
**FAM122C**	**family with sequence similarity 122C**
**FAM126B**	**family with sequence similarity 126, member B**
**FAM129A**	**family with sequence similarity 129, member A**
**FAM129C**	**family with sequence similarity 129, member C**
**FAM131A**	**family with sequence similarity 131, member A**
**FAM131C**	**family with sequence similarity 131, member C**
**FAM133B**	**family with sequence similarity 133, member B**
**FAM134A**	**family with sequence similarity 134, member A**
**FAM134C**	**family with sequence similarity 134, member C**
**FAM135A**	**family with sequence similarity 135, member A**
**FAM13B**	**family with sequence similarity 13, member B**
**FAM149B1**	**family with sequence similarity 149, member B1**
**FAM160A2**	**family with sequence similarity 160, member A2**
**FAM161A**	**family with sequence similarity 161, member A**
**FAM161B**	**family with sequence similarity 161, member B**
**FAM162A**	**family with sequence similarity 162, member A**
**FAM167A**	**family with sequence similarity 167, member A**
**FAM168A**	**family with sequence similarity 168, member A**
**FAM172A**	**family with sequence similarity 172, member A**
**FAM172BP**	**family with sequence similarity 172, member B pseudogene**
**FAM173A**	**family with sequence similarity 173, member A**
**FAM173B**	**family with sequence similarity 173, member B**
**FAM175A**	**family with sequence similarity 175, member A**
**FAM177A1**	**family with sequence similarity 177, member A1**
**FAM178A**	**family with sequence similarity 178, member A**
**FAM179B**	**family with sequence similarity 179, member B**
**FAM184A**	**family with sequence similarity 184, member A**
**FAM199X**	**family with sequence similarity 199, X-linked**
**FAM200A**	**family with sequence similarity 200, member A**
**FAM200B**	**family with sequence similarity 200, member B**
**FAM207A**	**family with sequence similarity 207, member A**
**FAM208A**	**family with sequence similarity 208, member A**
**FAM210A**	**family with sequence similarity 210, member A**
**FAM212A**	**family with sequence similarity 212, member A**
**FAM212B**	**family with sequence similarity 212, member B**
**FAM213A**	**family with sequence similarity 213, member A**
**FAM216A**	**family with sequence similarity 216, member A**
**FAM218A**	**family with sequence similarity 218, member A**
**FAM219B**	**family with sequence similarity 219, member B**
**FAM222A**	**family with sequence similarity 222, member A**

**FAM222B**	**family with sequence similarity 222, member B**
**FAM227B**	**family with sequence similarity 227, member B**
**FAM32A**	**family with sequence similarity 32, member A**
**FAM35A**	**family with sequence similarity 35, member A**
**FAM3C**	**family with sequence similarity 3, member C**
**FAM41C**	**family with sequence similarity 41, member C**
**FAM49B**	**family with sequence similarity 49, member B**
**FAM50B**	**family with sequence similarity 50, member B**
**FAM53C**	**family with sequence similarity 53, member C**
**FAM60A**	**family with sequence similarity 60, member A**
**FAM63B**	**family with sequence similarity 63, member B**
**FAM69A**	**family with sequence similarity 69, member A**
**FAM76B**	**family with sequence similarity 76, member B**
**FAM78A**	**family with sequence similarity 78, member A**
**FAM83D**	**family with sequence similarity 83, member D**
**FAM83E**	**family with sequence similarity 83, member E**
**FAM8A1**	**family with sequence similarity 8, member A1**
**FAM96A**	**family with sequence similarity 96, member A**
**FAM96B**	**family with sequence similarity 96, member B**
**FAM98A**	**family with sequence similarity 98, member A**
**FAM98B**	**family with sequence similarity 98, member B**
**FANCA**	**Fanconi anemia, complementation group A**
**FANCD2**	**Fanconi anemia, complementation group D2**
**FANCF**	**Fanconi anemia, complementation group F**
**FANCG**	**Fanconi anemia, complementation group G**
**FARP2**	**FERM, RhoGEF and pleckstrin domain protein 2**
**FARSA**	**phenylalanyl-tRNA synthetase, alpha subunit**
**FARSB**	**phenylalanyl-tRNA synthetase, beta subunit**
**FAS**	**Fas cell surface death receptor**
**FAS-AS1**	**FAS antisense RNA 1**
**FASN**	**fatty acid synthase**
**FASTK**	**Fas-activated serine/threonine kinase**
**FASTKD1**	**FAST kinase domains 1**
**FASTKD2**	**FAST kinase domains 2**
**FASTKD5**	**FAST kinase domains 5**
**FAU**	**Finkel-Biskis-Reilly murine sarcoma virus (FBR-MuSV) ubiquitously expressed**
**FBL**	**fibrillarin**
**FBXL12**	**F-box and leucine-rich repeat protein 12**
**FBXL14**	**F-box and leucine-rich repeat protein 14**
**FBXL15**	**F-box and leucine-rich repeat protein 15**
**FBXL17**	**F-box and leucine-rich repeat protein 17**

**FBXL18**	**F-box and leucine-rich repeat protein 18**
**FBXL19**	**F-box and leucine-rich repeat protein 19**
**FBXL19-AS1**	**FBXL19 antisense RNA 1 (head to head)**
**FBXL20**	**F-box and leucine-rich repeat protein 20**
**FBXL22**	**F-box and leucine-rich repeat protein 22**
**FBXL5**	**F-box and leucine-rich repeat protein 5**
**FBXL8**	**F-box and leucine-rich repeat protein 8**
**FBXO10**	**F-box protein 10**
**FBXO11**	**F-box protein 11**
**FBXO15**	**F-box protein 15**
**FBXO18**	**F-box protein, helicase, 18**
**FBXO22**	**F-box protein 22**
**FBXO24**	**F-box protein 24**
**FBXO30**	**F-box protein 30**
**FBXO31**	**F-box protein 31**
**FBXO33**	**F-box protein 33**
**FBXO34**	**F-box protein 34**
**FBXO36**	**F-box protein 36**
**FBXO38**	**F-box protein 38**
**FBXO4**	**F-box protein 4**
**FBXO41**	**F-box protein 41**
**FBXO45**	**F-box protein 45**
**FBXO46**	**F-box protein 46**
**FBXO5**	**F-box protein 5**
**FBXO8**	**F-box protein 8**
**FBXW11**	**F-box and WD repeat domain containing 11**
**FBXW2**	**F-box and WD repeat domain containing 2**
**FBXW4**	**F-box and WD repeat domain containing 4**
**FBXW5**	**F-box and WD repeat domain containing 5**
**FBXW7**	**F-box and WD repeat domain containing 7, E3 ubiquitin protein ligase**
**FBXW8**	**F-box and WD repeat domain containing 8**
**FBXW9**	**F-box and WD repeat domain containing 9**
**FCER2**	**Fc fragment of IgE, low affinity II, receptor for (CD23)**
**FCHSD2**	**FCH and double SH3 domains 2**
**FCMR**	**Fc fragment of IgM receptor**
**FCRL5**	**Fc receptor-like 5**
**FCRLA**	**Fc receptor-like A**
**FDPS**	**farnesyl diphosphate synthase**
**FDX1L**	**ferredoxin 1-like**
**FDXACB1**	**ferredoxin-fold anticodon binding domain containing 1**
**FDXR**	**ferredoxin reductase**

**FEM1A**	**fem-1 homolog a (C. elegans)**
**FEM1B**	**fem-1 homolog b (C. elegans)**
**FEN1**	**flap structure-specific endonuclease 1**
**FER**	**fer (fps/fes related) tyrosine kinase**
**FERMT3**	**fermitin family member 3**
**FFAR1**	**free fatty acid receptor 1**
**FGD3**	**FYVE, RhoGEF and PH domain containing 3**
**FGD6**	**FYVE, RhoGEF and PH domain containing 6**
**FGF11**	**fibroblast growth factor 11**
**FGF14-IT1**	**FGF14 intronic transcript 1**
**FGF2**	**fibroblast growth factor 2 (basic)**
**FGFR1OP2**	**FGFR1 oncogene partner 2**
**FGR**	**FGR proto-oncogene, Src family tyrosine kinase**
**FHOD1**	**formin homology 2 domain containing 1**
**FIBP**	**fibroblast growth factor (acidic) intracellular binding protein**
**FIP1L1**	**factor interacting with PAPOLA and CPSF1**
**FIS1**	**fission 1 (mitochondrial outer membrane) homolog (S. cerevisiae)**
**FIZ1**	**FLT3-interacting zinc finger 1**
**FKBP11**	**FK506 binding protein 11, 19 kDa**
**FKBP15**	**FK506 binding protein 15, 133kDa**
**FKBP2**	**FK506 binding protein 2, 13kDa**
**FKBP5**	**FK506 binding protein 5**
**FKBP7**	**FK506 binding protein 7**
**FKTN**	**fukutin**
**FLAD1**	**flavin adenine dinucleotide synthetase 1**
**FLI1**	**Fli-1 proto-oncogene, ETS transcription factor**
**FLJ10038**	**uncharacterized protein FLJ10038**
**FLJ13224**	**uncharacterized LOC79857**
**FLJ21408**	**uncharacterized LOC400512**
**FLJ22447**	**uncharacterized LOC400221**
**FLJ31306**	
**FLJ37035**	**uncharacterized LOC399821**
**FLJ37453**	**uncharacterized LOC729614**
**FLJ42351**	**uncharacterized LOC400999**
**FLOT2**	**flotillin 2**
**FLT3LG**	**fms-related tyrosine kinase 3 ligand**
**FLVCR1**	**feline leukemia virus subgroup C cellular receptor 1**
**FLVCR1-AS1**	**FLVCR1 antisense RNA 1 (head to head)**
**FLYWCH2**	**FLYWCH family member 2**
**FMNL3**	**formin-like 3**
**FNBP1**	**formin binding protein 1**

**FNBP4**	**formin binding protein 4**
**FNDC3A**	**fibronectin type III domain containing 3A**
**FNDC3B**	**fibronectin type III domain containing 3B**
**FNIP1**	**folliculin interacting protein 1**
**FNTA**	**farnesyltransferase, CAAX box, alpha**
**FNTB**	**farnesyltransferase, CAAX box, beta**
**FOSB**	**FBJ murine osteosarcoma viral oncogene homolog B**
**FOXI1**	**forkhead box I1**
**FOXJ3**	**forkhead box J3**
**FOXN2**	**forkhead box N2**
**FOXO3**	**forkhead box O3**
**FOXP4**	**forkhead box P4**
**FOXRED2**	**FAD-dependent oxidoreductase domain containing 2**
**FPGS**	**folylpolyglutamate synthase**
**FPGT**	**fucose-1-phosphate guanylyltransferase**
**FPGT-TNNI3K**	**FPGT-TNNI3K readthrough**
**FRA10AC1**	**fragile site, folic acid type, rare, fra(10)(q23.3) or fra(10)(q24.2) candidate 1**
**FRG1**	**FSHD region gene 1**
**FRS3**	**fibroblast growth factor receptor substrate 3**
**FSCN1**	**fascin actin-bundling protein 1**
**FSD1L**	**fibronectin type III and SPRY domain containing 1-like**
**FSD2**	**fibronectin type III and SPRY domain containing 2**
**FTH1**	**ferritin, heavy polypeptide 1**
**FTL**	**ferritin, light polypeptide**
**FTSJ2**	**FtsJ RNA methyltransferase homolog 2 (E. coli)**
**FTSJ3**	**FtsJ homolog 3 (E. coli)**
**FUBP1**	**far upstream element (FUSE) binding protein 1**
**FUCA2**	**fucosidase, alpha-L- 2, plasma**
**FUK**	**fucokinase**
**FUNDC2**	**FUN14 domain containing 2**
**FUS**	**FUS RNA binding protein**
**FUT10**	**fucosyltransferase 10 (alpha (1,3) fucosyltransferase)**
**FUT11**	**fucosyltransferase 11 (alpha (1,3) fucosyltransferase)**
**FUT8**	**fucosyltransferase 8 (alpha (1,6) fucosyltransferase)**
**FYCO1**	**FYVE and coiled-coil domain containing 1**
**FYTTD1**	**forty-two-three domain containing 1**
**FZR1**	**fizzy/cell division cycle 20 related 1 (Drosophila)**
**G0S2**	**G0/G1 switch 2**
**G3BP1**	**GTPase activating protein (SH3 domain) binding protein 1**
**G3BP2**	**GTPase activating protein (SH3 domain) binding protein 2**
**G6PC3**	**glucose 6 phosphatase, catalytic, 3**

GAB2	GRB2-associated binding protein 2
GABARAP	GABA(A) receptor-associated protein
GABPA	GA binding protein transcription factor, alpha subunit 60kDa
GABPB1	GA binding protein transcription factor, beta subunit 1
GABPB2	GA binding protein transcription factor, beta subunit 2
GADD45A	growth arrest and DNA-damage-inducible, alpha
GADD45B	growth arrest and DNA-damage-inducible, beta
GADD45GIP1	growth arrest and DNA-damage-inducible, gamma interacting protein 1
GAK	cyclin G associated kinase
GALE	UDP-galactose-4-epimerase
GALK2	galactokinase 2
GALM	galactose mutarotase (aldose 1-epimerase)
GALNT4	polypeptide N-acetylgalactosaminyltransferase 4
GALR2	galanin receptor 2
GANAB	glucosidase, alpha; neutral AB
GANC	glucosidase, alpha; neutral C
GAPDH	glyceraldehyde-3-phosphate dehydrogenase
GAPVD1	GTPase activating protein and VPS9 domains 1
GAR1	GAR1 ribonucleoprotein
GARS	glycyl-tRNA synthetase
GART	phosphoribosylglycinamide formyltransferase, phosphoribosylglycinamide synthetase, phosphoribosylaminoimidazole synthetase
GAS5	growth arrest-specific 5 (non-protein coding)
GATAD1	GATA zinc finger domain containing 1
GATAD2B	GATA zinc finger domain containing 2B
GATB	glutamyl-tRNA(Gln) amidotransferase, subunit B
GATC	glutamyl-tRNA(Gln) amidotransferase, subunit C
GATS	GATS, stromal antigen 3 opposite strand
GATSL3	GATS protein-like 3
GBA	glucosidase, beta, acid
GBAP1	glucosidase, beta, acid pseudogene 1
GBE1	glucan (1,4-alpha-), branching enzyme 1
GCA	grancalcin, EF-hand calcium binding protein
GCDH	glutaryl-CoA dehydrogenase
GCFC2	GC-rich sequence DNA-binding factor 2
GCHFR	GTP cyclohydrolase I feedback regulator
GCLC	glutamate-cysteine ligase, catalytic subunit
GCN1L1	GCN1 general control of amino-acid synthesis 1-like 1 (yeast)
GCNT1	glucosaminyl (N-acetyl) transferase 1, core 2
GDF9	growth differentiation factor 9
GDI2	GDP dissociation inhibitor 2
GEM	GTP binding protein overexpressed in skeletal muscle

GEMIN7	gem (nuclear organelle) associated protein 7
GET4	golgi to ER traffic protein 4 homolog (S. cerevisiae)
GFER	growth factor, augmenter of liver regeneration
GFI1	growth factor independent 1 transcription repressor
GFM1	G elongation factor, mitochondrial 1
GFM2	G elongation factor, mitochondrial 2
GFOD2	glucose-fructose oxidoreductase domain containing 2
GGA1	golgi-associated, gamma adaptin ear containing, ARF binding protein 1
GGA2	golgi-associated, gamma adaptin ear containing, ARF binding protein 2
GGA3	golgi-associated, gamma adaptin ear containing, ARF binding protein 3
GGCT	gamma-glutamylcyclotransferase
GGCX	gamma-glutamyl carboxylase
GGN	gametogenetin
GGNBP2	gametogenetin binding protein 2
GGPS1	geranylgeranyl diphosphate synthase 1
GGT5	gamma-glutamyltransferase 5
GHITM	growth hormone inducible transmembrane protein
GHRL	ghrelin/obestatin prepropeptide
GID4	GID complex subunit 4
GIMAP8	GTPase, IMAP family member 8
GIN1	gypsy retrotransposon integrase 1
GINM1	glycoprotein integral membrane 1
GINS1	GINS complex subunit 1 (Psf1 homolog)
GINS3	GINS complex subunit 3 (Psf3 homolog)
GINS4	GINS complex subunit 4 (Sld5 homolog)
GIPR	gastric inhibitory polypeptide receptor
GLB1	galactosidase, beta 1
GLB1L	galactosidase, beta 1-like
GLCCI1	glucocorticoid induced 1
GLE1	GLE1 RNA export mediator
GLG1	golgi glycoprotein 1
GLI1	GLI family zinc finger 1
GLI4	GLI family zinc finger 4
GLIPR1L2	GLI pathogenesis-related 1 like 2
GLOD4	glyoxalase domain containing 4
GLS	glutaminase
GLS2	glutaminase 2 (liver, mitochondrial)
GLTPD2	glycolipid transfer protein domain containing 2
GLTSCR2	glioma tumor suppressor candidate region gene 2
GLUD1	glutamate dehydrogenase 1
GLYCTK	glycerate kinase

GMCL1	germ cell-less, spermatogenesis associated 1
GMEB1	glucocorticoid modulatory element binding protein 1
GMEB2	glucocorticoid modulatory element binding protein 2
GMFB	glia maturation factor, beta
GMIP	GEM interacting protein
GMNN	geminin, DNA replication inhibitor
GMPPB	GDP-mannose pyrophosphorylase B
GMPR2	guanosine monophosphate reductase 2
GNA13	guanine nucleotide binding protein (G protein), alpha 13
GNAI2	guanine nucleotide binding protein (G protein), alpha inhibiting activity polypeptide 2
GNAI3	guanine nucleotide binding protein (G protein), alpha inhibiting activity polypeptide 3
GNAL	guanine nucleotide binding protein (G protein), alpha activating activity polypeptide, olfactory type
GNB1L	guanine nucleotide binding protein (G protein), beta polypeptide 1-like
GNB2	guanine nucleotide binding protein (G protein), beta polypeptide 2
GNB2L1	guanine nucleotide binding protein (G protein), beta polypeptide 2-like 1
GNG2	guanine nucleotide binding protein (G protein), gamma 2
GNG3	guanine nucleotide binding protein (G protein), gamma 3
GNG5	guanine nucleotide binding protein (G protein), gamma 5
GNGT2	guanine nucleotide binding protein (G protein), gamma transducing activity polypeptide 2
GNL2	guanine nucleotide binding protein-like 2 (nucleolar)
GNL3	guanine nucleotide binding protein-like 3 (nucleolar)
GNL3L	guanine nucleotide binding protein-like 3 (nucleolar)-like
GNPAT	glyceronephosphate O-acyltransferase
GNRHR2	gonadotropin-releasing hormone (type 2) receptor 2, pseudogene
GOLGA1	golgin A1
GOLGA3	golgin A3
GOLGA4	golgin A4
GOLGA5	golgin A5
GOLGB1	golgin B1
GOLIM4	golgi integral membrane protein 4
GOLPH3	golgi phosphoprotein 3 (coat-protein)
GOLT1B	golgi transport 1B
GORAB	golgin, RAB6-interacting
GORASP1	golgi reassembly stacking protein 1, 65kDa
GORASP2	golgi reassembly stacking protein 2, 55kDa
GOSR2	golgi SNAP receptor complex member 2
GOT2	glutamic-oxaloacetic transaminase 2, mitochondrial
GP1BA	glycoprotein Ib (platelet), alpha polypeptide

GP9	glycoprotein IX (platelet)
GPAA1	glycosylphosphatidylinositol anchor attachment 1
GPALPP1	GPALPP motifs containing 1
GPAM	glycerol-3-phosphate acyltransferase, mitochondrial
GPATCH11	G patch domain containing 11
GPATCH2L	G patch domain containing 2-like
GPATCH3	G patch domain containing 3
GPATCH4	G patch domain containing 4
GPATCH8	G patch domain containing 8
GPBP1	GC-rich promoter binding protein 1
GPBP1L1	GC-rich promoter binding protein 1-like 1
GPCPD1	glycerophosphocholine phosphodiesterase GDE1 homolog (S. cerevisiae)
GPD2	glycerol-3-phosphate dehydrogenase 2 (mitochondrial)
GPER1	G protein-coupled estrogen receptor 1
GPHN	gephyrin
GPN1	GPN-loop GTPase 1
GPN2	GPN-loop GTPase 2
GPN3	GPN-loop GTPase 3
GPR107	G protein-coupled receptor 107
GPR108	G protein-coupled receptor 108
GPR132	G protein-coupled receptor 132
GPR137	G protein-coupled receptor 137
GPR15	G protein-coupled receptor 15
GPR155	G protein-coupled receptor 155
GPR171	G protein-coupled receptor 171
GPR18	G protein-coupled receptor 18
GPR180	G protein-coupled receptor 180
GPR183	G protein-coupled receptor 183
GPR19	G protein-coupled receptor 19
GPR55	G protein-coupled receptor 55
GPS2	G protein pathway suppressor 2
GPX1	glutathione peroxidase 1
GPX3	glutathione peroxidase 3
GPX4	glutathione peroxidase 4
GRAMD1A	GRAM domain containing 1A
GRAMD1C	GRAM domain containing 1C
GRAMD2	GRAM domain containing 2
GRB2	growth factor receptor-bound protein 2
GRIPAP1	GRIP1 associated protein 1
GRK4	G protein-coupled receptor kinase 4
GRK5	G protein-coupled receptor kinase 5

**GRK6**	**G protein-coupled receptor kinase 6**
**GRN**	**granulin**
**GRPEL2**	**GrpE-like 2, mitochondrial (E. coli)**
**GRSF1**	**G-rich RNA sequence binding factor 1**
**GRWD1**	**glutamate-rich WD repeat containing 1**
**GSAP**	**gamma-secretase activating protein**
**GSDMB**	**gasdermin B**
**GSDMD**	**gasdermin D**
**GSE1**	**Gse1 coiled-coil protein**
**GSG2**	**germ cell associated 2 (haspin)**
**GSK3A**	**glycogen synthase kinase 3 alpha**
**GSK3B**	**glycogen synthase kinase 3 beta**
**GSS**	**glutathione synthetase**
**GSTA4**	**glutathione S-transferase alpha 4**
**GSTCD**	**glutathione S-transferase, C-terminal domain containing**
**GSTK1**	**glutathione S-transferase kappa 1**
**GSTO1**	**glutathione S-transferase omega 1**
**GSTO2**	**glutathione S-transferase omega 2**
**GSTT1**	**glutathione S-transferase theta 1**
**GTF2A1**	**general transcription factor IIA, 1, 19/37kDa**
**GTF2A2**	**general transcription factor IIA, 2, 12kDa**
**GTF2E1**	**general transcription factor IIE, polypeptide 1, alpha 56kDa**
**GTF2E2**	**general transcription factor IIE, polypeptide 2, beta 34kDa**
**GTF2H1**	**general transcription factor IIH, polypeptide 1, 62kDa**
**GTF2H3**	**general transcription factor IIH, polypeptide 3, 34kDa**
**GTF2I**	**general transcription factor IIi**
**GTF3A**	**general transcription factor IIIA**
**GTF3C2**	**general transcription factor IIIC, polypeptide 2, beta 110kDa**
**GTF3C3**	**general transcription factor IIIC, polypeptide 3, 102kDa**
**GTF3C4**	**general transcription factor IIIC, polypeptide 4, 90kDa**
**GTF3C5**	**general transcription factor IIIC, polypeptide 5, 63kDa**
**GTF3C6**	**general transcription factor IIIC, polypeptide 6, alpha 35kDa**
**GTPBP10**	**GTP-binding protein 10 (putative)**
**GTPBP2**	**GTP binding protein 2**
**GTPBP3**	**GTP binding protein 3 (mitochondrial)**
**GTPBP4**	**GTP binding protein 4**
**GTPBP8**	**GTP-binding protein 8 (putative)**
**GTSE1**	**G-2 and S-phase expressed 1**
**GTSE1-AS1**	**GTSE1 antisense RNA 1 (head to head)**
**GUCD1**	**guanylyl cyclase domain containing 1**
**GUF1**	**GUF1 GTPase homolog (S. cerevisiae)**

**GUSB**	**glucuronidase, beta**
**GUSBP11**	**glucuronidase, beta pseudogene 11**
**GUSBP5**	**glucuronidase, beta pseudogene 5**
**GYPC**	**glycophorin C (Gerbich blood group)**
**GYS1**	**glycogen synthase 1 (muscle)**
**H1FX**	**H1 histone family, member X**
**H1FX-AS1**	**H1FX antisense RNA 1**
**H2AFJ**	**H2A histone family, member J**
**H2AFV**	**H2A histone family, member V**
**H2AFX**	**H2A histone family, member X**
**H2AFZ**	**H2A histone family, member Z**
**H3F3A**	**H3 histone, family 3A**
**H3F3AP4**	**H3 histone, family 3A, pseudogene 4**
**H3F3B**	**H3 histone, family 3B (H3.3B)**
**H6PD**	**hexose-6-phosphate dehydrogenase (glucose 1-dehydrogenase)**
**HACD2**	**3-hydroxyacyl-CoA dehydratase 2**
**HACL1**	**2-hydroxyacyl-CoA lyase 1**
**HADH**	**hydroxyacyl-CoA dehydrogenase**
**HADHA**	**hydroxyacyl-CoA dehydrogenase/3-ketoacyl-CoA thiolase/enoyl-CoA hydratase (trifunctional protein), alpha subunit**
**HADHB**	**hydroxyacyl-CoA dehydrogenase/3-ketoacyl-CoA thiolase/enoyl-CoA hydratase (trifunctional protein), beta subunit**
**HAGH**	**hydroxyacylglutathione hydrolase**
**HAGHL**	**hydroxyacylglutathione hydrolase-like**
**HAMP**	**hepcidin antimicrobial peptide**
**HARS**	**histidyl-tRNA synthetase**
**HARS2**	**histidyl-tRNA synthetase 2, mitochondrial**
**HAUS4**	**HAUS augmin-like complex, subunit 4**
**HAUS5**	**HAUS augmin-like complex, subunit 5**
**HAUS6**	**HAUS augmin-like complex, subunit 6**
**HAUS8**	**HAUS augmin-like complex, subunit 8**
**HAX1**	**HCLS1 associated protein X-1**
**HBP1**	**HMG-box transcription factor 1**
**HBQ1**	**hemoglobin, theta 1**
**HBS1L**	**HBS1-like translational GTPase**
**HCFC1**	**host cell factor C1**
**HCG11**	**HLA complex group 11 (non-protein coding)**
**HCG25**	**HLA complex group 25 (non-protein coding)**
**HCG26**	**HLA complex group 26 (non-protein coding)**
**HCLS1**	**hematopoietic cell-specific Lyn substrate 1**
**HCRT**	**hypocretin (orexin) neuropeptide precursor**
**HCST**	**hematopoietic cell signal transducer**

HDAC1	histone deacetylase 1
HDAC10	histone deacetylase 10
HDAC2	histone deacetylase 2
HDAC4	histone deacetylase 4
HDDC3	HD domain containing 3
HDGF	hepatoma-derived growth factor
HDHD1	haloacid dehalogenase-like hydrolase domain containing 1
HDHD2	haloacid dehalogenase-like hydrolase domain containing 2
HDLBP	high density lipoprotein binding protein
HEATR1	HEAT repeat containing 1
HEATR5A	HEAT repeat containing 5A
HEATR5B	HEAT repeat containing 5B
HECTD1	HECT domain containing E3 ubiquitin protein ligase 1
HELLS	helicase, lymphoid-specific
HELQ	helicase, POLQ-like
HEMK1	HemK methyltransferase family member 1
HERC1	HECT and RLD domain containing E3 ubiquitin protein ligase family member 1
HERC4	HECT and RLD domain containing E3 ubiquitin protein ligase 4
HERC5	HECT and RLD domain containing E3 ubiquitin protein ligase 5
HERC6	HECT and RLD domain containing E3 ubiquitin protein ligase family member 6
HERPUD1	homocysteine-inducible, endoplasmic reticulum stress-inducible, ubiquitin-like domain member 1
HES1	hes family bHLH transcription factor 1
HEXA	hexosaminidase A (alpha polypeptide)
HEXA-AS1	HEXA antisense RNA 1
HEXIM1	hexamethylene bis-acetamide inducible 1
HEXIM2	hexamethylene bis-acetamide inducible 2
HHLA3	HERV-H LTR-associating 3
HIAT1	hippocampus abundant transcript 1
HIBADH	3-hydroxyisobutyrate dehydrogenase
HIC1	hypermethylated in cancer 1
HIF1A	hypoxia inducible factor 1, alpha subunit (basic helix-loop-helix transcription factor)
HIF1AN	hypoxia inducible factor 1, alpha subunit inhibitor
HIGD1B	HIG1 hypoxia inducible domain family, member 1B
HIGD2A	HIG1 hypoxia inducible domain family, member 2A
HILPDA	hypoxia inducible lipid droplet-associated
HINT1	histidine triad nucleotide binding protein 1
HINT2	histidine triad nucleotide binding protein 2
HINT3	histidine triad nucleotide binding protein 3
HIPK1	homeodomain interacting protein kinase 1
HIPK3	homeodomain interacting protein kinase 3

HIRA	histone cell cycle regulator
HIRIP3	HIRA interacting protein 3
HIST1H1B	histone cluster 1, H1b
HIST1H1C	histone cluster 1, H1c
HIST1H1D	histone cluster 1, H1d
HIST1H1E	histone cluster 1, H1e
HIST1H2AB	histone cluster 1, H2ab
HIST1H2AC	histone cluster 1, H2ac
HIST1H2AD	histone cluster 1, H2ad
HIST1H2AE	histone cluster 1, H2ae
HIST1H2AG	histone cluster 1, H2ag
HIST1H2AH	histone cluster 1, H2ah
HIST1H2AI	histone cluster 1, H2ai
HIST1H2AJ	histone cluster 1, H2aj
HIST1H2AK	histone cluster 1, H2ak
HIST1H2AL	histone cluster 1, H2al
HIST1H2AM	histone cluster 1, H2am
HIST1H2BB	histone cluster 1, H2bb
HIST1H2BC	histone cluster 1, H2bc
HIST1H2BD	histone cluster 1, H2bd
HIST1H2BE	histone cluster 1, H2be
HIST1H2BF	histone cluster 1, H2bf
HIST1H2BG	histone cluster 1, H2bg
HIST1H2BH	histone cluster 1, H2bh
HIST1H2BI	histone cluster 1, H2bi
HIST1H2BJ	histone cluster 1, H2bj
HIST1H2BK	histone cluster 1, H2bk
HIST1H2BL	histone cluster 1, H2bl
HIST1H2BM	histone cluster 1, H2bm
HIST1H2BN	histone cluster 1, H2bn
HIST1H2BO	histone cluster 1, H2bo
HIST1H3A	histone cluster 1, H3a
HIST1H3B	histone cluster 1, H3b
HIST1H3C	histone cluster 1, H3c
HIST1H3D	histone cluster 1, H3d
HIST1H3F	histone cluster 1, H3f
HIST1H3G	histone cluster 1, H3g
HIST1H3H	histone cluster 1, H3h
HIST1H3I	histone cluster 1, H3i
HIST1H3J	histone cluster 1, H3j
HIST1H4A	histone cluster 1, H4a

**HIST1H4B**	**histone cluster 1, H4b**
**HIST1H4C**	**histone cluster 1, H4c**
**HIST1H4D**	**histone cluster 1, H4d**
**HIST1H4E**	**histone cluster 1, H4e**
**HIST1H4F**	**histone cluster 1, H4f**
**HIST1H4H**	**histone cluster 1, H4h**
**HIST1H4I**	**histone cluster 1, H4i**
**HIST1H4J**	**histone cluster 1, H4j**
**HIST1H4K**	**histone cluster 1, H4k**
**HIST1H4L**	**histone cluster 1, H4l**
**HIST2H2AB**	**histone cluster 2, H2ab**
**HIST2H2AC**	**histone cluster 2, H2ac**
**HIST2H2BE**	**histone cluster 2, H2be**
**HIST2H2BF**	**histone cluster 2, H2bf**
**HIST2H3D**	**histone cluster 2, H3d**
**HIST3H2A**	**histone cluster 3, H2a**
**HIST3H2BB**	**histone cluster 3, H2bb**
**HIST4H4**	**histone cluster 4, H4**
**HIVEP1**	**human immunodeficiency virus type I enhancer binding protein 1**
**HIVEP2**	**human immunodeficiency virus type I enhancer binding protein 2**
**HKR1**	**HKR1, GLI-Kruppel zinc finger family member**
**HLA-DQA1**	**major histocompatibility complex, class II, DQ alpha 1**
**HLA-DRB5**	**major histocompatibility complex, class II, DR beta 5**
**HLA-E**	**major histocompatibility complex, class I, E**
**HLA-H**	**major histocompatibility complex, class I, H (pseudogene)**
**HMBOX1**	**homeobox containing 1**
**HMBS**	**hydroxymethylbilane synthase**
**HMCES**	**5-hydroxymethylcytosine (hmC) binding, ES cell-specific**
**HMGA1**	**high mobility group AT-hook 1**
**HMGB1**	**high mobility group box 1**
**HMGB2**	**high mobility group box 2**
**HMGCL**	**3-hydroxymethyl-3-methylglutaryl-CoA lyase**
**HMGCR**	**3-hydroxy-3-methylglutaryl-CoA reductase**
**HMGN2**	**high mobility group nucleosomal binding domain 2**
**HMGN3**	**high mobility group nucleosomal binding domain 3**
**HMGN4**	**high mobility group nucleosomal binding domain 4**
**HMGXB3**	**HMG box domain containing 3**
**HMHA1**	**histocompatibility (minor) HA-1**
**HMMR**	**hyaluronan-mediated motility receptor (RHAMM)**
**HMOX2**	**heme oxygenase 2**
**HMSD**	**histocompatibility (minor) serpin domain containing**

**HN1**	**hematological and neurological expressed 1**
**HN1L**	**hematological and neurological expressed 1-like**
**HNRNPA0**	**heterogeneous nuclear ribonucleoprotein A0**
**HNRNPA1**	**heterogeneous nuclear ribonucleoprotein A1**
**HNRNPA1L2**	**heterogeneous nuclear ribonucleoprotein A1-like 2**
**HNRNPA1P10**	**heterogeneous nuclear ribonucleoprotein A1 pseudogene 10**
**HNRNPA2B1**	**heterogeneous nuclear ribonucleoprotein A2/B1**
**HNRNPA3**	**heterogeneous nuclear ribonucleoprotein A3**
**HNRNPAB**	**heterogeneous nuclear ribonucleoprotein A/B**
**HNRNPC**	**heterogeneous nuclear ribonucleoprotein C (C1/C2)**
**HNRNPD**	**heterogeneous nuclear ribonucleoprotein D (AU-rich element RNA binding protein 1, 37kDa)**
**HNRNPDL**	**heterogeneous nuclear ribonucleoprotein D-like**
**HNRNPF**	**heterogeneous nuclear ribonucleoprotein F**
**HNRNPH1**	**heterogeneous nuclear ribonucleoprotein H1 (H)**
**HNRNPH3**	**heterogeneous nuclear ribonucleoprotein H3 (2H9)**
**HNRNPK**	**heterogeneous nuclear ribonucleoprotein K**
**HNRNPL**	**heterogeneous nuclear ribonucleoprotein L**
**HNRNPLL**	**heterogeneous nuclear ribonucleoprotein L-like**
**HNRNPR**	**heterogeneous nuclear ribonucleoprotein R**
**HNRNPU**	**heterogeneous nuclear ribonucleoprotein U (scaffold attachment factor A)**
**HNRNPU-AS1**	**HNRNPU antisense RNA 1**
**HNRNPUL1**	**heterogeneous nuclear ribonucleoprotein U-like 1**
**HNRNPUL2**	**heterogeneous nuclear ribonucleoprotein U-like 2**
**HNRNPUL2-BSCL2**	**HNRNPUL2-BSCL2 readthrough (NMD candidate)**
**HOMER1**	**homer scaffolding protein 1**
**HOOK2**	**hook microtubule-tethering protein 2**
**HOXC4**	**homeobox C4**
**HOXC5**	**homeobox C5**
**HOXC6**	**homeobox C6**
**HPD**	**4-hydroxyphenylpyruvate dioxygenase**
**HPDL**	**4-hydroxyphenylpyruvate dioxygenase-like**
**HPS4**	**Hermansky-Pudlak syndrome 4**
**HPS5**	**Hermansky-Pudlak syndrome 5**
**HPSE2**	**heparanase 2 (inactive)**
**HRAS**	**Harvey rat sarcoma viral oncogene homolog**
**HS1BP3**	**HCLS1 binding protein 3**
**HS2ST1**	**heparan sulfate 2-O-sulfotransferase 1**
**HSBP1L1**	**heat shock factor binding protein 1-like 1**
**HSD11B1L**	**hydroxysteroid (11-beta) dehydrogenase 1-like**
**HSD17B11**	**hydroxysteroid (17-beta) dehydrogenase 11**

**HSD17B12**	**hydroxysteroid (17-beta) dehydrogenase 12**
**HSD3B7**	**hydroxy-delta-5-steroid dehydrogenase, 3 beta- and steroid delta-isomerase 7**
**HSDL2**	**hydroxysteroid dehydrogenase like 2**
**HSF1**	**heat shock transcription factor 1**
**HSF2BP**	**heat shock transcription factor 2 binding protein**
**HSF4**	**heat shock transcription factor 4**
**HSH2D**	**hematopoietic SH2 domain containing**
**HSP90AB1**	**heat shock protein 90kDa alpha (cytosolic), class B member 1**
**HSP90B1**	**heat shock protein 90kDa beta (Grp94), member 1**
**HSPA12B**	**heat shock 70kD protein 12B**
**HSPA2**	**heat shock 70kDa protein 2**
**HSPA4**	**heat shock 70kDa protein 4**
**HSPA4L**	**heat shock 70kDa protein 4-like**
**HSPA5**	**heat shock 70kDa protein 5 (glucose-regulated protein, 78kDa)**
**HSPA8**	**heat shock 70kDa protein 8**
**HSPA9**	**heat shock 70kDa protein 9 (mortalin)**
**HSPB1**	**heat shock 27kDa protein 1**
**HSPB11**	**heat shock protein family B (small), member 11**
**HSPB6**	**heat shock protein, alpha-crystallin-related, B6**
**HSPBAP1**	**HSPB (heat shock 27kDa) associated protein 1**
**HSPD1**	**heat shock 60kDa protein 1 (chaperonin)**
**HSPE1**	**heat shock 10kDa protein 1**
**HSPE1-MOB4**	**HSPE1-MOB4 readthrough**
**HTR1A**	**5-hydroxytryptamine (serotonin) receptor 1A, G protein-coupled**
**HTR5A**	**5-hydroxytryptamine (serotonin) receptor 5A, G protein-coupled**
**HTRA2**	**HtrA serine peptidase 2**
**HTRA4**	**HtrA serine peptidase 4**
**HTT**	**huntingtin**
**HTT-AS**	**HTT antisense RNA (head to head)**
**HUS1**	**HUS1 checkpoint homolog (S. pombe)**
**HUWE1**	**HECT, UBA and WWE domain containing 1, E3 ubiquitin protein ligase**
**HYAL3**	**hyaluronoglucosaminidase 3**
**HYI**	**hydroxypyruvate isomerase (putative)**
**HYOU1**	**hypoxia up-regulated 1**
**HYPK**	**huntingtin interacting protein K**
**IAH1**	**isoamyl acetate-hydrolyzing esterase 1 homolog (S. cerevisiae)**
**IARS**	**isoleucyl-tRNA synthetase**
**IBA57**	**IBA57, iron-sulfur cluster assembly homolog (S. cerevisiae)**
**IBTK**	**inhibitor of Bruton agammaglobulinemia tyrosine kinase**
**ICAM1**	**intercellular adhesion molecule 1**
**ICAM2**	**intercellular adhesion molecule 2**

**ICAM4**	**intercellular adhesion molecule 4 (Landsteiner-Wiener blood group)**
**ICE1**	**interactor of little elongation complex ELL subunit 1**
**ICE2**	**interactor of little elongation complex ELL subunit 2**
**ICK**	**intestinal cell (MAK-like) kinase**
**ICOSLG**	**inducible T-cell co-stimulator ligand**
**ICT1**	**immature colon carcinoma transcript 1**
**ID2**	**inhibitor of DNA binding 2, dominant negative helix-loop-helix protein**
**ID3**	**inhibitor of DNA binding 3, dominant negative helix-loop-helix protein**
**IDE**	**insulin-degrading enzyme**
**IDH3A**	**isocitrate dehydrogenase 3 (NAD+) alpha**
**IDH3B**	**isocitrate dehydrogenase 3 (NAD+) beta**
**IDI1**	**isopentenyl-diphosphate delta isomerase 1**
**IDI2-AS1**	**IDI2 antisense RNA 1**
**IER2**	**immediate early response 2**
**IER3**	**immediate early response 3**
**IER3IP1**	**immediate early response 3 interacting protein 1**
**IER5**	**immediate early response 5**
**IFI16**	**interferon, gamma-inducible protein 16**
**IFI30**	**interferon, gamma-inducible protein 30**
**IFIH1**	**interferon induced with helicase C domain 1**
**IFITM1**	**interferon induced transmembrane protein 1**
**IFNAR1**	**interferon (alpha, beta and omega) receptor 1**
**IFNAR2**	**interferon (alpha, beta and omega) receptor 2**
**IFNGR1**	**interferon gamma receptor 1**
**IFRD2**	**interferon-related developmental regulator 2**
**IFT172**	**intraflagellar transport 172**
**IFT22**	**intraflagellar transport 22**
**IFT46**	**intraflagellar transport 46**
**IFT52**	**intraflagellar transport 52**
**IFT74**	**intraflagellar transport 74**
**IFT80**	**intraflagellar transport 80**
**IGDCC4**	**immunoglobulin superfamily, DCC subclass, member 4**
**IGF2BP3**	**insulin-like growth factor 2 mRNA binding protein 3**
**IGF2R**	**insulin-like growth factor 2 receptor**
**IGFLR1**	**IGF-like family receptor 1**
**IGHMBP2**	**immunoglobulin mu binding protein 2**
**IK**	**IK cytokine, down-regulator of HLA II**
**IKBIP**	**IKBKB interacting protein**
**IKBKB**	**inhibitor of kappa light polypeptide gene enhancer in B-cells, kinase beta**
**IKZF1**	**IKAROS family zinc finger 1 (Ikaros)**
**IKZF2**	**IKAROS family zinc finger 2 (Helios)**

**IKZF3**	**IKAROS family zinc finger 3 (Aiolos)**
**IL10RA**	**interleukin 10 receptor, alpha**
**IL10RB**	**interleukin 10 receptor, beta**
**IL11RA**	**interleukin 11 receptor, alpha**
**IL12RB1**	**interleukin 12 receptor, beta 1**
**IL18BP**	**interleukin 18 binding protein**
**IL21R**	**interleukin 21 receptor**
**IL22RA2**	**interleukin 22 receptor, alpha 2**
**IL23R**	**interleukin 23 receptor**
**IL2RB**	**interleukin 2 receptor, beta**
**IL2RG**	**interleukin 2 receptor, gamma**
**IL4I1**	**interleukin 4 induced 1**
**IL6ST**	**interleukin 6 signal transducer**
**IL7**	**interleukin 7**
**ILDR1**	**immunoglobulin-like domain containing receptor 1**
**ILF2**	**interleukin enhancer binding factor 2**
**ILF3**	**interleukin enhancer binding factor 3, 90kDa**
**ILK**	**integrin-linked kinase**
**IMMP1L**	**IMP1 inner mitochondrial membrane peptidase-like (S. cerevisiae)**
**IMMT**	**inner membrane protein, mitochondrial**
**IMP3**	**IMP3, U3 small nucleolar ribonucleoprotein**
**IMP4**	**IMP4, U3 small nucleolar ribonucleoprotein**
**IMPA1**	**inositol(myo)-1(or 4)-monophosphatase 1**
**IMPDH2**	**IMP (inosine 5'-monophosphate) dehydrogenase 2**
**INAFM1**	**InaF-motif containing 1**
**ING1**	**inhibitor of growth family, member 1**
**ING3**	**inhibitor of growth family, member 3**
**ING4**	**inhibitor of growth family, member 4**
**ING5**	**inhibitor of growth family, member 5**
**INO80B**	**INO80 complex subunit B**
**INO80B-WBP1**	**INO80B-WBP1 readthrough (NMD candidate)**
**INO80C**	**INO80 complex subunit C**
**INO80D**	**INO80 complex subunit D**
**INO80E**	**INO80 complex subunit E**
**INPP5D**	**inositol polyphosphate-5-phosphatase, 145kDa**
**INPP5E**	**inositol polyphosphate-5-phosphatase, 72 kDa**
**INPP5F**	**inositol polyphosphate-5-phosphatase F**
**INPP5K**	**inositol polyphosphate-5-phosphatase K**
**INSIG1**	**insulin induced gene 1**
**INSM2**	**insulinoma-associated 2**
**INTS1**	**integrator complex subunit 1**

**INTS10**	**integrator complex subunit 10**
**INTS12**	**integrator complex subunit 12**
**INTS2**	**integrator complex subunit 2**
**INTS4**	**integrator complex subunit 4**
**INTS5**	**integrator complex subunit 5**
**INTS6**	**integrator complex subunit 6**
**INTS7**	**integrator complex subunit 7**
**INTS9**	**integrator complex subunit 9**
**IP6K1**	**inositol hexakisphosphate kinase 1**
**IPCEF1**	**interaction protein for cytohesin exchange factors 1**
**IPMK**	**inositol polyphosphate multikinase**
**IPO13**	**importin 13**
**IPO4**	**importin 4**
**IPO7**	**importin 7**
**IPO9**	**importin 9**
**IPP**	**intracisternal A particle-promoted polypeptide**
**IPPK**	**inositol 1,3,4,5,6-pentakisphosphate 2-kinase**
**IQCB1**	**IQ motif containing B1**
**IQCC**	**IQ motif containing C**
**IQCD**	**IQ motif containing D**
**IQCE**	**IQ motif containing E**
**IQCH**	**IQ motif containing H**
**IQGAP2**	**IQ motif containing GTPase activating protein 2**
**IRAK1**	**interleukin-1 receptor-associated kinase 1**
**IRAK1BP1**	**interleukin-1 receptor-associated kinase 1 binding protein 1**
**IRAK4**	**interleukin-1 receptor-associated kinase 4**
**IREB2**	**iron-responsive element binding protein 2**
**IRF1**	**interferon regulatory factor 1**
**IRF2**	**interferon regulatory factor 2**
**IRF2BP1**	**interferon regulatory factor 2 binding protein 1**
**IRF2BP2**	**interferon regulatory factor 2 binding protein 2**
**IRF2BPL**	**interferon regulatory factor 2 binding protein-like**
**IRF3**	**interferon regulatory factor 3**
**IRF4**	**interferon regulatory factor 4**
**IRF5**	**interferon regulatory factor 5**
**IRF7**	**interferon regulatory factor 7**
**IRF8**	**interferon regulatory factor 8**
**IRF9**	**interferon regulatory factor 9**
**IRGM**	**immunity-related GTPase family, M**
**IRGQ**	**immunity-related GTPase family, Q**
**ISCU**	**iron-sulfur cluster assembly enzyme**

ISG15	ISG15 ubiquitin-like modifier
ISG20	interferon stimulated exonuclease gene 20kDa
ISG20L2	interferon stimulated exonuclease gene 20kDa-like 2
ISOC1	isochorismatase domain containing 1
IST1	increased sodium tolerance 1 homolog (yeast)
ISY1	ISY1 splicing factor homolog (S. cerevisiae)
ISY1-RAB43	ISY1-RAB43 readthrough
ISYNA1	inositol-3-phosphate synthase 1
ITFG2	integrin alpha FG-GAP repeat containing 2
ITFG3	integrin alpha FG-GAP repeat containing 3
ITGA4	integrin, alpha 4 (antigen CD49D, alpha 4 subunit of VLA-4 receptor)
ITGAL	integrin, alpha L (antigen CD11A (p180), lymphocyte function-associated antigen 1; alpha polypeptide)
ITGAV	integrin, alpha V
ITGAX	integrin, alpha X (complement component 3 receptor 4 subunit)
ITGB1	integrin, beta 1 (fibronectin receptor, beta polypeptide, antigen CD29 includes MDF2, MSK12)
ITGB1BP1	integrin beta 1 binding protein 1
ITGB1BP2	integrin beta 1 binding protein (melusin) 2
ITGB3	integrin, beta 3 (platelet glycoprotein IIIa, antigen CD61)
ITGB3BP	integrin beta 3 binding protein (beta3-endonexin)
ITGB7	integrin, beta 7
ITGB8	integrin, beta 8
ITPA	inosine triphosphatase (nucleoside triphosphate pyrophosphatase)
ITPK1-AS1	ITPK1 antisense RNA 1
ITPKB	inositol-trisphosphate 3-kinase B
ITPKC	inositol-trisphosphate 3-kinase C
ITPR1	inositol 1,4,5-trisphosphate receptor, type 1
ITPR2	inositol 1,4,5-trisphosphate receptor, type 2
ITPR3	inositol 1,4,5-trisphosphate receptor, type 3
ITPRIPL1	inositol 1,4,5-trisphosphate receptor interacting protein-like 1
ITSN2	intersectin 2
IVL	involucrin
IVNS1ABP	influenza virus NS1A binding protein
IWS1	IWS1 homolog (S. cerevisiae)
IYD	iodotyrosine deiodinase
IZUMO4	IZUMO family member 4
JADE1	jade family PHD finger 1
JADE2	jade family PHD finger 2
JAGN1	jagunal homolog 1 (Drosophila)
JAK2	Janus kinase 2
JAK3	Janus kinase 3

**JMJD1C**	**jumonji domain containing 1C**
**JMJD4**	**jumonji domain containing 4**
**JMJD6**	**jumonji domain containing 6**
**JMJD7**	**jumonji domain containing 7**
**JMJD7-PLA2G4B**	**JMJD7-PLA2G4B readthrough**
**JMJD8**	**jumonji domain containing 8**
**JOSD1**	**Josephin domain containing 1**
**JOSD2**	**Josephin domain containing 2**
**JPH4**	**junctophilin 4**
**JPX**	**JPX transcript, XIST activator (non-protein coding)**
**JTB**	**jumping translocation breakpoint**
**JUN**	**jun proto-oncogene**
**JUNB**	**jun B proto-oncogene**
**JUND**	**jun D proto-oncogene**
**KANSL1L**	**KAT8 regulatory NSL complex subunit 1-like**
**KANSL2**	**KAT8 regulatory NSL complex subunit 2**
**KANSL3**	**KAT8 regulatory NSL complex subunit 3**
**KARS**	**lysyl-tRNA synthetase**
**KAT5**	**K(lysine) acetyltransferase 5**
**KAT6A**	**K(lysine) acetyltransferase 6A**
**KAT6B**	**K(lysine) acetyltransferase 6B**
**KAT7**	**K(lysine) acetyltransferase 7**
**KAT8**	**K(lysine) acetyltransferase 8**
**KATNA1**	**katanin p60 (ATPase containing) subunit A 1**
**KATNBL1**	**katanin p80 subunit B-like 1**
**KAZALD1**	**Kazal-type serine peptidase inhibitor domain 1**
**KBTBD2**	**kelch repeat and BTB (POZ) domain containing 2**
**KBTBD4**	**kelch repeat and BTB (POZ) domain containing 4**
**KBTBD6**	**kelch repeat and BTB (POZ) domain containing 6**
**KBTBD7**	**kelch repeat and BTB (POZ) domain containing 7**
**KBTBD8**	**kelch repeat and BTB (POZ) domain containing 8**
**KCMF1**	**potassium channel modulatory factor 1**
**KCNC3**	**potassium channel, voltage gated Shaw related subfamily C, member 3**
**KCND1**	**potassium channel, voltage gated Shal related subfamily D, member 1**
**KCNIP2**	**Kv channel interacting protein 2**
**KCNK7**	**potassium channel, two pore domain subfamily K, member 7**
**KCNMB2**	**potassium channel subfamily M regulatory beta subunit 2**
**KCNN4**	**potassium channel, calcium activated intermediate/small conductance subfamily N alpha, member 4**
**KCTD11**	**potassium channel tetramerization domain containing 11**
**KCTD18**	**potassium channel tetramerization domain containing 18**
**KCTD20**	**potassium channel tetramerization domain containing 20**

**KCTD5**	**potassium channel tetramerization domain containing 5**
**KCTD9**	**potassium channel tetramerization domain containing 9**
**KDELC1**	**KDEL (Lys-Asp-Glu-Leu) containing 1**
**KDELC2**	**KDEL (Lys-Asp-Glu-Leu) containing 2**
**KDELR1**	**KDEL (Lys-Asp-Glu-Leu) endoplasmic reticulum protein retention receptor 1**
**KDM1A**	**lysine (K)-specific demethylase 1A**
**KDM1B**	**lysine (K)-specific demethylase 1B**
**KDM2A**	**lysine (K)-specific demethylase 2A**
**KDM3A**	**lysine (K)-specific demethylase 3A**
**KDM3B**	**lysine (K)-specific demethylase 3B**
**KDM4A**	**lysine (K)-specific demethylase 4A**
**KDM4B**	**lysine (K)-specific demethylase 4B**
**KDM4C**	**lysine (K)-specific demethylase 4C**
**KDM5A**	**lysine (K)-specific demethylase 5A**
**KDM5C**	**lysine (K)-specific demethylase 5C**
**KDSR**	**3-ketodihydrosphingosine reductase**
**KEAP1**	**kelch-like ECH-associated protein 1**
**KHDRBS1**	**KH domain containing, RNA binding, signal transduction associated 1**
**KHK**	**ketohexokinase (fructokinase)**
**KHNYN**	**KH and NYN domain containing**
**KHSRP**	**KH-type splicing regulatory protein**
**KIAA0020**	**KIAA0020**
**KIAA0100**	**KIAA0100**
**KIAA0141**	**KIAA0141**
**KIAA0226**	**KIAA0226**
**KIAA0226L**	**KIAA0226-like**
**KIAA0319L**	**KIAA0319-like**
**KIAA0368**	**KIAA0368**
**KIAA0391**	**KIAA0391**
**KIAA0586**	**KIAA0586**
**KIAA0753**	**KIAA0753**
**KIAA0825**	**KIAA0825**
**KIAA0907**	**KIAA0907**
**KIAA0922**	**KIAA0922**
**KIAA1033**	**KIAA1033**
**KIAA1143**	**KIAA1143**
**KIAA1429**	**KIAA1429**
**KIAA1524**	**KIAA1524**
**KIAA1551**	**KIAA1551**
**KIAA1683**	**KIAA1683**
**KIAA1715**	**KIAA1715**

KIAA1919	KIAA1919
KIAA1958	KIAA1958
KIAA2026	KIAA2026
KIF11	kinesin family member 11
KIF13A	kinesin family member 13A
KIF15	kinesin family member 15
KIF18B	kinesin family member 18B
KIF1BP	KIF1 binding protein
KIF20A	kinesin family member 20A
KIF20B	kinesin family member 20B
KIF21A	kinesin family member 21A
KIF21B	kinesin family member 21B
KIF22	kinesin family member 22
KIF27	kinesin family member 27
KIF2A	kinesin heavy chain member 2A
KIF2C	kinesin family member 2C
KIF3B	kinesin family member 3B
KIF9	kinesin family member 9
KIFC3	kinesin family member C3
KLC2	kinesin light chain 2
KLC3	kinesin light chain 3
KLC4	kinesin light chain 4
KLF10	Kruppel-like factor 10
KLF12	Kruppel-like factor 12
KLF13	Kruppel-like factor 13
KLF6	Kruppel-like factor 6
KLF7	Kruppel-like factor 7 (ubiquitous)
KLHDC10	kelch domain containing 10
KLHDC2	kelch domain containing 2
KLHDC4	kelch domain containing 4
KLHDC9	kelch domain containing 9
KLHL11	kelch-like family member 11
KLHL12	kelch-like family member 12
KLHL18	kelch-like family member 18
KLHL20	kelch-like family member 20
KLHL21	kelch-like family member 21
KLHL24	kelch-like family member 24
KLHL28	kelch-like family member 28
KLHL6	kelch-like family member 6
KLHL8	kelch-like family member 8
KLKP1	kallikrein pseudogene 1

**KLLN**	**killin, p53-regulated DNA replication inhibitor**
**KLRD1**	**killer cell lectin-like receptor subfamily D, member 1**
**KMO**	**kynurenine 3-monooxygenase (kynurenine 3-hydroxylase)**
**KMT2C**	**lysine (K)-specific methyltransferase 2C**
**KMT2D**	**lysine (K)-specific methyltransferase 2D**
**KMT2E**	**lysine (K)-specific methyltransferase 2E**
**KNSTRN**	**kinetochore-localized astrin/SPAG5 binding protein**
**KNTC1**	**kinetochore associated 1**
**KPNA5**	**karyopherin alpha 5 (importin alpha 6)**
**KPNA6**	**karyopherin alpha 6 (importin alpha 7)**
**KPNB1**	**karyopherin (importin) beta 1**
**KPTN**	**kaptin (actin binding protein)**
**KRI1**	**KRI1 homolog (S. cerevisiae)**
**KRR1**	**KRR1, small subunit (SSU) processome component, homolog (yeast)**
**KRTAP21-3**	**keratin associated protein 21-3**
**KRTCAP2**	**keratinocyte associated protein 2**
**KSR1**	**kinase suppressor of ras 1**
**KTI12**	**KTI12 homolog, chromatin associated (S. cerevisiae)**
**KXD1**	**KxDL motif containing 1**
**L3MBTL2**	**l(3)mbt-like 2 (Drosophila)**
**LACE1**	**lactation elevated 1**
**LAGE3**	**L antigen family, member 3**
**LAMC1**	**laminin, gamma 1 (formerly LAMB2)**
**LAMP1**	**lysosomal-associated membrane protein 1**
**LAMP3**	**lysosomal-associated membrane protein 3**
**LAMTOR1**	**late endosomal/lysosomal adaptor, MAPK and MTOR activator 1**
**LAMTOR2**	**late endosomal/lysosomal adaptor, MAPK and MTOR activator 2**
**LAMTOR3**	**late endosomal/lysosomal adaptor, MAPK and MTOR activator 3**
**LAMTOR4**	**late endosomal/lysosomal adaptor, MAPK and MTOR activator 4**
**LAMTOR5**	**late endosomal/lysosomal adaptor, MAPK and MTOR activator 5**
**LANCL2**	**LanC lantibiotic synthetase component C-like 2 (bacterial)**
**LAP3**	**leucine aminopeptidase 3**
**LAPTM4A**	**lysosomal protein transmembrane 4 alpha**
**LAPTM5**	**lysosomal protein transmembrane 5**
**LARP1B**	**La ribonucleoprotein domain family, member 1B**
**LARP4**	**La ribonucleoprotein domain family, member 4**
**LARP7**	**La ribonucleoprotein domain family, member 7**
**LARS**	**leucyl-tRNA synthetase**
**LAS1L**	**LAS1-like (S. cerevisiae)**
**LASP1**	**LIM and SH3 protein 1**
**LAT2**	**linker for activation of T cells family, member 2**

**LATS1**	**large tumor suppressor kinase 1**
**LAX1**	**lymphocyte transmembrane adaptor 1**
**LBH**	**limb bud and heart development**
**LBX2**	**ladybird homeobox 2**
**LCAT**	**lecithin-cholesterol acyltransferase**
**LCK**	**LCK proto-oncogene, Src family tyrosine kinase**
**LCLAT1**	**lysocardiolipin acyltransferase 1**
**LCMT2**	**leucine carboxyl methyltransferase 2**
**LCP1**	**lymphocyte cytosolic protein 1 (L-plastin)**
**LDAH**	**lipid droplet associated hydrolase**
**LDHA**	**lactate dehydrogenase A**
**LDHB**	**lactate dehydrogenase B**
**LDLR**	**low density lipoprotein receptor**
**LEAP2**	**liver expressed antimicrobial peptide 2**
**LEKR1**	**leucine, glutamate and lysine rich 1**
**LEMD2**	**LEM domain containing 2**
**LEMD3**	**LEM domain containing 3**
**LENEP**	**lens epithelial protein**
**LENG1**	**leukocyte receptor cluster (LRC) member 1**
**LENG8**	**leukocyte receptor cluster (LRC) member 8**
**LENG9**	**leukocyte receptor cluster (LRC) member 9**
**LETM1**	**leucine zipper-EF-hand containing transmembrane protein 1**
**LETM2**	**leucine zipper-EF-hand containing transmembrane protein 2**
**LETMD1**	**LETM1 domain containing 1**
**LGALS1**	**lectin, galactoside-binding, soluble, 1**
**LGALS17A**	**Charcot-Leyden crystal protein pseudogene**
**LHB**	**luteinizing hormone beta polypeptide**
**LIAS**	**lipoic acid synthetase**
**LILRB1**	**leukocyte immunoglobulin-like receptor, subfamily B (with TM and ITIM domains), member 1**
**LIMD1**	**LIM domains containing 1**
**LIMD2**	**LIM domain containing 2**
**LIME1**	**Lck interacting transmembrane adaptor 1**
**LIN52**	**lin-52 DREAM MuvB core complex component**
**LIN54**	**lin-54 DREAM MuvB core complex component**
**LIN7B**	**lin-7 homolog B (C. elegans)**
**LIN7C**	**lin-7 homolog C (C. elegans)**
**LIN9**	**lin-9 DREAM MuvB core complex component**
**LINC00029**	**long intergenic non-protein coding RNA 29**
**LINC00167**	**long intergenic non-protein coding RNA 167**
**LINC00176**	**long intergenic non-protein coding RNA 176**
**LINC00200**	**long intergenic non-protein coding RNA 200**

**LINC00235**	**long intergenic non-protein coding RNA 235**
**LINC00265**	**long intergenic non-protein coding RNA 265**
**LINC00273**	**long intergenic non-protein coding RNA 273**
**LINC00309**	**long intergenic non-protein coding RNA 309**
**LINC00324**	**long intergenic non-protein coding RNA 324**
**LINC00339**	**long intergenic non-protein coding RNA 339**
**LINC00426**	**long intergenic non-protein coding RNA 426**
**LINC00467**	**long intergenic non-protein coding RNA 467**
**LINC00471**	**long intergenic non-protein coding RNA 471**
**LINC00476**	**long intergenic non-protein coding RNA 476**
**LINC00493**	**long intergenic non-protein coding RNA 493**
**LINC00515**	**long intergenic non-protein coding RNA 515**
**LINC00526**	**long intergenic non-protein coding RNA 526**
**LINC01465**	**long intergenic non-protein coding RNA 1465**
**LINC01547**	**long intergenic non-protein coding RNA 1547**
**LINC01550**	**long intergenic non-protein coding RNA 1550**
**LINGO3**	**leucine rich repeat and Ig domain containing 3**
**LIPH**	**lipase, member H**
**LIPT1**	**lipoyltransferase 1**
**LLPH**	**LLP homolog, long-term synaptic facilitation (Aplysia)**
**LMAN2**	**lectin, mannose-binding 2**
**LMBR1**	**limb development membrane protein 1**
**LMBRD1**	**LMBR1 domain containing 1**
**LMF2**	**lipase maturation factor 2**
**LMNB1**	**lamin B1**
**LMO2**	**LIM domain only 2 (rhombotin-like 1)**
**LNP1**	**leukemia NUP98 fusion partner 1**
**LNPEP**	**leucyl/cystinyl aminopeptidase**
**LOC100128164**	**four and a half LIM domains 1 pseudogene**
**LOC100128573**	**uncharacterized LOC100128573**
**LOC100128682**	
**LOC100129034**	**uncharacterized LOC100129034**
**LOC100129046**	**uncharacterized LOC100129046**
**LOC100129083**	**uncharacterized LOC100129083**
**LOC100129148**	**uncharacterized LOC100129148**
**LOC100129361**	
**LOC100129518**	**uncharacterized LOC100129518**
**LOC100129917**	**uncharacterized LOC100129917**
**LOC100130093**	**uncharacterized LOC100130093**
**LOC100130451**	**uncharacterized LOC100130451**
**LOC100130691**	**uncharacterized LOC100130691**

**LOC100130872**	**uncharacterized LOC100130872**
**LOC100130950**	**uncharacterized LOC100130950**
**LOC100130987**	**uncharacterized LOC100130987**
**LOC100130992**	**uncharacterized LOC100130992**
**LOC100131564**	**uncharacterized LOC100131564**
**LOC100131655**	**uncharacterized LOC100131655**
**LOC100131691**	
**LOC100132352**	**FSHD region gene 1 pseudogene**
**LOC100132356**	**uncharacterized LOC100132356**
**LOC100132891**	
**LOC100133315**	**transient receptor potential cation channel, subfamily C, member 2-like**
**LOC100133985**	**uncharacterized LOC100133985**
**LOC100268168**	**uncharacterized LOC100268168**
**LOC100270746**	**uncharacterized LOC100270746**
**LOC100287015**	**uncharacterized LOC100287015**
**LOC100287036**	**uncharacterized LOC100287036**
**LOC100287042**	**uncharacterized LOC100287042**
**LOC100288069**	**uncharacterized LOC100288069**
**LOC100288842**	**UDP-GlcNAc:betaGal beta-1,3-N-acetylglucosaminyltransferase 5 pseudogene**
**LOC100288846**	**uncharacterized LOC100288846**
**LOC100289230**	**uncharacterized LOC100289230**
**LOC100289361**	**uncharacterized LOC100289361**
**LOC100289495**	**uncharacterized LOC100289495**
**LOC100289511**	**uncharacterized LOC100289511**
**LOC100499194**	**uncharacterized LOC100499194**
**LOC100499489**	**uncharacterized LOC100499489**
**LOC100505549**	**uncharacterized LOC100505549**
**LOC100505622**	**uncharacterized LOC100505622**
**LOC100505658**	**uncharacterized LOC100505658**
**LOC100505666**	**uncharacterized LOC100505666**
**LOC100505812**	
**LOC100506083**	**uncharacterized LOC100506083**
**LOC100506207**	**uncharacterized LOC100506207**
**LOC100506469**	
**LOC100506548**	**uncharacterized LOC100506548**
**LOC100506713**	
**LOC100506746**	**uncharacterized LOC100506746**
**LOC100506801**	**uncharacterized LOC100506801**
**LOC100506804**	**uncharacterized LOC100506804**
**LOC100507053**	**uncharacterized LOC100507053**
**LOC100507156**	**uncharacterized LOC100507156**

LOC100507217	
LOC100507250	uncharacterized LOC100507250
LOC100507346	uncharacterized LOC100507346
LOC100507462	uncharacterized LOC100507462
LOC100507501	
LOC100507557	uncharacterized LOC100507557
LOC100507564	uncharacterized LOC100507564
LOC100507577	uncharacterized LOC100507577
LOC100507600	uncharacterized LOC100507600
LOC100507634	uncharacterized LOC100507634
LOC100630918	
LOC145783	uncharacterized LOC145783
LOC148413	uncharacterized LOC148413
LOC153684	uncharacterized LOC153684
LOC155060	AI894139 pseudogene
LOC256880	uncharacterized LOC256880
LOC257396	uncharacterized LOC257396
LOC283070	
LOC283710	uncharacterized LOC283710
LOC284023	uncharacterized LOC284023
LOC284454	uncharacterized LOC284454
LOC284648	uncharacterized LOC284648
LOC285696	uncharacterized LOC285696
LOC286059	tumor necrosis factor receptor superfamily, member 10d, decoy with truncated death domain pseudogene
LOC286190	
LOC286367	
LOC339803	uncharacterized LOC339803
LOC339807	uncharacterized LOC339807
LOC344967	acyl-CoA thioesterase 7 pseudogene
LOC374443	C-type lectin domain family 2, member D pseudogene
LOC389641	uncharacterized LOC389641
LOC391322	D-dopachrome tautomerase-like
LOC400958	uncharacterized LOC400958
LOC401052	uncharacterized LOC401052
LOC440028	uncharacterized LOC440028
LOC440600	uncharacterized LOC440600
LOC440700	carbonic anhydrase XIV (CA14) pseudogene
LOC63930	uncharacterized LOC63930
LOC643770	uncharacterized LOC643770
LOC644656	uncharacterized LOC644656
LOC644961	

LOC645513	uncharacterized LOC645513
LOC646626	uncharacterized LOC646626
LOC646862	
LOC648987	uncharacterized LOC648987
LOC652276	potassium channel tetramerization domain containing 5 pseudogene
LOC728175	uncharacterized LOC728175
LOC728613	programmed cell death 6 pseudogene
LOC728743	zinc finger protein pseudogene
LOC729603	calcineurin-like EF-hand protein 1 pseudogene
LOC81691	exonuclease NEF-sp
LOC91450	uncharacterized LOC91450
LOC93622	Morf4 family associated protein 1-like 1 pseudogene
LOH12CR1	loss of heterozygosity, 12, chromosomal region 1
LOH12CR2	loss of heterozygosity, 12, chromosomal region 2 (non-protein coding)
LONP1	lon peptidase 1, mitochondrial
LOXL3	lysyl oxidase-like 3
LPAR2	lysophosphatidic acid receptor 2
LPAR5	lysophosphatidic acid receptor 5
LPCAT3	lysophosphatidylcholine acyltransferase 3
LPCAT4	lysophosphatidylcholine acyltransferase 4
LPXN	leupaxin
LRBA	LPS-responsive vesicle trafficking, beach and anchor containing
LRCH1	leucine-rich repeats and calponin homology (CH) domain containing 1
LRCH3	leucine-rich repeats and calponin homology (CH) domain containing 3
LRCH4	leucine-rich repeats and calponin homology (CH) domain containing 4
LRFN4	leucine rich repeat and fibronectin type III domain containing 4
LRIF1	ligand dependent nuclear receptor interacting factor 1
LRIG2	leucine-rich repeats and immunoglobulin-like domains 2
LRMP	lymphoid-restricted membrane protein
LRP10	low density lipoprotein receptor-related protein 10
LRP2BP	LRP2 binding protein
LRP8	low density lipoprotein receptor-related protein 8, apolipoprotein e receptor
LRPAP1	low density lipoprotein receptor-related protein associated protein 1
LRPPRC	leucine-rich pentatricopeptide repeat containing
LRR1	leucine rich repeat protein 1
LRRC20	leucine rich repeat containing 20
LRRC24	leucine rich repeat containing 24
LRRC27	leucine rich repeat containing 27
LRRC28	leucine rich repeat containing 28
LRRC29	leucine rich repeat containing 29
LRRC34	leucine rich repeat containing 34

**LRRC37A3**	**leucine rich repeat containing 37, member A3**
**LRRC3C**	**leucine rich repeat containing 3C**
**LRRC40**	**leucine rich repeat containing 40**
**LRRC41**	**leucine rich repeat containing 41**
**LRRC42**	**leucine rich repeat containing 42**
**LRRC43**	**leucine rich repeat containing 43**
**LRRC56**	**leucine rich repeat containing 56**
**LRRC59**	**leucine rich repeat containing 59**
**LRRC61**	**leucine rich repeat containing 61**
**LRRC73**	**leucine rich repeat containing 73**
**LRRC75A-AS1**	**LRRC75A antisense RNA 1**
**LRRC8B**	**leucine rich repeat containing 8 family, member B**
**LRRFIP2**	**leucine rich repeat (in FLII) interacting protein 2**
**LRRIQ3**	**leucine-rich repeats and IQ motif containing 3**
**LRRN3**	**leucine rich repeat neuronal 3**
**LRSAM1**	**leucine rich repeat and sterile alpha motif containing 1**
**LRWD1**	**leucine-rich repeats and WD repeat domain containing 1**
**LSG1**	**large 60S subunit nuclear export GTPase 1**
**LSM1**	**LSM1, U6 small nuclear RNA associated**
**LSM11**	**LSM11, U7 small nuclear RNA associated**
**LSM12**	**LSM12 homolog (S. cerevisiae)**
**LSM14A**	**LSM14A, SCD6 homolog A (S. cerevisiae)**
**LSM3**	**LSM3 homolog, U6 small nuclear RNA associated (S. cerevisiae)**
**LSM4**	**LSM4 homolog, U6 small nuclear RNA associated (S. cerevisiae)**
**LSM7**	**LSM7 homolog, U6 small nuclear RNA associated (S. cerevisiae)**
**LSP1**	**lymphocyte-specific protein 1**
**LSR**	**lipolysis stimulated lipoprotein receptor**
**LTA4H**	**leukotriene A4 hydrolase**
**LTB4R**	**leukotriene B4 receptor**
**LTB4R2**	**leukotriene B4 receptor 2**
**LTC4S**	**leukotriene C4 synthase**
**LTN1**	**listerin E3 ubiquitin protein ligase 1**
**LTV1**	**LTV1 ribosome biogenesis factor**
**LUC7L**	**LUC7-like (S. cerevisiae)**
**LUC7L2**	**LUC7-like 2 (S. cerevisiae)**
**LUC7L3**	**LUC7-like 3 (S. cerevisiae)**
**LXN**	**latexin**
**LY75**	**lymphocyte antigen 75**
**LY75-CD302**	**LY75-CD302 readthrough**
**LY86**	**lymphocyte antigen 86**
**LY9**	**lymphocyte antigen 9**

LYL1	lymphoblastic leukemia associated hematopoiesis regulator 1
LYN	LYN proto-oncogene, Src family tyrosine kinase
LYPLA2	lysophospholipase II
LYRM1	LYR motif containing 1
LYRM2	LYR motif containing 2
LYRM5	LYR motif containing 5
LYSMD1	LysM, putative peptidoglycan-binding, domain containing 1
LYSMD3	LysM, putative peptidoglycan-binding, domain containing 3
LYST	lysosomal trafficking regulator
LZIC	leucine zipper and CTNNBIP1 domain containing
LZTFL1	leucine zipper transcription factor-like 1
LZTR1	leucine-zipper-like transcription regulator 1
M6PR	mannose-6-phosphate receptor (cation dependent)
MAD1L1	MAD1 mitotic arrest deficient-like 1 (yeast)
MAD2L1	MAD2 mitotic arrest deficient-like 1 (yeast)
MAD2L1BP	MAD2L1 binding protein
MADD	MAP-kinase activating death domain
MAEA	macrophage erythroblast attacher
MAF1	MAF1 homolog (S. cerevisiae)
MAGOH	mago-nashi homolog, proliferation-associated (Drosophila)
MAGOHB	mago-nashi homolog B (Drosophila)
MAK	male germ cell-associated kinase
MAK16	MAK16 homolog (S. cerevisiae)
MALAT1	metastasis associated lung adenocarcinoma transcript 1 (non-protein coding)
MALSU1	mitochondrial assembly of ribosomal large subunit 1
MALT1	MALT1 paracaspase
MAMDC4	MAM domain containing 4
MAML1	mastermind-like 1 (Drosophila)
MAML2	mastermind-like 2 (Drosophila)
MAMSTR	MEF2 activating motif and SAP domain containing transcriptional regulator
MAN1A1	mannosidase, alpha, class 1A, member 1
MAN1B1	mannosidase, alpha, class 1B, member 1
MAN2A2	mannosidase, alpha, class 2A, member 2
MAN2B1	mannosidase, alpha, class 2B, member 1
MAN2C1	mannosidase, alpha, class 2C, member 1
MANBAL	mannosidase, beta A, lysosomal-like
MAP1LC3B	microtubule-associated protein 1 light chain 3 beta
MAP1LC3B2	microtubule-associated protein 1 light chain 3 beta 2
MAP2K1	mitogen-activated protein kinase kinase 1
MAP2K2	mitogen-activated protein kinase kinase 2
MAP3K10	mitogen-activated protein kinase kinase kinase 10

MAP3K11	mitogen-activated protein kinase kinase kinase 11
MAP3K12	mitogen-activated protein kinase kinase kinase 12
MAP3K14	mitogen-activated protein kinase kinase kinase 14
MAP3K5	mitogen-activated protein kinase kinase kinase 5
MAP3K6	mitogen-activated protein kinase kinase kinase 6
MAP3K7	mitogen-activated protein kinase kinase kinase 7
MAP3K8	mitogen-activated protein kinase kinase kinase 8
MAP4	microtubule-associated protein 4
MAP4K1	mitogen-activated protein kinase kinase kinase kinase 1
MAP4K2	mitogen-activated protein kinase kinase kinase kinase 2
MAPK1	mitogen-activated protein kinase 1
MAPK13	mitogen-activated protein kinase 13
MAPK14	mitogen-activated protein kinase 14
MAPK1IP1L	mitogen-activated protein kinase 1 interacting protein 1-like
MAPK6	mitogen-activated protein kinase 6
MAPK7	mitogen-activated protein kinase 7
MAPK8IP3	mitogen-activated protein kinase 8 interacting protein 3
MAPKAP1	mitogen-activated protein kinase associated protein 1
MAPKAPK5	mitogen-activated protein kinase-activated protein kinase 5
MAPKAPK5-AS1	MAPKAPK5 antisense RNA 1
MAPKBP1	mitogen-activated protein kinase binding protein 1
Mar-03	membrane-associated ring finger (C3HC4) 3, E3 ubiquitin protein ligase
Mar-06	membrane-associated ring finger (C3HC4) 6, E3 ubiquitin protein ligase
Mar-07	membrane-associated ring finger (C3HC4) 7, E3 ubiquitin protein ligase
MARCKS	myristoylated alanine-rich protein kinase C substrate
MARCKSL1	MARCKS-like 1
MARS	methionyl-tRNA synthetase
MARVELD2	MARVEL domain containing 2
MAST2	microtubule associated serine/threonine kinase 2
MAST3	microtubule associated serine/threonine kinase 3
MASTL	microtubule associated serine/threonine kinase-like
MAT2A	methionine adenosyltransferase II, alpha
MATR3	matrin 3
MAU2	MAU2 sister chromatid cohesion factor
MAZ	MYC-associated zinc finger protein (purine-binding transcription factor)
MB21D1	Mab-21 domain containing 1
MBD1	methyl-CpG binding domain protein 1
MBD2	methyl-CpG binding domain protein 2
MBD3	methyl-CpG binding domain protein 3
MBD5	methyl-CpG binding domain protein 5
MBD6	methyl-CpG binding domain protein 6

**MBLAC1**	**metallo-beta-lactamase domain containing 1**
**MBLAC2**	**metallo-beta-lactamase domain containing 2**
**MBNL1**	**muscleblind-like splicing regulator 1**
**MBOAT7**	**membrane bound O-acyltransferase domain containing 7**
**MBP**	**myelin basic protein**
**MBTD1**	**mbt domain containing 1**
**MBTPS1**	**membrane-bound transcription factor peptidase, site 1**
**MC1R**	**melanocortin 1 receptor (alpha melanocyte stimulating hormone receptor)**
**MCAT**	**malonyl CoA:ACP acyltransferase (mitochondrial)**
**MCCC1**	**methylcrotonoyl-CoA carboxylase 1 (alpha)**
**MCCC2**	**methylcrotonoyl-CoA carboxylase 2 (beta)**
**MCCD1**	**mitochondrial coiled-coil domain 1**
**MCEE**	**methylmalonyl CoA epimerase**
**MCFD2**	**multiple coagulation factor deficiency 2**
**MCL1**	**myeloid cell leukemia 1**
**MCM2**	**minichromosome maintenance complex component 2**
**MCM3**	**minichromosome maintenance complex component 3**
**MCM3AP**	**minichromosome maintenance complex component 3 associated protein**
**MCM4**	**minichromosome maintenance complex component 4**
**MCM5**	**minichromosome maintenance complex component 5**
**MCM6**	**minichromosome maintenance complex component 6**
**MCM7**	**minichromosome maintenance complex component 7**
**MCM8**	**minichromosome maintenance complex component 8**
**MCMBP**	**minichromosome maintenance complex binding protein**
**MCOLN1**	**mucolipin 1**
**MCOLN2**	**mucolipin 2**
**MCPH1**	**microcephalin 1**
**MCRS1**	**microspherule protein 1**
**MCTS1**	**malignant T cell amplified sequence 1**
**MCTS2P**	**malignant T cell amplified sequence 2, pseudogene**
**MCU**	**mitochondrial calcium uniporter**
**MCUR1**	**mitochondrial calcium uniporter regulator 1**
**MDC1**	**mediator of DNA-damage checkpoint 1**
**MDH1**	**malate dehydrogenase 1, NAD (soluble)**
**MDH1B**	**malate dehydrogenase 1B, NAD (soluble)**
**MDH2**	**malate dehydrogenase 2, NAD (mitochondrial)**
**MDK**	**midkine (neurite growth-promoting factor 2)**
**MDM2**	**MDM2 proto-oncogene, E3 ubiquitin protein ligase**
**MDM4**	**MDM4, p53 regulator**
**MDN1**	**MDN1, midasin homolog (yeast)**
**MDP1**	**magnesium-dependent phosphatase 1**

**MEAF6**	**MYST/Esa1-associated factor 6**
**MED1**	**mediator complex subunit 1**
**MED10**	**mediator complex subunit 10**
**MED11**	**mediator complex subunit 11**
**MED13**	**mediator complex subunit 13**
**MED16**	**mediator complex subunit 16**
**MED17**	**mediator complex subunit 17**
**MED18**	**mediator complex subunit 18**
**MED19**	**mediator complex subunit 19**
**MED20**	**mediator complex subunit 20**
**MED22**	**mediator complex subunit 22**
**MED23**	**mediator complex subunit 23**
**MED26**	**mediator complex subunit 26**
**MED28**	**mediator complex subunit 28**
**MED29**	**mediator complex subunit 29**
**MED31**	**mediator complex subunit 31**
**MED4**	**mediator complex subunit 4**
**MED7**	**mediator complex subunit 7**
**MEF2A**	**myocyte enhancer factor 2A**
**MEF2B**	**myocyte enhancer factor 2B**
**MEF2BNB**	**MEF2B neighbor**
**MEF2BNB-MEF2B**	**MEF2BNB-MEF2B readthrough**
**MEF2D**	**myocyte enhancer factor 2D**
**MEGF8**	**multiple EGF-like-domains 8**
**MEIG1**	**meiosis/spermiogenesis associated 1**
**MEIS2**	**Meis homeobox 2**
**MELK**	**maternal embryonic leucine zipper kinase**
**MEMO1**	**mediator of cell motility 1**
**MEPCE**	**methylphosphate capping enzyme**
**MESDC1**	**mesoderm development candidate 1**
**MESDC2**	**mesoderm development candidate 2**
**METAP1**	**methionyl aminopeptidase 1**
**METAP1D**	**methionyl aminopeptidase type 1D (mitochondrial)**
**METAP2**	**methionyl aminopeptidase 2**
**METTL1**	**methyltransferase like 1**
**METTL10**	**methyltransferase like 10**
**METTL12**	**methyltransferase like 12**
**METTL13**	**methyltransferase like 13**
**METTL15**	**methyltransferase like 15**
**METTL16**	**methyltransferase like 16**
**METTL18**	**methyltransferase like 18**

**METTL21A**	**methyltransferase like 21A**
**METTL21B**	**methyltransferase like 21B**
**METTL23**	**methyltransferase like 23**
**METTL25**	**methyltransferase like 25**
**METTL2B**	**methyltransferase like 2B**
**METTL5**	**methyltransferase like 5**
**METTL6**	**methyltransferase like 6**
**METTL7A**	**methyltransferase like 7A**
**METTL8**	**methyltransferase like 8**
**METTL9**	**methyltransferase like 9**
**MEX3C**	**mex-3 RNA binding family member C**
**MFAP1**	**microfibrillar-associated protein 1**
**MFAP3**	**microfibrillar-associated protein 3**
**MFI2-AS1**	**MFI2 antisense RNA 1**
**MFN1**	**mitofusin 1**
**MFN2**	**mitofusin 2**
**MFNG**	**MFNG O-fucosylpeptide 3-beta-N-acetylglucosaminyltransferase**
**MFSD1**	**major facilitator superfamily domain containing 1**
**MFSD10**	**major facilitator superfamily domain containing 10**
**MFSD11**	**major facilitator superfamily domain containing 11**
**MFSD12**	**major facilitator superfamily domain containing 12**
**MFSD2A**	**major facilitator superfamily domain containing 2A**
**MFSD3**	**major facilitator superfamily domain containing 3**
**MFSD6L**	**major facilitator superfamily domain containing 6-like**
**MFSD8**	**major facilitator superfamily domain containing 8**
**MFSD9**	**major facilitator superfamily domain containing 9**
**MGA**	**MGA, MAX dimerization protein**
**MGAT1**	**mannosyl (alpha-1,3-)-glycoprotein beta-1,2-N-acetylglucosaminyltransferase**
**MGAT2**	**mannosyl (alpha-1,6-)-glycoprotein beta-1,2-N-acetylglucosaminyltransferase**
**MGAT5**	**mannosyl (alpha-1,6-)-glycoprotein beta-1,6-N-acetyl-glucosaminyltransferase**
**MGC16275**	**uncharacterized protein MGC16275**
**MGC45922**	**uncharacterized LOC284365**
**MGC72080**	**MGC72080 pseudogene**
**MGEA5**	**meningioma expressed antigen 5 (hyaluronidase)**
**MGLL**	**monoglyceride lipase**
**MGME1**	**mitochondrial genome maintenance exonuclease 1**
**MGRN1**	**mahogunin ring finger 1, E3 ubiquitin protein ligase**
**MIA-RAB4B**	**MIA-RAB4B readthrough (NMD candidate)**
**MIB1**	**mindbomb E3 ubiquitin protein ligase 1**
**MIB2**	**mindbomb E3 ubiquitin protein ligase 2**
**MICAL1**	**microtubule associated monooxygenase, calponin and LIM domain containing 1**

MICU2	mitochondrial calcium uptake 2
MIDN	midnolin
MIER1	mesoderm induction early response 1, transcriptional regulator
MIF	macrophage migration inhibitory factor (glycosylation-inhibiting factor)
MIF4GD	MIF4G domain containing
MIIP	migration and invasion inhibitory protein
MINA	MYC induced nuclear antigen
MINOS1	mitochondrial inner membrane organizing system 1
MINOS1-NBL1	MINOS1-NBL1 readthrough
MINPP1	multiple inositol-polyphosphate phosphatase 1
MIR103A2	microRNA 103a-2
MIR103B2	microRNA 103b-2
MIR107	microRNA 107
MIR1181	microRNA 1181
MIR1204	microRNA 1204
MIR1207	microRNA 1207
MIR1226	microRNA 1226
MIR1227	microRNA 1227
MIR1229	microRNA 1229
MIR1238	microRNA 1238
MIR1245A	microRNA 1245a
MIR1245B	microRNA 1245b
MIR1247	microRNA 1247
MIR1248	microRNA 1248
MIR1260B	microRNA 1260b
MIR128-1	microRNA 128-1
MIR1281	microRNA 1281
MIR1282	microRNA 1282
MIR1286	microRNA 1286
MIR1292	microRNA 1292
MIR1304	microRNA 1304
MIR132	microRNA 132
MIR141	microRNA 141
MIR142	microRNA 142
MIR146A	microRNA 146a
MIR1470	microRNA 1470
MIR150	microRNA 150
MIR1538	microRNA 1538
MIR1539	microRNA 1539
MIR17	microRNA 17
MIR17HG	miR-17-92 cluster host gene

MIR181A2HG	MIR181A2 host gene
MIR18A	microRNA 18a
MIR1908	microRNA 1908
MIR191	microRNA 191
MIR1913	microRNA 1913
MIR195	microRNA 195
MIR19A	microRNA 19a
MIR19B1	microRNA 19b-1
MIR200C	microRNA 200c
MIR20A	microRNA 20a
MIR21	microRNA 21
MIR212	microRNA 212
MIR22HG	MIR22 host gene
MIR23A	microRNA 23a
MIR24-2	microRNA 24-2
MIR25	microRNA 25
MIR27A	microRNA 27a
MIR2861	microRNA 2861
MIR29A	microRNA 29a
MIR29B1	microRNA 29b-1
MIR3124	microRNA 3124
MIR3138	microRNA 3138
MIR3142	microRNA 3142
MIR3143	microRNA 3143
MIR3150A	microRNA 3150a
MIR3150B	microRNA 3150b
MIR3153	microRNA 3153
MIR3158-1	microRNA 3158-1
MIR3158-2	microRNA 3158-2
MIR3175	microRNA 3175
MIR3177	microRNA 3177
MIR3178	microRNA 3178
MIR3187	microRNA 3187
MIR3188	microRNA 3188
MIR3190	microRNA 3190
MIR3191	microRNA 3191
MIR3199-1	microRNA 3199-1
MIR3199-2	microRNA 3199-2
MIR320A	microRNA 320a
MIR339	microRNA 339
MIR33B	microRNA 33b

**MIR3613**	**microRNA 3613**
**MIR3615**	**microRNA 3615**
**MIR3648-1**	**microRNA 3648-1**
**MIR3651**	**microRNA 3651**
**MIR3652**	**microRNA 3652**
**MIR3655**	**microRNA 3655**
**MIR3656**	**microRNA 3656**
**MIR3659**	**microRNA 3659**
**MIR365A**	**microRNA 365a**
**MIR3661**	**microRNA 3661**
**MIR3662**	**microRNA 3662**
**MIR3665**	**microRNA 3665**
**MIR3678**	**microRNA 3678**
**MIR3680-1**	**microRNA 3680-1**
**MIR3684**	**microRNA 3684**
**MIR3687-1**	**microRNA 3687-1**
**MIR3912**	**microRNA 3912**
**MIR3913-1**	**microRNA 3913-1**
**MIR3913-2**	**microRNA 3913-2**
**MIR3918**	**microRNA 3918**
**MIR3925**	**microRNA 3925**
**MIR3945**	**microRNA 3945**
**MIR3960**	**microRNA 3960**
**MIR425**	**microRNA 425**
**MIR4258**	**microRNA 4258**
**MIR4278**	**microRNA 4278**
**MIR4285**	**microRNA 4285**
**MIR4321**	**microRNA 4321**
**MIR4322**	**microRNA 4322**
**MIR4323**	**microRNA 4323**
**MIR4426**	**microRNA 4426**
**MIR4427**	**microRNA 4427**
**MIR4434**	**microRNA 4434**
**MIR4437**	**microRNA 4437**
**MIR4442**	**microRNA 4442**
**MIR4449**	**microRNA 4449**
**MIR4453**	**microRNA 4453**
**MIR4456**	**microRNA 4456**
**MIR4462**	**microRNA 4462**
**MIR4467**	**microRNA 4467**
**MIR4470**	**microRNA 4470**

**MIR4471**	**microRNA 4471**
**MIR4476**	**microRNA 4476**
**MIR4479**	**microRNA 4479**
**MIR4482**	**microRNA 4482**
**MIR4491**	**microRNA 4491**
**MIR4493**	**microRNA 4493**
**MIR4505**	**microRNA 4505**
**MIR4513**	**microRNA 4513**
**MIR4519**	**microRNA 4519**
**MIR4521**	**microRNA 4521**
**MIR4522**	**microRNA 4522**
**MIR4523**	**microRNA 4523**
**MIR4526**	**microRNA 4526**
**MIR4530**	**microRNA 4530**
**MIR4532**	**microRNA 4532**
**MIR4632**	**microRNA 4632**
**MIR4638**	**microRNA 4638**
**MIR4645**	**microRNA 4645**
**MIR4647**	**microRNA 4647**
**MIR4649**	**microRNA 4649**
**MIR4651**	**microRNA 4651**
**MIR4658**	**microRNA 4658**
**MIR4659A**	**microRNA 4659a**
**MIR4659B**	**microRNA 4659b**
**MIR4665**	**microRNA 4665**
**MIR4674**	**microRNA 4674**
**MIR4678**	**microRNA 4678**
**MIR4687**	**microRNA 4687**
**MIR4690**	**microRNA 4690**
**MIR4707**	**microRNA 4707**
**MIR4708**	**microRNA 4708**
**MIR4713**	**microRNA 4713**
**MIR4721**	**microRNA 4721**
**MIR4724**	**microRNA 4724**
**MIR4727**	**microRNA 4727**
**MIR4734**	**microRNA 4734**
**MIR4736**	**microRNA 4736**
**MIR4738**	**microRNA 4738**
**MIR4745**	**microRNA 4745**
**MIR4746**	**microRNA 4746**
**MIR4749**	**microRNA 4749**

**MIR4751**	**microRNA 4751**
**MIR4754**	**microRNA 4754**
**MIR4757**	**microRNA 4757**
**MIR4767**	**microRNA 4767**
**MIR486-1**	**microRNA 486-1**
**MIR497**	**microRNA 497**
**MIR497HG**	**mir-497-195 cluster host gene**
**MIR548C**	**microRNA 548c**
**MIR548Z**	**microRNA 548z**
**MIR564**	**microRNA 564**
**MIR567**	**microRNA 567**
**MIR573**	**microRNA 573**
**MIR589**	**microRNA 589**
**MIR600**	**microRNA 600**
**MIR611**	**microRNA 611**
**MIR612**	**microRNA 612**
**MIR623**	**microRNA 623**
**MIR630**	**microRNA 630**
**MIR632**	**microRNA 632**
**MIR636**	**microRNA 636**
**MIR638**	**microRNA 638**
**MIR639**	**microRNA 639**
**MIR641**	**microRNA 641**
**MIR658**	**microRNA 658**
**MIR663A**	**microRNA 663a**
**MIR718**	**microRNA 718**
**MIR759**	**microRNA 759**
**MIR762**	**microRNA 762**
**MIR766**	**microRNA 766**
**MIR92B**	**microRNA 92b**
**MIR933**	**microRNA 933**
**MIRLET7D**	**microRNA let-7d**
**MIRLET7I**	**microRNA let-7i**
**MIS12**	**MIS12 kinetochore complex component**
**MIS18A**	**MIS18 kinetochore protein A**
**MIS18BP1**	**MIS18 binding protein 1**
**MITD1**	**MIT, microtubule interacting and transport, domain containing 1**
**MKKS**	**McKusick-Kaufman syndrome**
**MKL1**	**megakaryoblastic leukemia (translocation) 1**
**MKLN1**	**muskelin 1, intracellular mediator containing kelch motifs**
**MKNK1**	**MAP kinase interacting serine/threonine kinase 1**

**MKNK2**	**MAP kinase interacting serine/threonine kinase 2**
**MKRN2**	**makorin ring finger protein 2**
**MKS1**	**Meckel syndrome, type 1**
**MLEC**	**malectin**
**MLF2**	**myeloid leukemia factor 2**
**MLH1**	**mutL homolog 1**
**MLH3**	**mutL homolog 3**
**MLLT1**	**myeloid/lymphoid or mixed-lineage leukemia (trithorax homolog, Drosophila); translocated to, 1**
**MLLT11**	**myeloid/lymphoid or mixed-lineage leukemia (trithorax homolog, Drosophila); translocated to, 11**
**MLLT6**	**myeloid/lymphoid or mixed-lineage leukemia (trithorax homolog, Drosophila); translocated to, 6**
**MLST8**	**MTOR associated protein, LST8 homolog (S. cerevisiae)**
**MLX**	**MLX, MAX dimerization protein**
**MMAA**	**methylmalonic aciduria (cobalamin deficiency) cblA type**
**MMAB**	**methylmalonic aciduria (cobalamin deficiency) cblB type**
**MMADHC**	**methylmalonic aciduria (cobalamin deficiency) cblD type, with homocystinuria**
**MMP23B**	**matrix metallopeptidase 23B**
**MMS19**	**MMS19 nucleotide excision repair homolog (S. cerevisiae)**
**MMS22L**	**MMS22-like, DNA repair protein**
**MNAT1**	**MNAT CDK-activating kinase assembly factor 1**
**MNT**	**MAX network transcriptional repressor**
**MOB1A**	**MOB kinase activator 1A**
**MOB3A**	**MOB kinase activator 3A**
**MOB4**	**MOB family member 4, phocein**
**MOCS2**	**molybdenum cofactor synthesis 2**
**MOGAT2**	**monoacylglycerol O-acyltransferase 2**
**MOGS**	**mannosyl-oligosaccharide glucosidase**
**MON1A**	**MON1 secretory trafficking family member A**
**MON1B**	**MON1 secretory trafficking family member B**
**MON2**	**MON2 homolog (S. cerevisiae)**
**MORC3**	**MORC family CW-type zinc finger 3**
**MORF4L1**	**mortality factor 4 like 1**
**MORF4L2**	**mortality factor 4 like 2**
**MOV10**	**Mov10 RISC complex RNA helicase**
**MPC1**	**mitochondrial pyruvate carrier 1**
**MPC2**	**mitochondrial pyruvate carrier 2**
**MPDU1**	**mannose-P-dolichol utilization defect 1**
**MPEG1**	**macrophage expressed 1**
**MPHOSPH10**	**M-phase phosphoprotein 10 (U3 small nucleolar ribonucleoprotein)**
**MPI**	**mannose phosphate isomerase**

**MPLKIP**	**M-phase specific PLK1 interacting protein**
**MPND**	**MPN domain containing**
**MPP6**	**membrane protein, palmitoylated 6 (MAGUK p55 subfamily member 6)**
**MPPE1**	**metallophosphoesterase 1**
**MPV17**	**MpV17 mitochondrial inner membrane protein**
**MPV17L2**	**MPV17 mitochondrial membrane protein-like 2**
**MPZL3**	**myelin protein zero-like 3**
**MREG**	**melanoregulin**
**MRFAP1**	**Morf4 family associated protein 1**
**MRFAP1L1**	**Morf4 family associated protein 1-like 1**
**MRI1**	**methylthioribose-1-phosphate isomerase 1**
**MRM1**	**mitochondrial rRNA methyltransferase 1 homolog (S. cerevisiae)**
**MROH6**	**maestro heat-like repeat family member 6**
**MROH8**	**maestro heat-like repeat family member 8**
**MRPL1**	**mitochondrial ribosomal protein L1**
**MRPL12**	**mitochondrial ribosomal protein L12**
**MRPL13**	**mitochondrial ribosomal protein L13**
**MRPL14**	**mitochondrial ribosomal protein L14**
**MRPL15**	**mitochondrial ribosomal protein L15**
**MRPL17**	**mitochondrial ribosomal protein L17**
**MRPL18**	**mitochondrial ribosomal protein L18**
**MRPL19**	**mitochondrial ribosomal protein L19**
**MRPL2**	**mitochondrial ribosomal protein L2**
**MRPL20**	**mitochondrial ribosomal protein L20**
**MRPL21**	**mitochondrial ribosomal protein L21**
**MRPL24**	**mitochondrial ribosomal protein L24**
**MRPL27**	**mitochondrial ribosomal protein L27**
**MRPL28**	**mitochondrial ribosomal protein L28**
**MRPL3**	**mitochondrial ribosomal protein L3**
**MRPL30**	**mitochondrial ribosomal protein L30**
**MRPL32**	**mitochondrial ribosomal protein L32**
**MRPL34**	**mitochondrial ribosomal protein L34**
**MRPL35**	**mitochondrial ribosomal protein L35**
**MRPL36**	**mitochondrial ribosomal protein L36**
**MRPL38**	**mitochondrial ribosomal protein L38**
**MRPL39**	**mitochondrial ribosomal protein L39**
**MRPL4**	**mitochondrial ribosomal protein L4**
**MRPL40**	**mitochondrial ribosomal protein L40**
**MRPL41**	**mitochondrial ribosomal protein L41**
**MRPL42**	**mitochondrial ribosomal protein L42**
**MRPL44**	**mitochondrial ribosomal protein L44**

**MRPL45**	**mitochondrial ribosomal protein L45**
**MRPL46**	**mitochondrial ribosomal protein L46**
**MRPL47**	**mitochondrial ribosomal protein L47**
**MRPL48**	**mitochondrial ribosomal protein L48**
**MRPL49**	**mitochondrial ribosomal protein L49**
**MRPL51**	**mitochondrial ribosomal protein L51**
**MRPL53**	**mitochondrial ribosomal protein L53**
**MRPL54**	**mitochondrial ribosomal protein L54**
**MRPL55**	**mitochondrial ribosomal protein L55**
**MRPL57**	**mitochondrial ribosomal protein L57**
**MRPL9**	**mitochondrial ribosomal protein L9**
**MRPS10**	**mitochondrial ribosomal protein S10**
**MRPS11**	**mitochondrial ribosomal protein S11**
**MRPS12**	**mitochondrial ribosomal protein S12**
**MRPS14**	**mitochondrial ribosomal protein S14**
**MRPS15**	**mitochondrial ribosomal protein S15**
**MRPS16**	**mitochondrial ribosomal protein S16**
**MRPS17**	**mitochondrial ribosomal protein S17**
**MRPS18C**	**mitochondrial ribosomal protein S18C**
**MRPS21**	**mitochondrial ribosomal protein S21**
**MRPS23**	**mitochondrial ribosomal protein S23**
**MRPS24**	**mitochondrial ribosomal protein S24**
**MRPS25**	**mitochondrial ribosomal protein S25**
**MRPS26**	**mitochondrial ribosomal protein S26**
**MRPS27**	**mitochondrial ribosomal protein S27**
**MRPS28**	**mitochondrial ribosomal protein S28**
**MRPS31**	**mitochondrial ribosomal protein S31**
**MRPS33**	**mitochondrial ribosomal protein S33**
**MRPS34**	**mitochondrial ribosomal protein S34**
**MRPS35**	**mitochondrial ribosomal protein S35**
**MRPS36**	**mitochondrial ribosomal protein S36**
**MRPS5**	**mitochondrial ribosomal protein S5**
**MRPS6**	**mitochondrial ribosomal protein S6**
**MRPS7**	**mitochondrial ribosomal protein S7**
**MRRF**	**mitochondrial ribosome recycling factor**
**MRTO4**	**mRNA turnover 4 homolog (S. cerevisiae)**
**MRVI1-AS1**	**MRVI1 antisense RNA 1**
**MS4A1**	**membrane-spanning 4-domains, subfamily A, member 1**
**MS4A10**	**membrane-spanning 4-domains, subfamily A, member 10**
**MS4A6A**	**membrane-spanning 4-domains, subfamily A, member 6A**
**MS4A7**	**membrane-spanning 4-domains, subfamily A, member 7**

MSC	musculin
MSH2	mutS homolog 2
MSH3	mutS homolog 3
MSH4	mutS homolog 4
MSH6	mutS homolog 6
MSL1	male-specific lethal 1 homolog (Drosophila)
MSL2	male-specific lethal 2 homolog (Drosophila)
MSLNL	mesothelin-like
MSMO1	methylsterol monooxygenase 1
MSRB1	methionine sulfoxide reductase B1
MST1	macrophage stimulating 1
MST1P2	macrophage stimulating 1 (hepatocyte growth factor-like) pseudogene 2
MSTO2P	misato family member 2, pseudogene
MTA2	metastasis associated 1 family, member 2
MTBP	MDM2 binding protein
MTCH1	mitochondrial carrier 1
MTDH	metadherin
MTERF1	mitochondrial transcription termination factor 1
MTERF3	mitochondrial transcription termination factor 3
MTERF4	mitochondrial transcription termination factor 4
MTF1	metal-regulatory transcription factor 1
MTF2	metal response element binding transcription factor 2
MTFR1L	mitochondrial fission regulator 1-like
MTFR2	mitochondrial fission regulator 2
MTG1	mitochondrial ribosome-associated GTPase 1
MTG2	mitochondrial ribosome-associated GTPase 2
MTHFD1	methylenetetrahydrofolate dehydrogenase (NADP+ dependent) 1, methenyltetrahydrofolate cyclohydrolase, formyltetrahydrofolate synthetase
MTHFD2	methylenetetrahydrofolate dehydrogenase (NADP+ dependent) 2, methenyltetrahydrofolate cyclohydrolase
MTHFS	5,10-methenyltetrahydrofolate synthetase (5-formyltetrahydrofolate cyclo-ligase)
MTIF2	mitochondrial translational initiation factor 2
MTIF3	mitochondrial translational initiation factor 3
MTMR14	myotubularin related protein 14
MTMR2	myotubularin related protein 2
MTMR4	myotubularin related protein 4
MTMR6	myotubularin related protein 6
MTMR9	myotubularin related protein 9
MTO1	mitochondrial tRNA translation optimization 1
MTOR	mechanistic target of rapamycin (serine/threonine kinase)
MTR	5-methyltetrahydrofolate-homocysteine methyltransferase
MTRF1	mitochondrial translational release factor 1

**MTRF1L**	**mitochondrial translational release factor 1-like**
**MTTP**	**microsomal triglyceride transfer protein**
**MTVR2**	**mouse mammary tumor virus receptor homolog 2**
**MTX2**	**metaxin 2**
**MTX3**	**metaxin 3**
**MUC1**	**mucin 1, cell surface associated**
**MUS81**	**MUS81 structure-specific endonuclease subunit**
**MUT**	**methylmalonyl CoA mutase**
**MUTYH**	**mutY homolog**
**MVB12A**	**multivesicular body subunit 12A**
**MVK**	**mevalonate kinase**
**MX2**	**MX dynamin-like GTPase 2**
**MXD1**	**MAX dimerization protein 1**
**MXD3**	**MAX dimerization protein 3**
**MYB**	**v-myb avian myeloblastosis viral oncogene homolog**
**MYBBP1A**	**MYB binding protein (P160) 1a**
**MYBL1**	**v-myb avian myeloblastosis viral oncogene homolog-like 1**
**MYBPC2**	**myosin binding protein C, fast type**
**MYC**	**v-myc avian myelocytomatosis viral oncogene homolog**
**MYCBP**	**MYC binding protein**
**MYCBP2**	**MYC binding protein 2, E3 ubiquitin protein ligase**
**MYD88**	**myeloid differentiation primary response 88**
**MYH7B**	**myosin, heavy chain 7B, cardiac muscle, beta**
**MYH9**	**myosin, heavy chain 9, non-muscle**
**MYL12A**	**myosin, light chain 12A, regulatory, non-sarcomeric**
**MYL12B**	**myosin, light chain 12B, regulatory**
**MYL5**	**myosin, light chain 5, regulatory**
**MYL6**	**myosin, light chain 6, alkali, smooth muscle and non-muscle**
**MYL6B**	**myosin, light chain 6B, alkali, smooth muscle and non-muscle**
**MYLK-AS1**	**MYLK antisense RNA 1**
**MYNN**	**myoneurin**
**MYO15A**	**myosin XVA**
**MYO19**	**myosin XIX**
**MYO1D**	**myosin ID**
**MYO1G**	**myosin IG**
**MYO9A**	**myosin IXA**
**MYO9B**	**myosin IXB**
**MZB1**	**marginal zone B and B1 cell-specific protein**
**MZF1**	**myeloid zinc finger 1**
**MZT1**	**mitotic spindle organizing protein 1**
**MZT2B**	**mitotic spindle organizing protein 2B**

**N4BP1**	**NEDD4 binding protein 1**
**N4BP2**	**NEDD4 binding protein 2**
**N4BP2L1**	**NEDD4 binding protein 2-like 1**
**N4BP2L2**	**NEDD4 binding protein 2-like 2**
**N4BP3**	**NEDD4 binding protein 3**
**N6AMT2**	**N-6 adenine-specific DNA methyltransferase 2 (putative)**
**NAA15**	**N(alpha)-acetyltransferase 15, NatA auxiliary subunit**
**NAA16**	**N(alpha)-acetyltransferase 16, NatA auxiliary subunit**
**NAA38**	**N(alpha)-acetyltransferase 38, NatC auxiliary subunit**
**NAA50**	**N(alpha)-acetyltransferase 50, NatE catalytic subunit**
**NAB2**	**NGFI-A binding protein 2 (EGR1 binding protein 2)**
**NABP1**	**nucleic acid binding protein 1**
**NACA**	**nascent polypeptide-associated complex alpha subunit**
**NACC1**	**nucleus accumbens associated 1, BEN and BTB (POZ) domain containing**
**NADK**	**NAD kinase**
**NAGK**	**N-acetylglucosamine kinase**
**NAGLU**	**N-acetylglucosaminidase, alpha**
**NAIF1**	**nuclear apoptosis inducing factor 1**
**NAMPT**	**nicotinamide phosphoribosyltransferase**
**NANOS1**	**nanos homolog 1 (Drosophila)**
**NANP**	**N-acetylneuraminic acid phosphatase**
**NANS**	**N-acetylneuraminic acid synthase**
**NAP1L1**	**nucleosome assembly protein 1-like 1**
**NAPA**	**N-ethylmaleimide-sensitive factor attachment protein, alpha**
**NAPEPLD**	**N-acyl phosphatidylethanolamine phospholipase D**
**NAPRT**	**nicotinate phosphoribosyltransferase**
**NAPSB**	**napsin B aspartic peptidase, pseudogene**
**NARF**	**nuclear prelamin A recognition factor**
**NARS**	**asparaginyl-tRNA synthetase**
**NASP**	**nuclear autoantigenic sperm protein (histone-binding)**
**NAT10**	**N-acetyltransferase 10 (GCN5-related)**
**NAT14**	**N-acetyltransferase 14 (GCN5-related, putative)**
**NAT6**	**N-acetyltransferase 6 (GCN5-related)**
**NBN**	**nibrin**
**NBPF1**	**neuroblastoma breakpoint family, member 1**
**NBR1**	**neighbor of BRCA1 gene 1**
**NBR2**	**neighbor of BRCA1 gene 2 (non-protein coding)**
**NCALD**	**neurocalcin delta**
**NCAPD2**	**non-SMC condensin I complex, subunit D2**
**NCAPD3**	**non-SMC condensin II complex, subunit D3**
**NCAPG**	**non-SMC condensin I complex, subunit G**

NCAPG2	non-SMC condensin II complex, subunit G2
NCAPH2	non-SMC condensin II complex, subunit H2
NCBP1	nuclear cap binding protein subunit 1, 80kDa
NCBP2	nuclear cap binding protein subunit 2, 20kDa
NCDN	neurochondrin
NCF2	neutrophil cytosolic factor 2
NCF4	neutrophil cytosolic factor 4, 40kDa
NCK1	NCK adaptor protein 1
NCK2	NCK adaptor protein 2
NCKAP1L	NCK-associated protein 1-like
NCKIPSD	NCK interacting protein with SH3 domain
NCL	nucleolin
NCLN	nicalin
NCMAP	noncompact myelin associated protein
NCOA2	nuclear receptor coactivator 2
NCOA3	nuclear receptor coactivator 3
NCOA5	nuclear receptor coactivator 5
NCOA7	nuclear receptor coactivator 7
NCOR1	nuclear receptor corepressor 1
NCOR2	nuclear receptor corepressor 2
NCR2	natural cytotoxicity triggering receptor 2
NCRUPAR	non-protein coding RNA, upstream of F2R/PAR1
NCSTN	nicastrin
NDC1	NDC1 transmembrane nucleoporin
NDFIP1	Nedd4 family interacting protein 1
NDFIP2	Nedd4 family interacting protein 2
NDNL2	necdin-like 2
NDOR1	NADPH dependent diflavin oxidoreductase 1
NDST2	N-deacetylase/N-sulfotransferase (heparan glucosaminyl) 2
NDUFA10	NADH dehydrogenase (ubiquinone) 1 alpha subcomplex, 10, 42kDa
NDUFA11	NADH dehydrogenase (ubiquinone) 1 alpha subcomplex, 11, 14.7kDa
NDUFA12	NADH dehydrogenase (ubiquinone) 1 alpha subcomplex, 12
NDUFA13	NADH dehydrogenase (ubiquinone) 1 alpha subcomplex, 13
NDUFA2	NADH dehydrogenase (ubiquinone) 1 alpha subcomplex, 2, 8kDa
NDUFA4	NDUFA4, mitochondrial complex associated
NDUFA4L2	NADH dehydrogenase (ubiquinone) 1 alpha subcomplex, 4-like 2
NDUFA5	NADH dehydrogenase (ubiquinone) 1 alpha subcomplex, 5
NDUFA6	NADH dehydrogenase (ubiquinone) 1 alpha subcomplex, 6, 14kDa
NDUFA7	NADH dehydrogenase (ubiquinone) 1 alpha subcomplex, 7, 14.5kDa
NDUFA9	NADH dehydrogenase (ubiquinone) 1 alpha subcomplex, 9, 39kDa
NDUFAB1	NADH dehydrogenase (ubiquinone) 1, alpha/beta subcomplex, 1, 8kDa

**NDUFAF1**	**NADH dehydrogenase (ubiquinone) complex I, assembly factor 1**
**NDUFAF3**	**NADH dehydrogenase (ubiquinone) complex I, assembly factor 3**
**NDUFAF4**	**NADH dehydrogenase (ubiquinone) complex I, assembly factor 4**
**NDUFAF5**	**NADH dehydrogenase (ubiquinone) complex I, assembly factor 5**
**NDUFAF6**	**NADH dehydrogenase (ubiquinone) complex I, assembly factor 6**
**NDUFAF7**	**NADH dehydrogenase (ubiquinone) complex I, assembly factor 7**
**NDUFB1**	**NADH dehydrogenase (ubiquinone) 1 beta subcomplex, 1, 7kDa**
**NDUFB10**	**NADH dehydrogenase (ubiquinone) 1 beta subcomplex, 10, 22kDa**
**NDUFB2**	**NADH dehydrogenase (ubiquinone) 1 beta subcomplex, 2, 8kDa**
**NDUFB3**	**NADH dehydrogenase (ubiquinone) 1 beta subcomplex, 3, 12kDa**
**NDUFB5**	**NADH dehydrogenase (ubiquinone) 1 beta subcomplex, 5, 16kDa**
**NDUFB6**	**NADH dehydrogenase (ubiquinone) 1 beta subcomplex, 6, 17kDa**
**NDUFB7**	**NADH dehydrogenase (ubiquinone) 1 beta subcomplex, 7, 18kDa**
**NDUFB9**	**NADH dehydrogenase (ubiquinone) 1 beta subcomplex, 9, 22kDa**
**NDUFC1**	**NADH dehydrogenase (ubiquinone) 1, subcomplex unknown, 1, 6kDa**
**NDUFC2**	**NADH dehydrogenase (ubiquinone) 1, subcomplex unknown, 2, 14.5kDa**
**NDUFC2-KCTD14**	**NDUFC2-KCTD14 readthrough**
**NDUFS1**	**NADH dehydrogenase (ubiquinone) Fe-S protein 1, 75kDa (NADH-coenzyme Q reductase)**
**NDUFS3**	**NADH dehydrogenase (ubiquinone) Fe-S protein 3, 30kDa (NADH-coenzyme Q reductase)**
**NDUFS5**	**NADH dehydrogenase (ubiquinone) Fe-S protein 5, 15kDa (NADH-coenzyme Q reductase)**
**NDUFS6**	**NADH dehydrogenase (ubiquinone) Fe-S protein 6, 13kDa (NADH-coenzyme Q reductase)**
**NDUFS7**	**NADH dehydrogenase (ubiquinone) Fe-S protein 7, 20kDa (NADH-coenzyme Q reductase)**
**NDUFS8**	**NADH dehydrogenase (ubiquinone) Fe-S protein 8, 23kDa (NADH-coenzyme Q reductase)**
**NDUFV3**	**NADH dehydrogenase (ubiquinone) flavoprotein 3, 10kDa**
**NEAT1**	**nuclear paraspeckle assembly transcript 1 (non-protein coding)**
**NECAB3**	**N-terminal EF-hand calcium binding protein 3**
**NECAP1**	**NECAP endocytosis associated 1**
**NECAP2**	**NECAP endocytosis associated 2**
**NEDD1**	**neural precursor cell expressed, developmentally down-regulated 1**
**NEDD8**	**neural precursor cell expressed, developmentally down-regulated 8**
**NEDD8-MDP1**	**NEDD8-MDP1 readthrough**
**NEDD9**	**neural precursor cell expressed, developmentally down-regulated 9**
**NEIL1**	**nei endonuclease VIII-like 1 (E. coli)**
**NEIL3**	**nei endonuclease VIII-like 3 (E. coli)**
**NEK1**	**NIMA-related kinase 1**
**NEK10**	**NIMA-related kinase 10**
**NEK4**	**NIMA-related kinase 4**

NEK6	NIMA-related kinase 6
NEK8	NIMA-related kinase 8
NEK9	NIMA-related kinase 9
NELFB	negative elongation factor complex member B
NEMF	nuclear export mediator factor
NENF	neudesin neurotrophic factor
NEU1	sialidase 1 (lysosomal sialidase)
NEU3	sialidase 3 (membrane sialidase)
NEURL2	neuralized E3 ubiquitin protein ligase 2
NEUROG2	neurogenin 2
NFAT5	nuclear factor of activated T-cells 5, tonicity-responsive
NFATC3	nuclear factor of activated T-cells, cytoplasmic, calcineurin-dependent 3
NFATC4	nuclear factor of activated T-cells, cytoplasmic, calcineurin-dependent 4
NFE2L1	nuclear factor, erythroid 2-like 1
NFE2L2	nuclear factor, erythroid 2-like 2
NFKB1	nuclear factor of kappa light polypeptide gene enhancer in B-cells 1
NFKB2	nuclear factor of kappa light polypeptide gene enhancer in B-cells 2 (p49/p100)
NFKBIA	nuclear factor of kappa light polypeptide gene enhancer in B-cells inhibitor, alpha
NFKBID	nuclear factor of kappa light polypeptide gene enhancer in B-cells inhibitor, delta
NFKBIE	nuclear factor of kappa light polypeptide gene enhancer in B-cells inhibitor, epsilon
NFKBIL1	nuclear factor of kappa light polypeptide gene enhancer in B-cells inhibitor-like 1
NFKBIZ	nuclear factor of kappa light polypeptide gene enhancer in B-cells inhibitor, zeta
NFS1	NFS1 cysteine desulfurase
NFU1	NFU1 iron-sulfur cluster scaffold
NFX1	nuclear transcription factor, X-box binding 1
NFYA	nuclear transcription factor Y, alpha
NFYC	nuclear transcription factor Y, gamma
NGLY1	N-glycanase 1
NGRN	neugrin, neurite outgrowth associated
NHEJ1	nonhomologous end-joining factor 1
NHLRC2	NHL repeat containing 2
NHLRC3	NHL repeat containing 3
NHP2	NHP2 ribonucleoprotein
NHP2L1	NHP2 non-histone chromosome protein 2-like 1 (S. cerevisiae)
NICN1	nicolin 1
NID1	nidogen 1
NIF3L1	NIF3 NGG1 interacting factor 3-like 1 (S. cerevisiae)
NINJ1	ninjurin 1
NIP7	NIP7, nucleolar pre-rRNA processing protein
NIPA2	non imprinted in Prader-Willi/Angelman syndrome 2
NIPAL1	NIPA-like domain containing 1

NIPBL	Nipped-B homolog (Drosophila)
NIPSNAP3A	nipsnap homolog 3A (C. elegans)
NIT1	nitrilase 1
NKAPP1	NFKB activating protein pseudogene 1
NKG7	natural killer cell granule protein 7
NKIRAS1	NFKB inhibitor interacting Ras-like 1
NKIRAS2	NFKB inhibitor interacting Ras-like 2
NKRF	NFKB repressing factor
NKTR	natural killer cell triggering receptor
NLE1	notchless homolog 1 (Drosophila)
NLGN3	neuroligin 3
NLRC3	NLR family, CARD domain containing 3
NLRX1	NLR family member X1
NMD3	NMD3 ribosome export adaptor
NME1	NME/NM23 nucleoside diphosphate kinase 1
NME1-NME2	NME1-NME2 readthrough
NME2	NME/NM23 nucleoside diphosphate kinase 2
NME3	NME/NM23 nucleoside diphosphate kinase 3
NME6	NME/NM23 nucleoside diphosphate kinase 6
NMI	N-myc (and STAT) interactor
NMNAT1	nicotinamide nucleotide adenylyltransferase 1
NMRAL1	NmrA-like family domain containing 1
NMT1	N-myristoyltransferase 1
NNT	nicotinamide nucleotide transhydrogenase
NOA1	nitric oxide associated 1
NOB1	NIN1/RPN12 binding protein 1 homolog (S. cerevisiae)
NOC3L	nucleolar complex associated 3 homolog (S. cerevisiae)
NOC4L	nucleolar complex associated 4 homolog (S. cerevisiae)
NOD2	nucleotide-binding oligomerization domain containing 2
NOL10	nucleolar protein 10
NOL11	nucleolar protein 11
NOL12	nucleolar protein 12
NOL7	nucleolar protein 7, 27kDa
NOL8	nucleolar protein 8
NOL9	nucleolar protein 9
NOLC1	nucleolar and coiled-body phosphoprotein 1
NOM1	nucleolar protein with MIF4G domain 1
NOP10	NOP10 ribonucleoprotein
NOP14	NOP14 nucleolar protein
NOP14-AS1	NOP14 antisense RNA 1
NOP16	NOP16 nucleolar protein

**NOP2**	**NOP2 nucleolar protein**
**NOP56**	**NOP56 ribonucleoprotein**
**NOP58**	**NOP58 ribonucleoprotein**
**NOP9**	**NOP9 nucleolar protein**
**NOSIP**	**nitric oxide synthase interacting protein**
**NOTCH1**	**notch 1**
**NOTCH2NL**	**notch 2 N-terminal like**
**NOVA2**	**neuro-oncological ventral antigen 2**
**NOXA1**	**NADPH oxidase activator 1**
**NOXO1**	**NADPH oxidase organizer 1**
**NPAS4**	**neuronal PAS domain protein 4**
**NPAT**	**nuclear protein, ataxia-telangiectasia locus**
**NPB**	**neuropeptide B**
**NPEPL1**	**aminopeptidase-like 1**
**NPL**	**N-acetylneuraminate pyruvate lyase (dihydrodipicolinate synthase)**
**NPLOC4**	**nuclear protein localization 4 homolog (S. cerevisiae)**
**NPM1**	**nucleophosmin (nucleolar phosphoprotein B23, numatrin)**
**NPM3**	**nucleophosmin/nucleoplasmin 3**
**NPRL2**	**nitrogen permease regulator-like 2 (S. cerevisiae)**
**NR1D2**	**nuclear receptor subfamily 1, group D, member 2**
**NR1H2**	**nuclear receptor subfamily 1, group H, member 2**
**NR1H3**	**nuclear receptor subfamily 1, group H, member 3**
**NR2C1**	**nuclear receptor subfamily 2, group C, member 1**
**NR2C2AP**	**nuclear receptor 2C2-associated protein**
**NR3C1**	**nuclear receptor subfamily 3, group C, member 1 (glucocorticoid receptor)**
**NR4A2**	**nuclear receptor subfamily 4, group A, member 2**
**NR6A1**	**nuclear receptor subfamily 6, group A, member 1**
**NRADDP**	**neurotrophin receptor associated death domain, pseudogene**
**NRAS**	**neuroblastoma RAS viral (v-ras) oncogene homolog**
**NRBF2**	**nuclear receptor binding factor 2**
**NRBP1**	**nuclear receptor binding protein 1**
**NRD1**	**nardilysin (N-arginine dibasic convertase)**
**NRF1**	**nuclear respiratory factor 1**
**NRM**	**nurim (nuclear envelope membrane protein)**
**NRROS**	**negative regulator of reactive oxygen species**
**NRXN3**	**neurexin 3**
**NSA2**	**NSA2 ribosome biogenesis homolog (S. cerevisiae)**
**NSD1**	**nuclear receptor binding SET domain protein 1**
**NSFL1C**	**NSFL1 (p97) cofactor (p47)**
**NSL1**	**NSL1, MIS12 kinetochore complex component**
**NSMAF**	**neutral sphingomyelinase (N-SMase) activation associated factor**

**NSMCE1**	**non-SMC element 1 homolog (S. cerevisiae)**
**NSUN2**	**NOP2/Sun RNA methyltransferase family, member 2**
**NSUN3**	**NOP2/Sun domain family, member 3**
**NSUN4**	**NOP2/Sun domain family, member 4**
**NSUN5**	**NOP2/Sun domain family, member 5**
**NSUN6**	**NOP2/Sun domain family, member 6**
**NT5C**	**5', 3'-nucleotidase, cytosolic**
**NT5C2**	**5'-nucleotidase, cytosolic II**
**NT5C3A**	**5'-nucleotidase, cytosolic IIIA**
**NT5DC1**	**5'-nucleotidase domain containing 1**
**NT5DC2**	**5'-nucleotidase domain containing 2**
**NT5DC3**	**5'-nucleotidase domain containing 3**
**NT5E**	**5'-nucleotidase, ecto (CD73)**
**NTM**	**neurotrimin**
**NTRK1**	**neurotrophic tyrosine kinase, receptor, type 1**
**NUBP1**	**nucleotide binding protein 1**
**NUCB2**	**nucleobindin 2**
**NUCKS1**	**nuclear casein kinase and cyclin-dependent kinase substrate 1**
**NUDCD1**	**NudC domain containing 1**
**NUDCD2**	**NudC domain containing 2**
**NUDCD3**	**NudC domain containing 3**
**NUDT1**	**nudix (nucleoside diphosphate linked moiety X)-type motif 1**
**NUDT15**	**nudix (nucleoside diphosphate linked moiety X)-type motif 15**
**NUDT16L1**	**nudix (nucleoside diphosphate linked moiety X)-type motif 16-like 1**
**NUDT17**	**nudix (nucleoside diphosphate linked moiety X)-type motif 17**
**NUDT21**	**nudix (nucleoside diphosphate linked moiety X)-type motif 21**
**NUDT22**	**nudix (nucleoside diphosphate linked moiety X)-type motif 22**
**NUDT3**	**nudix (nucleoside diphosphate linked moiety X)-type motif 3**
**NUDT5**	**nudix (nucleoside diphosphate linked moiety X)-type motif 5**
**NUDT6**	**nudix (nucleoside diphosphate linked moiety X)-type motif 6**
**NUDT8**	**nudix (nucleoside diphosphate linked moiety X)-type motif 8**
**NUDT9**	**nudix (nucleoside diphosphate linked moiety X)-type motif 9**
**NUF2**	**NUF2, NDC80 kinetochore complex component**
**NUFIP1**	**nuclear fragile X mental retardation protein interacting protein 1**
**NUFIP2**	**nuclear fragile X mental retardation protein interacting protein 2**
**NUMBL**	**numb homolog (Drosophila)-like**
**NUP107**	**nucleoporin 107kDa**
**NUP153**	**nucleoporin 153kDa**
**NUP155**	**nucleoporin 155kDa**
**NUP188**	**nucleoporin 188kDa**
**NUP205**	**nucleoporin 205kDa**

**NUP210**	**nucleoporin 210kDa**
**NUP210L**	**nucleoporin 210kDa-like**
**NUP214**	**nucleoporin 214kDa**
**NUP35**	**nucleoporin 35kDa**
**NUP37**	**nucleoporin 37kDa**
**NUP43**	**nucleoporin 43kDa**
**NUP54**	**nucleoporin 54kDa**
**NUP62**	**nucleoporin 62kDa**
**NUP85**	**nucleoporin 85kDa**
**NUP98**	**nucleoporin 98kDa**
**NUPL1**	**nucleoporin like 1**
**NUPL2**	**nucleoporin like 2**
**NUS1**	**nuclear undecaprenyl pyrophosphate synthase 1 homolog (S. cerevisiae)**
**NUSAP1**	**nucleolar and spindle associated protein 1**
**NUTF2**	**nuclear transport factor 2**
**NVL**	**nuclear VCP-like**
**NXF1**	**nuclear RNA export factor 1**
**NXT1**	**nuclear transport factor 2-like export factor 1**
**NXT2**	**nuclear transport factor 2-like export factor 2**
**OARD1**	**O-acyl-ADP-ribose deacylase 1**
**OAS2**	**2'-5'-oligoadenylate synthetase 2, 69/71kDa**
**OAZ1**	**ornithine decarboxylase antizyme 1**
**OAZ2**	**ornithine decarboxylase antizyme 2**
**OAZ3**	**ornithine decarboxylase antizyme 3**
**OBFC1**	**oligonucleotide/oligosaccharide-binding fold containing 1**
**OCEL1**	**occludin/ELL domain containing 1**
**OCIAD1**	**OCIA domain containing 1**
**OCLN**	**occludin**
**ODC1**	**ornithine decarboxylase 1**
**ODF2**	**outer dense fiber of sperm tails 2**
**ODF3B**	**outer dense fiber of sperm tails 3B**
**OFD1**	**oral-facial-digital syndrome 1**
**OGDH**	**oxoglutarate (alpha-ketoglutarate) dehydrogenase (lipoamide)**
**OGFOD1**	**2-oxoglutarate and iron-dependent oxygenase domain containing 1**
**OGFOD2**	**2-oxoglutarate and iron-dependent oxygenase domain containing 2**
**OGG1**	**8-oxoguanine DNA glycosylase**
**OGT**	**O-linked N-acetylglucosamine (GlcNAc) transferase**
**OIP5**	**Opa interacting protein 5**
**OIP5-AS1**	**OIP5 antisense RNA 1**
**OLA1**	**Obg-like ATPase 1**
**OLFML3**	**olfactomedin-like 3**

**OLMALINC**	**oligodendrocyte maturation-associated long intergenic non-coding RNA**
**OPA1**	**optic atrophy 1 (autosomal dominant)**
**OR2G6**	**olfactory receptor, family 2, subfamily G, member 6**
**OR2L13**	**olfactory receptor, family 2, subfamily L, member 13**
**ORAI1**	**ORAI calcium release-activated calcium modulator 1**
**ORAI3**	**ORAI calcium release-activated calcium modulator 3**
**ORAOV1**	**oral cancer overexpressed 1**
**ORC1**	**origin recognition complex, subunit 1**
**ORC2**	**origin recognition complex, subunit 2**
**ORC3**	**origin recognition complex, subunit 3**
**ORC4**	**origin recognition complex, subunit 4**
**ORC5**	**origin recognition complex, subunit 5**
**ORMDL1**	**ORMDL sphingolipid biosynthesis regulator 1**
**ORMDL2**	**ORMDL sphingolipid biosynthesis regulator 2**
**ORMDL3**	**ORMDL sphingolipid biosynthesis regulator 3**
**OS9**	**osteosarcoma amplified 9, endoplasmic reticulum lectin**
**OSBPL3**	**oxysterol binding protein-like 3**
**OSBPL8**	**oxysterol binding protein-like 8**
**OSGEP**	**O-sialoglycoprotein endopeptidase**
**OSGEPL1**	**O-sialoglycoprotein endopeptidase-like 1**
**OSM**	**oncostatin M**
**OSR2**	**odd-skipped related transciption factor 2**
**OST4**	**oligosaccharyltransferase 4 homolog (S. cerevisiae)**
**OTUB1**	**OTU deubiquitinase, ubiquitin aldehyde binding 1**
**OTUD6B**	**OTU domain containing 6B**
**OTUD7B**	**OTU deubiquitinase 7B**
**OTULIN**	**OTU deubiquitinase with linear linkage specificity**
**OVCA2**	**ovarian tumor suppressor candidate 2**
**OXA1L**	**oxidase (cytochrome c) assembly 1-like**
**OXCT1**	**3-oxoacid CoA transferase 1**
**OXLD1**	**oxidoreductase-like domain containing 1**
**OXNAD1**	**oxidoreductase NAD-binding domain containing 1**
**OXSM**	**3-oxoacyl-ACP synthase, mitochondrial**
**OXSR1**	**oxidative stress responsive 1**
**P2RX2**	**purinergic receptor P2X, ligand gated ion channel, 2**
**P2RY11**	**purinergic receptor P2Y, G-protein coupled, 11**
**P2RY12**	**purinergic receptor P2Y, G-protein coupled, 12**
**P2RY6**	**pyrimidinergic receptor P2Y, G-protein coupled, 6**
**P2RY8**	**purinergic receptor P2Y, G-protein coupled, 8**
**P3H1**	**prolyl 3-hydroxylase 1**
**P3H2**	**prolyl 3-hydroxylase 2**

**P4HA1**	**prolyl 4-hydroxylase, alpha polypeptide I**
**P4HB**	**prolyl 4-hydroxylase, beta polypeptide**
**P4HTM**	**prolyl 4-hydroxylase, transmembrane (endoplasmic reticulum)**
**PA2G4**	**proliferation-associated 2G4, 38kDa**
**PAAF1**	**proteasomal ATPase-associated factor 1**
**PABPC1**	**poly(A) binding protein, cytoplasmic 1**
**PABPC1L**	**poly(A) binding protein, cytoplasmic 1-like**
**PABPC4**	**poly(A) binding protein, cytoplasmic 4 (inducible form)**
**PABPN1**	**poly(A) binding protein, nuclear 1**
**PACS1**	**phosphofurin acidic cluster sorting protein 1**
**PACS2**	**phosphofurin acidic cluster sorting protein 2**
**PACSIN2**	**protein kinase C and casein kinase substrate in neurons 2**
**PAF1**	**Paf1, RNA polymerase II associated factor, homolog (S. cerevisiae)**
**PAFAH1B2**	**platelet-activating factor acetylhydrolase 1b, catalytic subunit 2 (30kDa)**
**PAFAH1B3**	**platelet-activating factor acetylhydrolase 1b, catalytic subunit 3 (29kDa)**
**PAFAH2**	**platelet-activating factor acetylhydrolase 2, 40kDa**
**PAGR1**	**PAXIP1 associated glutamate-rich protein 1**
**PAICS**	**phosphoribosylaminoimidazole carboxylase, phosphoribosylaminoimidazole succinocarboxamide synthetase**
**PAIP2**	**poly(A) binding protein interacting protein 2**
**PAK1IP1**	**PAK1 interacting protein 1**
**PAK2**	**p21 protein (Cdc42/Rac)-activated kinase 2**
**PALB2**	**partner and localizer of BRCA2**
**PALD1**	**phosphatase domain containing, paladin 1**
**PAM16**	**presequence translocase-associated motor 16 homolog (S. cerevisiae)**
**PAN2**	**PAN2 poly(A) specific ribonuclease subunit**
**PAN3**	**PAN3 poly(A) specific ribonuclease subunit**
**PAN3-AS1**	**PAN3 antisense RNA 1**
**PANK1**	**pantothenate kinase 1**
**PANK2**	**pantothenate kinase 2**
**PANK3**	**pantothenate kinase 3**
**PANX1**	**pannexin 1**
**PAPD5**	**PAP associated domain containing 5**
**PAPD7**	**PAP associated domain containing 7**
**PAQR4**	**progestin and adipoQ receptor family member IV**
**PAQR6**	**progestin and adipoQ receptor family member VI**
**PAQR8**	**progestin and adipoQ receptor family member VIII**
**PARD6A**	**par-6 family cell polarity regulator alpha**
**PARK7**	**parkinson protein 7**
**PARL**	**presenilin associated, rhomboid-like**
**PARP15**	**poly (ADP-ribose) polymerase family, member 15**
**PARP2**	**poly (ADP-ribose) polymerase 2**

**PARP3**	**poly (ADP-ribose) polymerase family, member 3**
**PARP9**	**poly (ADP-ribose) polymerase family, member 9**
**PARPBP**	**PARP1 binding protein**
**PARVG**	**parvin, gamma**
**PATL1**	**protein associated with topoisomerase II homolog 1 (yeast)**
**PATZ1**	**POZ (BTB) and AT hook containing zinc finger 1**
**PAX5**	**paired box 5**
**PAXBP1**	**PAX3 and PAX7 binding protein 1**
**PAXIP1**	**PAX interacting (with transcription-activation domain) protein 1**
**PBLD**	**phenazine biosynthesis-like protein domain containing**
**PBRM1**	**polybromo 1**
**PBX2**	**pre-B-cell leukemia homeobox 2**
**PBX4**	**pre-B-cell leukemia homeobox 4**
**PBXIP1**	**pre-B-cell leukemia homeobox interacting protein 1**
**PCBD2**	**pterin-4 alpha-carbinolamine dehydratase/dimerization cofactor of hepatocyte nuclear factor 1 alpha (TCF1) 2**
**PCBP1**	**poly(rC) binding protein 1**
**PCBP1-AS1**	**PCBP1 antisense RNA 1**
**PCBP2**	**poly(rC) binding protein 2**
**PCCB**	**propionyl CoA carboxylase, beta polypeptide**
**PCED1B**	**PC-esterase domain containing 1B**
**PCF11**	**PCF11 cleavage and polyadenylation factor subunit**
**PCGF1**	**polycomb group ring finger 1**
**PCGF2**	**polycomb group ring finger 2**
**PCID2**	**PCI domain containing 2**
**PCIF1**	**PDX1 C-terminal inhibiting factor 1**
**PCK2**	**phosphoenolpyruvate carboxykinase 2 (mitochondrial)**
**PCM1**	**pericentriolar material 1**
**PCMTD1**	**protein-L-isoaspartate (D-aspartate) O-methyltransferase domain containing 1**
**PCNA**	**proliferating cell nuclear antigen**
**PCNA-AS1**	**PCNA antisense RNA 1**
**PCNP**	**PEST proteolytic signal containing nuclear protein**
**PCNT**	**pericentrin**
**PCNXL3**	**pecanex-like 3 (Drosophila)**
**PCNXL4**	**pecanex-like 4 (Drosophila)**
**PCSK7**	**proprotein convertase subtilisin/kexin type 7**
**PCYT1A**	**phosphate cytidylyltransferase 1, choline, alpha**
**PCYT2**	**phosphate cytidylyltransferase 2, ethanolamine**
**PDAP1**	**PDGFA associated protein 1**
**PDCD1**	**programmed cell death 1**
**PDCD10**	**programmed cell death 10**
**PDCD1LG2**	**programmed cell death 1 ligand 2**

**PDCD2L**	**programmed cell death 2-like**
**PDCD4**	**programmed cell death 4 (neoplastic transformation inhibitor)**
**PDCD5**	**programmed cell death 5**
**PDCD6**	**programmed cell death 6**
**PDCD6IP**	**programmed cell death 6 interacting protein**
**PDCL**	**phosducin-like**
**PDDC1**	**Parkinson disease 7 domain containing 1**
**PDE12**	**phosphodiesterase 12**
**PDE1B**	**phosphodiesterase 1B, calmodulin-dependent**
**PDE4A**	**phosphodiesterase 4A, cAMP-specific**
**PDE7A**	**phosphodiesterase 7A**
**PDE8A**	**phosphodiesterase 8A**
**PDE9A**	**phosphodiesterase 9A**
**PDF**	**peptide deformylase (mitochondrial)**
**PDIA4**	**protein disulfide isomerase family A, member 4**
**PDIA6**	**protein disulfide isomerase family A, member 6**
**PDIK1L**	**PDLIM1 interacting kinase 1 like**
**PDS5A**	**PDS5 cohesin associated factor A**
**PDSS2**	**prenyl (decaprenyl) diphosphate synthase, subunit 2**
**PDXDC1**	**pyridoxal-dependent decarboxylase domain containing 1**
**PDXP**	**pyridoxal (pyridoxine, vitamin B6) phosphatase**
**PEA15**	**phosphoprotein enriched in astrocytes 15**
**PEF1**	**penta-EF-hand domain containing 1**
**PEMT**	**phosphatidylethanolamine N-methyltransferase**
**PER1**	**period circadian clock 1**
**PET100**	**PET100 homolog (S. cerevisiae)**
**PET117**	**PET117 homolog (S. cerevisiae)**
**PEX11B**	**peroxisomal biogenesis factor 11 beta**
**PEX12**	**peroxisomal biogenesis factor 12**
**PEX13**	**peroxisomal biogenesis factor 13**
**PEX14**	**peroxisomal biogenesis factor 14**
**PEX16**	**peroxisomal biogenesis factor 16**
**PEX19**	**peroxisomal biogenesis factor 19**
**PEX2**	**peroxisomal biogenesis factor 2**
**PEX3**	**peroxisomal biogenesis factor 3**
**PEX6**	**peroxisomal biogenesis factor 6**
**PFAS**	**phosphoribosylformylglycinamidine synthase**
**PFDN2**	**prefoldin subunit 2**
**PFDN4**	**prefoldin subunit 4**
**PFDN5**	**prefoldin subunit 5**
**PFKL**	**phosphofructokinase, liver**

**PFKM**	**phosphofructokinase, muscle**
**PFN4**	**profilin family, member 4**
**PGAM1**	**phosphoglycerate mutase 1 (brain)**
**PGAM2**	**phosphoglycerate mutase 2 (muscle)**
**PGAM5**	**phosphoglycerate mutase family member 5**
**PGAP1**	**post-GPI attachment to proteins 1**
**PGAP2**	**post-GPI attachment to proteins 2**
**PGBD2**	**piggyBac transposable element derived 2**
**PGBD4**	**piggyBac transposable element derived 4**
**PGD**	**phosphogluconate dehydrogenase**
**PGGT1B**	**protein geranylgeranyltransferase type I, beta subunit**
**PGLS**	**6-phosphogluconolactonase**
**PGM1**	**phosphoglucomutase 1**
**PGM2**	**phosphoglucomutase 2**
**PGP**	**phosphoglycolate phosphatase**
**PGRMC2**	**progesterone receptor membrane component 2**
**PHACTR4**	**phosphatase and actin regulator 4**
**PHAX**	**phosphorylated adaptor for RNA export**
**PHB2**	**prohibitin 2**
**PHC3**	**polyhomeotic homolog 3 (Drosophila)**
**PHF1**	**PHD finger protein 1**
**PHF11**	**PHD finger protein 11**
**PHF12**	**PHD finger protein 12**
**PHF19**	**PHD finger protein 19**
**PHF2**	**PHD finger protein 2**
**PHF21A**	**PHD finger protein 21A**
**PHF23**	**PHD finger protein 23**
**PHF5A**	**PHD finger protein 5A**
**PHF8**	**PHD finger protein 8**
**PHIP**	**pleckstrin homology domain interacting protein**
**PHKG2**	**phosphorylase kinase, gamma 2 (testis)**
**PHLDA1**	**pleckstrin homology-like domain, family A, member 1**
**PHLPP1**	**PH domain and leucine rich repeat protein phosphatase 1**
**PHOSPHO1**	**phosphatase, orphan 1**
**PHPT1**	**phosphohistidine phosphatase 1**
**PHTF1**	**putative homeodomain transcription factor 1**
**PHTF2**	**putative homeodomain transcription factor 2**
**PI4KA**	**phosphatidylinositol 4-kinase, catalytic, alpha**
**PIAS1**	**protein inhibitor of activated STAT, 1**
**PIAS3**	**protein inhibitor of activated STAT, 3**
**PIBF1**	**progesterone immunomodulatory binding factor 1**

**PICALM**	**phosphatidylinositol binding clathrin assembly protein**
**PIF1**	**PIF1 5'-to-3' DNA helicase**
**PIGB**	**phosphatidylinositol glycan anchor biosynthesis, class B**
**PIGC**	**phosphatidylinositol glycan anchor biosynthesis, class C**
**PIGG**	**phosphatidylinositol glycan anchor biosynthesis, class G**
**PIGK**	**phosphatidylinositol glycan anchor biosynthesis, class K**
**PIGL**	**phosphatidylinositol glycan anchor biosynthesis, class L**
**PIGO**	**phosphatidylinositol glycan anchor biosynthesis, class O**
**PIGQ**	**phosphatidylinositol glycan anchor biosynthesis, class Q**
**PIGT**	**phosphatidylinositol glycan anchor biosynthesis, class T**
**PIGU**	**phosphatidylinositol glycan anchor biosynthesis, class U**
**PIGV**	**phosphatidylinositol glycan anchor biosynthesis, class V**
**PIGW**	**phosphatidylinositol glycan anchor biosynthesis, class W**
**PIGX**	**phosphatidylinositol glycan anchor biosynthesis, class X**
**PIH1D1**	**PIH1 domain containing 1**
**PIK3CA**	**phosphatidylinositol-4,5-bisphosphate 3-kinase, catalytic subunit alpha**
**PIK3CD**	**phosphatidylinositol-4,5-bisphosphate 3-kinase, catalytic subunit delta**
**PIK3CD-AS1**	**PIK3CD antisense RNA 1**
**PIK3CG**	**phosphatidylinositol-4,5-bisphosphate 3-kinase, catalytic subunit gamma**
**PIK3IP1**	**phosphoinositide-3-kinase interacting protein 1**
**PIK3R1**	**phosphoinositide-3-kinase, regulatory subunit 1 (alpha)**
**PIK3R2**	**phosphoinositide-3-kinase, regulatory subunit 2 (beta)**
**PIK3R3**	**phosphoinositide-3-kinase, regulatory subunit 3 (gamma)**
**PIK3R5**	**phosphoinositide-3-kinase, regulatory subunit 5**
**PIK3R6**	**phosphoinositide-3-kinase, regulatory subunit 6**
**PIKFYVE**	**phosphoinositide kinase, FYVE finger containing**
**PILRB**	**paired immunoglobin-like type 2 receptor beta**
**PIM2**	**Pim-2 proto-oncogene, serine/threonine kinase**
**PIM3**	**Pim-3 proto-oncogene, serine/threonine kinase**
**PIN1**	**peptidylprolyl cis/trans isomerase, NIMA-interacting 1**
**PINX1**	**PIN2/TERF1 interacting, telomerase inhibitor 1**
**PIP4K2B**	**phosphatidylinositol-5-phosphate 4-kinase, type II, beta**
**PIP4K2C**	**phosphatidylinositol-5-phosphate 4-kinase, type II, gamma**
**PIP5K1A**	**phosphatidylinositol-4-phosphate 5-kinase, type I, alpha**
**PIP5K1B**	**phosphatidylinositol-4-phosphate 5-kinase, type I, beta**
**PIP5KL1**	**phosphatidylinositol-4-phosphate 5-kinase-like 1**
**PISD**	**phosphatidylserine decarboxylase**
**PITPNB**	**phosphatidylinositol transfer protein, beta**
**PITPNC1**	**phosphatidylinositol transfer protein, cytoplasmic 1**
**PITPNM1**	**phosphatidylinositol transfer protein, membrane-associated 1**
**PJA2**	**praja ring finger 2, E3 ubiquitin protein ligase**

**PKD1L1**	**polycystic kidney disease 1 like 1**
**PKD2L2**	**polycystic kidney disease 2-like 2**
**PKIB**	**protein kinase (cAMP-dependent, catalytic) inhibitor beta**
**PKM**	**pyruvate kinase, muscle**
**PKN2**	**protein kinase N2**
**PLA1A**	**phospholipase A1 member A**
**PLA2G4F**	**phospholipase A2, group IVF**
**PLA2G6**	**phospholipase A2, group VI (cytosolic, calcium-independent)**
**PLAA**	**phospholipase A2-activating protein**
**PLAG1**	**pleiomorphic adenoma gene 1**
**PLAGL1**	**pleiomorphic adenoma gene-like 1**
**PLAU**	**plasminogen activator, urokinase**
**PLCXD2**	**phosphatidylinositol-specific phospholipase C, X domain containing 2**
**PLD3**	**phospholipase D family, member 3**
**PLD4**	**phospholipase D family, member 4**
**PLD6**	**phospholipase D family, member 6**
**PLEC**	**plectin**
**PLEK**	**pleckstrin**
**PLEKHA1**	**pleckstrin homology domain containing, family A (phosphoinositide binding specific) member 1**
**PLEKHA2**	**pleckstrin homology domain containing, family A (phosphoinositide binding specific) member 2**
**PLEKHA3**	**pleckstrin homology domain containing, family A (phosphoinositide binding specific) member 3**
**PLEKHA8P1**	**pleckstrin homology domain containing, family A member 8 pseudogene 1**
**PLEKHB2**	**pleckstrin homology domain containing, family B (evectins) member 2**
**PLEKHG2**	**pleckstrin homology domain containing, family G (with RhoGef domain) member 2**
**PLEKHG4**	**pleckstrin homology domain containing, family G (with RhoGef domain) member 4**
**PLEKHH3**	**pleckstrin homology domain containing, family H (with MyTH4 domain) member 3**
**PLEKHJ1**	**pleckstrin homology domain containing, family J member 1**
**PLEKHM3**	**pleckstrin homology domain containing, family M, member 3**
**PLEKHO1**	**pleckstrin homology domain containing, family O member 1**
**PLGRKT**	**plasminogen receptor, C-terminal lysine transmembrane protein**
**PLK1**	**polo-like kinase 1**
**PLK3**	**polo-like kinase 3**
**PLK4**	**polo-like kinase 4**
**PLOD1**	**procollagen-lysine, 2-oxoglutarate 5-dioxygenase 1**
**PLRG1**	**pleiotropic regulator 1**
**PLSCR3**	**phospholipid scramblase 3**
**PLXNB2**	**plexin B2**
**PLXNC1**	**plexin C1**
**PMAIP1**	**phorbol-12-myristate-13-acetate-induced protein 1**

**PMCH**	**pro-melanin-concentrating hormone**
**PMF1**	**polyamine-modulated factor 1**
**PMF1-BGLAP**	**PMF1-BGLAP readthrough**
**PMM1**	**phosphomannomutase 1**
**PMPCA**	**peptidase (mitochondrial processing) alpha**
**PMS1**	**PMS1 postmeiotic segregation increased 1 (S. cerevisiae)**
**PMS2**	**PMS2 postmeiotic segregation increased 2 (S. cerevisiae)**
**PMS2P1**	**postmeiotic segregation increased 2 pseudogene 1**
**PMS2P4**	**postmeiotic segregation increased 2 pseudogene 4**
**PMVK**	**phosphomevalonate kinase**
**PNISR**	**PNN-interacting serine/arginine-rich protein**
**PNKD**	**paroxysmal nonkinesigenic dyskinesia**
**PNKP**	**polynucleotide kinase 3'-phosphatase**
**PNMA1**	**paraneoplastic Ma antigen 1**
**PNN**	**pinin, desmosome associated protein**
**PNO1**	**partner of NOB1 homolog (S. cerevisiae)**
**PNP**	**purine nucleoside phosphorylase**
**PNPLA2**	**patatin-like phospholipase domain containing 2**
**PNPLA4**	**patatin-like phospholipase domain containing 4**
**PNPLA6**	**patatin-like phospholipase domain containing 6**
**PNPLA7**	**patatin-like phospholipase domain containing 7**
**PNPLA8**	**patatin-like phospholipase domain containing 8**
**PNPT1**	**polyribonucleotide nucleotidyltransferase 1**
**PNRC1**	**proline-rich nuclear receptor coactivator 1**
**PNRC2**	**proline-rich nuclear receptor coactivator 2**
**POC1A**	**POC1 centriolar protein A**
**POC1B**	**POC1 centriolar protein B**
**POC1B-GALNT4**	**POC1B-GALNT4 readthrough**
**POC5**	**POC5 centriolar protein**
**POGLUT1**	**protein O-glucosyltransferase 1**
**POLA2**	**polymerase (DNA directed), alpha 2, accessory subunit**
**POLB**	**polymerase (DNA directed), beta**
**POLD1**	**polymerase (DNA directed), delta 1, catalytic subunit**
**POLD2**	**polymerase (DNA directed), delta 2, accessory subunit**
**POLD3**	**polymerase (DNA-directed), delta 3, accessory subunit**
**POLD4**	**polymerase (DNA-directed), delta 4, accessory subunit**
**POLDIP3**	**polymerase (DNA-directed), delta interacting protein 3**
**POLE**	**polymerase (DNA directed), epsilon, catalytic subunit**
**POLE2**	**polymerase (DNA directed), epsilon 2, accessory subunit**
**POLE3**	**polymerase (DNA directed), epsilon 3, accessory subunit**
**POLE4**	**polymerase (DNA-directed), epsilon 4, accessory subunit**

**POLG**	**polymerase (DNA directed), gamma**
**POLG2**	**polymerase (DNA directed), gamma 2, accessory subunit**
**POLH**	**polymerase (DNA directed), eta**
**POLI**	**polymerase (DNA directed) iota**
**POLM**	**polymerase (DNA directed), mu**
**POLR1A**	**polymerase (RNA) I polypeptide A, 194kDa**
**POLR1B**	**polymerase (RNA) I polypeptide B, 128kDa**
**POLR1C**	**polymerase (RNA) I polypeptide C, 30kDa**
**POLR2A**	**polymerase (RNA) II (DNA directed) polypeptide A, 220kDa**
**POLR2B**	**polymerase (RNA) II (DNA directed) polypeptide B, 140kDa**
**POLR2C**	**polymerase (RNA) II (DNA directed) polypeptide C, 33kDa**
**POLR2F**	**polymerase (RNA) II (DNA directed) polypeptide F**
**POLR2G**	**polymerase (RNA) II (DNA directed) polypeptide G**
**POLR2H**	**polymerase (RNA) II (DNA directed) polypeptide H**
**POLR2I**	**polymerase (RNA) II (DNA directed) polypeptide I, 14.5kDa**
**POLR2J4**	**polymerase (RNA) II (DNA directed) polypeptide J4, pseudogene**
**POLR2K**	**polymerase (RNA) II (DNA directed) polypeptide K, 7.0kDa**
**POLR2L**	**polymerase (RNA) II (DNA directed) polypeptide L, 7.6kDa**
**POLR3A**	**polymerase (RNA) III (DNA directed) polypeptide A, 155kDa**
**POLR3B**	**polymerase (RNA) III (DNA directed) polypeptide B**
**POLR3C**	**polymerase (RNA) III (DNA directed) polypeptide C (62kD)**
**POLR3D**	**polymerase (RNA) III (DNA directed) polypeptide D, 44kDa**
**POLR3E**	**polymerase (RNA) III (DNA directed) polypeptide E (80kD)**
**POLR3G**	**polymerase (RNA) III (DNA directed) polypeptide G (32kD)**
**POLR3H**	**polymerase (RNA) III (DNA directed) polypeptide H (22.9kD)**
**POLR3K**	**polymerase (RNA) III (DNA directed) polypeptide K, 12.3 kDa**
**POLRMT**	**polymerase (RNA) mitochondrial (DNA directed)**
**POM121C**	**POM121 transmembrane nucleoporin C**
**POMP**	**proteasome maturation protein**
**POMT1**	**protein-O-mannosyltransferase 1**
**PON2**	**paraoxonase 2**
**POP4**	**processing of precursor 4, ribonuclease P/MRP subunit (S. cerevisiae)**
**POP5**	**processing of precursor 5, ribonuclease P/MRP subunit (S. cerevisiae)**
**POP7**	**processing of precursor 7, ribonuclease P/MRP subunit (S. cerevisiae)**
**POR**	**P450 (cytochrome) oxidoreductase**
**POT1**	**protection of telomeres 1**
**POU2AF1**	**POU class 2 associating factor 1**
**POU2F1**	**POU class 2 homeobox 1**
**POU2F2**	**POU class 2 homeobox 2**
**POU2F3**	**POU class 2 homeobox 3**
**PP7080**	**uncharacterized LOC25845**

**PPA2**	**pyrophosphatase (inorganic) 2**
**PPAN**	**peter pan homolog (Drosophila)**
**PPAN-P2RY11**	**PPAN-P2RY11 readthrough**
**PPAPDC1B**	**phosphatidic acid phosphatase type 2 domain containing 1B**
**PPARGC1B**	**peroxisome proliferator-activated receptor gamma, coactivator 1 beta**
**PPAT**	**phosphoribosyl pyrophosphate amidotransferase**
**PPCDC**	**phosphopantothenoylcysteine decarboxylase**
**PPCS**	**phosphopantothenoylcysteine synthetase**
**PPFIA3**	**protein tyrosine phosphatase, receptor type, f polypeptide (PTPRF), interacting protein (liprin), alpha 3**
**PPFIBP2**	**PTPRF interacting protein, binding protein 2 (liprin beta 2)**
**PPIA**	**peptidylprolyl isomerase A (cyclophilin A)**
**PPIB**	**peptidylprolyl isomerase B (cyclophilin B)**
**PPID**	**peptidylprolyl isomerase D**
**PPIG**	**peptidylprolyl isomerase G (cyclophilin G)**
**PPIH**	**peptidylprolyl isomerase H (cyclophilin H)**
**PPIL2**	**peptidylprolyl isomerase (cyclophilin)-like 2**
**PPIL3**	**peptidylprolyl isomerase (cyclophilin)-like 3**
**PPIL4**	**peptidylprolyl isomerase (cyclophilin)-like 4**
**PPM1B**	**protein phosphatase, Mg2+/Mn2+ dependent, 1B**
**PPM1D**	**protein phosphatase, Mg2+/Mn2+ dependent, 1D**
**PPM1G**	**protein phosphatase, Mg2+/Mn2+ dependent, 1G**
**PPM1K**	**protein phosphatase, Mg2+/Mn2+ dependent, 1K**
**PPM1M**	**protein phosphatase, Mg2+/Mn2+ dependent, 1M**
**PPME1**	**protein phosphatase methylesterase 1**
**PPOX**	**protoporphyrinogen oxidase**
**PPP1CA**	**protein phosphatase 1, catalytic subunit, alpha isozyme**
**PPP1CB**	**protein phosphatase 1, catalytic subunit, beta isozyme**
**PPP1R12A**	**protein phosphatase 1, regulatory subunit 12A**
**PPP1R14B**	**protein phosphatase 1, regulatory (inhibitor) subunit 14B**
**PPP1R15A**	**protein phosphatase 1, regulatory subunit 15A**
**PPP1R15B**	**protein phosphatase 1, regulatory subunit 15B**
**PPP1R16B**	**protein phosphatase 1, regulatory subunit 16B**
**PPP1R2**	**protein phosphatase 1, regulatory (inhibitor) subunit 2**
**PPP1R35**	**protein phosphatase 1, regulatory subunit 35**
**PPP1R37**	**protein phosphatase 1, regulatory subunit 37**
**PPP1R3E**	**protein phosphatase 1, regulatory subunit 3E**
**PPP1R8**	**protein phosphatase 1, regulatory subunit 8**
**PPP1R9B**	**protein phosphatase 1, regulatory subunit 9B**
**PPP2CA**	**protein phosphatase 2, catalytic subunit, alpha isozyme**
**PPP2CB**	**protein phosphatase 2, catalytic subunit, beta isozyme**
**PPP2R1A**	**protein phosphatase 2, regulatory subunit A, alpha**

**PPP2R3C**	**protein phosphatase 2, regulatory subunit B'', gamma**
**PPP2R4**	**protein phosphatase 2A activator, regulatory subunit 4**
**PPP2R5A**	**protein phosphatase 2, regulatory subunit B', alpha**
**PPP2R5E**	**protein phosphatase 2, regulatory subunit B', epsilon isoform**
**PPP3CB**	**protein phosphatase 3, catalytic subunit, beta isozyme**
**PPP3CC**	**protein phosphatase 3, catalytic subunit, gamma isozyme**
**PPP3R1**	**protein phosphatase 3, regulatory subunit B, alpha**
**PPP4C**	**protein phosphatase 4, catalytic subunit**
**PPP4R2**	**protein phosphatase 4, regulatory subunit 2**
**PPP6C**	**protein phosphatase 6, catalytic subunit**
**PPP6R1**	**protein phosphatase 6, regulatory subunit 1**
**PPP6R3**	**protein phosphatase 6, regulatory subunit 3**
**PPRC1**	**peroxisome proliferator-activated receptor gamma, coactivator-related 1**
**PPT1**	**palmitoyl-protein thioesterase 1**
**PPTC7**	**PTC7 protein phosphatase homolog (S. cerevisiae)**
**PQBP1**	**polyglutamine binding protein 1**
**PRADC1**	**protease-associated domain containing 1**
**PRAF2**	**PRA1 domain family, member 2**
**PRC1**	**protein regulator of cytokinesis 1**
**PRCP**	**prolylcarboxypeptidase (angiotensinase C)**
**PRDM10**	**PR domain containing 10**
**PRDX1**	**peroxiredoxin 1**
**PRDX2**	**peroxiredoxin 2**
**PRDX3**	**peroxiredoxin 3**
**PRDX5**	**peroxiredoxin 5**
**PRELID1**	**PRELI domain containing 1**
**PRH1-PRR4**	**PRH1-PRR4 readthrough**
**PRICKLE4**	**prickle homolog 4 (Drosophila)**
**PRIM1**	**primase, DNA, polypeptide 1 (49kDa)**
**PRIM2**	**primase, DNA, polypeptide 2 (58kDa)**
**PRIMPOL**	**primase and polymerase (DNA-directed)**
**PRKAB1**	**protein kinase, AMP-activated, beta 1 non-catalytic subunit**
**PRKAB2**	**protein kinase, AMP-activated, beta 2 non-catalytic subunit**
**PRKACA**	**protein kinase, cAMP-dependent, catalytic, alpha**
**PRKAG1**	**protein kinase, AMP-activated, gamma 1 non-catalytic subunit**
**PRKCD**	**protein kinase C, delta**
**PRKCDBP**	**protein kinase C, delta binding protein**
**PRKCE**	**protein kinase C, epsilon**
**PRKCI**	**protein kinase C, iota**
**PRKCSH**	**protein kinase C substrate 80K-H**
**PRKD2**	**protein kinase D2**

PRKDC	protein kinase, DNA-activated, catalytic polypeptide
PRKRIP1	PRKR interacting protein 1 (IL11 inducible)
PRLH	prolactin releasing hormone
PRMT1	protein arginine methyltransferase 1
PRMT2	protein arginine methyltransferase 2
PRMT3	protein arginine methyltransferase 3
PRMT5	protein arginine methyltransferase 5
PRMT7	protein arginine methyltransferase 7
PRMT9	protein arginine methyltransferase 9
PROB1	proline-rich basic protein 1
PROCA1	protein interacting with cyclin A1
PRORSD1P	prolyl-tRNA synthetase associated domain containing 1, pseudogene
PROSC	proline synthetase co-transcribed homolog (bacterial)
PROSER1	proline and serine rich 1
PROSER3	proline and serine rich 3
PRPF3	pre-mRNA processing factor 3
PRPF31	pre-mRNA processing factor 31
PRPF38A	pre-mRNA processing factor 38A
PRPF38B	pre-mRNA processing factor 38B
PRPF4	pre-mRNA processing factor 4
PRPF40A	PRP40 pre-mRNA processing factor 40 homolog A (S. cerevisiae)
PRPF40B	PRP40 pre-mRNA processing factor 40 homolog B (S. cerevisiae)
PRPF6	pre-mRNA processing factor 6
PRR11	proline rich 11
PRR14L	proline rich 14-like
PRR19	proline rich 19
PRR29	proline rich 29
PRR7	proline rich 7 (synaptic)
PRRC2C	proline-rich coiled-coil 2C
PRRG2	proline rich Gla (G-carboxyglutamic acid) 2
PRRT2	proline-rich transmembrane protein 2
PRRT3	proline-rich transmembrane protein 3
PRSS27	protease, serine 27
PSD	pleckstrin and Sec7 domain containing
PSD4	pleckstrin and Sec7 domain containing 4
PSEN1	presenilin 1
PSIP1	PC4 and SFRS1 interacting protein 1
PSMA2	proteasome (prosome, macropain) subunit, alpha type, 2
PSMA3	proteasome (prosome, macropain) subunit, alpha type, 3
PSMA4	proteasome (prosome, macropain) subunit, alpha type, 4
PSMA6	proteasome (prosome, macropain) subunit, alpha type, 6

**PSMB10**	**proteasome (prosome, macropain) subunit, beta type, 10**
**PSMB2**	**proteasome (prosome, macropain) subunit, beta type, 2**
**PSMB3**	**proteasome (prosome, macropain) subunit, beta type, 3**
**PSMB4**	**proteasome (prosome, macropain) subunit, beta type, 4**
**PSMB7**	**proteasome (prosome, macropain) subunit, beta type, 7**
**PSMB8**	**proteasome (prosome, macropain) subunit, beta type, 8**
**PSMC2**	**proteasome (prosome, macropain) 26S subunit, ATPase, 2**
**PSMC3IP**	**PSMC3 interacting protein**
**PSMC4**	**proteasome (prosome, macropain) 26S subunit, ATPase, 4**
**PSMC5**	**proteasome (prosome, macropain) 26S subunit, ATPase, 5**
**PSMD1**	**proteasome (prosome, macropain) 26S subunit, non-ATPase, 1**
**PSMD12**	**proteasome (prosome, macropain) 26S subunit, non-ATPase, 12**
**PSMD13**	**proteasome (prosome, macropain) 26S subunit, non-ATPase, 13**
**PSMD14**	**proteasome (prosome, macropain) 26S subunit, non-ATPase, 14**
**PSMD2**	**proteasome (prosome, macropain) 26S subunit, non-ATPase, 2**
**PSMD3**	**proteasome (prosome, macropain) 26S subunit, non-ATPase, 3**
**PSMD4**	**proteasome (prosome, macropain) 26S subunit, non-ATPase, 4**
**PSMD5**	**proteasome (prosome, macropain) 26S subunit, non-ATPase, 5**
**PSMD9**	**proteasome (prosome, macropain) 26S subunit, non-ATPase, 9**
**PSME1**	**proteasome (prosome, macropain) activator subunit 1 (PA28 alpha)**
**PSME2**	**proteasome (prosome, macropain) activator subunit 2 (PA28 beta)**
**PSME3**	**proteasome (prosome, macropain) activator subunit 3 (PA28 gamma; Ki)**
**PSMG1**	**proteasome (prosome, macropain) assembly chaperone 1**
**PSMG3**	**proteasome (prosome, macropain) assembly chaperone 3**
**PSPH**	**phosphoserine phosphatase**
**PTAR1**	**protein prenyltransferase alpha subunit repeat containing 1**
**PTBP1**	**polypyrimidine tract binding protein 1**
**PTCD2**	**pentatricopeptide repeat domain 2**
**PTCD3**	**pentatricopeptide repeat domain 3**
**PTDSS1**	**phosphatidylserine synthase 1**
**PTDSS2**	**phosphatidylserine synthase 2**
**PTEN**	**phosphatase and tensin homolog**
**PTGES2**	**prostaglandin E synthase 2**
**PTGES3**	**prostaglandin E synthase 3 (cytosolic)**
**PTGR2**	**prostaglandin reductase 2**
**PTK2**	**protein tyrosine kinase 2**
**PTK2B**	**protein tyrosine kinase 2 beta**
**PTK6**	**protein tyrosine kinase 6**
**PTMA**	**prothymosin, alpha**
**PTOV1**	**prostate tumor overexpressed 1**
**PTP4A1**	**protein tyrosine phosphatase type IVA, member 1**

**PTP4A2**	**protein tyrosine phosphatase type IVA, member 2**
**PTPN1**	**protein tyrosine phosphatase, non-receptor type 1**
**PTPN11**	**protein tyrosine phosphatase, non-receptor type 11**
**PTPN12**	**protein tyrosine phosphatase, non-receptor type 12**
**PTPN2**	**protein tyrosine phosphatase, non-receptor type 2**
**PTPN22**	**protein tyrosine phosphatase, non-receptor type 22 (lymphoid)**
**PTPN23**	**protein tyrosine phosphatase, non-receptor type 23**
**PTPN4**	**protein tyrosine phosphatase, non-receptor type 4 (megakaryocyte)**
**PTPN6**	**protein tyrosine phosphatase, non-receptor type 6**
**PTPN7**	**protein tyrosine phosphatase, non-receptor type 7**
**PTPN9**	**protein tyrosine phosphatase, non-receptor type 9**
**PTPRC**	**protein tyrosine phosphatase, receptor type, C**
**PTPRCAP**	**protein tyrosine phosphatase, receptor type, C-associated protein**
**PTRH2**	**peptidyl-tRNA hydrolase 2**
**PTRHD1**	**peptidyl-tRNA hydrolase domain containing 1**
**PTS**	**6-pyruvoyltetrahydropterin synthase**
**PUF60**	**poly-U binding splicing factor 60KDa**
**PURA**	**purine-rich element binding protein A**
**PURB**	**purine-rich element binding protein B**
**PUS10**	**pseudouridylate synthase 10**
**PUS3**	**pseudouridylate synthase 3**
**PUS7L**	**pseudouridylate synthase 7 homolog (S. cerevisiae)-like**
**PUSL1**	**pseudouridylate synthase-like 1**
**PVRIG**	**poliovirus receptor related immunoglobulin domain containing**
**PVT1**	**Pvt1 oncogene (non-protein coding)**
**PWP1**	**PWP1 homolog (S. cerevisiae)**
**PWP2**	**PWP2 periodic tryptophan protein homolog (yeast)**
**PWWP2A**	**PWWP domain containing 2A**
**PXK**	**PX domain containing serine/threonine kinase**
**PXMP2**	**peroxisomal membrane protein 2, 22kDa**
**PXMP4**	**peroxisomal membrane protein 4, 24kDa**
**PXT1**	**peroxisomal, testis specific 1**
**PYCARD**	**PYD and CARD domain containing**
**PYCR2**	**pyrroline-5-carboxylate reductase family, member 2**
**PYCRL**	**pyrroline-5-carboxylate reductase-like**
**PYGM**	**phosphorylase, glycogen, muscle**
**PYGO2**	**pygopus family PHD finger 2**
**PYROXD1**	**pyridine nucleotide-disulphide oxidoreductase domain 1**
**QKI**	**QKI, KH domain containing, RNA binding**
**QPCTL**	**glutaminyl-peptide cyclotransferase-like**
**QRICH1**	**glutamine-rich 1**

QSER1	glutamine and serine rich 1
QTRT1	queuine tRNA-ribosyltransferase 1
R3HCC1	R3H domain and coiled-coil containing 1
R3HCC1L	R3H domain and coiled-coil containing 1-like
R3HDM1	R3H domain containing 1
RAB10	RAB10, member RAS oncogene family
RAB11A	RAB11A, member RAS oncogene family
RAB11FIP1	RAB11 family interacting protein 1 (class I)
RAB12	RAB12, member RAS oncogene family
RAB13	RAB13, member RAS oncogene family
RAB14	RAB14, member RAS oncogene family
RAB18	RAB18, member RAS oncogene family
RAB1B	RAB1B, member RAS oncogene family
RAB21	RAB21, member RAS oncogene family
RAB24	RAB24, member RAS oncogene family
RAB27A	RAB27A, member RAS oncogene family
RAB30	RAB30, member RAS oncogene family
RAB33B	RAB33B, member RAS oncogene family
RAB34	RAB34, member RAS oncogene family
RAB35	RAB35, member RAS oncogene family
RAB3GAP1	RAB3 GTPase activating protein subunit 1 (catalytic)
RAB3GAP2	RAB3 GTPase activating protein subunit 2 (non-catalytic)
RAB40C	RAB40C, member RAS oncogene family
RAB43	RAB43, member RAS oncogene family
RAB4B	RAB4B, member RAS oncogene family
RAB4B-EGLN2	RAB4B-EGLN2 readthrough (NMD candidate)
RAB5A	RAB5A, member RAS oncogene family
RAB5B	RAB5B, member RAS oncogene family
RAB8A	RAB8A, member RAS oncogene family
RABEP2	rabaptin, RAB GTPase binding effector protein 2
RABEPK	Rab9 effector protein with kelch motifs
RABGAP1	RAB GTPase activating protein 1
RABGAP1L	RAB GTPase activating protein 1-like
RABGEF1	RAB guanine nucleotide exchange factor (GEF) 1
RABGGTA	Rab geranylgeranyltransferase, alpha subunit
RABGGTB	Rab geranylgeranyltransferase, beta subunit
RABIF	RAB interacting factor
RABL3	RAB, member of RAS oncogene family-like 3
RAC2	ras-related C3 botulinum toxin substrate 2 (rho family, small GTP binding protein Rac2)
RAD1	RAD1 checkpoint DNA exonuclease
RAD17	RAD17 homolog (S. pombe)

**RAD18**	**RAD18 E3 ubiquitin protein ligase**
**RAD23A**	**RAD23 homolog A (S. cerevisiae)**
**RAD23B**	**RAD23 homolog B (S. cerevisiae)**
**RAD50**	**RAD50 homolog (S. cerevisiae)**
**RAD51**	**RAD51 recombinase**
**RAD51AP1**	**RAD51 associated protein 1**
**RAD51C**	**RAD51 paralog C**
**RAD52**	**RAD52 homolog (S. cerevisiae)**
**RAD9A**	**RAD9 homolog A (S. pombe)**
**RAD9B**	**RAD9 homolog B (S. pombe)**
**RAF1**	**Raf-1 proto-oncogene, serine/threonine kinase**
**RALA**	**v-ral simian leukemia viral oncogene homolog A (ras related)**
**RALBP1**	**ralA binding protein 1**
**RALGDS**	**ral guanine nucleotide dissociation stimulator**
**RALY**	**RALY heterogeneous nuclear ribonucleoprotein**
**RAN**	**RAN, member RAS oncogene family**
**RANBP1**	**RAN binding protein 1**
**RANBP2**	**RAN binding protein 2**
**RANBP3**	**RAN binding protein 3**
**RANBP6**	**RAN binding protein 6**
**RANBP9**	**RAN binding protein 9**
**RANGAP1**	**Ran GTPase activating protein 1**
**RANGRF**	**RAN guanine nucleotide release factor**
**RAP1A**	**RAP1A, member of RAS oncogene family**
**RAP2A**	**RAP2A, member of RAS oncogene family**
**RAP2B**	**RAP2B, member of RAS oncogene family**
**RAPGEF6**	**Rap guanine nucleotide exchange factor (GEF) 6**
**RARA**	**retinoic acid receptor, alpha**
**RARS**	**arginyl-tRNA synthetase**
**RARS2**	**arginyl-tRNA synthetase 2, mitochondrial**
**RASA1**	**RAS p21 protein activator (GTPase activating protein) 1**
**RASA3**	**RAS p21 protein activator 3**
**RASAL3**	**RAS protein activator like 3**
**RASGRF2**	**Ras protein-specific guanine nucleotide-releasing factor 2**
**RASGRP1**	**RAS guanyl releasing protein 1 (calcium and DAG-regulated)**
**RASSF2**	**Ras association (RalGDS/AF-6) domain family member 2**
**RASSF5**	**Ras association (RalGDS/AF-6) domain family member 5**
**RAVER1**	**ribonucleoprotein, PTB-binding 1**
**RB1**	**retinoblastoma 1**
**RBAK**	**RB-associated KRAB zinc finger**
**RBAK-RBAKDN**	**RBAK-RBAKDN readthrough**

**RBBP4**	**retinoblastoma binding protein 4**
**RBBP5**	**retinoblastoma binding protein 5**
**RBBP6**	**retinoblastoma binding protein 6**
**RBFA**	**ribosome binding factor A (putative)**
**RBKS**	**ribokinase**
**RBL1**	**retinoblastoma-like 1**
**RBM12**	**RNA binding motif protein 12**
**RBM12B**	**RNA binding motif protein 12B**
**RBM12B-AS1**	**RBM12B antisense RNA 1**
**RBM14**	**RNA binding motif protein 14**
**RBM14-RBM4**	**RBM14-RBM4 readthrough**
**RBM15**	**RNA binding motif protein 15**
**RBM15B**	**RNA binding motif protein 15B**
**RBM17**	**RNA binding motif protein 17**
**RBM18**	**RNA binding motif protein 18**
**RBM22**	**RNA binding motif protein 22**
**RBM23**	**RNA binding motif protein 23**
**RBM25**	**RNA binding motif protein 25**
**RBM26**	**RNA binding motif protein 26**
**RBM26-AS1**	**RBM26 antisense RNA 1**
**RBM27**	**RNA binding motif protein 27**
**RBM28**	**RNA binding motif protein 28**
**RBM3**	**RNA binding motif (RNP1, RRM) protein 3**
**RBM34**	**RNA binding motif protein 34**
**RBM38**	**RNA binding motif protein 38**
**RBM39**	**RNA binding motif protein 39**
**RBM4**	**RNA binding motif protein 4**
**RBM42**	**RNA binding motif protein 42**
**RBM43**	**RNA binding motif protein 43**
**RBM45**	**RNA binding motif protein 45**
**RBM47**	**RNA binding motif protein 47**
**RBM4B**	**RNA binding motif protein 4B**
**RBM5**	**RNA binding motif protein 5**
**RBM6**	**RNA binding motif protein 6**
**RBM7**	**RNA binding motif protein 7**
**RBMX**	**RNA binding motif protein, X-linked**
**RBMXL1**	**RNA binding motif protein, X-linked-like 1**
**RBP5**	**retinol binding protein 5, cellular**
**RBPJ**	**recombination signal binding protein for immunoglobulin kappa J region**
**RBSN**	**rabenosyn, RAB effector**
**RBX1**	**ring-box 1, E3 ubiquitin protein ligase**

**RC3H2**	**ring finger and CCCH-type domains 2**
**RCC1**	**regulator of chromosome condensation 1**
**RCC2**	**regulator of chromosome condensation 2**
**RCCD1**	**RCC1 domain containing 1**
**RCE1**	**Ras converting CAAX endopeptidase 1**
**RCHY1**	**ring finger and CHY zinc finger domain containing 1, E3 ubiquitin protein ligase**
**RCOR1**	**REST corepressor 1**
**RCOR3**	**REST corepressor 3**
**RCSD1**	**RCSD domain containing 1**
**RDH10**	**retinol dehydrogenase 10 (all-trans)**
**RDH11**	**retinol dehydrogenase 11 (all-trans/9-cis/11-cis)**
**RDX**	**radixin**
**REC8**	**REC8 meiotic recombination protein**
**RECQL**	**RecQ helicase-like**
**RECQL5**	**RecQ protein-like 5**
**REEP2**	**receptor accessory protein 2**
**REL**	**v-rel avian reticuloendotheliosis viral oncogene homolog**
**RELA**	**v-rel avian reticuloendotheliosis viral oncogene homolog A**
**RELB**	**v-rel avian reticuloendotheliosis viral oncogene homolog B**
**RELT**	**RELT tumor necrosis factor receptor**
**REPIN1**	**replication initiator 1**
**RETSAT**	**retinol saturase (all-trans-retinol 13,14-reductase)**
**REV1**	**REV1, polymerase (DNA directed)**
**REV3L**	**REV3-like, polymerase (DNA directed), zeta, catalytic subunit**
**REXO1**	**REX1, RNA exonuclease 1 homolog (S. cerevisiae)**
**REXO4**	**REX4, RNA exonuclease 4 homolog (S. cerevisiae)**
**RFC1**	**replication factor C (activator 1) 1, 145kDa**
**RFC2**	**replication factor C (activator 1) 2, 40kDa**
**RFC3**	**replication factor C (activator 1) 3, 38kDa**
**RFC4**	**replication factor C (activator 1) 4, 37kDa**
**RFFL**	**ring finger and FYVE-like domain containing E3 ubiquitin protein ligase**
**RFK**	**riboflavin kinase**
**RFT1**	**RFT1 homolog (S. cerevisiae)**
**RFTN1**	**raftlin, lipid raft linker 1**
**RFWD2**	**ring finger and WD repeat domain 2, E3 ubiquitin protein ligase**
**RFWD3**	**ring finger and WD repeat domain 3**
**RFX1**	**regulatory factor X, 1 (influences HLA class II expression)**
**RFX3**	**regulatory factor X, 3 (influences HLA class II expression)**
**RFX5**	**regulatory factor X, 5 (influences HLA class II expression)**
**RFXANK**	**regulatory factor X-associated ankyrin-containing protein**
**RGL1**	**ral guanine nucleotide dissociation stimulator-like 1**

**RGL2**	**ral guanine nucleotide dissociation stimulator-like 2**
**RGS1**	**regulator of G-protein signaling 1**
**RGS10**	**regulator of G-protein signaling 10**
**RGS14**	**regulator of G-protein signaling 14**
**RGS16**	**regulator of G-protein signaling 16**
**RGS5**	**regulator of G-protein signaling 5**
**RGS6**	**regulator of G-protein signaling 6**
**RHBDD1**	**rhomboid domain containing 1**
**RHBDD3**	**rhomboid domain containing 3**
**RHBDL1**	**rhomboid, veinlet-like 1 (Drosophila)**
**RHEBL1**	**Ras homolog enriched in brain like 1**
**RHOA**	**ras homolog family member A**
**RHOC**	**ras homolog family member C**
**RHOF**	**ras homolog family member F (in filopodia)**
**RHOG**	**ras homolog family member G**
**RHOH**	**ras homolog family member H**
**RHOQ**	**ras homolog family member Q**
**RHOT2**	**ras homolog family member T2**
**RHOV**	**ras homolog family member V**
**RIBC1**	**RIB43A domain with coiled-coils 1**
**RIC1**	**RAB6A GEF complex partner 1**
**RIC8A**	**RIC8 guanine nucleotide exchange factor A**
**RIF1**	**replication timing regulatory factor 1**
**RILP**	**Rab interacting lysosomal protein**
**RILPL2**	**Rab interacting lysosomal protein-like 2**
**RIN1**	**Ras and Rab interactor 1**
**RINL**	**Ras and Rab interactor-like**
**RINT1**	**RAD50 interactor 1**
**RIOK1**	**RIO kinase 1**
**RIOK2**	**RIO kinase 2**
**RIOK3**	**RIO kinase 3**
**RIPK2**	**receptor-interacting serine-threonine kinase 2**
**RIT1**	**Ras-like without CAAX 1**
**RITA1**	**RBPJ interacting and tubulin associated 1**
**RLF**	**rearranged L-myc fusion**
**RLIM**	**ring finger protein, LIM domain interacting**
**RLTPR**	**RGD motif, leucine rich repeats, tropomodulin domain and proline-rich containing**
**RMDN1**	**regulator of microtubule dynamics 1**
**RMDN3**	**regulator of microtubule dynamics 3**
**RMI1**	**RecQ mediated genome instability 1**
**RMI2**	**RecQ mediated genome instability 2**

**RMND1**	**required for meiotic nuclear division 1 homolog (S. cerevisiae)**
**RMND5B**	**required for meiotic nuclear division 5 homolog B (S. cerevisiae)**
**RMRP**	**RNA component of mitochondrial RNA processing endoribonuclease**
**RNASE4**	**ribonuclease, RNase A family, 4**
**RNASE6**	**ribonuclease, RNase A family, k6**
**RNASEH1**	**ribonuclease H1**
**RNASEH2C**	**ribonuclease H2, subunit C**
**RNASEK**	**ribonuclease, RNase K**
**RNASEK-C17ORF49**	**RNASEK-C17orf49 readthrough**
**RNASET2**	**ribonuclease T2**
**RND1**	**Rho family GTPase 1**
**RNF115**	**ring finger protein 115**
**RNF121**	**ring finger protein 121**
**RNF123**	**ring finger protein 123**
**RNF126**	**ring finger protein 126**
**RNF13**	**ring finger protein 13**
**RNF135**	**ring finger protein 135**
**RNF138**	**ring finger protein 138, E3 ubiquitin protein ligase**
**RNF139**	**ring finger protein 139**
**RNF141**	**ring finger protein 141**
**RNF146**	**ring finger protein 146**
**RNF149**	**ring finger protein 149**
**RNF151**	**ring finger protein 151**
**RNF167**	**ring finger protein 167**
**RNF168**	**ring finger protein 168, E3 ubiquitin protein ligase**
**RNF181**	**ring finger protein 181**
**RNF187**	**ring finger protein 187**
**RNF19A**	**ring finger protein 19A, RBR E3 ubiquitin protein ligase**
**RNF19B**	**ring finger protein 19B**
**RNF213**	**ring finger protein 213**
**RNF216**	**ring finger protein 216**
**RNF25**	**ring finger protein 25**
**RNF31**	**ring finger protein 31**
**RNF32**	**ring finger protein 32**
**RNF34**	**ring finger protein 34, E3 ubiquitin protein ligase**
**RNF38**	**ring finger protein 38**
**RNF4**	**ring finger protein 4**
**RNF41**	**ring finger protein 41, E3 ubiquitin protein ligase**
**RNF43**	**ring finger protein 43**
**RNF44**	**ring finger protein 44**
**RNF7**	**ring finger protein 7**

**RNFT1**	**ring finger protein, transmembrane 1**
**RNGTT**	**RNA guanylyltransferase and 5'-phosphatase**
**RNH1**	**ribonuclease/angiogenin inhibitor 1**
**RNLS**	**renalase, FAD-dependent amine oxidase**
**RNMT**	**RNA (guanine-7-) methyltransferase**
**RNMTL1**	**RNA methyltransferase like 1**
**RNPC3**	**RNA-binding region (RNP1, RRM) containing 3**
**RNPS1**	**RNA binding protein S1, serine-rich domain**
**RNU11**	**RNA, U11 small nuclear**
**RNU12**	**RNA, U12 small nuclear**
**RNU4ATAC**	**RNA, U4atac small nuclear (U12-dependent splicing)**
**RNU86**	**RNA, U86 small nucleolar**
**ROGDI**	**rogdi homolog (Drosophila)**
**ROMO1**	**reactive oxygen species modulator 1**
**ROPN1L**	**rhophilin associated tail protein 1-like**
**RPA1**	**replication protein A1, 70kDa**
**RPA2**	**replication protein A2, 32kDa**
**RPAP1**	**RNA polymerase II associated protein 1**
**RPAP3**	**RNA polymerase II associated protein 3**
**RPF1**	**ribosome production factor 1 homolog (S. cerevisiae)**
**RPF2**	**ribosome production factor 2 homolog (S. cerevisiae)**
**RPIA**	**ribose 5-phosphate isomerase A**
**RPL10**	**ribosomal protein L10**
**RPL10A**	**ribosomal protein L10a**
**RPL11**	**ribosomal protein L11**
**RPL12**	**ribosomal protein L12**
**RPL13**	**ribosomal protein L13**
**RPL13A**	**ribosomal protein L13a**
**RPL13AP5**	**ribosomal protein L13a pseudogene 5**
**RPL14**	**ribosomal protein L14**
**RPL15**	**ribosomal protein L15**
**RPL17**	**ribosomal protein L17**
**RPL17-C18ORF32**	**RPL17-C18orf32 readthrough**
**RPL18**	**ribosomal protein L18**
**RPL18A**	**ribosomal protein L18a**
**RPL19**	**ribosomal protein L19**
**RPL21**	**ribosomal protein L21**
**RPL21P28**	**ribosomal protein L21 pseudogene 28**
**RPL22**	**ribosomal protein L22**
**RPL22L1**	**ribosomal protein L22-like 1**
**RPL23**	**ribosomal protein L23**

**RPL23A**	**ribosomal protein L23a**
**RPL24**	**ribosomal protein L24**
**RPL26**	**ribosomal protein L26**
**RPL26L1**	**ribosomal protein L26-like 1**
**RPL27**	**ribosomal protein L27**
**RPL27A**	**ribosomal protein L27a**
**RPL28**	**ribosomal protein L28**
**RPL29**	**ribosomal protein L29**
**RPL3**	**ribosomal protein L3**
**RPL31**	**ribosomal protein L31**
**RPL32**	**ribosomal protein L32**
**RPL32P3**	**ribosomal protein L32 pseudogene 3**
**RPL34**	**ribosomal protein L34**
**RPL35**	**ribosomal protein L35**
**RPL35A**	**ribosomal protein L35a**
**RPL36**	**ribosomal protein L36**
**RPL36A**	**ribosomal protein L36a**
**RPL36A-HNRNPH2**	**RPL36A-HNRNPH2 readthrough**
**RPL36AL**	**ribosomal protein L36a-like**
**RPL37**	**ribosomal protein L37**
**RPL37A**	**ribosomal protein L37a**
**RPL38**	**ribosomal protein L38**
**RPL39**	**ribosomal protein L39**
**RPL4**	**ribosomal protein L4**
**RPL41**	**ribosomal protein L41**
**RPL5**	**ribosomal protein L5**
**RPL6**	**ribosomal protein L6**
**RPL7**	**ribosomal protein L7**
**RPL7A**	**ribosomal protein L7a**
**RPL7L1**	**ribosomal protein L7-like 1**
**RPL8**	**ribosomal protein L8**
**RPL9**	**ribosomal protein L9**
**RPLP0**	**ribosomal protein, large, P0**
**RPLP1**	**ribosomal protein, large, P1**
**RPLP2**	**ribosomal protein, large, P2**
**RPN1**	**ribophorin I**
**RPN2**	**ribophorin II**
**RPP14**	**ribonuclease P/MRP 14kDa subunit**
**RPP25L**	**ribonuclease P/MRP 25kDa subunit-like**
**RPP38**	**ribonuclease P/MRP 38kDa subunit**
**RPP40**	**ribonuclease P/MRP 40kDa subunit**

RPPH1	ribonuclease P RNA component H1
RPRD1A	regulation of nuclear pre-mRNA domain containing 1A
RPRD1B	regulation of nuclear pre-mRNA domain containing 1B
RPRD2	regulation of nuclear pre-mRNA domain containing 2
RPS10	ribosomal protein S10
RPS10-NUDT3	RPS10-NUDT3 readthrough
RPS11	ribosomal protein S11
RPS12	ribosomal protein S12
RPS13	ribosomal protein S13
RPS14	ribosomal protein S14
RPS15	ribosomal protein S15
RPS15A	ribosomal protein S15a
RPS16	ribosomal protein S16
RPS19	ribosomal protein S19
RPS19BP1	ribosomal protein S19 binding protein 1
RPS2	ribosomal protein S2
RPS20	ribosomal protein S20
RPS21	ribosomal protein S21
RPS23	ribosomal protein S23
RPS24	ribosomal protein S24
RPS25	ribosomal protein S25
RPS26	ribosomal protein S26
RPS27	ribosomal protein S27
RPS27A	ribosomal protein S27a
RPS27L	ribosomal protein S27-like
RPS28	ribosomal protein S28
RPS29	ribosomal protein S29
RPS2P32	ribosomal protein S2 pseudogene 32
RPS3	ribosomal protein S3
RPS3A	ribosomal protein S3A
RPS4X	ribosomal protein S4, X-linked
RPS5	ribosomal protein S5
RPS6	ribosomal protein S6
RPS6KA3	ribosomal protein S6 kinase, 90kDa, polypeptide 3
RPS6KB1	ribosomal protein S6 kinase, 70kDa, polypeptide 1
RPS6KB2	ribosomal protein S6 kinase, 70kDa, polypeptide 2
RPS7	ribosomal protein S7
RPS8	ribosomal protein S8
RPS9	ribosomal protein S9
RPSA	ribosomal protein SA
RPTOR	regulatory associated protein of MTOR, complex 1

**RPUSD2**	**RNA pseudouridylate synthase domain containing 2**
**RPUSD4**	**RNA pseudouridylate synthase domain containing 4**
**RQCD1**	**RCD1 required for cell differentiation1 homolog (S. pombe)**
**RRAGA**	**Ras-related GTP binding A**
**RRAS**	**related RAS viral (r-ras) oncogene homolog**
**RREB1**	**ras responsive element binding protein 1**
**RRM1**	**ribonucleotide reductase M1**
**RRM2**	**ribonucleotide reductase M2**
**RRM2B**	**ribonucleotide reductase M2 B (TP53 inducible)**
**RRNAD1**	**ribosomal RNA adenine dimethylase domain containing 1**
**RRP1**	**ribosomal RNA processing 1**
**RRP15**	**ribosomal RNA processing 15 homolog (S. cerevisiae)**
**RRP1B**	**ribosomal RNA processing 1B**
**RRP36**	**ribosomal RNA processing 36 homolog (S. cerevisiae)**
**RRP7BP**	**ribosomal RNA processing 7 homolog B (S. cerevisiae), pseudogene**
**RRP8**	**ribosomal RNA processing 8, methyltransferase, homolog (yeast)**
**RRP9**	**ribosomal RNA processing 9, small subunit (SSU) processome component, homolog (yeast)**
**RRS1**	**RRS1 ribosome biogenesis regulator homolog (S. cerevisiae)**
**RSAD1**	**radical S-adenosyl methionine domain containing 1**
**RSBN1**	**round spermatid basic protein 1**
**RSF1**	**remodeling and spacing factor 1**
**RSL1D1**	**ribosomal L1 domain containing 1**
**RSL24D1**	**ribosomal L24 domain containing 1**
**RSPH3**	**radial spoke 3 homolog (Chlamydomonas)**
**RSPH6A**	**radial spoke head 6 homolog A (Chlamydomonas)**
**RSRC1**	**arginine/serine-rich coiled-coil 1**
**RSRC2**	**arginine/serine-rich coiled-coil 2**
**RSRP1**	**arginine/serine-rich protein 1**
**RTEL1**	**regulator of telomere elongation helicase 1**
**RTEL1-TNFRSF6B**	**RTEL1-TNFRSF6B readthrough (NMD candidate)**
**RTFDC1**	**replication termination factor 2 domain containing 1**
**RTN4**	**reticulon 4**
**RTTN**	**rotatin**
**RUFY1**	**RUN and FYVE domain containing 1**
**RUNX1**	**runt-related transcription factor 1**
**RUNX3**	**runt-related transcription factor 3**
**RUSC1**	**RUN and SH3 domain containing 1**
**RUSC1-AS1**	**RUSC1 antisense RNA 1**
**RUVBL1**	**RuvB-like AAA ATPase 1**
**RUVBL2**	**RuvB-like AAA ATPase 2**
**RWDD1**	**RWD domain containing 1**

RWDD3	RWD domain containing 3
RXFP4	relaxin/insulin-like family peptide receptor 4
S100A13	S100 calcium binding protein A13
S100PBP	S100P binding protein
S1PR1	sphingosine-1-phosphate receptor 1
S1PR2	sphingosine-1-phosphate receptor 2
S1PR4	sphingosine-1-phosphate receptor 4
SAC3D1	SAC3 domain containing 1
SACM1L	SAC1 suppressor of actin mutations 1-like (yeast)
SAE1	SUMO1 activating enzyme subunit 1
SAFB	scaffold attachment factor B
SAFB2	scaffold attachment factor B2
SAMD1	sterile alpha motif domain containing 1
SAMD10	sterile alpha motif domain containing 10
SAMD4B	sterile alpha motif domain containing 4B
SAMHD1	SAM domain and HD domain 1
SAP18	Sin3A-associated protein, 18kDa
SAP30	Sin3A-associated protein, 30kDa
SAP30BP	SAP30 binding protein
SAP30L	SAP30-like
SAR1A	secretion associated, Ras related GTPase 1A
SAR1B	secretion associated, Ras related GTPase 1B
SARM1	sterile alpha and TIR motif containing 1
SARNP	SAP domain containing ribonucleoprotein
SARS	seryl-tRNA synthetase
SARS2	seryl-tRNA synthetase 2, mitochondrial
SART1	squamous cell carcinoma antigen recognized by T cells
SART3	squamous cell carcinoma antigen recognized by T cells 3
SASH3	SAM and SH3 domain containing 3
SAT1	spermidine/spermine N1-acetyltransferase 1
SAV1	salvador family WW domain containing protein 1
SBDS	Shwachman-Bodian-Diamond syndrome
SCAF1	SR-related CTD-associated factor 1
SCAF11	SR-related CTD-associated factor 11
SCAF8	SR-related CTD-associated factor 8
SCAI	suppressor of cancer cell invasion
SCAMP1	secretory carrier membrane protein 1
SCAMP3	secretory carrier membrane protein 3
SCAMP4	secretory carrier membrane protein 4
SCAND1	SCAN domain containing 1
SCARB1	scavenger receptor class B, member 1

**SCARF1**	**scavenger receptor class F, member 1**
**SCARNA16**	**small Cajal body-specific RNA 16**
**SCARNA2**	**small Cajal body-specific RNA 2**
**SCFD1**	**sec1 family domain containing 1**
**SCFD2**	**sec1 family domain containing 2**
**SCIMP**	**SLP adaptor and CSK interacting membrane protein**
**SCN4A**	**sodium channel, voltage gated, type IV alpha subunit**
**SCN5A**	**sodium channel, voltage gated, type V alpha subunit**
**SCNM1**	**sodium channel modifier 1**
**SCO2**	**SCO2 cytochrome c oxidase assembly protein**
**SCRIB**	**scribbled planar cell polarity protein**
**SCRN2**	**secernin 2**
**SCYL3**	**SCY1-like 3 (S. cerevisiae)**
**SDAD1**	**SDA1 domain containing 1**
**SDC1**	**syndecan 1**
**SDCBP**	**syndecan binding protein (syntenin)**
**SDCCAG3**	**serologically defined colon cancer antigen 3**
**SDCCAG8**	**serologically defined colon cancer antigen 8**
**SDE2**	**SDE2 telomere maintenance homolog (S. pombe)**
**SDF2**	**stromal cell-derived factor 2**
**SDF4**	**stromal cell derived factor 4**
**SDHAF2**	**succinate dehydrogenase complex assembly factor 2**
**SDHAF3**	**succinate dehydrogenase complex assembly factor 3**
**SDHAP3**	**succinate dehydrogenase complex, subunit A, flavoprotein pseudogene 3**
**SDHD**	**succinate dehydrogenase complex, subunit D, integral membrane protein**
**SDR39U1**	**short chain dehydrogenase/reductase family 39U, member 1**
**SEC11A**	**SEC11 homolog A (S. cerevisiae)**
**SEC11C**	**SEC11 homolog C (S. cerevisiae)**
**SEC13**	**SEC13 homolog (S. cerevisiae)**
**SEC14L1**	**SEC14-like 1 (S. cerevisiae)**
**SEC14L5**	**SEC14-like 5 (S. cerevisiae)**
**SEC16A**	**SEC16 homolog A (S. cerevisiae)**
**SEC1P**	**secretory blood group 1, pseudogene**
**SEC22A**	**SEC22 vesicle trafficking protein homolog A (S. cerevisiae)**
**SEC22B**	**SEC22 vesicle trafficking protein homolog B (S. cerevisiae) (gene/pseudogene)**
**SEC22C**	**SEC22 vesicle trafficking protein homolog C (S. cerevisiae)**
**SEC23B**	**Sec23 homolog B (S. cerevisiae)**
**SEC23IP**	**SEC23 interacting protein**
**SEC24A**	**SEC24 family member A**
**SEC24C**	**SEC24 family member C**
**SEC31A**	**SEC31 homolog A (S. cerevisiae)**

**SEC61A1**	**Sec61 alpha 1 subunit (S. cerevisiae)**
**SEC61A2**	**Sec61 alpha 2 subunit (S. cerevisiae)**
**SEC61B**	**Sec61 beta subunit**
**SEC61G**	**Sec61 gamma subunit**
**SEC62**	**SEC62 homolog (S. cerevisiae)**
**SECISBP2**	**SECIS binding protein 2**
**SECISBP2L**	**SECIS binding protein 2-like**
**SEH1L**	**SEH1-like (S. cerevisiae)**
**SELK**	**selenoprotein K**
**SELO**	**selenoprotein O**
**SELPLG**	**selectin P ligand**
**SELT**	**selenoprotein T**
**SEMA3B**	**sema domain, immunoglobulin domain (Ig), short basic domain, secreted, (semaphorin) 3B**
**SEMA4C**	**sema domain, immunoglobulin domain (Ig), transmembrane domain (TM) and short cytoplasmic domain, (semaphorin) 4C**
**SEMA6A**	**sema domain, transmembrane domain (TM), and cytoplasmic domain, (semaphorin) 6A**
**SEMA7A**	**semaphorin 7A, GPI membrane anchor (John Milton Hagen blood group)**
**SENP1**	**SUMO1/sentrin specific peptidase 1**
**SENP2**	**SUMO1/sentrin/SMT3 specific peptidase 2**
**SENP3**	**SUMO1/sentrin/SMT3 specific peptidase 3**
**SENP3-EIF4A1**	**SENP3-EIF4A1 readthrough (NMD candidate)**
**SENP5**	**SUMO1/sentrin specific peptidase 5**
**SENP6**	**SUMO1/sentrin specific peptidase 6**
**Sep-15**	**15 kDa selenoprotein**
**SEPHS2**	**selenophosphate synthetase 2**
**SEPSECS**	**Sep (O-phosphoserine) tRNA:Sec (selenocysteine) tRNA synthase**
**Sep-01**	**septin 1**
**Sep-11**	**septin 11**
**Sep-02**	**septin 2**
**Sep-07**	**septin 7**
**SEPT7P2**	**septin 7 pseudogene 2**
**Sep-09**	**septin 9**
**SERBP1**	**SERPINE1 mRNA binding protein 1**
**SERF2**	**small EDRK-rich factor 2**
**SERF2-C15ORF63**	**SERF2-C15orf63 readthrough**
**SERGEF**	**secretion regulating guanine nucleotide exchange factor**
**SERHL2**	**serine hydrolase-like 2**
**SERINC1**	**serine incorporator 1**
**SERINC3**	**serine incorporator 3**

SERINC4	serine incorporator 4
SERP1	stress-associated endoplasmic reticulum protein 1
SERPINB8	serpin peptidase inhibitor, clade B (ovalbumin), member 8
SERPINH1	serpin peptidase inhibitor, clade H (heat shock protein 47), member 1, (collagen binding protein 1)
SERPINI1	serpin peptidase inhibitor, clade I (neuroserpin), member 1
SERTAD1	SERTA domain containing 1
SERTAD2	SERTA domain containing 2
SERTAD3	SERTA domain containing 3
SESN1	sestrin 1
SESN3	sestrin 3
SET	SET nuclear proto-oncogene
SETBP1	SET binding protein 1
SETD1A	SET domain containing 1A
SETD1B	SET domain containing 1B
SETD2	SET domain containing 2
SETD4	SET domain containing 4
SETD5	SET domain containing 5
SETD6	SET domain containing 6
SETD9	SET domain containing 9
SETDB1	SET domain, bifurcated 1
SETDB2	SET domain, bifurcated 2
SETMAR	SET domain and mariner transposase fusion gene
SETX	senataxin
SF1	splicing factor 1
SF3A1	splicing factor 3a, subunit 1, 120kDa
SF3A2	splicing factor 3a, subunit 2, 66kDa
SF3A3	splicing factor 3a, subunit 3, 60kDa
SF3B1	splicing factor 3b, subunit 1, 155kDa
SF3B2	splicing factor 3b, subunit 2, 145kDa
SF3B3	splicing factor 3b, subunit 3, 130kDa
SF3B4	splicing factor 3b, subunit 4, 49kDa
SF3B5	splicing factor 3b, subunit 5, 10kDa
SF3B6	splicing factor 3b, subunit 6, 14kDa
SFI1	Sfi1 homolog, spindle assembly associated (yeast)
SFMBT2	Scm-like with four mbt domains 2
SFN	stratifin
SFPQ	splicing factor proline/glutamine-rich
SFR1	SWI5-dependent recombination repair 1
SFT2D1	SFT2 domain containing 1
SFT2D2	SFT2 domain containing 2
SFT2D3	SFT2 domain containing 3

**SFXN2**	**sideroflexin 2**
**SFXN4**	**sideroflexin 4**
**SFXN5**	**sideroflexin 5**
**SGK494**	**uncharacterized serine/threonine-protein kinase SgK494**
**SGOL1**	**shugoshin-like 1 (S. pombe)**
**SGOL2**	**shugoshin-like 2 (S. pombe)**
**SGPP2**	**sphingosine-1-phosphate phosphatase 2**
**SGSH**	**N-sulfoglucosamine sulfohydrolase**
**SGSM2**	**small G protein signaling modulator 2**
**SGSM3**	**small G protein signaling modulator 3**
**SGTA**	**small glutamine-rich tetratricopeptide repeat (TPR)-containing, alpha**
**SH2B1**	**SH2B adaptor protein 1**
**SH2B3**	**SH2B adaptor protein 3**
**SH2D2A**	**SH2 domain containing 2A**
**SH2D4B**	**SH2 domain containing 4B**
**SH3BGRL3**	**SH3 domain binding glutamate-rich protein like 3**
**SH3BP1**	**SH3-domain binding protein 1**
**SH3BP5L**	**SH3-binding domain protein 5-like**
**SH3D21**	**SH3 domain containing 21**
**SH3GL1**	**SH3-domain GRB2-like 1**
**SH3GLB1**	**SH3-domain GRB2-like endophilin B1**
**SH3KBP1**	**SH3-domain kinase binding protein 1**
**SH3RF1**	**SH3 domain containing ring finger 1**
**SHANK3**	**SH3 and multiple ankyrin repeat domains 3**
**SHARPIN**	**SHANK-associated RH domain interactor**
**SHC1**	**SHC (Src homology 2 domain containing) transforming protein 1**
**SHF**	**Src homology 2 domain containing F**
**SHISA8**	**shisa family member 8**
**SHMT2**	**serine hydroxymethyltransferase 2 (mitochondrial)**
**SHOC2**	**soc-2 suppressor of clear homolog (C. elegans)**
**SHPK**	**sedoheptulokinase**
**SHQ1**	**SHQ1, H/ACA ribonucleoprotein assembly factor**
**SHROOM1**	**shroom family member 1**
**SIAH2**	**siah E3 ubiquitin protein ligase 2**
**SIDT1**	**SID1 transmembrane family, member 1**
**SIGLEC10**	**sialic acid binding Ig-like lectin 10**
**SIGLEC5**	**sialic acid binding Ig-like lectin 5**
**SIGMAR1**	**sigma non-opioid intracellular receptor 1**
**SIK2**	**salt-inducible kinase 2**
**SIK3**	**SIK family kinase 3**
**SIN3A**	**SIN3 transcription regulator family member A**

**SIPA1**	**signal-induced proliferation-associated 1**
**SIPA1L3**	**signal-induced proliferation-associated 1 like 3**
**SIRT1**	**sirtuin 1**
**SIRT3**	**sirtuin 3**
**SIRT5**	**sirtuin 5**
**SIRT7**	**sirtuin 7**
**SIT1**	**signaling threshold regulating transmembrane adaptor 1**
**SIX5**	**SIX homeobox 5**
**SKA2**	**spindle and kinetochore associated complex subunit 2**
**SKA3**	**spindle and kinetochore associated complex subunit 3**
**SKAP2**	**src kinase associated phosphoprotein 2**
**SKIDA1**	**SKI/DACH domain containing 1**
**SKIL**	**SKI-like proto-oncogene**
**SKIV2L2**	**superkiller viralicidic activity 2-like 2 (S. cerevisiae)**
**SKP1**	**S-phase kinase-associated protein 1**
**SLAIN1**	**SLAIN motif family, member 1**
**SLAMF6**	**SLAM family member 6**
**SLAMF7**	**SLAM family member 7**
**SLBP**	**stem-loop binding protein**
**SLC10A7**	**solute carrier family 10, member 7**
**SLC11A2**	**solute carrier family 11 (proton-coupled divalent metal ion transporter), member 2**
**SLC12A2**	**solute carrier family 12 (sodium/potassium/chloride transporter), member 2**
**SLC12A4**	**solute carrier family 12 (potassium/chloride transporter), member 4**
**SLC12A6**	**solute carrier family 12 (potassium/chloride transporter), member 6**
**SLC15A3**	**solute carrier family 15 (oligopeptide transporter), member 3**
**SLC15A4**	**solute carrier family 15 (oligopeptide transporter), member 4**
**SLC16A1**	**solute carrier family 16 (monocarboxylate transporter), member 1**
**SLC16A13**	**solute carrier family 16, member 13**
**SLC16A14**	**solute carrier family 16, member 14**
**SLC16A6**	**solute carrier family 16, member 6**
**SLC17A5**	**solute carrier family 17 (acidic sugar transporter), member 5**
**SLC18B1**	**solute carrier family 18, subfamily B, member 1**
**SLC1A1**	**solute carrier family 1 (neuronal/epithelial high affinity glutamate transporter, system Xag), member 1**
**SLC1A4**	**solute carrier family 1 (glutamate/neutral amino acid transporter), member 4**
**SLC20A1**	**solute carrier family 20 (phosphate transporter), member 1**
**SLC22A20**	**solute carrier family 22, member 20**
**SLC23A1**	**solute carrier family 23 (ascorbic acid transporter), member 1**
**SLC25A10**	**solute carrier family 25 (mitochondrial carrier; dicarboxylate transporter), member 10**
**SLC25A11**	**solute carrier family 25 (mitochondrial carrier; oxoglutarate carrier), member 11**
**SLC25A13**	**solute carrier family 25 (aspartate/glutamate carrier), member 13**

**SLC25A16**	**solute carrier family 25 (mitochondrial carrier), member 16**
**SLC25A19**	**solute carrier family 25 (mitochondrial thiamine pyrophosphate carrier), member 19**
**SLC25A22**	**solute carrier family 25 (mitochondrial carrier: glutamate), member 22**
**SLC25A23**	**solute carrier family 25 (mitochondrial carrier; phosphate carrier), member 23**
**SLC25A25**	**solute carrier family 25 (mitochondrial carrier; phosphate carrier), member 25**
**SLC25A28**	**solute carrier family 25 (mitochondrial iron transporter), member 28**
**SLC25A3**	**solute carrier family 25 (mitochondrial carrier; phosphate carrier), member 3**
**SLC25A32**	**solute carrier family 25 (mitochondrial folate carrier), member 32**
**SLC25A33**	**solute carrier family 25 (pyrimidine nucleotide carrier), member 33**
**SLC25A36**	**solute carrier family 25 (pyrimidine nucleotide carrier), member 36**
**SLC25A38**	**solute carrier family 25, member 38**
**SLC25A39**	**solute carrier family 25, member 39**
**SLC25A4**	**solute carrier family 25 (mitochondrial carrier; adenine nucleotide translocator), member 4**
**SLC25A40**	**solute carrier family 25, member 40**
**SLC25A42**	**solute carrier family 25, member 42**
**SLC25A45**	**solute carrier family 25, member 45**
**SLC25A46**	**solute carrier family 25, member 46**
**SLC25A5**	**solute carrier family 25 (mitochondrial carrier; adenine nucleotide translocator), member 5**
**SLC25A5-AS1**	**SLC25A5 antisense RNA 1**
**SLC25A51**	**solute carrier family 25, member 51**
**SLC25A6**	**solute carrier family 25 (mitochondrial carrier; adenine nucleotide translocator), member 6**
**SLC26A11**	**solute carrier family 26 (anion exchanger), member 11**
**SLC26A2**	**solute carrier family 26 (anion exchanger), member 2**
**SLC26A6**	**solute carrier family 26 (anion exchanger), member 6**
**SLC27A3**	**solute carrier family 27 (fatty acid transporter), member 3**
**SLC27A5**	**solute carrier family 27 (fatty acid transporter), member 5**
**SLC2A1**	**solute carrier family 2 (facilitated glucose transporter), member 1**
**SLC2A5**	**solute carrier family 2 (facilitated glucose/fructose transporter), member 5**
**SLC30A6**	**solute carrier family 30 (zinc transporter), member 6**
**SLC31A1**	**solute carrier family 31 (copper transporter), member 1**
**SLC33A1**	**solute carrier family 33 (acetyl-CoA transporter), member 1**
**SLC35A1**	**solute carrier family 35 (CMP-sialic acid transporter), member A1**
**SLC35A2**	**solute carrier family 35 (UDP-galactose transporter), member A2**
**SLC35A3**	**solute carrier family 35 (UDP-N-acetylglucosamine (UDP-GlcNAc) transporter), member A3**
**SLC35A4**	**solute carrier family 35, member A4**
**SLC35A5**	**solute carrier family 35, member A5**
**SLC35B1**	**solute carrier family 35, member B1**

**SLC35B2**	**solute carrier family 35 (adenosine 3'-phospho 5'-phosphosulfate transporter), member B2**
**SLC35B3**	**solute carrier family 35 (adenosine 3'-phospho 5'-phosphosulfate transporter), member B3**
**SLC35C2**	**solute carrier family 35 (GDP-fucose transporter), member C2**
**SLC35E1**	**solute carrier family 35, member E1**
**SLC35E3**	**solute carrier family 35, member E3**
**SLC35F2**	**solute carrier family 35, member F2**
**SLC35F5**	**solute carrier family 35, member F5**
**SLC35F6**	**solute carrier family 35, member F6**
**SLC35G1**	**solute carrier family 35, member G1**
**SLC36A1**	**solute carrier family 36 (proton/amino acid symporter), member 1**
**SLC37A4**	**solute carrier family 37 (glucose-6-phosphate transporter), member 4**
**SLC38A1**	**solute carrier family 38, member 1**
**SLC38A10**	**solute carrier family 38, member 10**
**SLC38A2**	**solute carrier family 38, member 2**
**SLC38A6**	**solute carrier family 38, member 6**
**SLC39A1**	**solute carrier family 39 (zinc transporter), member 1**
**SLC39A10**	**solute carrier family 39 (zinc transporter), member 10**
**SLC39A13**	**solute carrier family 39 (zinc transporter), member 13**
**SLC39A3**	**solute carrier family 39 (zinc transporter), member 3**
**SLC39A6**	**solute carrier family 39 (zinc transporter), member 6**
**SLC39A8**	**solute carrier family 39 (zinc transporter), member 8**
**SLC39A9**	**solute carrier family 39, member 9**
**SLC3A2**	**solute carrier family 3 (amino acid transporter heavy chain), member 2**
**SLC43A3**	**solute carrier family 43, member 3**
**SLC44A1**	**solute carrier family 44 (choline transporter), member 1**
**SLC44A2**	**solute carrier family 44 (choline transporter), member 2**
**SLC45A2**	**solute carrier family 45, member 2**
**SLC4A1AP**	**solute carrier family 4 (anion exchanger), member 1, adaptor protein**
**SLC50A1**	**solute carrier family 50 (sugar efflux transporter), member 1**
**SLC5A3**	**solute carrier family 5 (sodium/myo-inositol cotransporter), member 3**
**SLC5A6**	**solute carrier family 5 (sodium/multivitamin and iodide cotransporter), member 6**
**SLC6A16**	**solute carrier family 6, member 16**
**SLC6A20**	**solute carrier family 6 (proline IMINO transporter), member 20**
**SLC6A6**	**solute carrier family 6 (neurotransmitter transporter), member 6**
**SLC7A1**	**solute carrier family 7 (cationic amino acid transporter, y+ system), member 1**
**SLC7A5**	**solute carrier family 7 (amino acid transporter light chain, L system), member 5**
**SLC7A5P2**	**solute carrier family 7 (amino acid transporter light chain, L system), member 5 pseudogene 2**
**SLC7A6OS**	**solute carrier family 7, member 6 opposite strand**
**SLC9A1**	**solute carrier family 9, subfamily A (NHE1, cation proton antiporter 1), member 1**

**SLC9A3R1**	**solute carrier family 9, subfamily A (NHE3, cation proton antiporter 3), member 3 regulator 1**
**SLC9A5**	**solute carrier family 9, subfamily A (NHE5, cation proton antiporter 5), member 5**
**SLC9A8**	**solute carrier family 9, subfamily A (NHE8, cation proton antiporter 8), member 8**
**SLC9B1**	**solute carrier family 9, subfamily B (NHA1, cation proton antiporter 1), member 1**
**SLFN12L**	**schlafen family member 12-like**
**SLFN13**	**schlafen family member 13**
**SLFNL1**	**schlafen-like 1**
**SLIRP**	**SRA stem-loop interacting RNA binding protein**
**SLMAP**	**sarcolemma associated protein**
**SLTM**	**SAFB-like, transcription modulator**
**SLU7**	**SLU7 splicing factor homolog (S. cerevisiae)**
**SLX4IP**	**SLX4 interacting protein**
**SMAD1**	**SMAD family member 1**
**SMAD4**	**SMAD family member 4**
**SMAP1**	**small ArfGAP 1**
**SMAP2**	**small ArfGAP2**
**SMARCA2**	**SWI/SNF related, matrix associated, actin dependent regulator of chromatin, subfamily a, member 2**
**SMARCA4**	**SWI/SNF related, matrix associated, actin dependent regulator of chromatin, subfamily a, member 4**
**SMARCA5**	**SWI/SNF related, matrix associated, actin dependent regulator of chromatin, subfamily a, member 5**
**SMARCAD1**	**SWI/SNF-related, matrix-associated actin-dependent regulator of chromatin, subfamily a, containing DEAD/H box 1**
**SMARCAL1**	**SWI/SNF related, matrix associated, actin dependent regulator of chromatin, subfamily a-like 1**
**SMARCC1**	**SWI/SNF related, matrix associated, actin dependent regulator of chromatin, subfamily c, member 1**
**SMARCC2**	**SWI/SNF related, matrix associated, actin dependent regulator of chromatin, subfamily c, member 2**
**SMARCD2**	**SWI/SNF related, matrix associated, actin dependent regulator of chromatin, subfamily d, member 2**
**SMARCE1**	**SWI/SNF related, matrix associated, actin dependent regulator of chromatin, subfamily e, member 1**
**SMC1A**	**structural maintenance of chromosomes 1A**
**SMC2**	**structural maintenance of chromosomes 2**
**SMC3**	**structural maintenance of chromosomes 3**
**SMC4**	**structural maintenance of chromosomes 4**
**SMCHD1**	**structural maintenance of chromosomes flexible hinge domain containing 1**
**SMCR8**	**Smith-Magenis syndrome chromosome region, candidate 8**
**SMDT1**	**single-pass membrane protein with aspartate-rich tail 1**
**SMEK1**	**SMEK homolog 1, suppressor of mek1 (Dictyostelium)**
**SMEK2**	**SMEK homolog 2, suppressor of mek1 (Dictyostelium)**

**SMG1**	**SMG1 phosphatidylinositol 3-kinase-related kinase**
**SMG5**	**SMG5 nonsense mediated mRNA decay factor**
**SMG7**	**SMG7 nonsense mediated mRNA decay factor**
**SMG7-AS1**	**SMG7 antisense RNA 1**
**SMG8**	**SMG8 nonsense mediated mRNA decay factor**
**SMG9**	**SMG9 nonsense mediated mRNA decay factor**
**SMIM12**	**small integral membrane protein 12**
**SMIM14**	**small integral membrane protein 14**
**SMIM15**	**small integral membrane protein 15**
**SMIM20**	**small integral membrane protein 20**
**SMIM4**	**small integral membrane protein 4**
**SMIM7**	**small integral membrane protein 7**
**SMPD1**	**sphingomyelin phosphodiesterase 1, acid lysosomal**
**SMPD4**	**sphingomyelin phosphodiesterase 4, neutral membrane (neutral sphingomyelinase-3)**
**SMYD4**	**SET and MYND domain containing 4**
**SNAP29**	**synaptosomal-associated protein, 29kDa**
**SNAP47**	**synaptosomal-associated protein, 47kDa**
**SNAPC3**	**small nuclear RNA activating complex, polypeptide 3, 50kDa**
**SNAPC4**	**small nuclear RNA activating complex, polypeptide 4, 190kDa**
**SNAPC5**	**small nuclear RNA activating complex, polypeptide 5, 19kDa**
**SNAPIN**	**SNAP-associated protein**
**SND1**	**staphylococcal nuclease and tudor domain containing 1**
**SNF8**	**SNF8, ESCRT-II complex subunit**
**SNHG1**	**small nucleolar RNA host gene 1**
**SNHG11**	**small nucleolar RNA host gene 11**
**SNHG12**	**small nucleolar RNA host gene 12**
**SNHG15**	**small nucleolar RNA host gene 15**
**SNHG20**	**small nucleolar RNA host gene 20**
**SNHG3**	**small nucleolar RNA host gene 3**
**SNHG4**	**small nucleolar RNA host gene 4**
**SNHG5**	**small nucleolar RNA host gene 5**
**SNHG6**	**small nucleolar RNA host gene 6**
**SNHG7**	**small nucleolar RNA host gene 7**
**SNHG8**	**small nucleolar RNA host gene 8**
**SNHG9**	**small nucleolar RNA host gene 9**
**SNN**	**stannin**
**SNORA1**	**small nucleolar RNA, H/ACA box 1**
**SNORA10**	**small nucleolar RNA, H/ACA box 10**
**SNORA13**	**small nucleolar RNA, H/ACA box 13**
**SNORA14B**	**small nucleolar RNA, H/ACA box 14B**
**SNORA16A**	**small nucleolar RNA, H/ACA box 16A**

**SNORA17**	**small nucleolar RNA, H/ACA box 17**
**SNORA18**	**small nucleolar RNA, H/ACA box 18**
**SNORA20**	**small nucleolar RNA, H/ACA box 20**
**SNORA21**	**small nucleolar RNA, H/ACA box 21**
**SNORA24**	**small nucleolar RNA, H/ACA box 24**
**SNORA25**	**small nucleolar RNA, H/ACA box 25**
**SNORA26**	**small nucleolar RNA, H/ACA box 26**
**SNORA27**	**small nucleolar RNA, H/ACA box 27**
**SNORA31**	**small nucleolar RNA, H/ACA box 31**
**SNORA32**	**small nucleolar RNA, H/ACA box 32**
**SNORA37**	**small nucleolar RNA, H/ACA box 37**
**SNORA4**	**small nucleolar RNA, H/ACA box 4**
**SNORA40**	**small nucleolar RNA, H/ACA box 40**
**SNORA43**	**small nucleolar RNA, H/ACA box 43**
**SNORA44**	**small nucleolar RNA, H/ACA box 44**
**SNORA45A**	**small nucleolar RNA, H/ACA box 45A**
**SNORA48**	**small nucleolar RNA, H/ACA box 48**
**SNORA52**	**small nucleolar RNA, H/ACA box 52**
**SNORA57**	**small nucleolar RNA, H/ACA box 57**
**SNORA6**	**small nucleolar RNA, H/ACA box 6**
**SNORA61**	**small nucleolar RNA, H/ACA box 61**
**SNORA63**	**small nucleolar RNA, H/ACA box 63**
**SNORA64**	**small nucleolar RNA, H/ACA box 64**
**SNORA65**	**small nucleolar RNA, H/ACA box 65**
**SNORA67**	**small nucleolar RNA, H/ACA box 67**
**SNORA68**	**small nucleolar RNA, H/ACA box 68**
**SNORA70**	**small nucleolar RNA, H/ACA box 70**
**SNORA71A**	**small nucleolar RNA, H/ACA box 71A**
**SNORA71B**	**small nucleolar RNA, H/ACA box 71B**
**SNORA71D**	**small nucleolar RNA, H/ACA box 71D**
**SNORA71E**	**small nucleolar RNA, H/ACA box 71E**
**SNORA72**	**small nucleolar RNA, H/ACA box 72**
**SNORA76C**	**small nucleolar RNA, H/ACA box 76C**
**SNORA78**	**small nucleolar RNA, H/ACA box 78**
**SNORA7A**	**small nucleolar RNA, H/ACA box 7A**
**SNORA8**	**small nucleolar RNA, H/ACA box 8**
**SNORA80B**	**small nucleolar RNA, H/ACA box 80B**
**SNORA81**	**small nucleolar RNA, H/ACA box 81**
**SNORA84**	**small nucleolar RNA, H/ACA box 84**
**SNORA9**	**small nucleolar RNA, H/ACA box 9**
**SNORD10**	**small nucleolar RNA, C/D box 10**

**SNORD101**	**small nucleolar RNA, C/D box 101**
**SNORD102**	**small nucleolar RNA, C/D box 102**
**SNORD104**	**small nucleolar RNA, C/D box 104**
**SNORD105**	**small nucleolar RNA, C/D box 105**
**SNORD105B**	**small nucleolar RNA, C/D box 105B**
**SNORD110**	**small nucleolar RNA, C/D box 110**
**SNORD12**	**small nucleolar RNA, C/D box 12**
**SNORD12B**	**small nucleolar RNA, C/D box 12B**
**SNORD12C**	**small nucleolar RNA, C/D box 12C**
**SNORD15A**	**small nucleolar RNA, C/D box 15A**
**SNORD15B**	**small nucleolar RNA, C/D box 15B**
**SNORD16**	**small nucleolar RNA, C/D box 16**
**SNORD17**	**small nucleolar RNA, C/D box 17**
**SNORD18A**	**small nucleolar RNA, C/D box 18A**
**SNORD18B**	**small nucleolar RNA, C/D box 18B**
**SNORD1A**	**small nucleolar RNA, C/D box 1A**
**SNORD1B**	**small nucleolar RNA, C/D box 1B**
**SNORD1C**	**small nucleolar RNA, C/D box 1C**
**SNORD2**	**small nucleolar RNA, C/D box 2**
**SNORD21**	**small nucleolar RNA, C/D box 21**
**SNORD22**	**small nucleolar RNA, C/D box 22**
**SNORD24**	**small nucleolar RNA, C/D box 24**
**SNORD25**	**small nucleolar RNA, C/D box 25**
**SNORD26**	**small nucleolar RNA, C/D box 26**
**SNORD27**	**small nucleolar RNA, C/D box 27**
**SNORD28**	**small nucleolar RNA, C/D box 28**
**SNORD29**	**small nucleolar RNA, C/D box 29**
**SNORD30**	**small nucleolar RNA, C/D box 30**
**SNORD31**	**small nucleolar RNA, C/D box 31**
**SNORD32A**	**small nucleolar RNA, C/D box 32A**
**SNORD33**	**small nucleolar RNA, C/D box 33**
**SNORD34**	**small nucleolar RNA, C/D box 34**
**SNORD35A**	**small nucleolar RNA, C/D box 35A**
**SNORD35B**	**small nucleolar RNA, C/D box 35B**
**SNORD36B**	**small nucleolar RNA, C/D box 36B**
**SNORD37**	**small nucleolar RNA, C/D box 37**
**SNORD38A**	**small nucleolar RNA, C/D box 38A**
**SNORD38B**	**small nucleolar RNA, C/D box 38B**
**SNORD42A**	**small nucleolar RNA, C/D box 42A**
**SNORD42B**	**small nucleolar RNA, C/D box 42B**
**SNORD43**	**small nucleolar RNA, C/D box 43**

**SNORD44**	**small nucleolar RNA, C/D box 44**
**SNORD45A**	**small nucleolar RNA, C/D box 45A**
**SNORD45B**	**small nucleolar RNA, C/D box 45B**
**SNORD45C**	**small nucleolar RNA, C/D box 45C**
**SNORD46**	**small nucleolar RNA, C/D box 46**
**SNORD47**	**small nucleolar RNA, C/D box 47**
**SNORD48**	**small nucleolar RNA, C/D box 48**
**SNORD49A**	**small nucleolar RNA, C/D box 49A**
**SNORD49B**	**small nucleolar RNA, C/D box 49B**
**SNORD4A**	**small nucleolar RNA, C/D box 4A**
**SNORD4B**	**small nucleolar RNA, C/D box 4B**
**SNORD5**	**small nucleolar RNA, C/D box 5**
**SNORD50A**	**small nucleolar RNA, C/D box 50A**
**SNORD50B**	**small nucleolar RNA, C/D box 50B**
**SNORD51**	**small nucleolar RNA, C/D box 51**
**SNORD52**	**small nucleolar RNA, C/D box 52**
**SNORD54**	**small nucleolar RNA, C/D box 54**
**SNORD55**	**small nucleolar RNA, C/D box 55**
**SNORD56B**	**small nucleolar RNA, C/D box 56B**
**SNORD57**	**small nucleolar RNA, C/D box 57**
**SNORD58A**	**small nucleolar RNA, C/D box 58A**
**SNORD58B**	**small nucleolar RNA, C/D box 58B**
**SNORD58C**	**small nucleolar RNA, C/D box 58C**
**SNORD59A**	**small nucleolar RNA, C/D box 59A**
**SNORD6**	**small nucleolar RNA, C/D box 6**
**SNORD60**	**small nucleolar RNA, C/D box 60**
**SNORD61**	**small nucleolar RNA, C/D box 61**
**SNORD65**	**small nucleolar RNA, C/D box 65**
**SNORD68**	**small nucleolar RNA, C/D box 68**
**SNORD72**	**small nucleolar RNA, C/D box 72**
**SNORD73A**	**small nucleolar RNA, C/D box 73A**
**SNORD74**	**small nucleolar RNA, C/D box 74**
**SNORD75**	**small nucleolar RNA, C/D box 75**
**SNORD76**	**small nucleolar RNA, C/D box 76**
**SNORD77**	**small nucleolar RNA, C/D box 77**
**SNORD81**	**small nucleolar RNA, C/D box 81**
**SNORD82**	**small nucleolar RNA, C/D box 82**
**SNORD83A**	**small nucleolar RNA, C/D box 83A**
**SNORD83B**	**small nucleolar RNA, C/D box 83B**
**SNORD88A**	**small nucleolar RNA, C/D box 88A**
**SNORD88B**	**small nucleolar RNA, C/D box 88B**

**SNORD88C**	**small nucleolar RNA, C/D box 88C**
**SNORD89**	**small nucleolar RNA, C/D box 89**
**SNORD93**	**small nucleolar RNA, C/D box 93**
**SNORD95**	**small nucleolar RNA, C/D box 95**
**SNORD96A**	**small nucleolar RNA, C/D box 96A**
**SNORD99**	**small nucleolar RNA, C/D box 99**
**SNRNP200**	**small nuclear ribonucleoprotein 200kDa (U5)**
**SNRNP25**	**small nuclear ribonucleoprotein 25kDa (U11/U12)**
**SNRNP27**	**small nuclear ribonucleoprotein 27kDa (U4/U6.U5)**
**SNRNP35**	**small nuclear ribonucleoprotein 35kDa (U11/U12)**
**SNRNP70**	**small nuclear ribonucleoprotein 70kDa (U1)**
**SNRPA**	**small nuclear ribonucleoprotein polypeptide A**
**SNRPA1**	**small nuclear ribonucleoprotein polypeptide A'**
**SNRPB**	**small nuclear ribonucleoprotein polypeptides B and B1**
**SNRPC**	**small nuclear ribonucleoprotein polypeptide C**
**SNRPD1**	**small nuclear ribonucleoprotein D1 polypeptide 16kDa**
**SNRPD2**	**small nuclear ribonucleoprotein D2 polypeptide 16.5kDa**
**SNRPD3**	**small nuclear ribonucleoprotein D3 polypeptide 18kDa**
**SNRPE**	**small nuclear ribonucleoprotein polypeptide E**
**SNRPG**	**small nuclear ribonucleoprotein polypeptide G**
**SNTB2**	**syntrophin, beta 2 (dystrophin-associated protein A1, 59kDa, basic component 2)**
**SNUPN**	**snurportin 1**
**SNX1**	**sorting nexin 1**
**SNX14**	**sorting nexin 14**
**SNX16**	**sorting nexin 16**
**SNX17**	**sorting nexin 17**
**SNX19**	**sorting nexin 19**
**SNX21**	**sorting nexin family member 21**
**SNX29**	**sorting nexin 29**
**SNX3**	**sorting nexin 3**
**SNX30**	**sorting nexin family member 30**
**SNX5**	**sorting nexin 5**
**SNX9**	**sorting nexin 9**
**SOCS1**	**suppressor of cytokine signaling 1**
**SOCS5**	**suppressor of cytokine signaling 5**
**SOD2**	**superoxide dismutase 2, mitochondrial**
**SON**	**SON DNA binding protein**
**SORL1**	**sortilin-related receptor, L(DLR class) A repeats containing**
**SOWAHD**	**sosondowah ankyrin repeat domain family member D**
**SOX12**	**SRY (sex determining region Y)-box 12**
**SOX15**	**SRY (sex determining region Y)-box 15**

**SOX4**	**SRY (sex determining region Y)-box 4**
**SOX9**	**SRY (sex determining region Y)-box 9**
**SP1**	**Sp1 transcription factor**
**SP100**	**SP100 nuclear antigen**
**SP140**	**SP140 nuclear body protein**
**SP3**	**Sp3 transcription factor**
**SP4**	**Sp4 transcription factor**
**SPACA5**	**sperm acrosome associated 5**
**SPAG1**	**sperm associated antigen 1**
**SPAG16**	**sperm associated antigen 16**
**SPAG5**	**sperm associated antigen 5**
**SPAG5-AS1**	**SPAG5 antisense RNA 1**
**SPAG7**	**sperm associated antigen 7**
**SPAG8**	**sperm associated antigen 8**
**SPAG9**	**sperm associated antigen 9**
**SPAST**	**spastin**
**SPATA2**	**spermatogenesis associated 2**
**SPATA24**	**spermatogenesis associated 24**
**SPATA2L**	**spermatogenesis associated 2-like**
**SPATA32**	**spermatogenesis associated 32**
**SPATA33**	**spermatogenesis associated 33**
**SPATA4**	**spermatogenesis associated 4**
**SPATA5**	**spermatogenesis associated 5**
**SPATA5L1**	**spermatogenesis associated 5-like 1**
**SPATA6L**	**spermatogenesis associated 6-like**
**SPATC1**	**spermatogenesis and centriole associated 1**
**SPATS2**	**spermatogenesis associated, serine-rich 2**
**SPCS2**	**signal peptidase complex subunit 2 homolog (S. cerevisiae)**
**SPDEF**	**SAM pointed domain containing ETS transcription factor**
**SPDYA**	**speedy/RINGO cell cycle regulator family member A**
**SPEF1**	**sperm flagellar 1**
**SPEN**	**spen family transcriptional repressor**
**SPERT**	**spermatid associated**
**SPG11**	**spastic paraplegia 11 (autosomal recessive)**
**SPG21**	**spastic paraplegia 21 (autosomal recessive, Mast syndrome)**
**SPHK2**	**sphingosine kinase 2**
**SPI1**	**Spi-1 proto-oncogene**
**SPIB**	**Spi-B transcription factor (Spi-1/PU.1 related)**
**SPICE1**	**spindle and centriole associated protein 1**
**SPN**	**sialophorin**
**SPNS1**	**spinster homolog 1 (Drosophila)**

**SPON2**	**spondin 2, extracellular matrix protein**
**SPOP**	**speckle-type POZ protein**
**SPPL2A**	**signal peptide peptidase like 2A**
**SPPL2B**	**signal peptide peptidase like 2B**
**SPRED2**	**sprouty-related, EVH1 domain containing 2**
**SPRN**	**shadow of prion protein homolog (zebrafish)**
**SPRTN**	**SprT-like N-terminal domain**
**SPRYD4**	**SPRY domain containing 4**
**SPSB2**	**splA/ryanodine receptor domain and SOCS box containing 2**
**SPTBN4**	**spectrin, beta, non-erythrocytic 4**
**SPTBN5**	**spectrin, beta, non-erythrocytic 5**
**SPTLC1**	**serine palmitoyltransferase, long chain base subunit 1**
**SPTY2D1**	**SPT2, Suppressor of Ty, domain containing 1 (S. cerevisiae)**
**SQRDL**	**sulfide quinone reductase-like (yeast)**
**SRA1**	**steroid receptor RNA activator 1**
**SRCAP**	**Snf2-related CREBBP activator protein**
**SRD5A1**	**steroid-5-alpha-reductase, alpha polypeptide 1 (3-oxo-5 alpha-steroid delta 4-dehydrogenase alpha 1)**
**SREBF1**	**sterol regulatory element binding transcription factor 1**
**SREBF2**	**sterol regulatory element binding transcription factor 2**
**SREK1**	**splicing regulatory glutamine/lysine-rich protein 1**
**SRF**	**serum response factor (c-fos serum response element-binding transcription factor)**
**SRFBP1**	**serum response factor binding protein 1**
**SRGAP1**	**SLIT-ROBO Rho GTPase activating protein 1**
**SRGN**	**serglycin**
**SRP14**	**signal recognition particle 14kDa (homologous Alu RNA binding protein)**
**SRP19**	**signal recognition particle 19kDa**
**SRP54**	**signal recognition particle 54kDa**
**SRP68**	**signal recognition particle 68kDa**
**SRP9**	**signal recognition particle 9kDa**
**SRPK1**	**SRSF protein kinase 1**
**SRRD**	**SRR1 domain containing**
**SRRM1**	**serine/arginine repetitive matrix 1**
**SRRM2**	**serine/arginine repetitive matrix 2**
**SRRM3**	**serine/arginine repetitive matrix 3**
**SRRT**	**serrate, RNA effector molecule**
**SRSF1**	**serine/arginine-rich splicing factor 1**
**SRSF10**	**serine/arginine-rich splicing factor 10**
**SRSF11**	**serine/arginine-rich splicing factor 11**
**SRSF2**	**serine/arginine-rich splicing factor 2**
**SRSF3**	**serine/arginine-rich splicing factor 3**
**SRSF4**	**serine/arginine-rich splicing factor 4**

**SRSF5**	**serine/arginine-rich splicing factor 5**
**SRSF6**	**serine/arginine-rich splicing factor 6**
**SRSF7**	**serine/arginine-rich splicing factor 7**
**SRSF8**	**serine/arginine-rich splicing factor 8**
**SRSF9**	**serine/arginine-rich splicing factor 9**
**SS18**	**synovial sarcoma translocation, chromosome 18**
**SSB**	**Sjogren syndrome antigen B (autoantigen La)**
**SSBP1**	**single-stranded DNA binding protein 1, mitochondrial**
**SSBP2**	**single-stranded DNA binding protein 2**
**SSBP3**	**single stranded DNA binding protein 3**
**SSH1**	**slingshot protein phosphatase 1**
**SSH2**	**slingshot protein phosphatase 2**
**SSNA1**	**Sjogren syndrome nuclear autoantigen 1**
**SSR1**	**signal sequence receptor, alpha**
**SSR3**	**signal sequence receptor, gamma (translocon-associated protein gamma)**
**SSSCA1**	**Sjogren syndrome/scleroderma autoantigen 1**
**SSU72**	**SSU72 RNA polymerase II CTD phosphatase homolog (S. cerevisiae)**
**ST13**	**suppression of tumorigenicity 13 (colon carcinoma) (Hsp70 interacting protein)**
**ST3GAL2**	**ST3 beta-galactoside alpha-2,3-sialyltransferase 2**
**ST3GAL3**	**ST3 beta-galactoside alpha-2,3-sialyltransferase 3**
**ST3GAL5**	**ST3 beta-galactoside alpha-2,3-sialyltransferase 5**
**ST3GAL6**	**ST3 beta-galactoside alpha-2,3-sialyltransferase 6**
**ST5**	**suppression of tumorigenicity 5**
**ST6GAL1**	**ST6 beta-galactosamide alpha-2,6-sialyltranferase 1**
**ST6GALNAC3**	**ST6 (alpha-N-acetyl-neuraminyl-2,3-beta-galactosyl-1,3)-N-acetylgalactosaminide alpha-2,6-sialyltransferase 3**
**ST6GALNAC6**	**ST6 (alpha-N-acetyl-neuraminyl-2,3-beta-galactosyl-1,3)-N-acetylgalactosaminide alpha-2,6-sialyltransferase 6**
**ST7-AS1**	**ST7 antisense RNA 1**
**ST7L**	**suppression of tumorigenicity 7 like**
**ST8SIA4**	**ST8 alpha-N-acetyl-neuraminide alpha-2,8-sialyltransferase 4**
**STAG1**	**stromal antigen 1**
**STAG3L4**	**stromal antigen 3-like 4 (pseudogene)**
**STAM**	**signal transducing adaptor molecule (SH3 domain and ITAM motif) 1**
**STAM2**	**signal transducing adaptor molecule (SH3 domain and ITAM motif) 2**
**STAMBPL1**	**STAM binding protein-like 1**
**STAP1**	**signal transducing adaptor family member 1**
**STARD10**	**StAR-related lipid transfer (START) domain containing 10**
**STARD3NL**	**STARD3 N-terminal like**
**STARD4**	**StAR-related lipid transfer (START) domain containing 4**
**STARD7**	**StAR-related lipid transfer (START) domain containing 7**
**STAT1**	**signal transducer and activator of transcription 1, 91kDa**

**STAT3**	**signal transducer and activator of transcription 3 (acute-phase response factor)**
**STAT4**	**signal transducer and activator of transcription 4**
**STAT5A**	**signal transducer and activator of transcription 5A**
**STAT5B**	**signal transducer and activator of transcription 5B**
**STAT6**	**signal transducer and activator of transcription 6, interleukin-4 induced**
**STAU2**	**staufen double-stranded RNA binding protein 2**
**STIL**	**SCL/TAL1 interrupting locus**
**STIM1**	**stromal interaction molecule 1**
**STIM2**	**stromal interaction molecule 2**
**STIP1**	**stress-induced phosphoprotein 1**
**STK11IP**	**serine/threonine kinase 11 interacting protein**
**STK16**	**serine/threonine kinase 16**
**STK17A**	**serine/threonine kinase 17a**
**STK17B**	**serine/threonine kinase 17b**
**STK3**	**serine/threonine kinase 3**
**STK36**	**serine/threonine kinase 36**
**STK38L**	**serine/threonine kinase 38 like**
**STK4**	**serine/threonine kinase 4**
**STK40**	**serine/threonine kinase 40**
**STOM**	**stomatin**
**STOML2**	**stomatin (EPB72)-like 2**
**STRADA**	**STE20-related kinase adaptor alpha**
**STRADB**	**STE20-related kinase adaptor beta**
**STRAP**	**serine/threonine kinase receptor associated protein**
**STRN**	**striatin, calmodulin binding protein**
**STT3A**	**STT3A, subunit of the oligosaccharyltransferase complex (catalytic)**
**STT3B**	**STT3B, subunit of the oligosaccharyltransferase complex (catalytic)**
**STUB1**	**STIP1 homology and U-box containing protein 1, E3 ubiquitin protein ligase**
**STX10**	**syntaxin 10**
**STX11**	**syntaxin 11**
**STX16**	**syntaxin 16**
**STX16-NPEPL1**	**STX16-NPEPL1 readthrough (NMD candidate)**
**STX18**	**syntaxin 18**
**STX4**	**syntaxin 4**
**STX6**	**syntaxin 6**
**STX7**	**syntaxin 7**
**STXBP3**	**syntaxin binding protein 3**
**STXBP4**	**syntaxin binding protein 4**
**STXBP5**	**syntaxin binding protein 5 (tomosyn)**
**STYK1**	**serine/threonine/tyrosine kinase 1**
**STYX**	**serine/threonine/tyrosine interacting protein**

**STYXL1**	**serine/threonine/tyrosine interacting-like 1**
**SUB1**	**SUB1 homolog (S. cerevisiae)**
**SUCLG2**	**succinate-CoA ligase, GDP-forming, beta subunit**
**SUDS3**	**suppressor of defective silencing 3 homolog (S. cerevisiae)**
**SUGCT**	**succinyl-CoA:glutarate-CoA transferase**
**SUGP1**	**SURP and G patch domain containing 1**
**SUGP2**	**SURP and G patch domain containing 2**
**SUGT1**	**SGT1, suppressor of G2 allele of SKP1 (S. cerevisiae)**
**SUGT1P3**	**SUGT1 pseudogene 3**
**SUMF1**	**sulfatase modifying factor 1**
**SUMF2**	**sulfatase modifying factor 2**
**SUMO1**	**small ubiquitin-like modifier 1**
**SUMO2**	**small ubiquitin-like modifier 2**
**SUOX**	**sulfite oxidase**
**SUPT16H**	**suppressor of Ty 16 homolog (S. cerevisiae)**
**SUPT20H**	**suppressor of Ty 20 homolog (S. cerevisiae)**
**SUPT4H1**	**suppressor of Ty 4 homolog 1 (S. cerevisiae)**
**SUPT5H**	**suppressor of Ty 5 homolog (S. cerevisiae)**
**SUPT6H**	**suppressor of Ty 6 homolog (S. cerevisiae)**
**SUPT7L**	**suppressor of Ty 7 (S. cerevisiae)-like**
**SUPV3L1**	**suppressor of var1, 3-like 1 (S. cerevisiae)**
**SURF6**	**surfeit 6**
**SUSD3**	**sushi domain containing 3**
**SVIP**	**small VCP/p97-interacting protein**
**SWSAP1**	**SWIM-type zinc finger 7 associated protein 1**
**SYCE2**	**synaptonemal complex central element protein 2**
**SYCP2**	**synaptonemal complex protein 2**
**SYF2**	**SYF2 pre-mRNA-splicing factor**
**SYNCRIP**	**synaptotagmin binding, cytoplasmic RNA interacting protein**
**SYNGAP1**	**synaptic Ras GTPase activating protein 1**
**SYNGR2**	**synaptogyrin 2**
**SYNRG**	**synergin, gamma**
**SYP**	**synaptophysin**
**SYPL1**	**synaptophysin-like 1**
**SYT5**	**synaptotagmin V**
**SYTL1**	**synaptotagmin-like 1**
**SYTL3**	**synaptotagmin-like 3**
**SYVN1**	**synovial apoptosis inhibitor 1, synoviolin**
**SZRD1**	**SUZ RNA binding domain containing 1**
**TAB1**	**TGF-beta activated kinase 1/MAP3K7 binding protein 1**
**TAB2**	**TGF-beta activated kinase 1/MAP3K7 binding protein 2**

**TACC3**	**transforming, acidic coiled-coil containing protein 3**
**TACO1**	**translational activator of mitochondrially encoded cytochrome c oxidase I**
**TACSTD2**	**tumor-associated calcium signal transducer 2**
**TADA1**	**transcriptional adaptor 1**
**TADA2A**	**transcriptional adaptor 2A**
**TADA3**	**transcriptional adaptor 3**
**TAF10**	**TAF10 RNA polymerase II, TATA box binding protein (TBP)-associated factor, 30kDa**
**TAF11**	**TAF11 RNA polymerase II, TATA box binding protein (TBP)-associated factor, 28kDa**
**TAF12**	**TAF12 RNA polymerase II, TATA box binding protein (TBP)-associated factor, 20kDa**
**TAF13**	**TAF13 RNA polymerase II, TATA box binding protein (TBP)-associated factor, 18kDa**
**TAF15**	**TAF15 RNA polymerase II, TATA box binding protein (TBP)-associated factor, 68kDa**
**TAF1D**	**TATA box binding protein (TBP)-associated factor, RNA polymerase I, D, 41kDa**
**TAF2**	**TAF2 RNA polymerase II, TATA box binding protein (TBP)-associated factor, 150kDa**
**TAF3**	**TAF3 RNA polymerase II, TATA box binding protein (TBP)-associated factor, 140kDa**
**TAF5**	**TAF5 RNA polymerase II, TATA box binding protein (TBP)-associated factor, 100kDa**
**TAF5L**	**TAF5-like RNA polymerase II, p300/CBP-associated factor (PCAF)-associated factor, 65kDa**
**TAF6**	**TAF6 RNA polymerase II, TATA box binding protein (TBP)-associated factor, 80kDa**
**TAF6L**	**TAF6-like RNA polymerase II, p300/CBP-associated factor (PCAF)-associated factor, 65kDa**
**TAF7**	**TAF7 RNA polymerase II, TATA box binding protein (TBP)-associated factor, 55kDa**
**TAF8**	**TAF8 RNA polymerase II, TATA box binding protein (TBP)-associated factor, 43kDa**
**TAF9**	**TAF9 RNA polymerase II, TATA box binding protein (TBP)-associated factor, 32kDa**
**TAGAP**	**T-cell activation RhoGTPase activating protein**
**TAGLN2**	**transgelin 2**
**TAMM41**	**TAM41, mitochondrial translocator assembly and maintenance protein, homolog (S. cerevisiae)**
**TANGO6**	**transport and golgi organization 6 homolog (Drosophila)**
**TANK**	**TRAF family member-associated NFKB activator**
**TAOK1**	**TAO kinase 1**
**TAOK2**	**TAO kinase 2**
**TAPBPL**	**TAP binding protein-like**
**TARBP1**	**TAR (HIV-1) RNA binding protein 1**
**TARBP2**	**TAR (HIV-1) RNA binding protein 2**
**TARDBP**	**TAR DNA binding protein**
**TARS**	**threonyl-tRNA synthetase**
**TARS2**	**threonyl-tRNA synthetase 2, mitochondrial (putative)**
**TARSL2**	**threonyl-tRNA synthetase-like 2**
**TAS1R1**	**taste receptor, type 1, member 1**
**TAS1R3**	**taste receptor, type 1, member 3**
**TATDN1**	**TatD DNase domain containing 1**
**TATDN3**	**TatD DNase domain containing 3**

**TAX1BP1**	**Tax1 (human T-cell leukemia virus type I) binding protein 1**
**TAX1BP3**	**Tax1 (human T-cell leukemia virus type I) binding protein 3**
**TBC1D1**	**TBC1 (tre-2/USP6, BUB2, cdc16) domain family, member 1**
**TBC1D10B**	**TBC1 domain family, member 10B**
**TBC1D13**	**TBC1 domain family, member 13**
**TBC1D14**	**TBC1 domain family, member 14**
**TBC1D15**	**TBC1 domain family, member 15**
**TBC1D17**	**TBC1 domain family, member 17**
**TBC1D19**	**TBC1 domain family, member 19**
**TBC1D22A**	**TBC1 domain family, member 22A**
**TBC1D22B**	**TBC1 domain family, member 22B**
**TBC1D23**	**TBC1 domain family, member 23**
**TBC1D25**	**TBC1 domain family, member 25**
**TBC1D30**	**TBC1 domain family, member 30**
**TBC1D32**	**TBC1 domain family, member 32**
**TBC1D4**	**TBC1 domain family, member 4**
**TBC1D5**	**TBC1 domain family, member 5**
**TBCA**	**tubulin folding cofactor A**
**TBCB**	**tubulin folding cofactor B**
**TBCC**	**tubulin folding cofactor C**
**TBCK**	**TBC1 domain containing kinase**
**TBK1**	**TANK-binding kinase 1**
**TBL1X**	**transducin (beta)-like 1X-linked**
**TBL3**	**transducin (beta)-like 3**
**TBPL1**	**TBP-like 1**
**TBRG4**	**transforming growth factor beta regulator 4**
**TBX6**	**T-box 6**
**TCAF1**	**TRPM8 channel-associated factor 1**
**TCAIM**	**T cell activation inhibitor, mitochondrial**
**TCAP**	**titin-cap**
**TCEA1**	**transcription elongation factor A (SII), 1**
**TCEA2**	**transcription elongation factor A (SII), 2**
**TCEB1**	**transcription elongation factor B (SIII), polypeptide 1 (15kDa, elongin C)**
**TCEB2**	**transcription elongation factor B (SIII), polypeptide 2 (18kDa, elongin B)**
**TCEB3**	**transcription elongation factor B (SIII), polypeptide 3 (110kDa, elongin A)**
**TCF12**	**transcription factor 12**
**TCF25**	**transcription factor 25 (basic helix-loop-helix)**
**TCF3**	**transcription factor 3**
**TCF7**	**transcription factor 7 (T-cell specific, HMG-box)**
**TCIRG1**	**T-cell, immune regulator 1, ATPase, H+ transporting, lysosomal V0 subunit A3**
**TCL1A**	**T-cell leukemia/lymphoma 1A**

**TCP1**	**t-complex 1**
**TCP10L**	**t-complex 10-like**
**TCTA**	**T-cell leukemia translocation altered**
**TCTE3**	**t-complex-associated-testis-expressed 3**
**TCTEX1D2**	**Tctex1 domain containing 2**
**TCTEX1D4**	**Tctex1 domain containing 4**
**TCTN1**	**tectonic family member 1**
**TCTN3**	**tectonic family member 3**
**TDG**	**thymine-DNA glycosylase**
**TDP2**	**tyrosyl-DNA phosphodiesterase 2**
**TDRD3**	**tudor domain containing 3**
**TDRD7**	**tudor domain containing 7**
**TEC**	**tec protein tyrosine kinase**
**TECR**	**trans-2,3-enoyl-CoA reductase**
**TEFM**	**transcription elongation factor, mitochondrial**
**TEN1**	**TEN1 CST complex subunit**
**TEN1-CDK3**	**TEN1-CDK3 readthrough (NMD candidate)**
**TERC**	**telomerase RNA component**
**TERF2**	**telomeric repeat binding factor 2**
**TERF2IP**	**telomeric repeat binding factor 2, interacting protein**
**TES**	**testin LIM domain protein**
**TESK1**	**testis-specific kinase 1**
**TESPA1**	**thymocyte expressed, positive selection associated 1**
**TET2**	**tet methylcytosine dioxygenase 2**
**TEX10**	**testis expressed 10**
**TEX14**	**testis expressed 14**
**TEX2**	**testis expressed 2**
**TEX264**	**testis expressed 264**
**TFAM**	**transcription factor A, mitochondrial**
**TFAP4**	**transcription factor AP-4 (activating enhancer binding protein 4)**
**TFB1M**	**transcription factor B1, mitochondrial**
**TFB2M**	**transcription factor B2, mitochondrial**
**TFCP2**	**transcription factor CP2**
**TFDP1**	**transcription factor Dp-1**
**TFEB**	**transcription factor EB**
**TFG**	**TRK-fused gene**
**TFPT**	**TCF3 (E2A) fusion partner (in childhood Leukemia)**
**TFRC**	**transferrin receptor**
**TGDS**	**TDP-glucose 4,6-dehydratase**
**TGFB1**	**transforming growth factor, beta 1**
**TGIF2**	**TGFB-induced factor homeobox 2**

**TGIF2-C20ORF24**	**TGIF2-C20orf24 readthrough**
**TGOLN2**	**trans-golgi network protein 2**
**TGS1**	**trimethylguanosine synthase 1**
**THADA**	**thyroid adenoma associated**
**THAP1**	**THAP domain containing, apoptosis associated protein 1**
**THAP2**	**THAP domain containing, apoptosis associated protein 2**
**THAP3**	**THAP domain containing, apoptosis associated protein 3**
**THAP5**	**THAP domain containing 5**
**THAP6**	**THAP domain containing 6**
**THAP7**	**THAP domain containing 7**
**THAP7-AS1**	**THAP7 antisense RNA 1**
**THAP8**	**THAP domain containing 8**
**THAP9**	**THAP domain containing 9**
**THEM4**	**thioesterase superfamily member 4**
**THG1L**	**tRNA-histidine guanylyltransferase 1-like (S. cerevisiae)**
**THNSL1**	**threonine synthase-like 1 (S. cerevisiae)**
**THOC1**	**THO complex 1**
**THOC2**	**THO complex 2**
**THOC5**	**THO complex 5**
**THOC7**	**THO complex 7**
**THOP1**	**thimet oligopeptidase 1**
**THRA**	**thyroid hormone receptor, alpha**
**THRAP3**	**thyroid hormone receptor associated protein 3**
**THSD1P1**	**thrombospondin, type I, domain containing 1 pseudogene 1**
**THUMPD1**	**THUMP domain containing 1**
**THUMPD2**	**THUMP domain containing 2**
**THUMPD3**	**THUMP domain containing 3**
**TIA1**	**TIA1 cytotoxic granule-associated RNA binding protein**
**TIAL1**	**TIA1 cytotoxic granule-associated RNA binding protein-like 1**
**TICAM1**	**toll-like receptor adaptor molecule 1**
**TICAM2**	**toll-like receptor adaptor molecule 2**
**TIFA**	**TRAF-interacting protein with forkhead-associated domain**
**TIGAR**	**TP53 induced glycolysis regulatory phosphatase**
**TIGD1**	**tigger transposable element derived 1**
**TIGD3**	**tigger transposable element derived 3**
**TIGD4**	**tigger transposable element derived 4**
**TIGD5**	**tigger transposable element derived 5**
**TIGD6**	**tigger transposable element derived 6**
**TIMELESS**	**timeless circadian clock**
**TIMM10**	**translocase of inner mitochondrial membrane 10 homolog (yeast)**
**TIMM10B**	**translocase of inner mitochondrial membrane 10 homolog B (yeast)**

**TIMM13**	**translocase of inner mitochondrial membrane 13 homolog (yeast)**
**TIMM17A**	**translocase of inner mitochondrial membrane 17 homolog A (yeast)**
**TIMM17B**	**translocase of inner mitochondrial membrane 17 homolog B (yeast)**
**TIMM21**	**translocase of inner mitochondrial membrane 21 homolog (yeast)**
**TIMM22**	**translocase of inner mitochondrial membrane 22 homolog (yeast)**
**TIMM44**	**translocase of inner mitochondrial membrane 44 homolog (yeast)**
**TIMM50**	**translocase of inner mitochondrial membrane 50 homolog (S. cerevisiae)**
**TIMM8A**	**translocase of inner mitochondrial membrane 8 homolog A (yeast)**
**TIMM8B**	**translocase of inner mitochondrial membrane 8 homolog B (yeast)**
**TIMM9**	**translocase of inner mitochondrial membrane 9 homolog (yeast)**
**TIMMDC1**	**translocase of inner mitochondrial membrane domain containing 1**
**TIMP1**	**TIMP metallopeptidase inhibitor 1**
**TINF2**	**TERF1 (TRF1)-interacting nuclear factor 2**
**TIPARP**	**TCDD-inducible poly(ADP-ribose) polymerase**
**TIPARP-AS1**	**TIPARP antisense RNA 1**
**TIPIN**	**TIMELESS interacting protein**
**TK1**	**thymidine kinase 1, soluble**
**TLCD1**	**TLC domain containing 1**
**TLDC1**	**TBC/LysM-associated domain containing 1**
**TLE3**	**transducin-like enhancer of split 3**
**TLE4**	**transducin-like enhancer of split 4**
**TLL2**	**tolloid-like 2**
**TLN1**	**talin 1**
**TLR1**	**toll-like receptor 1**
**TLR10**	**toll-like receptor 10**
**TLR6**	**toll-like receptor 6**
**TLR9**	**toll-like receptor 9**
**TM2D1**	**TM2 domain containing 1**
**TM2D3**	**TM2 domain containing 3**
**TM7SF2**	**transmembrane 7 superfamily member 2**
**TM7SF3**	**transmembrane 7 superfamily member 3**
**TM9SF1**	**transmembrane 9 superfamily member 1**
**TM9SF2**	**transmembrane 9 superfamily member 2**
**TM9SF4**	**transmembrane 9 superfamily protein member 4**
**TMA16**	**translation machinery associated 16 homolog (S. cerevisiae)**
**TMA7**	**translation machinery associated 7 homolog (S. cerevisiae)**
**TMBIM1**	**transmembrane BAX inhibitor motif containing 1**
**TMBIM4**	**transmembrane BAX inhibitor motif containing 4**
**TMBIM6**	**transmembrane BAX inhibitor motif containing 6**
**TMC6**	**transmembrane channel-like 6**
**TMC8**	**transmembrane channel-like 8**

**TMCO1**	**transmembrane and coiled-coil domains 1**
**TMCO3**	**transmembrane and coiled-coil domains 3**
**TMCO6**	**transmembrane and coiled-coil domains 6**
**TMED1**	**transmembrane emp24 protein transport domain containing 1**
**TMED2**	**transmembrane emp24 domain trafficking protein 2**
**TMED4**	**transmembrane emp24 protein transport domain containing 4**
**TMED5**	**transmembrane emp24 protein transport domain containing 5**
**TMED9**	**transmembrane emp24 protein transport domain containing 9**
**TMEM101**	**transmembrane protein 101**
**TMEM106A**	**transmembrane protein 106A**
**TMEM109**	**transmembrane protein 109**
**TMEM115**	**transmembrane protein 115**
**TMEM116**	**transmembrane protein 116**
**TMEM117**	**transmembrane protein 117**
**TMEM120B**	**transmembrane protein 120B**
**TMEM123**	**transmembrane protein 123**
**TMEM126A**	**transmembrane protein 126A**
**TMEM126B**	**transmembrane protein 126B**
**TMEM127**	**transmembrane protein 127**
**TMEM128**	**transmembrane protein 128**
**TMEM129**	**transmembrane protein 129, E3 ubiquitin protein ligase**
**TMEM131**	**transmembrane protein 131**
**TMEM132A**	**transmembrane protein 132A**
**TMEM134**	**transmembrane protein 134**
**TMEM135**	**transmembrane protein 135**
**TMEM138**	**transmembrane protein 138**
**TMEM141**	**transmembrane protein 141**
**TMEM147**	**transmembrane protein 147**
**TMEM14B**	**transmembrane protein 14B**
**TMEM14C**	**transmembrane protein 14C**
**TMEM150A**	**transmembrane protein 150A**
**TMEM154**	**transmembrane protein 154**
**TMEM156**	**transmembrane protein 156**
**TMEM167A**	**transmembrane protein 167A**
**TMEM167B**	**transmembrane protein 167B**
**TMEM168**	**transmembrane protein 168**
**TMEM17**	**transmembrane protein 17**
**TMEM170A**	**transmembrane protein 170A**
**TMEM173**	**transmembrane protein 173**
**TMEM175**	**transmembrane protein 175**
**TMEM179B**	**transmembrane protein 179B**

**TMEM18**	**transmembrane protein 18**
**TMEM183A**	**transmembrane protein 183A**
**TMEM183B**	**transmembrane protein 183B**
**TMEM184C**	**transmembrane protein 184C**
**TMEM185B**	**transmembrane protein 185B**
**TMEM187**	**transmembrane protein 187**
**TMEM189**	**transmembrane protein 189**
**TMEM189-UBE2V1**	**TMEM189-UBE2V1 readthrough**
**TMEM19**	**transmembrane protein 19**
**TMEM194B**	**transmembrane protein 194B**
**TMEM198B**	**transmembrane protein 198B, pseudogene**
**TMEM203**	**transmembrane protein 203**
**TMEM206**	**transmembrane protein 206**
**TMEM208**	**transmembrane protein 208**
**TMEM209**	**transmembrane protein 209**
**TMEM217**	**transmembrane protein 217**
**TMEM222**	**transmembrane protein 222**
**TMEM223**	**transmembrane protein 223**
**TMEM230**	**transmembrane protein 230**
**TMEM237**	**transmembrane protein 237**
**TMEM238**	**transmembrane protein 238**
**TMEM242**	**transmembrane protein 242**
**TMEM243**	**transmembrane protein 243, mitochondrial**
**TMEM248**	**transmembrane protein 248**
**TMEM255A**	**transmembrane protein 255A**
**TMEM258**	**transmembrane protein 258**
**TMEM259**	**transmembrane protein 259**
**TMEM260**	**transmembrane protein 260**
**TMEM30A**	**transmembrane protein 30A**
**TMEM33**	**transmembrane protein 33**
**TMEM38A**	**transmembrane protein 38A**
**TMEM39A**	**transmembrane protein 39A**
**TMEM39B**	**transmembrane protein 39B**
**TMEM41A**	**transmembrane protein 41A**
**TMEM41B**	**transmembrane protein 41B**
**TMEM42**	**transmembrane protein 42**
**TMEM43**	**transmembrane protein 43**
**TMEM44**	**transmembrane protein 44**
**TMEM5**	**transmembrane protein 5**
**TMEM60**	**transmembrane protein 60**
**TMEM62**	**transmembrane protein 62**

**TMEM63B**	**transmembrane protein 63B**
**TMEM64**	**transmembrane protein 64**
**TMEM68**	**transmembrane protein 68**
**TMEM69**	**transmembrane protein 69**
**TMEM79**	**transmembrane protein 79**
**TMEM80**	**transmembrane protein 80**
**TMEM87A**	**transmembrane protein 87A**
**TMEM87B**	**transmembrane protein 87B**
**TMEM88**	**transmembrane protein 88**
**TMEM91**	**transmembrane protein 91**
**TMEM9B**	**TMEM9 domain family, member B**
**TMF1**	**TATA element modulatory factor 1**
**TMPO**	**thymopoietin**
**TMPPE**	**transmembrane protein with metallophosphoesterase domain**
**TMSB10**	**thymosin beta 10**
**TMSB15B**	**thymosin beta 15B**
**TMSB4X**	**thymosin beta 4, X-linked**
**TMUB2**	**transmembrane and ubiquitin-like domain containing 2**
**TMX2**	**thioredoxin-related transmembrane protein 2**
**TMX2-CTNND1**	**TMX2-CTNND1 readthrough (NMD candidate)**
**TMX3**	**thioredoxin-related transmembrane protein 3**
**TNFAIP3**	**tumor necrosis factor, alpha-induced protein 3**
**TNFAIP8**	**tumor necrosis factor, alpha-induced protein 8**
**TNFAIP8L1**	**tumor necrosis factor, alpha-induced protein 8-like 1**
**TNFAIP8L2**	**tumor necrosis factor, alpha-induced protein 8-like 2**
**TNFAIP8L2-SCNM1**	**TNFAIP8L2-SCNM1 readthrough**
**TNFRSF10A**	**tumor necrosis factor receptor superfamily, member 10a**
**TNFRSF10B**	**tumor necrosis factor receptor superfamily, member 10b**
**TNFRSF13B**	**tumor necrosis factor receptor superfamily, member 13B**
**TNFRSF13C**	**tumor necrosis factor receptor superfamily, member 13C**
**TNFRSF14**	**tumor necrosis factor receptor superfamily, member 14**
**TNFRSF17**	**tumor necrosis factor receptor superfamily, member 17**
**TNFRSF19**	**tumor necrosis factor receptor superfamily, member 19**
**TNFRSF1B**	**tumor necrosis factor receptor superfamily, member 1B**
**TNFRSF4**	**tumor necrosis factor receptor superfamily, member 4**
**TNFRSF6B**	**tumor necrosis factor receptor superfamily, member 6b, decoy**
**TNFRSF8**	**tumor necrosis factor receptor superfamily, member 8**
**TNFSF13B**	**tumor necrosis factor (ligand) superfamily, member 13b**
**TNFSF14**	**tumor necrosis factor (ligand) superfamily, member 14**
**TNFSF4**	**tumor necrosis factor (ligand) superfamily, member 4**
**TNFSF9**	**tumor necrosis factor (ligand) superfamily, member 9**

**TNIK**	**TRAF2 and NCK interacting kinase**
**TNIP1**	**TNFAIP3 interacting protein 1**
**TNIP3**	**TNFAIP3 interacting protein 3**
**TNK2**	**tyrosine kinase, non-receptor, 2**
**TNKS1BP1**	**tankyrase 1 binding protein 1, 182kDa**
**TNPO1**	**transportin 1**
**TNPO2**	**transportin 2**
**TNPO3**	**transportin 3**
**TNRC6B**	**trinucleotide repeat containing 6B**
**TOB1**	**transducer of ERBB2, 1**
**TOB2**	**transducer of ERBB2, 2**
**TOE1**	**target of EGR1, member 1 (nuclear)**
**TOM1**	**target of myb1 (chicken)**
**TOMM20**	**translocase of outer mitochondrial membrane 20 homolog (yeast)**
**TOMM20L**	**translocase of outer mitochondrial membrane 20 homolog (yeast)-like**
**TOMM22**	**translocase of outer mitochondrial membrane 22 homolog (yeast)**
**TOMM40**	**translocase of outer mitochondrial membrane 40 homolog (yeast)**
**TOMM5**	**translocase of outer mitochondrial membrane 5 homolog (yeast)**
**TOMM6**	**translocase of outer mitochondrial membrane 6 homolog (yeast)**
**TOMM7**	**translocase of outer mitochondrial membrane 7 homolog (yeast)**
**TOMM70A**	**translocase of outer mitochondrial membrane 70 homolog A (S. cerevisiae)**
**TOP2B**	**topoisomerase (DNA) II beta 180kDa**
**TOP3A**	**topoisomerase (DNA) III alpha**
**TOP3B**	**topoisomerase (DNA) III beta**
**TOPBP1**	**topoisomerase (DNA) II binding protein 1**
**TOPORS**	**topoisomerase I binding, arginine/serine-rich, E3 ubiquitin protein ligase**
**TOR1A**	**torsin family 1, member A (torsin A)**
**TOR1AIP1**	**torsin A interacting protein 1**
**TOR2A**	**torsin family 2, member A**
**TOR3A**	**torsin family 3, member A**
**TOR4A**	**torsin family 4, member A**
**TP53**	**tumor protein p53**
**TP53I13**	**tumor protein p53 inducible protein 13**
**TP53I3**	**tumor protein p53 inducible protein 3**
**TPCN1**	**two pore segment channel 1**
**TPD52**	**tumor protein D52**
**TPD52L2**	**tumor protein D52-like 2**
**TPGS1**	**tubulin polyglutamylase complex subunit 1**
**TPI1**	**triosephosphate isomerase 1**
**TPI1P2**	**triosephosphate isomerase 1 pseudogene 2**
**TPK1**	**thiamin pyrophosphokinase 1**

**TPM4**	**tropomyosin 4**
**TPMT**	**thiopurine S-methyltransferase**
**TPP1**	**tripeptidyl peptidase I**
**TPR**	**translocated promoter region, nuclear basket protein**
**TPRA1**	**transmembrane protein, adipocyte asscociated 1**
**TPRKB**	**TP53RK binding protein**
**TPRN**	**taperin**
**TPT1**	**tumor protein, translationally-controlled 1**
**TPX2**	**TPX2, microtubule-associated**
**TRA2A**	**transformer 2 alpha homolog (Drosophila)**
**TRA2B**	**transformer 2 beta homolog (Drosophila)**
**TRADD**	**TNFRSF1A-associated via death domain**
**TRAF1**	**TNF receptor-associated factor 1**
**TRAF2**	**TNF receptor-associated factor 2**
**TRAF3**	**TNF receptor-associated factor 3**
**TRAF3IP2**	**TRAF3 interacting protein 2**
**TRAF3IP2-AS1**	**TRAF3IP2 antisense RNA 1**
**TRAF4**	**TNF receptor-associated factor 4**
**TRAF6**	**TNF receptor-associated factor 6, E3 ubiquitin protein ligase**
**TRAF7**	**TNF receptor-associated factor 7, E3 ubiquitin protein ligase**
**TRAK2**	**trafficking protein, kinesin binding 2**
**TRAM1**	**translocation associated membrane protein 1**
**TRAPPC12**	**trafficking protein particle complex 12**
**TRAPPC13**	**trafficking protein particle complex 13**
**TRAPPC2**	**trafficking protein particle complex 2**
**TRAPPC2P1**	
**TRAPPC3**	**trafficking protein particle complex 3**
**TRAPPC4**	**trafficking protein particle complex 4**
**TRAPPC5**	**trafficking protein particle complex 5**
**TRAPPC6B**	**trafficking protein particle complex 6B**
**TRAPPC8**	**trafficking protein particle complex 8**
**TRERF1**	**transcriptional regulating factor 1**
**TREX1**	**three prime repair exonuclease 1**
**TRIAP1**	**TP53 regulated inhibitor of apoptosis 1**
**TRIB1**	**tribbles pseudokinase 1**
**TRIM13**	**tripartite motif containing 13**
**TRIM14**	**tripartite motif containing 14**
**TRIM23**	**tripartite motif containing 23**
**TRIM25**	**tripartite motif containing 25**
**TRIM28**	**tripartite motif containing 28**
**TRIM33**	**tripartite motif containing 33**

**TRIM35**	**tripartite motif containing 35**
**TRIM37**	**tripartite motif containing 37**
**TRIM38**	**tripartite motif containing 38**
**TRIM4**	**tripartite motif containing 4**
**TRIM41**	**tripartite motif containing 41**
**TRIM46**	**tripartite motif containing 46**
**TRIM5**	**tripartite motif containing 5**
**TRIM52**	**tripartite motif containing 52**
**TRIM59**	**tripartite motif containing 59**
**TRIM61**	**tripartite motif containing 61**
**TRIM65**	**tripartite motif containing 65**
**TRIM7**	**tripartite motif containing 7**
**TRIM8**	**tripartite motif containing 8**
**TRIO**	**trio Rho guanine nucleotide exchange factor**
**TRIP11**	**thyroid hormone receptor interactor 11**
**TRIP12**	**thyroid hormone receptor interactor 12**
**TRIP4**	**thyroid hormone receptor interactor 4**
**TRIP6**	**thyroid hormone receptor interactor 6**
**TRIQK**	**triple QxxK/R motif containing**
**TRMT1**	**tRNA methyltransferase 1 homolog (S. cerevisiae)**
**TRMT10A**	**tRNA methyltransferase 10 homolog A (S. cerevisiae)**
**TRMT10C**	**tRNA methyltransferase 10 homolog C (S. cerevisiae)**
**TRMT11**	**tRNA methyltransferase 11 homolog (S. cerevisiae)**
**TRMT112**	**tRNA methyltransferase 11-2 homolog (S. cerevisiae)**
**TRMT2A**	**tRNA methyltransferase 2 homolog A (S. cerevisiae)**
**TRMT44**	**tRNA methyltransferase 44 homolog (S. cerevisiae)**
**TRMT5**	**tRNA methyltransferase 5**
**TRMT6**	**tRNA methyltransferase 6**
**TRMT61A**	**tRNA methyltransferase 61A**
**TRMT61B**	**tRNA methyltransferase 61B**
**TRNAU1AP**	**tRNA selenocysteine 1 associated protein 1**
**TRNT1**	**tRNA nucleotidyl transferase, CCA-adding, 1**
**TROAP**	**trophinin associated protein**
**TROVE2**	**TROVE domain family, member 2**
**TRPC4AP**	**transient receptor potential cation channel, subfamily C, member 4 associated protein**
**TRPM7**	**transient receptor potential cation channel, subfamily M, member 7**
**TRPT1**	**tRNA phosphotransferase 1**
**TRPV2**	**transient receptor potential cation channel, subfamily V, member 2**
**TRPV3**	**transient receptor potential cation channel, subfamily V, member 3**
**TRRAP**	**transformation/transcription domain-associated protein**
**TRUB2**	**TruB pseudouridine (psi) synthase family member 2**

**TSACC**	**TSSK6 activating co-chaperone**
**TSC22D1**	**TSC22 domain family, member 1**
**TSC22D2**	**TSC22 domain family, member 2**
**TSC22D4**	**TSC22 domain family, member 4**
**TSEN15**	**TSEN15 tRNA splicing endonuclease subunit**
**TSEN2**	**TSEN2 tRNA splicing endonuclease subunit**
**TSEN34**	**TSEN34 tRNA splicing endonuclease subunit**
**TSFM**	**Ts translation elongation factor, mitochondrial**
**TSG101**	**tumor susceptibility 101**
**TSGA10**	**testis specific, 10**
**TSN**	**translin**
**TSNAX**	**translin-associated factor X**
**TSNAX-DISC1**	**TSNAX-DISC1 readthrough (NMD candidate)**
**TSPAN17**	**tetraspanin 17**
**TSPAN31**	**tetraspanin 31**
**TSPAN4**	**tetraspanin 4**
**TSPY26P**	**testis specific protein, Y-linked 26, pseudogene**
**TSPYL1**	**TSPY-like 1**
**TSPYL2**	**TSPY-like 2**
**TSPYL4**	**TSPY-like 4**
**TSR1**	**TSR1, 20S rRNA accumulation, homolog (S. cerevisiae)**
**TSR2**	**TSR2, 20S rRNA accumulation, homolog (S. cerevisiae)**
**TSSC1**	**tumor suppressing subtransferable candidate 1**
**TSSC4**	**tumor suppressing subtransferable candidate 4**
**TSSK3**	**testis-specific serine kinase 3**
**TSSK6**	**testis-specific serine kinase 6**
**TSTA3**	**tissue specific transplantation antigen P35B**
**TSTD1**	**thiosulfate sulfurtransferase (rhodanese)-like domain containing 1**
**TSTD2**	**thiosulfate sulfurtransferase (rhodanese)-like domain containing 2**
**TTC1**	**tetratricopeptide repeat domain 1**
**TTC14**	**tetratricopeptide repeat domain 14**
**TTC17**	**tetratricopeptide repeat domain 17**
**TTC19**	**tetratricopeptide repeat domain 19**
**TTC21A**	**tetratricopeptide repeat domain 21A**
**TTC21B**	**tetratricopeptide repeat domain 21B**
**TTC23**	**tetratricopeptide repeat domain 23**
**TTC26**	**tetratricopeptide repeat domain 26**
**TTC27**	**tetratricopeptide repeat domain 27**
**TTC28-AS1**	**TTC28 antisense RNA 1**
**TTC30B**	**tetratricopeptide repeat domain 30B**
**TTC31**	**tetratricopeptide repeat domain 31**

**TTC32**	**tetratricopeptide repeat domain 32**
**TTC33**	**tetratricopeptide repeat domain 33**
**TTC37**	**tetratricopeptide repeat domain 37**
**TTC39C**	**tetratricopeptide repeat domain 39C**
**TTC4**	**tetratricopeptide repeat domain 4**
**TTC41P**	**tetratricopeptide repeat domain 41, pseudogene**
**TTC7A**	**tetratricopeptide repeat domain 7A**
**TTC9**	**tetratricopeptide repeat domain 9**
**TTC9C**	**tetratricopeptide repeat domain 9C**
**TTF2**	**transcription termination factor, RNA polymerase II**
**TTI1**	**TELO2 interacting protein 1**
**TTI2**	**TELO2 interacting protein 2**
**TTK**	**TTK protein kinase**
**TTLL12**	**tubulin tyrosine ligase-like family member 12**
**TTLL3**	**tubulin tyrosine ligase-like family member 3**
**TTLL5**	**tubulin tyrosine ligase-like family member 5**
**TTPAL**	**tocopherol (alpha) transfer protein-like**
**TTYH1**	**tweety family member 1**
**TTYH2**	**tweety family member 2**
**TTYH3**	**tweety family member 3**
**TUBA1A**	**tubulin, alpha 1a**
**TUBA1B**	**tubulin, alpha 1b**
**TUBA1C**	**tubulin, alpha 1c**
**TUBB4B**	**tubulin, beta 4B class IVb**
**TUBD1**	**tubulin, delta 1**
**TUBG1**	**tubulin, gamma 1**
**TUBGCP2**	**tubulin, gamma complex associated protein 2**
**TUBGCP3**	**tubulin, gamma complex associated protein 3**
**TUBGCP6**	**tubulin, gamma complex associated protein 6**
**TUFM**	**Tu translation elongation factor, mitochondrial**
**TULP3**	**tubby like protein 3**
**TUSC2**	**tumor suppressor candidate 2**
**TUT1**	**terminal uridylyl transferase 1, U6 snRNA-specific**
**TVP23B**	**trans-golgi network vesicle protein 23 homolog B (S. cerevisiae)**
**TWF2**	**twinfilin actin binding protein 2**
**TWISTNB**	**TWIST neighbor**
**TXLNA**	**taxilin alpha**
**TXLNB**	**taxilin beta**
**TXN**	**thioredoxin**
**TXN2**	**thioredoxin 2**
**TXNDC11**	**thioredoxin domain containing 11**

**TXNDC12**	**thioredoxin domain containing 12 (endoplasmic reticulum)**
**TXNDC15**	**thioredoxin domain containing 15**
**TXNDC17**	**thioredoxin domain containing 17**
**TXNDC9**	**thioredoxin domain containing 9**
**TXNIP**	**thioredoxin interacting protein**
**TXNL1**	**thioredoxin-like 1**
**TYK2**	**tyrosine kinase 2**
**TYMP**	**thymidine phosphorylase**
**TYMS**	**thymidylate synthetase**
**TYMSOS**	**TYMS opposite strand**
**TYSND1**	**trypsin domain containing 1**
**TYW1**	**tRNA-yW synthesizing protein 1 homolog (S. cerevisiae)**
**TYW3**	**tRNA-yW synthesizing protein 3 homolog (S. cerevisiae)**
**TYW5**	**tRNA-yW synthesizing protein 5**
**U2AF2**	**U2 small nuclear RNA auxiliary factor 2**
**U2SURP**	**U2 snRNP-associated SURP domain containing**
**UBA1**	**ubiquitin-like modifier activating enzyme 1**
**UBA52**	**ubiquitin A-52 residue ribosomal protein fusion product 1**
**UBA6**	**ubiquitin-like modifier activating enzyme 6**
**UBAC2**	**UBA domain containing 2**
**UBAC2-AS1**	**UBAC2 antisense RNA 1**
**UBALD1**	**UBA-like domain containing 1**
**UBALD2**	**UBA-like domain containing 2**
**UBAP1**	**ubiquitin associated protein 1**
**UBAP2**	**ubiquitin associated protein 2**
**UBAP2L**	**ubiquitin associated protein 2-like**
**UBB**	**ubiquitin B**
**UBC**	**ubiquitin C**
**UBE2A**	**ubiquitin-conjugating enzyme E2A**
**UBE2B**	**ubiquitin-conjugating enzyme E2B**
**UBE2C**	**ubiquitin-conjugating enzyme E2C**
**UBE2D2**	**ubiquitin-conjugating enzyme E2D 2**
**UBE2D3**	**ubiquitin-conjugating enzyme E2D 3**
**UBE2D4**	**ubiquitin-conjugating enzyme E2D 4 (putative)**
**UBE2G1**	**ubiquitin-conjugating enzyme E2G 1**
**UBE2G2**	**ubiquitin-conjugating enzyme E2G 2**
**UBE2I**	**ubiquitin-conjugating enzyme E2I**
**UBE2K**	**ubiquitin-conjugating enzyme E2K**
**UBE2L3**	**ubiquitin-conjugating enzyme E2L 3**
**UBE2M**	**ubiquitin-conjugating enzyme E2M**
**UBE2N**	**ubiquitin-conjugating enzyme E2N**

UBE2Q1	ubiquitin-conjugating enzyme E2Q family member 1
UBE2QL1	ubiquitin-conjugating enzyme E2Q family-like 1
UBE2R2	ubiquitin-conjugating enzyme E2R 2
UBE2S	ubiquitin-conjugating enzyme E2S
UBE2V2	ubiquitin-conjugating enzyme E2 variant 2
UBE2Z	ubiquitin-conjugating enzyme E2Z
UBE3D	ubiquitin protein ligase E3D
UBE4A	ubiquitination factor E4A
UBFD1	ubiquitin family domain containing 1
UBL5	ubiquitin-like 5
UBL7	ubiquitin-like 7
UBLCP1	ubiquitin-like domain containing CTD phosphatase 1
UBOX5	U-box domain containing 5
UBP1	upstream binding protein 1 (LBP-1a)
UBQLN1	ubiquilin 1
UBQLN4	ubiquilin 4
UBR1	ubiquitin protein ligase E3 component n-recognin 1
UBR2	ubiquitin protein ligase E3 component n-recognin 2
UBR4	ubiquitin protein ligase E3 component n-recognin 4
UBR5	ubiquitin protein ligase E3 component n-recognin 5
UBTD1	ubiquitin domain containing 1
UBTF	upstream binding transcription factor, RNA polymerase I
UBXN1	UBX domain protein 1
UBXN2A	UBX domain protein 2A
UBXN2B	UBX domain protein 2B
UBXN4	UBX domain protein 4
UBXN7	UBX domain protein 7
UBXN8	UBX domain protein 8
UCHL3	ubiquitin carboxyl-terminal esterase L3 (ubiquitin thiolesterase)
UCHL5	ubiquitin carboxyl-terminal hydrolase L5
UCKL1	uridine-cytidine kinase 1-like 1
UCP2	uncoupling protein 2 (mitochondrial, proton carrier)
UEVLD	UEV and lactate/malate dehyrogenase domains
UFC1	ubiquitin-fold modifier conjugating enzyme 1
UFL1	UFM1-specific ligase 1
UFSP1	UFM1-specific peptidase 1 (non-functional)
UFSP2	UFM1-specific peptidase 2
UGCG	UDP-glucose ceramide glucosyltransferase
UGDH	UDP-glucose 6-dehydrogenase
UGGT1	UDP-glucose glycoprotein glucosyltransferase 1
UGP2	UDP-glucose pyrophosphorylase 2

UHRF1BP1	UHRF1 binding protein 1
UHRF1BP1L	UHRF1 binding protein 1-like
UHRF2	ubiquitin-like with PHD and ring finger domains 2, E3 ubiquitin protein ligase
UIMC1	ubiquitin interaction motif containing 1
ULK3	unc-51 like kinase 3
ULK4	unc-51 like kinase 4
UMODL1	uromodulin-like 1
UMPS	uridine monophosphate synthetase
UNC45A	unc-45 homolog A (C. elegans)
UNC50	unc-50 homolog (C. elegans)
UNK	unkempt family zinc finger
UPF2	UPF2 regulator of nonsense transcripts homolog (yeast)
UPF3A	UPF3 regulator of nonsense transcripts homolog A (yeast)
UQCC1	ubiquinol-cytochrome c reductase complex assembly factor 1
UQCC2	ubiquinol-cytochrome c reductase complex assembly factor 2
UQCC3	ubiquinol-cytochrome c reductase complex assembly factor 3
UQCR11	ubiquinol-cytochrome c reductase, complex III subunit XI
UQCRC1	ubiquinol-cytochrome c reductase core protein I
UQCRC2	ubiquinol-cytochrome c reductase core protein II
UQCRH	ubiquinol-cytochrome c reductase hinge protein
UQCRQ	ubiquinol-cytochrome c reductase, complex III subunit VII, 9.5kDa
URB2	URB2 ribosome biogenesis 2 homolog (S. cerevisiae)
URGCP	upregulator of cell proliferation
URM1	ubiquitin related modifier 1
UROS	uroporphyrinogen III synthase
USE1	unconventional SNARE in the ER 1 homolog (S. cerevisiae)
USF1	upstream transcription factor 1
USF2	upstream transcription factor 2, c-fos interacting
USO1	USO1 vesicle transport factor
USP1	ubiquitin specific peptidase 1
USP12	ubiquitin specific peptidase 12
USP15	ubiquitin specific peptidase 15
USP16	ubiquitin specific peptidase 16
USP19	ubiquitin specific peptidase 19
USP21	ubiquitin specific peptidase 21
USP24	ubiquitin specific peptidase 24
USP3	ubiquitin specific peptidase 3
USP30	ubiquitin specific peptidase 30
USP32	ubiquitin specific peptidase 32
USP34	ubiquitin specific peptidase 34
USP36	ubiquitin specific peptidase 36

USP37	ubiquitin specific peptidase 37
USP38	ubiquitin specific peptidase 38
USP39	ubiquitin specific peptidase 39
USP4	ubiquitin specific peptidase 4 (proto-oncogene)
USP42	ubiquitin specific peptidase 42
USP45	ubiquitin specific peptidase 45
USP47	ubiquitin specific peptidase 47
USP48	ubiquitin specific peptidase 48
USP49	ubiquitin specific peptidase 49
USP5	ubiquitin specific peptidase 5 (isopeptidase T)
USP53	ubiquitin specific peptidase 53
USP9X	ubiquitin specific peptidase 9, X-linked
USPL1	ubiquitin specific peptidase like 1
UTP11L	UTP11-like, U3 small nucleolar ribonucleoprotein (yeast)
UTP18	UTP18 small subunit (SSU) processome component homolog (yeast)
UTP23	UTP23, small subunit (SSU) processome component, homolog (yeast)
UTP3	UTP3, small subunit (SSU) processome component, homolog (S. cerevisiae)
UTS2B	urotensin 2B
UVRAG	UV radiation resistance associated
UXS1	UDP-glucuronate decarboxylase 1
VAMP1	vesicle-associated membrane protein 1 (synaptobrevin 1)
VAMP4	vesicle-associated membrane protein 4
VAPA	VAMP (vesicle-associated membrane protein)-associated protein A, 33kDa
VASP	vasodilator-stimulated phosphoprotein
VAV1	vav 1 guanine nucleotide exchange factor
VCP	valosin containing protein
VCPIP1	valosin containing protein (p97)/p47 complex interacting protein 1
VCPKMT	valosin containing protein lysine (K) methyltransferase
VDAC1	voltage-dependent anion channel 1
VDAC2	voltage-dependent anion channel 2
VDAC3	voltage-dependent anion channel 3
VEGFA	vascular endothelial growth factor A
VEZF1	vascular endothelial zinc finger 1
VEZT	vezatin, adherens junctions transmembrane protein
VHL	von Hippel-Lindau tumor suppressor, E3 ubiquitin protein ligase
VKORC1	vitamin K epoxide reductase complex, subunit 1
VMAC	vimentin-type intermediate filament associated coiled-coil protein
VMP1	vacuole membrane protein 1
VPREB3	pre-B lymphocyte 3
VPS13B	vacuolar protein sorting 13 homolog B (yeast)
VPS13C	vacuolar protein sorting 13 homolog C (S. cerevisiae)

**VPS18**	**vacuolar protein sorting 18 homolog (S. cerevisiae)**
**VPS25**	**vacuolar protein sorting 25 homolog (S. cerevisiae)**
**VPS26A**	**vacuolar protein sorting 26 homolog A (S. pombe)**
**VPS26B**	**vacuolar protein sorting 26 homolog B (S. pombe)**
**VPS28**	**vacuolar protein sorting 28 homolog (S. cerevisiae)**
**VPS29**	**vacuolar protein sorting 29 homolog (S. cerevisiae)**
**VPS33A**	**vacuolar protein sorting 33 homolog A (S. cerevisiae)**
**VPS36**	**vacuolar protein sorting 36 homolog (S. cerevisiae)**
**VPS37A**	**vacuolar protein sorting 37 homolog A (S. cerevisiae)**
**VPS37C**	**vacuolar protein sorting 37 homolog C (S. cerevisiae)**
**VPS39**	**vacuolar protein sorting 39 homolog (S. cerevisiae)**
**VPS45**	**vacuolar protein sorting 45 homolog (S. cerevisiae)**
**VPS4B**	**vacuolar protein sorting 4 homolog B (S. cerevisiae)**
**VPS53**	**vacuolar protein sorting 53 homolog (S. cerevisiae)**
**VPS72**	**vacuolar protein sorting 72 homolog (S. cerevisiae)**
**VPS9D1**	**VPS9 domain containing 1**
**VRK2**	**vaccinia related kinase 2**
**VSX2**	**visual system homeobox 2**
**VTA1**	**vesicle (multivesicular body) trafficking 1**
**VTI1A**	**vesicle transport through interaction with t-SNAREs 1A**
**VTI1B**	**vesicle transport through interaction with t-SNAREs 1B**
**VTN**	**vitronectin**
**VTRNA1-1**	**vault RNA 1-1**
**VWA8**	**von Willebrand factor A domain containing 8**
**VWA9**	**von Willebrand factor A domain containing 9**
**VWCE**	**von Willebrand factor C and EGF domains**
**WAC**	**WW domain containing adaptor with coiled-coil**
**WARS**	**tryptophanyl-tRNA synthetase**
**WARS2**	**tryptophanyl tRNA synthetase 2, mitochondrial**
**WASF2**	**WAS protein family, member 2**
**WASL**	**Wiskott-Aldrich syndrome-like**
**WBP1**	**WW domain binding protein 1**
**WBP1L**	**WW domain binding protein 1-like**
**WBP2**	**WW domain binding protein 2**
**WBP2NL**	**WBP2 N-terminal like**
**WBP4**	**WW domain binding protein 4**
**WBSCR22**	**Williams Beuren syndrome chromosome region 22**
**WDFY2**	**WD repeat and FYVE domain containing 2**
**WDFY4**	**WDFY family member 4**
**WDPCP**	**WD repeat containing planar cell polarity effector**
**WDR1**	**WD repeat domain 1**

**WDR11**	**WD repeat domain 11**
**WDR18**	**WD repeat domain 18**
**WDR24**	**WD repeat domain 24**
**WDR25**	**WD repeat domain 25**
**WDR27**	**WD repeat domain 27**
**WDR31**	**WD repeat domain 31**
**WDR33**	**WD repeat domain 33**
**WDR34**	**WD repeat domain 34**
**WDR36**	**WD repeat domain 36**
**WDR4**	**WD repeat domain 4**
**WDR41**	**WD repeat domain 41**
**WDR43**	**WD repeat domain 43**
**WDR44**	**WD repeat domain 44**
**WDR47**	**WD repeat domain 47**
**WDR53**	**WD repeat domain 53**
**WDR54**	**WD repeat domain 54**
**WDR55**	**WD repeat domain 55**
**WDR5B**	**WD repeat domain 5B**
**WDR6**	**WD repeat domain 6**
**WDR60**	**WD repeat domain 60**
**WDR62**	**WD repeat domain 62**
**WDR74**	**WD repeat domain 74**
**WDR75**	**WD repeat domain 75**
**WDR78**	**WD repeat domain 78**
**WDR81**	**WD repeat domain 81**
**WDR82**	**WD repeat domain 82**
**WDR83**	**WD repeat domain 83**
**WDR83OS**	**WD repeat domain 83 opposite strand**
**WDR87**	**WD repeat domain 87**
**WDR89**	**WD repeat domain 89**
**WDR90**	**WD repeat domain 90**
**WDR92**	**WD repeat domain 92**
**WDTC1**	**WD and tetratricopeptide repeats 1**
**WFDC3**	**WAP four-disulfide core domain 3**
**WHSC1**	**Wolf-Hirschhorn syndrome candidate 1**
**WIBG**	**within bgcn homolog (Drosophila)**
**WIPI1**	**WD repeat domain, phosphoinositide interacting 1**
**WIZ**	**widely interspaced zinc finger motifs**
**WNK1**	**WNK lysine deficient protein kinase 1**
**WNT1**	**wingless-type MMTV integration site family, member 1**
**WNT10A**	**wingless-type MMTV integration site family, member 10A**

**WNT2B**	**wingless-type MMTV integration site family, member 2B**
**WRAP53**	**WD repeat containing, antisense to TP53**
**WSB1**	**WD repeat and SOCS box containing 1**
**WSB2**	**WD repeat and SOCS box containing 2**
**WTAP**	**Wilms tumor 1 associated protein**
**WWOX**	**WW domain containing oxidoreductase**
**WWP2**	**WW domain containing E3 ubiquitin protein ligase 2**
**XAB2**	**XPA binding protein 2**
**XBP1**	**X-box binding protein 1**
**XIST**	**X inactive specific transcript (non-protein coding)**
**XKR7**	**XK, Kell blood group complex subunit-related family, member 7**
**XPC**	**xeroderma pigmentosum, complementation group C**
**XPNPEP3**	**X-prolyl aminopeptidase 3, mitochondrial**
**XPO1**	**exportin 1**
**XPO4**	**exportin 4**
**XPO5**	**exportin 5**
**XPOT**	**exportin, tRNA**
**XRCC2**	**X-ray repair complementing defective repair in Chinese hamster cells 2**
**XRCC4**	**X-ray repair complementing defective repair in Chinese hamster cells 4**
**XRCC5**	**X-ray repair complementing defective repair in Chinese hamster cells 5 (double-strand-break rejoining)**
**XRCC6**	**X-ray repair complementing defective repair in Chinese hamster cells 6**
**XRCC6BP1**	**XRCC6 binding protein 1**
**XRN1**	**5'-3' exoribonuclease 1**
**XRN2**	**5'-3' exoribonuclease 2**
**XRRA1**	**X-ray radiation resistance associated 1**
**XXYLT1**	**xyloside xylosyltransferase 1**
**XYLT2**	**xylosyltransferase II**
**YAE1D1**	**Yae1 domain containing 1**
**YARS**	**tyrosyl-tRNA synthetase**
**YARS2**	**tyrosyl-tRNA synthetase 2, mitochondrial**
**YBEY**	**ybeY metallopeptidase (putative)**
**YBX1**	**Y box binding protein 1**
**YBX3**	**Y box binding protein 3**
**YDJC**	**YdjC homolog (bacterial)**
**YEATS4**	**YEATS domain containing 4**
**YIF1A**	**Yip1 interacting factor homolog A (S. cerevisiae)**
**YIPF2**	**Yip1 domain family, member 2**
**YIPF3**	**Yip1 domain family, member 3**
**YLPM1**	**YLP motif containing 1**
**YME1L1**	**YME1-like 1 ATPase**
**YOD1**	**YOD1 deubiquitinase**

**YTHDC1**	**YTH domain containing 1**
**YTHDF2**	**YTH N(6)-methyladenosine RNA binding protein 2**
**YTHDF3**	**YTH N(6)-methyladenosine RNA binding protein 3**
**YWHAE**	**tyrosine 3-monooxygenase/tryptophan 5-monooxygenase activation protein, epsilon**
**YWHAG**	**tyrosine 3-monooxygenase/tryptophan 5-monooxygenase activation protein, gamma**
**YWHAQ**	**tyrosine 3-monooxygenase/tryptophan 5-monooxygenase activation protein, theta**
**YWHAZ**	**tyrosine 3-monooxygenase/tryptophan 5-monooxygenase activation protein, zeta**
**YY1AP1**	**YY1 associated protein 1**
**ZACN**	**zinc activated ligand-gated ion channel**
**ZBED5**	**zinc finger, BED-type containing 5**
**ZBP1**	**Z-DNA binding protein 1**
**ZBTB1**	**zinc finger and BTB domain containing 1**
**ZBTB10**	**zinc finger and BTB domain containing 10**
**ZBTB11**	**zinc finger and BTB domain containing 11**
**ZBTB17**	**zinc finger and BTB domain containing 17**
**ZBTB2**	**zinc finger and BTB domain containing 2**
**ZBTB21**	**zinc finger and BTB domain containing 21**
**ZBTB25**	**zinc finger and BTB domain containing 25**
**ZBTB3**	**zinc finger and BTB domain containing 3**
**ZBTB32**	**zinc finger and BTB domain containing 32**
**ZBTB37**	**zinc finger and BTB domain containing 37**
**ZBTB4**	**zinc finger and BTB domain containing 4**
**ZBTB40**	**zinc finger and BTB domain containing 40**
**ZBTB41**	**zinc finger and BTB domain containing 41**
**ZBTB43**	**zinc finger and BTB domain containing 43**
**ZBTB45**	**zinc finger and BTB domain containing 45**
**ZBTB5**	**zinc finger and BTB domain containing 5**
**ZBTB6**	**zinc finger and BTB domain containing 6**
**ZBTB7B**	**zinc finger and BTB domain containing 7B**
**ZBTB8OS**	**zinc finger and BTB domain containing 8 opposite strand**
**ZC2HC1A**	**zinc finger, C2HC-type containing 1A**
**ZC2HC1B**	**zinc finger, C2HC-type containing 1B**
**ZC3H10**	**zinc finger CCCH-type containing 10**
**ZC3H12D**	**zinc finger CCCH-type containing 12D**
**ZC3H13**	**zinc finger CCCH-type containing 13**
**ZC3H14**	**zinc finger CCCH-type containing 14**
**ZC3H15**	**zinc finger CCCH-type containing 15**
**ZC3H3**	**zinc finger CCCH-type containing 3**
**ZC3H4**	**zinc finger CCCH-type containing 4**

ZC3H6	zinc finger CCCH-type containing 6
ZC3H7A	zinc finger CCCH-type containing 7A
ZC3H7B	zinc finger CCCH-type containing 7B
ZC3H8	zinc finger CCCH-type containing 8
ZC3HAV1	zinc finger CCCH-type, antiviral 1
ZC3HC1	zinc finger, C3HC-type containing 1
ZCCHC10	zinc finger, CCHC domain containing 10
ZCCHC2	zinc finger, CCHC domain containing 2
ZCCHC3	zinc finger, CCHC domain containing 3
ZCCHC4	zinc finger, CCHC domain containing 4
ZCCHC6	zinc finger, CCHC domain containing 6
ZCCHC8	zinc finger, CCHC domain containing 8
ZCCHC9	zinc finger, CCHC domain containing 9
ZCWPW1	zinc finger, CW type with PWWP domain 1
ZDHHC12	zinc finger, DHHC-type containing 12
ZDHHC16	zinc finger, DHHC-type containing 16
ZDHHC3	zinc finger, DHHC-type containing 3
ZDHHC4	zinc finger, DHHC-type containing 4
ZDHHC5	zinc finger, DHHC-type containing 5
ZDHHC6	zinc finger, DHHC-type containing 6
ZEB1	zinc finger E-box binding homeobox 1
ZEB1-AS1	ZEB1 antisense RNA 1
ZFAND2A	zinc finger, AN1-type domain 2A
ZFAND2B	zinc finger, AN1-type domain 2B
ZFAND3	zinc finger, AN1-type domain 3
ZFAND5	zinc finger, AN1-type domain 5
ZFAS1	ZNFX1 antisense RNA 1
ZFAT	zinc finger and AT hook domain containing
ZFAT-AS1	ZFAT antisense RNA 1
ZFC3H1	zinc finger, C3H1-type containing
ZFHX2	zinc finger homeobox 2
ZFP3	ZFP3 zinc finger protein
ZFP30	ZFP30 zinc finger protein
ZFP36	ZFP36 ring finger protein
ZFP36L1	ZFP36 ring finger protein-like 1
ZFP36L2	ZFP36 ring finger protein-like 2
ZFP62	ZFP62 zinc finger protein
ZFP64	ZFP64 zinc finger protein
ZFP69	ZFP69 zinc finger protein
ZFP69B	ZFP69 zinc finger protein B
ZFP90	ZFP90 zinc finger protein

**ZFP91**	**ZFP91 zinc finger protein**
**ZFP91-CNTF**	**ZFP91-CNTF readthrough (NMD candidate)**
**ZFPL1**	**zinc finger protein-like 1**
**ZFR**	**zinc finger RNA binding protein**
**ZFYVE26**	**zinc finger, FYVE domain containing 26**
**ZFYVE27**	**zinc finger, FYVE domain containing 27**
**ZGRF1**	**zinc finger, GRF-type containing 1**
**ZHX2**	**zinc fingers and homeoboxes 2**
**ZKSCAN2**	**zinc finger with KRAB and SCAN domains 2**
**ZKSCAN3**	**zinc finger with KRAB and SCAN domains 3**
**ZKSCAN5**	**zinc finger with KRAB and SCAN domains 5**
**ZMAT2**	**zinc finger, matrin-type 2**
**ZMAT3**	**zinc finger, matrin-type 3**
**ZMPSTE24**	**zinc metallopeptidase STE24**
**ZMYM1**	**zinc finger, MYM-type 1**
**ZMYM2**	**zinc finger, MYM-type 2**
**ZMYM5**	**zinc finger, MYM-type 5**
**ZMYM6**	**zinc finger, MYM-type 6**
**ZMYND12**	**zinc finger, MYND-type containing 12**
**ZMYND8**	**zinc finger, MYND-type containing 8**
**ZNF10**	**zinc finger protein 10**
**ZNF101**	**zinc finger protein 101**
**ZNF114**	**zinc finger protein 114**
**ZNF121**	**zinc finger protein 121**
**ZNF131**	**zinc finger protein 131**
**ZNF132**	**zinc finger protein 132**
**ZNF138**	**zinc finger protein 138**
**ZNF140**	**zinc finger protein 140**
**ZNF142**	**zinc finger protein 142**
**ZNF143**	**zinc finger protein 143**
**ZNF146**	**zinc finger protein 146**
**ZNF148**	**zinc finger protein 148**
**ZNF182**	**zinc finger protein 182**
**ZNF184**	**zinc finger protein 184**
**ZNF195**	**zinc finger protein 195**
**ZNF2**	**zinc finger protein 2**
**ZNF200**	**zinc finger protein 200**
**ZNF202**	**zinc finger protein 202**
**ZNF207**	**zinc finger protein 207**
**ZNF212**	**zinc finger protein 212**
**ZNF219**	**zinc finger protein 219**

**ZNF23**	**zinc finger protein 23**
**ZNF234**	**zinc finger protein 234**
**ZNF236**	**zinc finger protein 236**
**ZNF24**	**zinc finger protein 24**
**ZNF250**	**zinc finger protein 250**
**ZNF251**	**zinc finger protein 251**
**ZNF254**	**zinc finger protein 254**
**ZNF26**	**zinc finger protein 26**
**ZNF263**	**zinc finger protein 263**
**ZNF267**	**zinc finger protein 267**
**ZNF268**	**zinc finger protein 268**
**ZNF271P**	**zinc finger protein 271, pseudogene**
**ZNF274**	**zinc finger protein 274**
**ZNF276**	**zinc finger protein 276**
**ZNF282**	**zinc finger protein 282**
**ZNF292**	**zinc finger protein 292**
**ZNF296**	**zinc finger protein 296**
**ZNF3**	**zinc finger protein 3**
**ZNF302**	**zinc finger protein 302**
**ZNF304**	**zinc finger protein 304**
**ZNF321P**	**zinc finger protein 321, pseudogene**
**ZNF335**	**zinc finger protein 335**
**ZNF33A**	**zinc finger protein 33A**
**ZNF33B**	**zinc finger protein 33B**
**ZNF34**	**zinc finger protein 34**
**ZNF35**	**zinc finger protein 35**
**ZNF366**	**zinc finger protein 366**
**ZNF367**	**zinc finger protein 367**
**ZNF37A**	**zinc finger protein 37A**
**ZNF384**	**zinc finger protein 384**
**ZNF394**	**zinc finger protein 394**
**ZNF397**	**zinc finger protein 397**
**ZNF398**	**zinc finger protein 398**
**ZNF408**	**zinc finger protein 408**
**ZNF410**	**zinc finger protein 410**
**ZNF425**	**zinc finger protein 425**
**ZNF426**	**zinc finger protein 426**
**ZNF428**	**zinc finger protein 428**
**ZNF432**	**zinc finger protein 432**
**ZNF433**	**zinc finger protein 433**
**ZNF436**	**zinc finger protein 436**

**ZNF436-AS1**	**ZNF436 antisense RNA 1**
**ZNF438**	**zinc finger protein 438**
**ZNF446**	**zinc finger protein 446**
**ZNF451**	**zinc finger protein 451**
**ZNF460**	**zinc finger protein 460**
**ZNF48**	**zinc finger protein 48**
**ZNF490**	**zinc finger protein 490**
**ZNF496**	**zinc finger protein 496**
**ZNF497**	**zinc finger protein 497**
**ZNF511**	**zinc finger protein 511**
**ZNF512B**	**zinc finger protein 512B**
**ZNF513**	**zinc finger protein 513**
**ZNF518A**	**zinc finger protein 518A**
**ZNF524**	**zinc finger protein 524**
**ZNF527**	**zinc finger protein 527**
**ZNF543**	**zinc finger protein 543**
**ZNF546**	**zinc finger protein 546**
**ZNF547**	**zinc finger protein 547**
**ZNF548**	**zinc finger protein 548**
**ZNF552**	**zinc finger protein 552**
**ZNF554**	**zinc finger protein 554**
**ZNF555**	**zinc finger protein 555**
**ZNF561**	**zinc finger protein 561**
**ZNF565**	**zinc finger protein 565**
**ZNF573**	**zinc finger protein 573**
**ZNF574**	**zinc finger protein 574**
**ZNF575**	**zinc finger protein 575**
**ZNF576**	**zinc finger protein 576**
**ZNF579**	**zinc finger protein 579**
**ZNF580**	**zinc finger protein 580**
**ZNF581**	**zinc finger protein 581**
**ZNF583**	**zinc finger protein 583**
**ZNF584**	**zinc finger protein 584**
**ZNF587**	**zinc finger protein 587**
**ZNF589**	**zinc finger protein 589**
**ZNF592**	**zinc finger protein 592**
**ZNF593**	**zinc finger protein 593**
**ZNF594**	**zinc finger protein 594**
**ZNF598**	**zinc finger protein 598**
**ZNF605**	**zinc finger protein 605**
**ZNF614**	**zinc finger protein 614**

ZNF616	zinc finger protein 616
ZNF621	zinc finger protein 621
ZNF622	zinc finger protein 622
ZNF623	zinc finger protein 623
ZNF627	zinc finger protein 627
ZNF628	zinc finger protein 628
ZNF639	zinc finger protein 639
ZNF644	zinc finger protein 644
ZNF646	zinc finger protein 646
ZNF653	zinc finger protein 653
ZNF655	zinc finger protein 655
ZNF668	zinc finger protein 668
ZNF671	zinc finger protein 671
ZNF672	zinc finger protein 672
ZNF678	zinc finger protein 678
ZNF687	zinc finger protein 687
ZNF688	zinc finger protein 688
ZNF689	zinc finger protein 689
ZNF692	zinc finger protein 692
ZNF696	zinc finger protein 696
ZNF70	zinc finger protein 70
ZNF706	zinc finger protein 706
ZNF720	zinc finger protein 720
ZNF721	zinc finger protein 721
ZNF724P	zinc finger protein 724, pseudogene
ZNF740	zinc finger protein 740
ZNF747	zinc finger protein 747
ZNF749	zinc finger protein 749
ZNF76	zinc finger protein 76
ZNF763	zinc finger protein 763
ZNF764	zinc finger protein 764
ZNF767P	zinc finger family member 767, pseudogene
ZNF768	zinc finger protein 768
ZNF77	zinc finger protein 77
ZNF770	zinc finger protein 770
ZNF775	zinc finger protein 775
ZNF783	zinc finger family member 783
ZNF784	zinc finger protein 784
ZNF785	zinc finger protein 785
ZNF786	zinc finger protein 786
ZNF789	zinc finger protein 789

**ZNF79**	**zinc finger protein 79**
**ZNF791**	**zinc finger protein 791**
**ZNF8**	**zinc finger protein 8**
**ZNF800**	**zinc finger protein 800**
**ZNF805**	**zinc finger protein 805**
**ZNF827**	**zinc finger protein 827**
**ZNF830**	**zinc finger protein 830**
**ZNF837**	**zinc finger protein 837**
**ZNF839**	**zinc finger protein 839**
**ZNF843**	**zinc finger protein 843**
**ZNF846**	**zinc finger protein 846**
**ZNF92**	**zinc finger protein 92**
**ZNFX1**	**zinc finger, NFX1-type containing 1**
**ZNHIT2**	**zinc finger, HIT-type containing 2**
**ZNHIT3**	**zinc finger, HIT-type containing 3**
**ZNHIT6**	**zinc finger, HIT-type containing 6**
**ZNRD1**	**zinc ribbon domain containing 1**
**ZNRF1**	**zinc and ring finger 1, E3 ubiquitin protein ligase**
**ZNRF2**	**zinc and ring finger 2, E3 ubiquitin protein ligase**
**ZPBP2**	**zona pellucida binding protein 2**
**ZRANB2**	**zinc finger, RAN-binding domain containing 2**
**ZRANB3**	**zinc finger, RAN-binding domain containing 3**
**ZSCAN12**	**zinc finger and SCAN domain containing 12**
**ZSCAN16**	**zinc finger and SCAN domain containing 16**
**ZSCAN21**	**zinc finger and SCAN domain containing 21**
**ZSCAN22**	**zinc finger and SCAN domain containing 22**
**ZSCAN25**	**zinc finger and SCAN domain containing 25**
**ZSCAN26**	**zinc finger and SCAN domain containing 26**
**ZSCAN30**	**zinc finger and SCAN domain containing 30**
**ZSCAN31**	**zinc finger and SCAN domain containing 31**
**ZSWIM1**	**zinc finger, SWIM-type containing 1**
**ZSWIM3**	**zinc finger, SWIM-type containing 3**
**ZSWIM4**	**zinc finger, SWIM-type containing 4**
**ZSWIM7**	**zinc finger, SWIM-type containing 7**
**ZSWIM8**	**zinc finger, SWIM-type containing 8**
**ZW10**	**zw10 kinetochore protein**
**ZWILCH**	**zwilch kinetochore protein**
**ZYX**	**zyxin**
**ZZEF1**	**zinc finger, ZZ-type with EF-hand domain 1**
**ZZZ3**	**zinc finger, ZZ-type containing 3**

Supplementary Table 6. List of 371 NFATC1 targets indicated in the KEGG metabolic pathway.

Gene_Name
AASDH
ACAA1
ACACA
ACADM
ACADS
ACADVL
ACAT1
ACAT2
ACO2
ACOT2
ACOX1
ACOX3
ACSL1
ACY3
ACYP2
ADH5
ADI1
ADSL
ADSS
AFMID
AGL
AGPAT3
AGPS
AHCYL2
AK2
AKR1A1
ALAS1
ALDH18A1
ALDH3A1
ALDH4A1
ALDH6A1

ALDH9A1
ALDOA
AMD1
AMDHD2
AMPD2
AMT
AOC2
APOE
APRT
ASAH1
ASH1L
ATIC
ATP5B
ATP5E
ATP5G1
ATP5G2
ATP5G3
ATP5I
ATP5J
ATP5L
ATP6V0A2
ATP6V0B
ATP6V0D1
ATP6V1A
ATP6V1C1
ATP6V1D
ATP6V1G1
ATP6V1G2
ATP6V1H
B4GALT1
B4GALT6
BCAT1
BCAT2
BCKDHA
CAD
CDIPT
CDS2
CMPK1
COX11
COX15
COX17

COX5A
COX5B
COX6C
COX7A2
COX7A2L
COX7B
COX7C
COX8A
CPT1C
CRLS1
CS
CTH
CYC1
CYP1B1
CYP51A1
DBT
DCK
DCTD
DCXR
DEGS1
DGAT1
DGKA
DGKH
DGUOK
DHODH
DLAT
DLD
DLST
DNMT1
DNMT3A
DOT1L
DUT
EBP
ECI1
EHMT1
ELOVL5
ENO1
ENO2
ENOPH1
FADS1
FADS2

FPGT
FUK
GALE
GALK2
GALM
GANC
GAPDH
GART
GBA
GBE1
GCDH
GGT5
GLB1
GLS
GLS2
GLUD1
GLYCTK
GMPPB
GMPR2
GNPAT
GOT2
GPAM
GPD2
GPX1
GPX3
GPX4
GUSB
GYS1
H6PD
HADH
HADHA
HADHB
HAGH
HAGHL
HEMK1
HEXA
HIBADH
HMGCL
HMGCR
HPD
HSD17B12

HYI
IARS
IDH3A
IDH3B
IL4I1
IMPA1
IMPDH2
INPP5E
INPP5K
IPMK
IPPK
ISYNA1
ITPA
ITPKB
JMJD7-PLA2G4B
KDSR
KHK
KMO
LAP3
LARS
LCAT
LCLAT1
LCMT2
LDHA
LDHB
LDLR
LPCAT3
LPCAT4
LRPAP1
LTA4H
LTC4S
LYPLA2
MAT2A
MBOAT7
MCCC1
MCCC2
MCEE
MDH1
MDH2
METTL2B
METTL6

MGLL
MIF
MINPP1
MPI
MSMO1
MTHFD1
MTHFD2
MTMR2
MTMR6
MTR
MUT
NAGK
NANP
NANS
NAT6
NDUFA10
NDUFA11
NDUFA2
NDUFA4
NDUFA4L2
NDUFA5
NDUFA6
NDUFA7
NDUFA9
NDUFAB1
NDUFB1
NDUFB10
NDUFB2
NDUFB3
NDUFB5
NDUFB6
NDUFB7
NDUFB9
NDUFC1
NDUFC2
NDUFS1
NDUFS3
NDUFS5
NDUFS6
NDUFS7
NDUFS8

NDUFV3
NEU1
NEU3
NME1
NME1-NME2
NME3
NME6
NPL
NSD1
NT5C
NT5C2
NT5E
NUDT5
NUDT9
ODC1
OGDH
OXCT1
P4HA1
PAFAH1B2
PAFAH1B3
PAFAH2
PAICS
PCCB
PCK2
PCYT1A
PCYT2
PDE1B
PDE4A
PDE7A
PDE8A
PDE9A
PEMT
PFAS
PFKL
PFKM
PGAM1
PGAM2
PGD
PGLS
PGM1
PGM2

PGP
PHOSPHO1
PHPT1
PI4KA
PIK3CA
PIK3CD
PIK3CG
PIKFYVE
PIP4K2B
PIP4K2C
PIP5K1A
PIP5K1B
PISD
PLA2G6
PLOD1
PMM1
PNP
PNPT1
POLA2
POLD1
POLD2
POLD3
POLD4
POLE
POLE2
POLE3
POLE4
POLR1A
POLR1B
POLR1C
POLR2A
POLR2B
POLR2C
POLR2F
POLR2G
POLR2H
POLR2I
POLR2K
POLR2L
POLR3A
POLR3B

POLR3C
POLR3D
POLR3G
POLR3H
POLR3K
PPA2
PPAT
PRIM1
PRIM2
PSPH
PTDSS1
PTDSS2
PTEN
PTGES2
PYCR2
PYCRL
PYGM
RBKS
RPIA
RRM1
RRM2
RRM2B
SAT1
SCARB1
SDHD
SETD1A
SETD1B
SETD2
SETDB1
SETDB2
SETMAR
SGPP2
SHMT2
SMPD1
SMPD4
SPHK2
SPTLC1
SRD5A1
SUCLG2
TCIRG1
TECR

TK1
TM7SF2
TPI1
TRMT11
TSTA3
TYMP
TYMS
UCKL1
UGCG
UGDH
UGP2
UMPS
UQCR11
UQCRC1
UQCRC2
UQCRH
UQCRQ
UXS1
VAPA
VDAC1
VDAC2
VDAC3
WARS
WARS2
WBSCR22
WHSC1
YOD1
ZNRD1

Supplementary Table 7. GSEA KEGG pathway enrichment analysis of the gene list in (B).

Gene Set Name [# Genes (K)]	Description	# Genes in Overlap (k)	p-value	FDR q-value
KEGG_OXIDATIVE_PHOSPHORYLATION [131]	Oxidative phosphorylation	64	1.29 e-92	2.4 e-90
KEGG_PURINE_METABOLISM [159]	Purine metabolism	65	1.57 e-87	1.46 e-85
KEGG_PYRIMIDINE_METABOLISM [98]	Pyrimidine metabolism	55	3.95 e-84	2.45 e-82
KEGG_HUNTINGTONS_DISEASE [180]	Huntington's disease	60	2.99 e-74	1.39 e-72
KEGG_PARKINSONS_DISEASE [128]	Parkinson's disease	50	4.95 e-66	1.84 e-64
KEGG_ALZHEIMERS_DISEASE [165]	Alzheimer's disease	49	9.34 e-58	2.9 e-56
KEGG_VALINE_LEUCINE_AND_ISOLEUCINE_DEG	Valine, leucine and isoleucine degradation	24	1.07 e-36	2.83 e-35
KEGG_RNA_POLYMERASE [29]	RNA polymerase	20	7.87 e-34	1.83 e-32
KEGG_LYSINE_DEGRADATION [44]	Lysine degradation	21	1.34 e-30	2.77 e-29
KEGG_GLYCEROPHOSPHOLIPID_METABOLISM [77]	Glycerophospholipid metabolism	24	2.45 e-29	4.55 e-28
KEGG_INOSITOL_PHOSPHATE_METABOLISM [54]	Inositol phosphate metabolism	21	3.16 e-28	5.34 e-27
KEGG_AMINO_SUGAR_AND_NUCLEOTIDE_SUGAR_	Amino sugar and nucleotide sugar metabolism	19	9.87 e-27	1.49 e-25
KEGG_GLYCOLYSIS_GLUCONEOGENESIS [62]	Glycolysis / Gluconeogenesis	21	1.04 e-26	1.49 e-25
KEGG_FATTY_ACID_METABOLISM [42]	Fatty acid metabolism	17	1.89 e-23	2.51 e-22
KEGG_PHOSPHATIDYLINOSITOL_SIGNALING_SY	Phosphatidylinositol signaling system	19	1.99 e-21	2.47 e-20

KEGG_FRUCTOSE_AND_MANNOSE_METAB OLISM [34]	Fructose and mannose metabolism	14	1.16 e-19	1.35 e-18
KEGG_SPHINGOLIPID_METABOLISM [39]	Sphingolipid metabolism	14	1.2 e-18	1.31 e-17
KEGG_CITRATE_CYCLE_TCA_CYCLE [31]	Citrate cycle (TCA cycle)	13	1.78 e-18	1.79 e-17
KEGG_PYRUVATE_METABOLISM [40]	Pyruvate metabolism	14	1.83 e-18	1.79 e-17
KEGG_TYROSINE_METABOLISM [42]	Tyrosine metabolism	14	4.09 e-18	3.8 e-17
KEGG_PROPANOATE_METABOLISM [33]	Propanoate metabolism	13	4.85 e-18	4.3 e-17
KEGG_CYSTEINE_AND_METHIONINE_METAB OLIS	Cysteine and methionine metabolism	13	7.78 e-18	6.58 e-17
KEGG_BIOSYNTHESIS_OF_UNSATURATED_F ATTY	Biosynthesis of unsaturated fatty acids	11	6.66 e-17	5.39 e-16
KEGG_GLYOXYLATE_AND_DICARBOXYLATE_ META	Glyoxylate and dicarboxylate metabolism	10	7.99 e-17	6.19 e-16
KEGG_TRYPTOPHAN_METABOLISM [40]	Tryptophan metabolism	13	9.55 e-17	7.11 e-16
KEGG_ARGININE_AND_PROLINE_METABOLI SM [54]	Arginine and proline metabolism	14	2.25 e-16	1.61 e-15
KEGG_ALANINE_ASPARTATE_AND_GLUTAM ATE_M	Alanine, aspartate and glutamate metabolism	11	1.11 e-14	7.66 e-14
KEGG_GALACTOSE_METABOLISM [26]	Galactose metabolism	10	4.84 e-14	3.1 e-13
KEGG_SELENOAMINO_ACID_METABOLISM [26]	Selenoamino acid metabolism	10	4.84 e-14	3.1 e-13
KEGG_DNA_REPLICATION [36]	DNA replication	11	5 e-14	3.1 e-13
KEGG_CARDIAC_MUSCLE_CONTRACTION [7 8]	Cardiac muscle contraction	14	5.68 e-14	3.41 e-13
KEGG_PENTOSE_PHOSPHATE_PATHWAY [27]	Pentose phosphate pathway	10	7.62 e-14	4.43 e-13
KEGG_ONE_CARBON_POOL_BY_FOLATE [17]	One carbon pool by folate	8	2.39 e-12	1.35 e-11
KEGG_STARCH_AND_SUCROSE_METABOLIS M [52]	Starch and sucrose metabolism	11	4.34 e-12	2.38 e-11
KEGG_HISTIDINE_METABOLISM [29]	Histidine metabolism	9	8.87 e-12	4.72 e-11
KEGG_GLYCEROLIPID_METABOLISM [49]	Glycerolipid metabolism	10	6.08 e-11	3.14 e-10
KEGG_GLUTATHIONE_METABOLISM [50]	Glutathione metabolism	10	7.54 e-11	3.79 e-10
KEGG_VIBRIO_CHOLERAE_INFECTION [54]	Vibrio cholerae infection	10	1.69 e-10	8.29 e-10
KEGG_PHENYLALANINE_METABOLISM [18]	Phenylalanine metabolism	7	3.08	1.47

			e-10	e-9
KEGG_GLYCINE_SERINE_AND_THREONINE_META	Glycine, serine and threonine metabolism	8	6.84 e-10	3.18 e-9
KEGG_BUTANOATE_METABOLISM [34]	Butanoate metabolism	8	1.53 e-9	6.96 e-9
KEGG_EPITHELIAL_CELL_SIGNALING_IN_HELI	Epithelial cell signaling in Helicobacter pylori infection	10	1.81 e-9	8.03 e-9
KEGG_BASE_EXCISION_REPAIR [35]	Base excision repair	8	1.97 e-9	8.52 e-9
KEGG_LYSOSOME [121]	Lysosome	12	4.47 e-9	1.89 e-8
KEGG_ARACHIDONIC_ACID_METABOLISM [58]	Arachidonic acid metabolism	9	7.28 e-9	3.01 e-8
KEGG_NUCLEOTIDE_EXCISION_REPAIR [44]	Nucleotide excision repair	8	1.37 e-8	5.54 e-8
KEGG_ETHER_LIPID_METABOLISM [33]	Ether lipid metabolism	7	3.62 e-8	1.43 e-7
KEGG_CYTOSOLIC_DNA_SENSING_PATHWAY [55]	Cytosolic DNA-sensing pathway	8	8.54 e-8	3.31 e-7
KEGG_OTHER_GLYCAN_DEGRADATION [16]	Other glycan degradation	5	4.22 e-7	1.6 e-6
KEGG_PPAR_SIGNALING_PATHWAY [69]	PPAR signaling pathway	8	5.18 e-7	1.93 e-6
KEGG_FC_GAMMA_R_MEDIATED_PHAGOCYTOSIS [96]	Fc gamma R-mediated phagocytosis	9	6.31 e-7	2.3 e-6
KEGG_DRUG_METABOLISM_OTHER_ENZYMES [51]	Drug metabolism - other enzymes	7	8.39 e-7	3 e-6
KEGG_ALPHA_LINOLENIC_ACID_METABOLISM [19]	alpha-Linolenic acid metabolism	5	1.1 e-6	3.85 e-6
KEGG_LIMONENE_AND_PINENE_DEGRADATION [10]	Limonene and pinene degradation	4	2.11 e-6	7.26 e-6
KEGG_NITROGEN_METABOLISM [23]	Nitrogen metabolism	5	3.07 e-6	1.02 e-5
KEGG_PROXIMAL_TUBULE_BICARBONATE_RECLA	Proximal tubule bicarbonate reclamation	5	3.07 e-6	1.02 e-5
KEGG_VALINE_LEUCINE_AND_ISOLEUCINE_BIO	Valine, leucine and isoleucine biosynthesis	4	3.28 e-6	1.07 e-5
KEGG_PEROXISOME [78]	Peroxisome	7	1.51 e-5	4.85 e-5
KEGG_STEROID_BIOSYNTHESIS [17]	Steroid biosynthesis	4	2.26 e-5	7.12 e-5
KEGG_BETA_ALANINE_METABOLISM [22]	beta-Alanine metabolism	4	6.66 e-5	2.07 e-4
KEGG_MISMATCH_REPAIR [23]	Mismatch repair	4	8 e-5	2.44 e-4
KEGG_INSULIN_SIGNALING_PATHWAY [137]	Insulin signaling pathway	8	8.51 e-5	2.55 e-4

KEGG_NICOTINATE_AND_NICOTINAMIDE_METAB	Nicotinate and nicotinamide metabolism	4	9.53 e-5	2.81 e-4
KEGG_VEGF_SIGNALING_PATHWAY [76]	VEGF signaling pathway	6	1.28 e-4	3.71 e-4
KEGG_HOMOLOGOUS_RECOMBINATION [28]	Homologous recombination	4	1.78 e-4	5.01 e-4
KEGG_PENTOSE_AND_GLUCURONATE_INTERCONV	Pentose and glucuronate interconversions	4	1.78 e-4	5.01 e-4

Supplementary Table 8. Astrocyte culture-medium composition (Astro-c)

General composition of complete Astrocyte growth media

50% Neurobasal
50% DMEM
1mM Sodium Pyruvate
2mM L-glutamine
1 x SATO
5ug/ml NAC
5ng/ml HBEGF

A. Neurobasal medium

**Components**	**Molecular Weight**	**Concentration (mg/L)**	**mM**
**Amino Acids**			
Glycine	75	30	0.4
L-Alanine	89	2	0.02247191
L-Arginine hydrochloride	211	84	0.39810428
L-Asparagine-H2O	150	0.83	0.005533333
L-Cysteine	121	31.5	0.2603306
L-Histidine hydrochloride-H2O	210	42	0.2
L-Isoleucine	131	105a	0.8015267a
L-Leucine	131	105a	0.8015267a
L-Lysine hydrochloride	183	146	0.7978142
L-Methionine	149	30	0.20134228
L-Phenylalanine	165	66	0.4
L-Proline	115	7.76	0.06747826
L-Serine	105	42	0.4
L-Threonine	119	95	0.79831934
L-Tryptophan	204	16	0.078431375
L-Tyrosine	181	72	0.39779004

L-Valine	117	94a	0.8034188a
**Vitamins**			
Choline chloride	140	4	0.028571429
D-Calcium pantothenate	477	4	0.008385744
Folic Acid	441	4	0.009070295
Niacinamide	122	4	0.032786883
Pyridoxal hydrochloride	204	4	0.019607844
Riboflavin	376	0.4	0.00106383
Thiamine hydrochloride	337	4	0.011869436
Vitamin B12	1355	0.0068	5.02E-06
i-Inositol	180	7.2	0.04
**Inorganic Salts**			
Calcium Chloride (CaCl2) (anhyd.)	111	200	1.8018018
Ferric Nitrate (Fe(NO3)3"9H2O)	404	0.1	2.48E-04
Magnesium Chloride (anhydrous)	95	77.3	0.8136842
Potassium Chloride (KCl)	75	400	5.3333335
Sodium Bicarbonate (NaHCO3)	84	2200	26.190475
Sodium Chloride (NaCl)	58	3000	51.724136
Sodium Phosphate monobasic (NaH2PO4-H2O)	138	125	0.9057971
Zinc sulfate (ZnSO4-7H2O)	288	0.194	6.74E-04
**Other Components**			
D-Glucose (Dextrose)	180	4500a	25a
HEPES	238	2600	10.92437
Sodium Pyruvate	110	25a	0.22727273a

B. DMEM

Components	Molecular Weight	Concentration (mg/L)	mM
**Amino Acids**			
Glycine	75	30	0.4
L-Arginine hydrochloride	211	84	0.39810428
L-Cystine 2HCl	313	63	0.20127796
L-Glutamine	146	584a	4a
L-Histidine hydrochloride-H2O	210	42	0.2
L-Isoleucine	131	105a	0.8015267a
L-Leucine	131	105a	0.8015267a
L-Lysine hydrochloride	183	146	0.7978142
L-Methionine	149	30	0.20134228

L-Phenylalanine	165	66	0.4
L-Serine	105	42	0.4
L-Threonine	119	95	0.79831934
L-Tryptophan	204	16	0.078431375
L-Tyrosine disodium salt dihydrate	261	104	0.39846742
L-Valine	117	94a	0.8034188a
**Vitamins**			
Choline chloride	140	4	0.028571429
D-Calcium pantothenate	477	4	0.008385744
Folic Acid	441	4	0.009070295
Niacinamide	122	4	0.032786883
Pyridoxine hydrochloride	206	4	0.019417476
Riboflavin	376	0.4	0.00106383
Thiamine hydrochloride	337	4	0.011869436
i-Inositol	180	7.2	0.04
**Inorganic Salts**			
Calcium Chloride (CaCl2) (anhyd.)	111	200	1.8018018
Ferric Nitrate (Fe(NO3)3"9H2O)	404	0.1	2.48E-04
Magnesium Sulfate (MgSO4) (anhyd.)	120	97.67	0.8139166
Potassium Chloride (KCl)	75	400	5.3333335
Sodium Bicarbonate (NaHCO3)	84	3700	44.04762
Sodium Chloride (NaCl)	58	6400	110.344826
Sodium Phosphate monobasic (NaH2PO4-H2O)	138	125	0.9057971
**Other Components**			
D-Glucose (Dextrose)	180	4500a	25a

C. SATO (100X)

In 80 ml Neurobasal medium
800 mg transferrin (Sigma T-1147)
800 mg BSA
128 mg putrescine (Sigma P5780)
20 ul progesterone (Sigma P8783) (from stock: 2.5 mg in 100 ul EtOH) 60 ng/mL (0.2uM)
800 ul sodium selenite (Sigma S5261) (4.0 mg + 10 ul 1N NaOH in 10 mL NB) 40 ng/mL
* Do not reuse progesterone and Na selenite stocks; make fresh each time.
Mix well and filter through pre-rinsed 0.22 um filter.
Make 200 ul or 800 ul aliquots; store at -20oC

Supplementary Table 9. Neuron culture-medium composition (Neuro-c)

Components	Molecular Weight	Concentration (mg/L)	mM
**Amino Acids**			
Glycine	75	30	0.4
L-Alanine	89	2	0.02247191
L-Arginine hydrochloride	211	84	0.39810428
L-Asparagine-H2O	150	0.83	0.005533333
L-Cysteine	121	31.5	0.2603306
L-Histidine hydrochloride-H2O	210	42	0.2
L-Isoleucine	131	105	0.8015267
L-Leucine	131	105	0.8015267a
L-Lysine hydrochloride	183	146a	0.7978142a
L-Methionine	149	30	0.20134228
L-Phenylalanine	165	66	0.4
L-Proline	115	7.76	0.06747826
L-Serine	105	42	0.4
L-Threonine	119	95	0.79831934
L-Tryptophan	204	16	0.078431375
L-Tyrosine	181	72	0.39779004
L-Valine	117	94a	0.8034188a
**Vitamins**			
Choline chloride	140	4	0.028571429
D-Calcium pantothenate	477	4	0.008385744
Folic Acid	441	4	0.009070295
Niacinamide	122	4	0.032786883
Pyridoxal hydrochloride	204	4	0.019607844
Riboflavin	376	0.4	0.00106383
Thiamine hydrochloride	337	4	0.011869436
Vitamin B12	1355	0.0068	5.02E-06
i-Inositol	180	7.2	0.04

**Inorganic Salts**			
Calcium Chloride (CaCl2) (anhyd.)	111	200	1.8018018
Ferric Nitrate (Fe(NO3)3"9H2O)	404	0.1	2.48E-04
Magnesium Chloride (anhydrous)	95	77.3	0.8136842
Potassium Chloride (KCl)	75	400	5.3333335
Sodium Bicarbonate (NaHCO3)	84	2200	26.190475
Sodium Chloride (NaCl)	58	3000	51.724136
Sodium Phosphate monobasic (NaH2PO4-H2O)	138	125	0.9057971
Zinc sulfate (ZnSO4-7H2O)	288	0.194	6.74E-04
**Other Components**			
D-Glucose (Dextrose)	180	4500a	25a
HEPES	238	2600	10.92437
Sodium Pyruvate	110	25	0.22727273a
B27 supplements			
GlutaMAX			

Supplementary Table 10. Complete list of special reagents

**Antibodies**			
PI3 Kinase p85 (phospho Y607)	Abcam	ab182651	WB
PKCα (phospho T638)	Abcam	ab32502	WB
MAP2	Abcam	ab5392	IHC/ICC
VGLUT1	Abcam	ab77822	IHC/ICC
VGAT	Abcam	ab42939	IHC/ICC
Synapsin 1	Abcam	ab8	IHC/ICC
TUJ1	Abcam	ab7751	IHC/ICC
IBA1	Abcam	ab5076	IHC/ICC
NG2	Abcam	ab83178	IHC/ICC
NCAN	Abcam	ab31979	WB
NFAT1 (phospho S326)	Affinit	AF8011	WB
Akt	Cell Signaling Technology	#9272	WB
Akt (Phospho T308)	Cell Signaling Technology	#4056	WB
Akt (Phospho S473)	Cell Signaling Technology	#9271	WB
PI3 Kinase p85	Cell Signaling Technology	#4292	WB
β-Actin	Cell Signaling Technology	#4970	WB
Raptor	Cell Signaling Technology	#48648	WB
Rictor	Cell Signaling Technology	#2114	WB
mTOR	Cell Signaling Technology	#2983	WB
S6K1 (Phospho T389)	Cell Signaling Technology	#97596	WB
S6K1	Cell Signaling Technology	#2708	WB
4E-BP1 (phospho T37/46)	Cell Signaling Technology	#2855	WB
4E-BP1	Cell Signaling Technology	#9644	WB
β-Catenin	Cell Signaling Technology	#9582	WB
GSK-3β	Cell Signaling Technology	#9315	WB
Cleaved Caspase-3	Cell Signaling Technology	#9661	WB
NFAT1	Cell Signaling Technology	#4389	WB
GFAP	Cell Signaling Technology	#12389	WB
PKCα	Cell Signaling Technology	#2056	WB
O4	Merck Millipore	MAB345	IHC/ICC
Wnt2	Proteintech	11160-1-AP	WB
FLAG	Sigma	F3165	WB

APOE	ThermoFisher	PA5-27088	WB
Wnt10a	ThermoFisher	PA5-20542	WB
Wnt3	ThermoFisher	39-0300	WB
Kremen-1	ThermoFisher	PA5-34520	WB
Wnt7a	ThermoFisher	PA5-28289	WB
Wnt6	ThermoFisher	PA5-34326	WB
Wnt5A	ThermoFisher	MA5-15511	WB
LRP6	ThermoFisher	PA5-13145	WB
**Special Reagents**			
LightCycler® 480 SYBR Green I Master	Roche	4707516001	
Sodium oxamate	Cayman	565-73-1	
BCATci-2	Cayman	406191-34-2	
UK5099	R&D	4186	
Etomoxir sodium salt	R&D	4539	
Rapamycin	R&D	1292	
Recombinant Human Wnt-3a protein	R&D	5036-WN	
Recombinant Mouse Wnt-3a protein	R&D	1324-WN	
Recombinant Human/Mouse Wnt-5a Protein	R&D	645-WN	
Recombinant Human Wnt6 protein	Abcam	ab159813	
Recombinant human Wnt7a protein	Abcam	ab129138	
Recombinant Mouse Protein Wnt-6 (Wnt6)	Cusabio	CSB-MP026140MO	
Recombinant Mouse Protein Wnt-7a (Wnt7a)	Cusabio	CSB-MP026141MO	
BAPTA	Sigma	HY-100545	
L-Glutamine-13C5	Sigma	605166	
L-Isoleucine-13C6,15N	Sigma	608092	
BPTES	Selleck	S7753	
LY294002	Selleck	S1105	
MK2206	Selleck	S1078	
KU0063794	Selleck	S1226	
Oligomycin	Tocris	4110	
Go6976	Tocris	2253	
Recombinant APOE3	Novus Biologicals	4144-AE-500	
Recombinant APOE4	Novus Biologicals	NBP1-99634	

Supplementary Table 11. Complete list of primers and plasmids.

Plasmid/constructs

M50 Super 8X TOPflash	Addgene	#12456
M50 Super 8X FOPflash	Addgene	#12457
LRP6 Human shRNA Plasmid Kit	Origene	TG311676
Lrp6 Mouse shRNA Plasmid Kit	Origene	TR509944
Lrp6 (NM_008514) Mouse Tagged ORF Clone Lentiviral Particle	Origene	MR212053L4V
Ctnnb1 Mouse shRNA Lentiviral Particle (Locus ID 12387)	Origene	TL500280V
Hif1a Mouse shRNA Plasmid Kit	Origene	TL517255
pCMV6-AC-LRP6-GFP	Origene	RG218918
FLAG-HA-pcDNA3.1	Addgene	#52535
HRE-luciferase reporter	Addgene	#26731
pcDNA flag PPAR gamma	Addgene	#8895
pSynSRE-T-Luc	Addgene	#60444
pSynSRE-Mut-T-Luc	Addgene	#60490
pCDNA3-STAT3	Addgene	#74433
pcDNA3.1 HA-YY1	Addgene	#104395
pcDNA3-Cyto-GCaMP3	Addgene	#64853
pGL3-NFAT luciferase reporter	Addgene	#17870
PPRE X 3-TK-Luc	Addgene	#1015
STAT3 Reporter Kit	Addgene	#79730
YY1 Luciferase reporter	Panomics	LR0090
pCMV6-AC-NFATC1-GFP	Origene	PS100010
HA-HIF1alpha-pcDNA3	Addgene	#18949
pcDNA3.1-2xFLAG-SREBP-1a	Addgene	Addgene

qPCR primers

Wnt signalling genes

Genebank accession	Gene symbol	Forward Primer (5' to 3')	Reverse Primer (5' to 3')
NM_001130	*Aes*	ACCCCAGCAACTCAAATTCAC	AAGCCGTAGGACATCTCGTAG

NM_000038	*Apc*	AAAATGTCCCTCCGTTCTTATGG	CTGAAGTTGAGCGTAATACCAGT
NM_003502	*Axin1*	GGTTTCCCCTTGGACCTCG	CCGTCGAAGTCTCACCTTTAATG
NM_004655	*Axin2*	TACACTCCTTATTGGGCGATCA	TTGGCTACTCGTAAAGTTTTGGT
NM_004326	*Bcl9*	AGGGAGCGAAGTATTTCCGC	GGGTCATCGAGTGTGGTGT
NM_033637	*Btrc*	CCAGACTCTGCTTAAACCAAGAA	GGGCACAATCATACTGGAAGTG
NM_053056	*Ccnd1*	GCTGCGAAGTGGAAACCATC	CCTCCTTCTGCACACATTTGAA
NM_001759	*Ccnd2*	ACCTTCCGCAGTGCTCCTA	CCCAGCCAAGAAACGGTCC
NM_001892	*Csnk1a1*	AGTGGCAGTGAAGCTAGAATCT	CGCCCAATACCCATTAGGAAGTT
NM_001895	*Csnk2a1*	CGAGTTGCTTCCCGATACTTC	ACTTGCCAGCATACAACCCAA
NM_001328	*Ctbp1*	CGACCCTTACTTGTCGGATGG	TTGACGGTGAAGTCGTTGATG
NM_001904	*Ctnnb1*	CATCTACACAGTTTGATGCTGCT	GCAGTTTTGTCAGTTCAGGGA
NM_020248	*Ctnnbip1*	CCTATGCAGGGGTGGTCAAC	CGACCTGGAAAACGCCATCA
NM_025212	*Cxxc4*	ATGCACCACCGAAACGACTC	GCAGTGTTCAGGGGATAAGGT
NM_014992	*Daam1*	ACCAGATTCATCGACTTGGATGG	CGGCCTTGAGAGTTGTTCATTA
NM_001343	*Dab2*	GTAGAAACAAGTGCAACCAATGG	GCCTTTGAACCTTGCTAAGAGA
NM_033425	*Dixdc1*	GTGCAAAGAGCGAGTCCATTA	GGTCTCCAGATAGGTTCCAGG
NM_012242	*Dkk1*	CCTTGAACTCGGTTCTCAATTCC	CAATGGTCTGGTACTTATTCCCG
NM_015881	*Dkk3*	ATGTGTGCAAGCCGACCTT	CCTCAGCGCCATCTCTTCA
NM_004421	*Dvl1*	GAGGGTGCTCACTCGGATG	GTGCCTGTCTCGTTGTCCA
NM_004422	*Dvl2*	GAGGAAGAGACTCCCTACCTG	CGGGCGTTGTCATCTGAAAT
NM_001429	*Ep300*	AGCCAAGCGGCCTAAACTC	TCACCACCATTGGTTAGTCCC
NM_012300	*Fbxw11*	AAGCTGATTGAACGAATGGTACG	CCACACCGCCAGTTAGATTCTAT
NM_022039	*Fbxw4*	GTGCTTACACACCATCCAGAC	ACTGTTGAGGTCCCAGATTCT
NM_002007	*Fgf4*	CTCGCCCTTCTTCACCGATG	GTAGGACTCGTAGGCGTTGTA
NM_005438	*Fosl1*	CAGGCGGAGACTGACAAACTG	TCCTTCCGGGATTTTGCAGAT
NM_003593	*Foxn1*	CTGCTCGTCATTTGTGTCCGA	AGCCAAAGCCAGGATACTTGT
NM_005479	*Frat1*	GGTCCCAACCAGAAACCCG	GGGGAAGCTTTGCACGTAAC
NM_001463	*Frzb*	TGGAACATGACTAAGATGCCCA	ACACAGACTTACAGGGCTTGAT
NM_003505	*Fzd1*	ATCGAAGCCAACTCACAGTATTT	CACGTTGTTAAGCCCCACG
NM_001466	*Fzd2*	GTGCCATCCTATCTCAGCTACA	CTGCATGTCTACCAAGTACGTG

NM_017412	*Fzd3*	GTTCATGGGGCATATAGGTGG	GCTGCTGTCTGTTGGTCATAA
NM_012193	*Fzd4*	CCTCGGCTACAACGTGACC	TGCACATTGGCACATAAACAGA
NM_003468	*Fzd5*	CATGCCCAACCAGTTCAACC	CGGCGAGCATTGGATCTCC
NM_003506	*Fzd6*	ATGGCCTACAACATGACGTTT	GTTTACGACAAGGTGGAACCA
NM_003507	*Fzd7*	GTGCCAACGGCCTGATGTA	AGGTGAGAACGGTAAAGAGCG
NM_031866	*Fzd8*	ATCGGCTACAACTACACCTACA	GTACATGCTGCACAGGAAGAA
NM_003508	*Fzd9*	TGCGAGAACCCCGAGAAGT	GGGACCAGAACACCTCGAC
NM_019884	*Gsk3a*	GGAAAGGCATCTGTCGGGG	GAGTGGCTACGACTGTGGTC
NM_002093	*Gsk3b*	GGCAGCATGAAAGTTAGCAGA	GGCGACCAGTTCTCCTGAATC
NM_002228	*Jun*	TCCAAGTGCCGAAAAAGGAAG	CGAGTTCTGAGCTTTCAAGGT
NM_001039570	*Kremen1*	CCCGAGTGTTTCACAGCCAAT	GGATGCTGGAAAGTCTCGTTC
NM_016269	*Lef1*	TGCCAAATATGAATAACGACCCA	GAGAAAAGTGCTCGTCACTGT
NM_002335	*Lrp5*	TGGCCCGAAACCTCTACTG	GCACACTCGATTTTAGGGTTCT
NM_002336	*Lrp6*	ACGATTGTAGTTGGAGGCTTG	ATGGCTTCTTCGCTGACATCA
NM_002750	*Mapk8*	TGTGTGGAATCAAGCACCTTC	AGGCGTCATCATAAAACTCGTTC
NM_002423	*Mmp7*	GAGTGAGCTACAGTGGGAACA	CTATGACGCGGGAGTTTAACAT
NM_002467	*Myc*	GGCTCCTGGCAAAAGGTCA	CTGCGTAGTTGTGCTGATGT
NM_172390	*Nfatc1*	CACCGCATCACAGGGAAGAC	GCACAGTCAATGACGGCTC
NM_033119	*Nkd1*	GGGAAACTTCACTCCAAGCC	CTCCCGATCCACTCCTCGAT
NM_016231	*Nlk*	CCAACCTCCACACATTGACTATT	ACTTTGACATGATCTGAGCTGAG
NM_000325	*Pitx2*	CGGCAGCGGACTCACTTTA	GTTGGTCCACACAGCGATTT
NM_022825	*Porcn*	CGAGGGGCACAGATGATTGT	AAGTAGAGGTAGCCCATGAACT
NM_006238	*Ppard*	CAGGGCTGACTGCAAACGA	CTGCCACAATGTCTCGATGTC
NM_153026	*Prickle1*	GCTGCCTTGAGTGTGAAACG	TGCCCGTCATAGGTCATCTGT
NM_015617	*Pygo1*	CAGCTCCCGCTTACAAAGTTT	GGTCCCTGTGTATTTGCCTTG
NM_001664	*Rhoa*	GGAAAGCAGGTAGAGTTGGCT	GGCTGTCGATGGAAAAACACAT
NM_021205	*Rhou*	GCTACCCCACCGAGTACATC	GGCTCACGACACTGAAGC
NM_003707	*Ruvbl1*	AGGTGAAGAGCACTACGAAGA	CTACTATGACGCCACATGCCT
NM_003012	*Sfrp1*	ACGTGGGCTACAAGAAGATGG	CAGCGACACGGGTAGATGG
NM_003014	*Sfrp4*	CCTGGAACATCACGCGGAT	CGGCTTGATAGGGTCGTGC
NM_022454	*Sox17*	GTGGACCGCACGGAATTTG	GGAGATTCACACCGGAGTCA
NM_003202	*Tcf7*	TTGATGCTAGGTTCTGGTGTAC	CCTTGGACTCTGCTTGTGTC

		C	
NM_031283	*Tcf7l1*	TCGTCCCTGGTCAACGAGT	ACTTCGGCGAAATAGTCCCG
NM_005077	*Tle1*	GAGTCCCTGGACCGGATTAAA	AATACATCACATAGTGCCTCTG
NM_020335	*Vangl2*	ACCGCTCTAAGAGTCGAGATG	GTTACTACTGTCGTCGTTTCCC
NM_007191	*Wif1*	TCTCCAAACACCTCAAAATGCT	GACACTCGCAGATGCGTCT
NM_003882	*Wisp1*	CCAGCCTAACTGCAAGTACAA	GGCGTCGTCCTCACATACC
NM_005430	*Wnt1*	CGATGGTGGGGTATTGTGAAC	CCGGATTTTGGCGTATCAGAC
NM_025216	*Wnt10a*	GGTCAGCACCCAATGACATTC	TGGATGGCGATCTGGATGC
NM_004626	*Wnt11*	GGAGTCGGCCTTCGTGTATG	GCCCGTAGCTGAGGTTGTC
NM_057168	*Wnt16*	AGTATGGCATGTGGTTCAGCA	GCGGCAGTCTACTGACATCAA
NM_003391	*Wnt2*	GATGCGTGCCATTAGCCAG	AGATTCCCGACTACTTCGGAG
NM_004185	*Wnt2b*	CGGGACCACACCGTCTTTG	GCGAGTAATAGCGTGGACTAC
NM_030753	*Wnt3*	AGGGCACCTCCACCATTTG	GACACTAACACGCCGAAGTCA
NM_033131	*Wnt3a*	TGACACGCTCATGTGCAGAA	CGAGACACCATCCCACCAAA
NM_030761	*Wnt4*	GTACGCCATCTCTTCGGCAG	GCGATGTTGTCAGAGCATCCT
NM_003392	*Wnt5a*	ATTCTTGGTGGTCGCTAGGTA	CGCCTTCTCCGATGTACTGC
NM_032642	*Wnt5b*	CGCTTCGCCAAGGAGTTTG	TGCCATCTTATACACAGCCCT
NM_006522	*Wnt6*	GGTGCGAGAGTGCCAGTTC	CGTCTCCCGAATGTCCTGTT
NM_004625	*Wnt7a*	CTGTGGCTGCGACAAAGAGAA	GCCGTGGCACTTACATTCC
NM_058238	*Wnt7b*	GAAGCAGGGCTACTACAACCA	CGGCCTCATTGTTATGCAGGT
NM_058244	*Wnt8a*	GAACTGCCCTGAAAATGCTCT	TCGAAGTCACCCATGCTACAG
NM_003395	*Wnt9a*	GGCAAGCATCTGAAGCACAAG	GCAGAAGCTAGGCGAGTCA
NM_001101	*Actb*	CATGTACGTTGCTATCCAGGC	CTCCTTAATGTCACGCACGAT
NM_004048	*B2m*	GAGGCTATCCAGCGTACTCCA	CGGCAGGCATACTCATCTTTT
NM_002046	*Gapdh*	ACAACTTTGGTATCGTGGAAGG	GCCATCACGCCACAGTTTC
NM_000194	*Hprt1*	CCTGGCGTCGTGATTAGTGAT	AGACGTTCAGTCCTGTCCATAA
NM_001002	*Rplp0*	AGCCCAGAACACTGGTCTC	ACTCAGGATTTCAATGGTGCC

Mouse KEGG pathway genes

Genebank accession	Gene symbol	Forward Primer (5' to 3')	Reverse Primer (5' to 3')
NM_030210	*Aacs*	GGGAGCCTGACAGCAAGAAG	CGGACAGACCAGTGGTATAAGTC
NM_011834	*Aadat*	ATGAATTACTCACGGTTCCTCAC	AACATGCTCGGGTTTGGAGAT
NM_009591	*Aanat*	TGAGCGGGAAGCCTTTATCTC	CTCCTGAGTAAGTCTCTCCTTGT
NM_173765	*Aasdh*	TCGGACTGCCTCTGTCTATATG	GGGTAAGTTTATCCCAGGTTG

			AC
NM_026276	*Aasdhppt*	TTCCCCGCTAAGAGGCTGT	GCATCGCCAGAAGCCATTC
NM_013930	*Aass*	GGCATCACCAAACTGGGCTA	GTGTGGGAAAAGAAGGCGTAT
NM_001170978	*Abat*	CTGAACACAATCCAGAATGCAGA	GGTTGTAACCTATGGGCACAG
NM_013454	*Abca1*	GCTTGTTGGCCTCAGTTAAGG	GTAGCTCAGGCGTACAGAGAT
NM_021022	*Abcb11*	TCTGACTCAGTGATTCTTCGCA	CCCATAAACATCAGCCAGTTGT
NM_031884	*Abcg5*	AGGGCCTCACATCAACAGAG	GCTGACGCTGTAGGACACAT
NM_026180	*Abcg8*	CTGTGGAATGGGACTGTACTTC	GTTGGACTGACCACTGTAGGT
NM_177470	*Acaa2*	CTGCTACGAGGTGTGTTCATC	AGCTCTGCATGACATTGCCC
NM_133360	*Acaca*	ATGGGCGGAATGGTCTCTTTC	TGGGGACCTTGTCTTCATCAT
NM_133904	*Acacb*	CGCTCACCAACAGTAAGGTGG	GCTTGGCAGGGAGTTCCTC
NM_025862	*Acad8*	ACCTGCTTCAGCGACTAGC	TCAGCCCTTTGGTCTACTTCA
NM_007381	*Acadl*	TCTTTTCCTCGGAGCATGACA	GACCTCTCTACTCACTTCTCCAG
NM_007382	*Acadm*	AGGGTTTAGTTTTGAGTTGACGG	CCCCGCTTTTGTCATATTCCG
NM_007383	*Acads*	TGGCGACGGTTACACACTG	GTAGGCCAGGTAATCCAAGCC
NM_025826	*Acadsb*	CCCAACCTGCTTGTCTCCTTG	ATCCCTGGATCACCGATTTCT
NM_017366	*Acadvl*	CTACTGTGCTTCAGGGACAAC	CAAAGGACTTCGATTCTGCCC
NM_144784	*Acat1*	CAGGAAGTAAGATGCCTGGAAC	TTCACCCCCTTGGATGACATT
NM_009338	*Acat2*	CCCGTGGTCATCGTCTCAG	GGACAGGGCACCATTGAAGG
NM_175731	*Acer1*	TCTGAGGTGGATTGGTGTGAG	TGAGGGGTCCAAAGATGAGGA
NM_139306	*Acer2*	TGTGGCATATTCTCATCTGCCT	CAATAAAAGCCCATTTCTCGCTG
NM_025408	*Acer3*	TGTGATTCACTGAGGAACTTTCG	AGAAACTTCACTTTTGGCCTGTA
NM_009599	*Ache*	CTCCCTGGTATCCCCTGCATA	GGATGCCCAGAAAAGCTGAGA
NM_134037	*Acly*	CAGCCAAGGCAATTTCAGAGC	CTCGACGTTTGATTAACTGGTCT
NM_001033041	*Acmsd*	CTACCAAAGGAATGGCCCGAT	GTGGAAAGAGCTTGGACTGTC
NM_007386	*Aco1*	AGAACCCATTTGCACACCTTG	AGCGTCCGTATCTTGAGTCCT
NM_080633	*Aco2*	ATCGAGCGGGGAAAGACATAC	TGATGGTACAGCCACCTTAGG
NM_012006	*Acot1*	ATACCCCCTGTGACTATCCTGA	CAAACACTCACTACCCAACTGT
NM_028790	*Acot12*	GTTTGAGGACACAGCGAGAAT	GAAGCAGGACAGGTTTTAAGTGA
NM_134188	*Acot2*	GTTGTGCCAACAGGATTGGAA	GCTCAGCGTCGCATTTGTC
NM_134247	*Acot4*	AGCAGTGCGGTACATGCTTC	AGAGCCATTGATGGAAACTGTG

NM_001146057	*Acot7*	TAGCCTTTTCGAGCCTGACG	TTGGCATCATCTGGACGCAT
NM_015729	*Acox1*	TAACTTCCTCACTCGAAGCCA	AGTTCCATGACCCATCTCTGTC
NM_030721	*Acox3*	ACCGGAAGAAAAAGACAGTGC	GAGGCTCTTGCTCGGTAGG
NM_007981	*Acsl1*	TGCCAGAGCTGATTGACATTC	GGCATACCAGAAGGTGGTGAG
NM_028817	*Acsl3*	AACCACGTATCTTCAACACCATC	AGTCCGGTTTGGAACTGACAG
NM_019477	*Acsl4*	CTCACCATTATATTGCTGCCTGT	TCTCTTTGCCATAGCGTTTTTCT
NM_027976	*Acsl5*	TCCTGACGTTTGGAACGGC	CTCCCTCAATCCCCACAGAC
NM_001033599	*Acsl6*	AAGTGACAGAGAGTCAGTGGG	TAGGGCGGAGAGCCTTCAT
NM_054094	*Acsm1*	TCTGCAAAGTCTTACAGGGCT	TCCTCAGGTGAATCATGGTCA
NM_212442	*Acsm3*	CTTTGGCCCCAGCAGTAGATG	GGCTGTCACTGGCATATTTCAT
NM_178758	*Acsm5*	CCGATCCCTGAGGTGGTAG	GGTGCCCTGTCTTTTCCAG
NM_080575	*Acss1*	GTTTGGGACACTCCTTACCATAC	AGGCAGTTGACAGACACATTC
NM_019811	*Acss2*	AAACACGCTCAGGGAAAATCA	ACCGTAGATGTATCCCCCAGG
NM_198636	*Acss3*	AATGTCGCAAAGTAACAGGCG	GTGGGTCTTGTACTCACCACC
NM_025371	*Acy1*	CAACCCAATCCAGACTATGGAGG	GTGGGAGTTTAGCAAGATGGAG
NM_027857	*Acy3*	GCACCCACGGGAATGAGATG	GAGCGGTTGAGATCACGGTC
NM_025421	*Acyp1*	AAAGGTTCAAGGGGTGTTTTTCC	CATGGTAGGGGAGAACGGACT
NM_029344	*Acyp2*	TGCTCAAGTCTGTGGACTACG	GAGAACTAGGACTCCCAACCTT
NM_007398	*Ada*	ACCCGCATTCAACAAACCCA	AGGGCGATGCCTCTCTTCT
NM_009622	*Adcy1*	GTCACCTTCGTGTCCTATGCC	TTCACACCAAAGAAGAGCAGG
NM_173029	*Adcy10*	AAGCTCATCCGCTCTATGCC	CCAAGCGTGATGTAGGTGGT
NM_153534	*Adcy2*	GACTGGCTCTACGAGTCCTAC	GGGCAGTGGGAACGGTTAT
NM_001159537	*Adcy3*	CTCGCTTTATGCGGCTGAC	ACATCACTACCACGTAGCAGT
NM_080435	*Adcy4*	AGTACCCACTGCTGATACTGC	AGCCACCCAAAGCACACAG
NM_001012765	*Adcy5*	CTTGGGGAGAAGCCGATTCC	ACCGCTTAGTGGAGGGTCT
NM_007405	*Adcy6*	GATGAACGGAAAACAGCTTGGG	GGTGGCTCCGCATTCTTGA
NM_001109756	*Adcy7*	AAGGGGCGCTACTTCCTAAAT	GTGTCTGCGGAGATCCTCA
NM_009623	*Adcy8*	CTCTACACCATCCAACCGACG	GCACCGAGTCGCTAGACAG
NM_009624	*Adcy9*	CAACAGCGTGAGGGTCAAGAT	CATGGAGTCGAATTTGGGGTC
NM_011996	*Adh4*	TGGCAGTCCCCTTTGCATT	ACTACCGGGAAGAGAGCTTTC
NM_007410	*Adh5*	AGTTCGGATTAAGATCCTTGCCA	ACTTTCCACAATTCCAGCACC
NM_009626	*Adh7*	ATGGGCACCGCTGGAAAAG	TAACACGGACTTCCTTAGCCT

NM_134052	*Adi1*	CACGCTCGGAGTGCTCTATTG	AGGATGTAGCGGATCTCCTCA
NM_134079	*Adk*	AGAGTCGGTATTGAAAGTGGCT	CTAAAGCAACTCACCGTCTCAT
NM_009634	*Adsl*	AGCCGCGAGATGTGTTTCTT	TCAATGTTGTTCAGGTTCGACTT
NM_009634	*Adsl*	AGCCGCGAGATGTGTTTCTT	TCAATGTTGTTCAGGTTCGACTT
NM_007422	*Adss*	ACACGGGGTAGAGAATTTGGA	GGTAAGGGCCAACGCAGTA
NM_007421	*Adssl1*	CTCACCTTGTGTTCGACTTCC	AGCAAAGCCCTTGAGCCTTTT
NM_027827	*Afmid*	AAAGATTGGCAGTGCCTAGATG	TCCAGCCAGGGTTATACAGTG
NM_023538	*Agk*	CTCATTCCTCCCAATGCACAA	TCCATGCCAGACAGGTGTAAA
NM_001081326	*Agl*	GTCCACAGATCGACCGAAACA	GTGCCACAGTTGAAGCGATTT
NM_001081408	*Agmat*	AGTGGTACAGATAGGCATCCG	AGGGGGACCAGTGACTTCAT
NM_001163379	*Agpat1*	TAAGATGGCCTTCTACAACGGC	CCATACAGGTATTTGACGTGGAG
NM_026212	*Agpat2*	CAGCCAGGTTCTACGCCAAG	TGATGCTCATGTTATCCACGGT
NM_053014	*Agpat3*	CTGCTTGCCTACCTGAAGACC	GATACGGCGGTATAGGTGCTT
NM_026644	*Agpat4*	CCAGTTTCTATGTCACCTGGTC	GCAGAGTCTGGCATTGATCTTG
NM_172666	*Agps*	AATGGATGGGGCTACAATGATTC	TCCAGACTTACTCCAAGGGTG
NM_016702	*Agxt*	AAGCCCCTGTCAGTTCCTAC	TCCTCCATGATCTGAAGCATCT
NM_001031851	*Agxt2*	TCACCTGAGAAATACCAGTCCC	CAAAGAGCCACTCCATGTGTC
NM_016661	*Ahcy*	GGACCGCTACTGGCTAAAGAA	GCAGGAAGTGAACCCCAACA
NM_145542	*Ahcyl1*	TCGAGGACGCCGAGAAGTA	GGGAATTTGGTGAACTCTTGCAT
NM_001171001	*Ahcyl2*	TTCAACAAACGTCCCACCAAA	GGATGTCTCATCGTCAGAGCTAT
NM_001198792	*Ak1*	GCTGCTGTGTGTCTAGTGAAC	CCTCTCCGATCCAGAGCTGA
NM_001033966	*Ak2*	GGCTTCCGAACCGGAGATTC	CAGACACAAAAGTTTTCAGCCAG
NM_021299	*Ak3*	GTGTCGTCACGCATCACCAA	GCCAACACACCGATTTCTGT
NM_001081277	*Ak5*	AGAAGCAAACTGATCCGTGAC	CTTCGTCCAAACTTTTCCCCTT
NM_030187	*Ak7*	CAGCAAAAATACCGGCCCTG	TTCTCCACGAAGCCTGTTTGG
NM_021473	*Akr1a1*	AGCCTGGTCAGGTGAAAGC	GGCCTCCCCAATCTCAGTT
NM_172398	*Akr1b10*	CTAGTGCCAAACCAGAGGACC	TCCTGTATTCGAGAAGGTGTCA
NM_030611	*Akr1c1*	GGACTAGCCATCCGAAGCAA	AGACCCGTACGAGTTCTGGA
NM_145364	*Akr1d1*	TGCACACCACCAAATATCCCT	CTTCACTGCCACATAGGTCTTC
NM_020559	*Alas1*	TCGCCGATGCCCATTCTTATC	GGCCCCAACTTCCATCATCT
NM_009653	*Alas2*	GCAGGGCAACAGGACTTTG	ACAGGACCGTAGCAACATAGC
NM_153554	*Aldh18a1*	GTCCAGCCCTCAGCTATTAGA	CTTCAGCTCACTTCGGTGGG

NM_053080	*Aldh1a3*	GGGTCACACTGGAGCTAGGA	CTGGCCTCTTCTTGGCGAA
NM_028270	*Aldh1b1*	CTCCAGGGCAGGACTACCTC	CATGCCACTCGTTGTTGATGA
NM_009656	*Aldh2*	GACGCCGTCAGCAGGAAAA	CGCCAATCGGTACAACAGC
NM_007437	*Aldh3a2*	GCGGAGGATGGTGCAAGAG	CATTGAGTTCACTTTTGCTCAGG
NM_026316	*Aldh3b1*	ATGGACTCGTTTGAAGACAAGC	GATGGCAATCTCAGACACCTC
NM_001177438	*Aldh3b2*	TTTGGAGGCAACGATGGCTT	CAGGGACCATAAGGCGGGTA
NM_175438	*Aldh4a1*	CGATGGAAGCACACCTCTTCT	GGCGACAACTGGTACTGTATATC
NM_172532	*Aldh5a1*	CGGTCAAGGAGAGGAGCTTAC	GGACTAGCCCTCGCTTATCTTT
NM_134042	*Aldh6a1*	AGCCGTTGAGTCCTGCAAAC	TCCCTTGTTCCAGTGTGATTAAC
NM_138600	*Aldh7a1*	TGCTGATCCATCATCCCCAGT	TGTTAGCAGGACAATAGGTCGT
NM_019993	*Aldh9a1*	GGCCGAGTGATTGCCACTT	AGGCCACTTTTCTTACTCCAGA
NM_001177308	*Aldoa*	CGTGTGAATCCCTGCATTGG	CAGCCCCTGGGTAGTTGTC
NM_144903	*Aldob*	GAAACCGCCTGCAAAGGATAA	GAGGGTCTCGTGGAAAAGGAT
NM_009657	*Aldoc*	AGAAGGAGTTGTCGGATATTGCT	TTCTCCACCCCAATTTGGCTC
NM_053156	*Allc*	TCTGAGTGCGTGGGAGGAAA	AATGCCCTGTATCCCCAGTTG
NM_007440	*Alox12*	GTTCCACACATCCGTTACACT	CCGAGTAAGCAACTGAACATGG
NM_009659	*Alox12b*	GGGGACGCTGGATTCAATTTC	GGCACTGTACCGTGTAGTCAT
NM_009660	*Alox15*	GGCTCCAACAACGAGGTCTAC	AGGTATTCTGACACATCCACCTT
NM_009662	*Alox5*	ACTACATCTACCTCAGCCTCATT	GGTGACATCGTAGGAGTCCAC
NM_009665	*Amd1*	AGGGATCTGGGGATCTTCGTA	TGCTTGTCAGTCTTTGTCACAC
NM_027908	*Amdhd1*	GTGGGCGGGTGAAAGAGTC	GGAGCAGAACAGTTCCTCCTC
NM_172935	*Amdhd2*	GATGTGCAGATCAACGGTGGA	CCTTGTGATAAACCTCTGGTGG
NM_001033303	*Ampd1*	GATGCAATGCGTAGCTTTGCT	CCTTGGAAACGCCTTTTTCTCC
NM_028779	*Ampd2*	CCTCTCCGCTACAGTCTGC	CTCCTTGCATTTGCCATCCAT
NM_009667	*Ampd3*	GTTGGCGGAGAAGGTGTTTG	CTGCGACCGGATCATCTTGAA
NM_001013814	*Amt*	CAGGCACAACCCTTGGTCC	TGCAGGTGTGAATCAACATGAC
NM_013913	*Angptl3*	GAGGAGCAGCTAACCAACTTAAT	TCTGCATGTGCTGTTGACTTAAT
NM_020581	*Angptl4*	CATCCTGGGACGAGATGAACT	TGACAAGCGTTACCACAGGC
NM_178932	*Aoc2*	CGAGAGGCACTAGCCATCATC	GCGCCGATAATAGGGCAGG
NM_009675	*Aoc3*	GAAGACCACCCTAGTGCTCCT	ATGAAGAGGTTGGCTCAGTCC
NM_009676	*Aox1*	GAGGAAGAATCTCCGACTCACA	TGGTGACTGCTGTACCATGTAG

NM_019735	*Apip*	CAAGGAGCACCCCCGATTC	TTGCCATGCTTCAAGCTGATT
NM_013474	*Apoa2*	TGGTCGCACTGCTGGTAAC	TTTGCCATATTCAGTCATGCTCT
NM_007468	*Apoa4*	CCAATGTGGTGTGGGATTACTT	AGTGACATCCGTCTTCTGAAAC
NM_009693	*Apob*	AAGCACCTCCGAAAGTACGTG	CTCCAGCTCTACCTTACAGTTGA
NM_001110009	*Apoc1*	TCCTGTCCTGATTGTGGTCGT	CCAAAGTGTTCCCAAACTCCTT
NM_009695	*Apoc2*	ATGGGGTCTCGGTTCTTCCT	GTCTTCTGGTACAGGTCTTTGG
NM_023114	*Apoc3*	TACAGGGCTACATGGAACAAGC	CAGGGATCTGAAGTGATTGTCC
NM_009696	*Apoe*	CTGACAGGATGCCTAGCCG	CGCAGGTAATCCCAGAAGC
NM_013475	*Apoh*	TGCCATGTTGCTATTGCAGGA	GGCTTGCAGGAGTAGACAATCT
NM_009698	*Aprt*	CCCTCTTGAAAGACCCGGAC	TCCAGAGAATAGGAGGCTGAC
NM_007482	*Arg1*	CTCCAAGCCAAAGTCCTTAGAG	AGGAGCTGTCATTAGGGACATC
NM_009705	*Arg2*	TCCTCCACGGGCAAATTCC	GCTGGACCATATTCCACTCCTA
NM_009713	*Arsa*	TAACATCCTGCTGATCTTTGCG	CTGTGAACCGTAGTCCACCTT
NM_019734	*Asah1*	CGTGGACAGAAGATTGCAGAA	TGGTGCCTTTTGAGCCAATAAT
NM_138679	*Ash1l*	CCTCGGTGGACTAAAGTGGTG	CGCTGGCTCAGAACTATTTGA
NM_133768	*Asl*	CTATGACCGGCATCTGTGGAA	AGCAACCTTGTCCAACCCTTG
NM_012055	*Asns*	GCAGTGTCTGAGTGCGATGAA	TCTTATCGGCTGCATTCCAAAC
NM_023113	*Aspa*	ACATGGCTGCTGTTATTCATCC	GGGTACACGGTACAGTCTCCA
NM_007494	*Ass1*	ACACCTCCTGCATCCTCGT	GCTCACATCCTCAATGAACACCT
NM_026195	*Atic*	GCCTCGTGGAATTTGCCAGA	AACCCTGTTAGCTCAGACACA
NM_138652	*Atp12a*	ATGCGCCGGAAAACAGAAATC	CCTCCTCCTGACTCTTGTTGG
NM_018731	*Atp4a*	GATGGAGATTAACGACCACCAG	ACGGGCAAACTTCACATACTC
NM_009724	*Atp4b*	CAGGAGAAGAAGTCATGCAGC	GAAACCTGCGTAGTACAGGCT
NM_007505	*Atp5a1*	TCTCCATGCCTCTAACACTCG	CCAGGTCAACAGACGTGTCAG
NM_016774	*Atp5b*	GGTTCATCCTGCCAGAGACTA	AATCCCTCATCGAACTGGACG
NM_001112738	*Atp5c1*	CCAGGAGACTGAAGTCCATCA	AGAACCTGTCCCATACACTCG
NM_025313	*Atp5d*	TGCTTCAGGCGCGTACATAC	CACTTGCTTGACGTTGGCA
NM_025983	*Atp5e*	CAGGCTGGACTCAGCTACATC	GTTCGCTTTGAACTCGGTCTT
NM_009725	*Atp5f1*	AGTTCCTTTACCCTAAGACTGGT	TTCATGCTCGACTGCTTTACTT
NM_001161419	*Atp5g1*	CCAGAGGCCCCATCTAAGC	CCCCAGAATGGCATAGGAGAAG
NM_026468	*Atp5g2*	CAGTGGAGTTGAAGCGACCA	TGTCGATGTCCCTTGAAATGG
NM_175015	*Atp5g3*	TCTGCATCAGTGTTATCTCGGC	CACCAGAACCAGCAACTCCTA
NM_020582	*Atp5J2*	TATTGGCCCAGAGTATCAGCA	GGGGTTTGTCGATGACTTCAA

			AT
NM_020582	*Atp5j2*	TGCCGAGCTGGATAATGATGC	ACCATGCTAATCCCCGAGATG
NM_013795	*Atp5l*	GAGAAGGCACCGTCGATGG	ACACTCTGAATAGCTGTAGGGAT
NM_138597	*Atp5o*	TCTCGACAGGTTCGGAGCTT	AGAGTACAGGGCGGTTGCATA
NM_018794	*Atp6ap1*	GGCGGCAACAGTGGTATCTC	CACAGATTCCGGTCACTCGAC
NM_001243051	*Atp6v0a1*	GGACCGACAGAGGAGGATG	GCCAAAGTCAAACTCTTCTGCG
NM_011596	*Atp6v0a2*	TGGTGCAGTTCCGAGACCT	GCAGGGGAATATCAGCTCTGG
NM_080467	*Atp6V0a4*	TGGAGGCTGCGTATTGCTG	CCTTCGGACTTCATTCACAAACT
NM_033617	*Atp6V0b*	AGTTGCTCTACCTCGGGATCT	ATGCCACATCAAAGCGAAAGC
NM_009729	*Atp6v0c*	TGTCCCGTTGTCCTAGCTC	CCATGACACCGAAAAACGAAGA
NM_013477	*Atp6v0d1*	GCTACTTGGAGGGATTAGTGCG	GCGGAACTCTACTACCATCTTCT
NM_175406	*Atp6v0d2*	CAGAGCTGTACTTCAATGTGGAC	AGGTCTCACACTGCACTAGGT
NM_133764	*Atp6V0e2*	CTGGTTCGTGCCCAAAGGA	GGCAATGAGCCAGAAGAGGTAA
NM_007508	*Atp6v1a*	CTACCCAAAATCCGCGATGAG	CCATGTCACCTTCCAATCGAA
NM_134157	*Atp6v1b1*	AAGTTTGCCCAGTATGCTGAG	GCAGGATGTCCCCTGTGAA
NM_007509	*Atp6v1b2*	ATGCGGGGAATCGTGAACG	AGGCTGGGATAGGTAGTTCCG
NM_025494	*Atp6v1c1*	ACTGAGTTCTGGCTCATATCTGC	TGGAAGAGACGGCAAGATTATTG
NM_001159632	*Atp6v1c2*	TCTGAGTTTTGGCTTATTTCGGC	TGTTGTGGGACAGGTTAGACTTT
NM_023721	*Atp6V1d*	GGCAAAGACCGGATTGAAATCT	GTCGAAATCGAAGAGTTAAGGCA
NM_007510	*Atp6v1e1*	AGGTCGCCTTCTCGAAACG	ATGAGGTCATCCCTTGCTCTG
NM_029121	*Atp6v1e2*	CACCCTGGAAACTCTGTTCTAC	CTGCACGTCTATGTCAGTCAG
NM_025381	*Atp6v1f*	GCGGGCAGAGGTAAGCTAATC	TTAGGGTGGCGGTTCTTGTTT
NM_024173	*Atp6v1g1*	CCCAGGCTGAAATTGAACAGT	TTCTGGAGGACGGTCATCTTC
NM_023179	*Atp6v1g2*	GAGGAGGCTCAAATGGAGGTG	CTGAACCTGCCGTCTTGTG
NM_177397	*Atp6v1g3*	GCCAAGGACAAGCTAGATGAG	AGCCCATTATCTTAGCCTGCT
NM_133826	*Atp6V1h*	GGATGCTGCTGTCCCAACTAA	TCTCTTGCTTGTCCTCGGAAC
NM_016709	*Auh*	AGCTGGCTCTAGCGTGTGA	GTTCTGTTCTAACACATGGCTGA
NM_022305	*B4galt1*	ATGAGGTTTCGTGAGCAGTTC	AGAGGTAATAGACGAGGGTGAC
NM_017377	*B4galt2*	TGCCTGCTGCACTTCCTTG	CAGGGCGGAATGACTGGAG
NM_019737	*B4galt6*	GGGTCTCCAATCGCTCTCTG	ATAAAGAGGTACGTGTTGGCG
NM_007519	*Baat*	GGAAACCTGTTAGTTCTCAGG	GTGGACCCCCATATAGTCTCC

		C	
NM_130452	*Bbox1*	ATGGGGCTCATTTGATGCAGA	GAAGTTTCCGAGCTTTTGCAG
NM_007532	*Bcat1*	CCCATCGTACCTCTTTCACCC	GGGAGCGTGGGAATACGTG
NM_001243052	*Bcat2*	CAGCCACACTAGGACAGGTCT	CAGCCTTGTTATTCCACTCCAC
NM_007533	*Bckdha*	CTCCTGTTGGGACGATCTGG	CATTGGGCTGGATGAACTCAA
NM_199195	*Bckdhb*	CTTCCGATGCACTGTTGGTTT	GATTTCCGCAATAGCTGTAGC A
NM_175177	*Bdh1*	ACAAGACACACGCTGTTGTTT	CTCTTCAAGCTGTCCAGTTCC
NM_027208	*Bdh2*	CGACTGGACGGCAAAGTTATT	CCTGGAGTTTGGACTCGTTGA
NM_016668	*Bhmt*	TTAGAACGCTTAAATGCCGGA G	GATGAAGCTGACGAACTGCCT
NM_007563	*Bpgm*	GGACCAGAAACTTAACAACGA CG	CAGGCTGTGTGAATGGACCT
NM_023525	*Cad*	CTGCCCGGATTGATTGATGTC	GGTATTAGGCATAGCACAAAC CA
NM_029502	*Cant1*	GCGGGAATGGAATGAGCCTAT	CCCTGATGGTGGGAGTACAG
NM_009804	*Cat*	AGCGACCAGATGAAGCAGTG	TCCGCTCTCTGTCAAAGTGTG
NM_007620	*Cbr1*	TCAATGACGACACCCCCTTC	CCTCTGTGATGGTCTCGCTTC
NM_173047	*Cbr3*	GTAACTGGGGCTAACAAAGGC	TTGACCAGCACGTTAAGTCCC
NM_178224	*Cbs*	GGGACAAGGATCGAGTCTGG A	AGCACTGTGTGATAATGTGGG
NM_001159556	*Cd36*	ATGGGCTGTGATCGGAACTG	GTCTTCCCAATAAGCATGTCTC C
NM_028176	*Cda*	GATCTTCTCTGGGTGCAACATA G	CCTGAAATCCTTGTACCCTTCG
NM_138754	*Cdipt*	TCCTGTTCGTGCCTAACCTTA	AGGAGTCCGCTGAGTAGATAG A
NM_033037	*Cdo1*	GGGGACGAAGTCAACGTGG	ACCCCAGCACAGAATCATCAG
NM_173370	*Cds1*	GGTGACCACGAAACCGAGAG	CCGCGAATCCACCAGTTCT
NM_138651	*Cds2*	ATGACCGAACTACGGCAGAG	GAGGTTGGTAGGGGAGCTG
NM_009885	*Cel*	CGCCTGGAGGTTCTATTTCTTG	GCCCTTGAAGATGTCAACAGA
NM_145475	*Cerk*	TCCGTGCTGTGGGTGAAAC	CGCAGTCGTCTTTTTCCTCAA
NM_009891	*Chat*	CCATTGTGAAGCGGTTTGGG	GCCAGGCGGTTGTTTAGATAC A
NM_175343	*Chdh*	CAGGCCCTTAGAGGCTGGA	CAAAGGTGTACTCATCCTTGCC
NM_023186	*Chia*	CAAGCTACTTCTCGTCACAGG	GGCAGGGGTTAATGTCATCAG
NM_027979	*Chit1*	TGGGCAGGTGTGATGACTCT	CCCTGGGAAAGAACCGAACTG
NM_001025566	*Chka*	GGGCCTACCTGTGGTGTAAG	CGCCTCAGCCCCTTGAAAT
NM_007692	*Chkb*	AGGATGCTAAGTGCCCAGAG	TCACGGGACAAACGCTCAG
NM_144807	*Chpt1*	ACTGAGATCCAGGTAGCTTTA GT	GTAGACCCATTCTTGCCAACA
NM_021273	*Ckb*	AGTTCCCTGATCTGAGCAGC	GAATGGCGTCGTCCAAAGTAA
NM_007710	*Ckm*	CTGACCCCTGACCTCTACAAT	CATGGCGGTCCTGGATGAT

NM_198415	*Ckmt2*	ACACCCAGTGGCTATACCCTG	CCGTAGGATGCTTCATCACCC
NM_009908	*Cmas*	CTTCCAGAGTGTGTGGGTTTC	CGTCTAGTGAGGTAGAGCTGTC
NM_025647	*Cmpk1*	TGAAGCCGTTGGTCGTGTTC	ACCATACTGTGAGTCTGGATTCT
NM_020557	*Cmpk2*	CCTTCAGGAACAAGTGTGCAT	TGGGAAGAACACGTAGACACAT
NM_177450	*Cndp1*	TTCTCTTCGGCACACTCTGG	GCTTCTGTCTGAGACGAGGC
NM_007744	*Comt*	CTGGGGGTTGGTGGCTATTG	CCCACTCCTTCTCTGAGCAG
NM_178379	*Cox10*	AGAAGAGCTATACAGGGATTGCC	CTGTGTGACATACATGCGCTT
NM_199008	*Cox11*	GTGCCCCTCTATCGGCTCTA	TAATGACCCGGTCCTTGACAG
NM_144874	*Cox15*	TAGGGTGGCGTCCAGAACA	GGTCACTCCTCCCAGAATAACTG
NM_009941	*Cox4l1*	ATTGGCAAGAGAGCCATTTCTAC	CACGCCGATCAGCGTAAGT
NM_053091	*Cox4l2*	CTGCCCGGAGTCTGGTAATG	CAGTCAACGTAGGGGGTCATC
NM_007747	*Cox5a*	GCCGCTGTCTGTTCCATTC	GCATCAATGTCTGGCTTGTTGAA
NM_009942	*Cox5b*	TTCAAGGTTACTTCGCGGAGT	CGGGACTAGATTAGGGTCTTCC
NM_007748	*Cox6a1*	TCAACGTGTTCCTCAAGTCGC	AGGGTATGGTTACCGTCTCCC
NM_009943	*Cox6a2*	CTGCTCCCTTAACTGCTGGAT	GATTGTGGAAAAGCGTGTGGT
NM_025628	*Cox6b1*	ACTACCTGGACTTCCACCG	ACCCATGACACGGGACAGA
NM_053071	*Cox6c*	GCGTCTGCGGGTTCATATTG	TCTGCATACGCCTTCTTTCTTG
NM_009944	*Cox7aa*	GCTCTGGTCCGGTCTTTTAGC	GTACTGGGAGGTCATTGTCGG
NM_009945	*Cox7a2*	GCTGGCCCTTCGTCAGATT	GGCATCCCATTATCCTCCTGAA
NM_009187	*Cox7a2l*	AAGTTTAGCAGTTTCACGCAGA	GTGTGGCAAATATGATAGGTGGT
NM_025379	*Cox7b*	TTGCCCTTAGCCAAAAACGC	TCATGGAAACTAGGTGCCCTC
NM_030052	*Cox7b2*	TCTCAAGACTCCAAGCATTCTGA	GACAGGTTCCACTCCACACC
NM_007749	*Cox7c*	ATGTTGGGCCAGAGTATCCG	ACCCAGATCCAAAGTACACGG
NM_001039049	*Cox8c*	GACTGGTGGTCTCCGGTTTG	GGCTTCGAGAACAGGACTGC
NM_001080809	*Cps1*	ACATGGTGACCAAGATTCCTCG	TTCCTCAAAGGTGCGACCAAT
NM_013495	*Cpt1a*	CTCCGCCTGAGCCATGAAG	CACCAGTGATGATGCCATTCT
NM_009948	*Cpt1b*	GCACACCAGGCAGTAGCTTT	CAGGAGTTGATTCCAGACAGGTA
NM_153679	*Cpt1c*	TCTTCACTGAGTTCCGATGGG	ACGCCAGAGATGCCTTTTCC
NM_009949	*Cpt2*	CAGCACAGCATCGTACCCA	TCCCAATGCCGTTCTCAAAAT
NM_030004	*Cryl1*	AGGAGTGTGTTCCAGAGAACC	TGGTGGATTGACAGGATGAGC
NM_026444	*Cs*	GGACAATTTTCCAACCAATCTG	TCGGTTCATTCCCTCTGCATA

		C	
NM_145953	*Cth*	TTCCTGCCTAGTTTCCAGCAT	GGAAGTCCTGCTTAAATGTGGTG
NM_001168571	*Ctps2*	GGCATTGGTAAAGGGATTATCGC	AAGGTGAGAAAGTTCCAGCATC
NM_028057	*Cyb5r1*	CTGATCCGGGCCATCTTGAAA	CTAGGTCCTCCCGCAGGATTA
NM_029787	*Cyb5r3*	CAGGGCTTCGTGAATGAGGAG	TCCACACATCAGTATCAGCGG
NM_025567	*Cyc1*	CAGCTTCCATTGCGGACAC	GGCACTCACGGCAGAATGAA
NM_019779	*Cyp11a1*	AGGTCCTTCAATGAGATCCCTT	TCCCTGTAAATGGGGCCATAC
NM_001033229	*Cyp11b1*	CAGATTGTGTTTGTGACGTTGC	CGGTTGAAGTACCATTCTGGC
NM_007809	*Cyp17a1*	GCCCAAGTCAAAGACACCTAAT	GTACCCAGGCGAAGAGAATAGA
NM_007810	*Cyp19a1*	ATGTTCTTGGAAATGCTGAACCC	AGGACCTGGTATTGAAGACGAG
NM_009993	*Cyp1a2*	AGTACATCTCCTTAGCCCCAG	GGTCCGGGTGGATTCTTCAG
NM_009994	*Cyp1B1*	CACCAGCCTTAGTGCAGACAG	GAGGACCACGGTTTCCGTTG
NM_024264	*Cyp27a1*	CCAGGCACAGGAGAGTACG	GGGCAAGTGCAGCACATAG
NM_010009	*Cyp27b1*	TCCTGGCTGAACTCTTCTGC	GGCAACGTAAACTGTGCGAA
NM_021282	*Cyp2e1*	CGTTGCCTTGCTTGTCTGGA	AAGAAAGGAATTGGGAAAGGTCC
NM_027816	*Cyp2u1*	TCCAAGGGTTCACCATTCCC	CGATGAGGACAGAAGTCGTCT
NM_007824	*Cyp7a1*	GGGATTGCTGTGGTAGTGAGC	GGTATGGAATCAACCCGTTGTC
NM_007825	*Cyp7b1*	GGAGCCACGACCCTAGATG	TGCCAAGATAAGGAAGCCAAC
NM_010018	*Dao*	GGTGGCAAGAGGAGTGGATG	TGGGATGATGTACGGAGAGTTG
NM_138942	*Dbh*	GAGGCGGCTTCCATGTACG	TCCAGGGGGATGTGGTAGG
NM_010022	*Dbt*	AGACTGACCTGTGTTCGCTAT	GAGTGACGTGGCTGACTGTA
NM_007832	*Dck*	TCTCCACGGTCTGCCCAAT	CAGAACCTCTTAGGTGGGGTG
NM_010024	*Dct*	TTCTGCTGGGTTGTCTGGG	CACAGATGTTGGTTGCCTCG
NM_001161516	*Dctd*	ATGGCCCGAGTATTTCATGGC	CATCACTGCACCCATTTGGC
NM_026428	*Dcxr*	ACTGTGCTGGCGTTGAAGG	CGGGTCCCACATTGCTTAGG
NM_001190448	*Ddc*	TAGCTGACTATCTGGATGGCAT	GTCCTCGTATGTTTCTGGCTC
NM_027442	*Ddo*	AAAGGGGAGTGGAGGTCTG	GGGTCACCTACAAGTCGCC
NM_007853	*Degs1*	GAATGGGTCTACACGGACCAG	CGAGAAGCATCATGGCTACAA
NM_001171002	*Degs2*	AGCGACTTCGAGTGGGTCTA	TCCCCGTACTAACCAGCAGG
NM_172733	*Dera*	TCGCAGAAGCGTGAAGAAGG	CTGCCCGGATTGGATATTTGG
NM_010046	*Dgat1*	TCCGTCCAGGGTGGTAGTG	TGAACAAAGAATCTTGCAGACGA
NM_016811	*Dgka*	GATGAACAGATTTTGCCAGGGA	GTAGCAGTACACATCACTGAGAC
NM_178681	*Dgkb*	CCGCTTCTAGTTTTTGTCAACC	AGACTGTAAACCTGACGAGGA

		C	TT
NM_177646	*Dgkd*	CCAGCCACCTGGGTACATTC	CAGGGTGCAGGGAAAAGGAG
NM_019505	*Dgke*	TGGTCCTATGGACGCTGTG	CTGAACAGGTCGGTGTCACG
NM_138650	*Dgkg*	ATGAGCGAAGAACAATGGGTC	GGGCTTGTGTGGGTCATACTG
NM_001081336	*Dgkh*	ACAGCTTTACGATCATCACTCC G	GGGCCACCTCATAGGGTTCT
NM_001081206	*Dgki*	ACTGGAGCGAGAATGCTGTG	TCCTCCTGAGAGCGGATTTTG
NM_199011	*Dgkq*	AGGTTTGAGAAGCCACGCATA	GACACGGAAGTAGGAGCCCT
NM_138306	*Dgkz*	CTCTTTGGGCACAGGAAAGC	TGCTGACTCACTCCAGTCCA
NM_001162521	*Dguok*	CGTCTCCGAGCGTCTTTCC	CGATGTTGCCTTCAATGCAGA G
NM_053272	*Dhcr24*	CTCTGGGTGCGAGTGAAGG	TTCCCGGACCTGTTTCTGGAT
NM_027903	*Dhdh*	AATTGCCAACGACTTTACCACC	TTGAATTTCTGTGCGAACTCCT
NM_020046	*Dhodh*	TCTTCACCTCTTACCTGACAGC	CATGTTGGAGTCCTGAAACGT A
NM_145614	*Dlat*	TCCCTCCGCATCAGAAGGTT	CCAACTGGAACATCTCTGGTC
NM_007861	*Dld*	GAGCTGGAGTCGTGTGTACC	CCTATCACTGTCACGTCAGCC
NM_030225	*Dlst*	GGAACTGCCCTCTAGGGAGA	GACGCTACCACTGTTAATGACC
NM_028772	*Dmgdh*	ATGGAAGGACAGAGCAGAGA C	AGCCTCTCGTAAAGTTTGATGC
NM_001199433	*Dnmt1*	AAGAATGGTGTTGTCTACCGA C	CATCCAGGTTGCTCCCCTTG
NM_007872	*Dnmt3a*	GAGGGAACTGAGACCCCAC	CTGGAAGGTGAGTCTTGGCA
NM_001122997	*Dnmt3b*	AGCGGGTATGAGGAGTGCAT	GGGAGCATCCTTCGTGTCTG
NM_001081695	*Dnmt3l*	GCTCTAAGACCCTTGAAACCTT G	GTCGGTTCACTTTGACTTCGTA
NM_170778	*Dpyd*	GACATCGAGAGTATCCTGGCT	AGGTAAAGCAGTTCTTGTCCG
NM_001164466	*Dpys*	CCACAGGGACGACTTCTCATC	CGCTGCATCTAGGATTCGCA
NM_001159646	*Dut*	GGCTACGACCTATTCAGTGCC	CTGTCTTCACGATGGCTTTCT
NM_007898	*Ebp*	ATGACCACCAATACGGTCCC	GCCAACCAGGATATGCGAAGT
NM_053119	*Echs1*	AGCCTGTAGCTCACTGTTGTC	ATGTACTGAAAGTTAGCACCC G
NM_010023	*Eci1*	GCAGGGGTTGCAGTGATGAA	GATGACACCTCGGATGCTCTT
NM_023737	*Ehhadh*	ATGGCTGAGTATCTGAGGCTG	GGTCCAAACTAGCTTTCTGGA G
NM_001109686	*Ehmt1*	CAGATGGAGAAACAAATGGGT CT	TTTGCTTCCCCACTTCTGTGT
NM_019423	*Elovl2*	CCTGCTCTCGATATGGCTGG	AAGAAGTGTGATTGCGAGGTT AT
NM_134255	*Elovl5*	ATGGAACATTTCGATGCGTCA	GTCCCAGCCATACAATGAGTA AG
NM_130450	*Elovl6*	GAAAAGCAGTTCAACGAGAAC G	AGATGCCGACCACCAAAGATA

NM_023119	*Eno1*	TGCGTCCACTGGCATCTAC	CAGAGCAGGCGCAATAGTTTTA
NM_013509	*Eno2*	GTCCCTGGCCGTGTGTAAG	CATCCCGAAAGCTCTCAGC
NM_007933	*Eno3*	CACAGCCAAGGGTCGATTCC	CCCAGGTATCGTGCTTTGTCT
NM_026421	*Enoph1*	ACCACAACCCCGATTGCTTT	TTCCTCGGCCTGTTTCCTCA
NM_008813	*Enpp1*	CTGGTTTTGTCAGTATGTGTGCT	CTCACCGCACCTGAATTTGTT
NM_015744	*Enpp2*	TTTGCACTATGCCAACAATCGG	GGAGGCACTTTAGTCCTGTACTT
NM_134005	*Enpp3*	CAGAGGAGCCCATTAAGAAAGAC	GTGCGATGAGTCAAAGCATTTT
NM_177304	*Enpp6*	CAGAGAGATTGTGAACAGAGGC	CCGATCATCTGGTGGACCT
NM_001030291	*Enpp7*	GACAGCATAAGCTACTCCTCGT	ACTGGGCTTTTACCCCTTCCT
NM_009848	*Entpd1*	AAGGTGAAGAGATTTTGCTCCAA	TTTGTTCTGGGTCAGTCCCAC
NM_009849	*Entpd2*	TGCGCCTACTCAACCTGAC	CCCAGCCATACTTGATGAAGTT
NM_178676	*Entpd3*	TTGTGAGCATTGTGGTACTTGT	GGCCACTGATACACGTAGACAG
NM_026174	*Entpd4*	ATGGGGAGGATTGGCATTTCC	GGCCTCAACGTCTGTGACTC
NM_001026214	*Entpd5*	TTGGCAGCACTGTCTTCTACA	GCCCGCATCAAACATAATTCC
NM_172117	*Entpd6*	GTTCAAGGCATCACCCTTCCT	CCATCTGGTAGGTAGACACACA
NM_007940	*Ephx2*	GCGTTCGACCTTGACGGAG	TGTAGCTTTCATCCATGAGTGGT
NM_029250	*Etnk1*	CTGTTCACAGATGGGATCACAA	CGCGGAAACTTTTCACTTCCTC
NM_175443	*Etnk2*	TTCAGCGTCACGGTGGAAC	GTGATGCCGTCTTTGAAGCG
NM_146094	*Fads1*	CAGGTTGCAGAAAAGCACAGA	CAGACTGGTAGTGCCACCTC
NM_019699	*Fads2*	AAGGGAGGTAACCAGGGAGAG	CCGCTGGGACCATTTGGTAA
NM_010176	*Fah*	GCCGAGGACTCCGACTTTC	CAGGTCCGGTAAAGAGGTGTT
NM_019395	*Fbp1*	CACCGCGATCAAAGCCATCT	AGGTAGCGTAGGACGACTTCA
NM_007994	*Fbp2*	ACCCTGACCCGTTACGTTATG	ACATTCACGCTCCCCGAAATC
NM_010191	*Fdft1*	ATGGAGTTCGTCAAGTGTCTAGG	CGTGCCGTATGTCCCCATC
NM_010210	*Fhit*	TCCTTCGCCCTGGTGAATAG	CCACTTCATCAGGATGTAGGTCA
NM_029330	*Fpgt*	TTTCTGAGCTGAGAGGCAAAC	ACAACTGTTGCTTGTAAGCGAG
NM_080845	*Ftcd*	ATGTGTCCCCAACTTCTCAGA	GAAAGTGTAGACAGTGCGGTT
NM_181666	*Fuk*	CTGACATGCCAGTACAAGGAC	GGGCACTCAAGTGTTCAGC
NM_021331	*G6Pc2*	CAGGAGGACTACCGGACTTAC	TCAACTGAAACCAAAGTGGGA

			A
NM_001159324	*Gaa*	GGGCCTGCACCCTTATCTC	GAGGTCGGTACGTCTTCCAC
NM_008077	*Gad1*	CACAGGTCACCCTCGATTTTT	ACCATCCAACGATCTCTCTCAT C
NM_008078	*Gad2*	TCCGGCTTTTGGTCCTTCG	ATGCCGCCCGTGAACTTTT
NM_008079	*Galc*	CGCCTACGTGCTAGACGAC	ACGATAGGGCTCTGGGTAATT T
NM_178389	*Gale*	CAGCCACACGGTATTGGAG	GTGCTTCTTAAAGAGGTGCTG T
NM_016905	*Galk1*	GCTCAGCGTCTCTGGAAGTG	GCGATGAGTTGGTCCATGATG
NM_175154	*Galk2*	AAGCAGGTATGTGAAGATGCG	GCTCCAGTAAGTCGTGAGCC
NM_176963	*Galm*	CTGCACGATCACTGCTCTG	CACTGCTCCAAAGTAGGGCTG
NM_016658	*Galt*	CTGAGCGTGATCTCGCCTC	GTACACGGTCCAACCATGAAC
NM_010255	*Gamt*	CACGCACCTGCAAATCCTG	TACCGAAGCCCACTTCCAAGA
NM_172672	*Ganc*	GCAGGACTGTAGGCATTTTCT	AGTTCGGCACCTGACCTTTG
NM_008084	*Gapdh*	AGGTCGGTGTGAACGGATTTG	TGTAGACCATGTAGTTGAGGT CA
NM_010256	*Gart*	TCCTCAGGTCAAGCAAGTGTT	TGGTCCGACAACTACGAGTTC
NM_025961	*Gatm*	GCTTCCTCCCGAAATTCCTGT	CCTCTAAAGGGTCCCATTCGT
NM_008094	*Gba*	GCCAGGCTCATCGGATTCTTC	CACGGGGTCAAGAGAGTCAC
NM_028803	*Gbe1*	TTCTGACGCAGCGGAGTATG	GCCACTCGGCTTGGAATGT
NM_013847	*Gcat*	GGACAGCGAACTGGAAGGG	AGTTATTGGCACAGAAGTTGA GG
NM_008097	*Gcdh*	CAGATCCGACCGGCCAAATC	CGGAAGGTGTCCCTTATCAGTT T
NM_010266	*Gda*	TCTGATGAGTGAACTTGGCAA C	CAATGTGCAATGGATGCTCCT
NM_013528	*Gfpt1*	GAAGCCAACGCCTGCAAAATC	CCAACGGGTATGAGCTATTCC
NM_013529	*Gfpt2*	ATGTGCGGAATCTTTGCCTAC	GTCATAGCCCCTGTACTCCAG
NM_008116	*Ggt1*	TTTGTCATCATCGGCCTCTGT	CCCGTCCAATCTCTGAGCAG
NM_011820	*Ggt5*	TTCAATGGGACAGAAACCTTG AG	TCCCTGTGTATAAGACCTCCG
NM_027819	*Ggt6*	TATCACAAGCTACAACTCTGG GA	GGGGGTATTTTGGAGTCTCCT TA
NM_144786	*Ggt7*	TAAGGACGAGGACGCCTTCTT	GCCCATCTCCGATGACGAC
NM_010294	*Gk2*	GCCTCGAAGCAAACCTCTG	TGTGTCAGTTCCACCTGATGA
NM_013463	*Gla*	TCTGTGAGCTTGCGCTTTGT	GCAGTCAAGGTTGCACATGAA A
NM_009752	*Glb1*	GCACGGCATCTATAATGTCACC	GTATCGGAATGGCTGTCCATC
NM_138595	*Gldc*	CTCCTGCCCAGACACGATG	GGACCGTCTTCTCGATGAGC
NM_025374	*Glo1*	GATTTGGTCACATTGGGATTG C	TCCTTTCATTTTCCCGTCATCAG
NM_001081081	*Gls*	CTACAGGATTGCGAACATCTG	ACACCATCTGACGTTGTCTGA

		AT	
NM_001033264	*Gls2*	CGTCCGGTACTACCTCGGT	TGTCCCTCTGCAATAGTGTAGAA
NM_008133	*Glud1*	CCCAACTTCTTCAAGATGGTGG	AGAGGCTCAACACATGGTTGC
NM_008131	*Glul*	TGAACAAAGGCATCAAGCAAATG	CAGTCCAGGGTACGGGTCTT
NM_001039586	*Glyctk*	CTTGCCCTGTCTCCTCCGA	TAGGTAGAGGTTTTCCCGCAG
NM_146041	*Gmds*	GCTCAAGCTCCCGCTAAGTG	GCCAAGTACGAACCATCCTGT
NM_133708	*Gmppa*	AGAAGGGGACTCGCTTCAG	CCAATGAGAAGAATCTCCTGCAT
NM_177910	*Gmppb*	ATGAAGGCACTGATCTTGGTG	GCAAGCGCCTCCACTTGAT
NM_025508	*Gmpr*	CGCATAGACGCGGACCTTAAA	AGTAGGTTTGCTTCGAGTTTCG
NM_177992	*Gmpr2*	AGTCTCGAAGTGAGGTAGAGC	GCCCACGGTATCCATATTGGC
NM_001033300	*Gmps*	GATGCAGTGGGAACTTTACTGT	AGCACGATTTAGCAAAGCTGT
NM_001190414	*Gne*	GGGAACAACCGAAAGCTCC	CCACGTCCAACTCAAAGAACG
NM_010321	*Gnmt*	AAGAGGGCTTCAGCGTGATG	CTGGCAAGTGAGCAAAACTGT
NM_010322	*Gnpat*	ATGGACGTTCCTAGCTCCTCC	GCGGGGTGTAGCACTTCATT
NM_011937	*Gnpda1*	GAGTGGGCGGCCAAGTATATT	ACTCGTCCATGTTGAAGGTTTTC
NM_001038015	*Gnpda2*	ATGGGCAGCCAAGTACATCTG	GGTTTCCACTCTTGTGGTATTCG
NM_019425	*Gnpnat1*	ATGAAACCCGATGAAACTCCC	GCCTCAAAACCAAGCCTTCTC
NM_010324	*Got1*	GCGCCTCCATCAGTCTTTG	ATTCATCTGTGCGGTACGCTC
NM_010325	*Got2*	GGACCTCCAGATCCCATCCT	GGTTTTCCGTTATCATCCCGGTA
NM_008149	*Gpam*	ACAGTTGGCACAATAGACGTTT	CCTTCCATTTCAGTGTTGCAGA
NM_001081089	*Gpat2*	CACTGCTCCGAGGTTTTGATG	AGGTTGGCAGCAATTCCATAC
NM_010271	*Gpd1*	ATGGCTGGCAAGAAAGTCTG	CGTGCTGAGTGTTGATGATCT
NM_001145820	*Gpd2*	GAAGGGGACTATTCTTGTGGGT	GGATGTCAAATTCGGGTGTGT
NM_182805	*Gpt*	TCCAGGCTTCAAGGAATGGAC	CAAGGCACGTTGCACGATG
NM_173866	*Gpt2*	AACCATTCACTGAGGTAATCCGA	GGGCTGTTTAGTAGGTTTGGGTA
NM_008160	*Gpx1*	AGTCCACCGTGTATGCCTTCT	GAGACGCGACATTCTCAATGA
NM_030677	*Gpx2*	GCCTCAAGTATGTCCGACCTG	GGAGAACGGGTCATCATAAGGG
NM_008161	*Gpx3*	CCTTTTAAGCAGTATGCAGGCA	CAAGCCAAATGGCCCAAGTT
NM_008162	*Gpx4*	GATGGAGCCCATTCCTGAACC	CCCTGTACTTATCCAGGCAGA
NM_010343	*Gpx5*	TCTAGCCAGCTATGTGCAGAC	TCCTTCCCATTAAGAGACAGAGC

NM_024198	*Gpx7*	TCCGAGCAGGACTTCTACGAC	TCTCCCTGTTGGTGTCTGGTT
NM_080289	*Grhpr*	GTGTCCTGACAGATGCCACTG	GCACATCCATAATGGGCTCC
NM_001252555	*Gstz1*	CCGGGAAGCCTATCCTCTACT	ATTCCTCAGTGAACTGTTGCC
NM_001033322	*Gucy1a2*	CCTTGCCTCAGACGATACACA	GCAGTCCGAGTACATTAGCAGT
NM_021896	*Gucy1a*	CCCCTGGTCAGGTTCCTAAG	GGAGACTCCCTTCTGCATTCT
NM_017469	*Gucy1b3*	TGCTGGTGATCCGCAATTATG	GGTTGAGGACTTTGCTTGCAG
NM_001127318	*Gucy2c*	GACTTTTCTCTCGCTTACTTGGA	TGAATGCACGAGCAAACTTGG
NM_001130693	*Gucy2d*	CTCTGTGGTATTTTGGAGTGTCC	GTGAGGCATCCTGATTAACTTGA
NM_001007576	*Gucy2f*	GGACCCTGGCCTTTTTCTCG	CCGATCTTGTAGGGGAGTGC
NM_008193	*Guk1*	AGTGTGTCCCATACTACAAGGAA	CCCTGCTGCAATATCACGC
NM_010368	*Gusb*	GGCTGGTGACCTACTGGATTT	GGCACTGGGAACCTGAAGT
NM_030678	*Gys1*	GAACGCAGTGCTTTTCGAGG	CCAGATAGTAGTTGTCACCCCAT
NM_145572	*Gys2*	ACCAAGGCCAAAACGACAG	GGGCTCACATTGTTCTACTTGA
NM_173371	*H6Pd*	ATGAAGCACACAGGCATTTGG	TCCAGGTATAGCTGAAACAGTCC
NM_025325	*Haao*	GAACGCCGTGTGAGAGTGAA	CCAACGAACATGATTTTGAGCTG
NM_008212	*Hadh*	TCAAGCATGTGACCGTCATCG	TGGATTTTGCCAGGATGTCTTC
NM_178878	*Hadha*	TGCATTTGCCGCAGCTTTAC	GTTGGCCCAGATTTCGTTCA
NM_145558	*Hadhb*	ACTACATCAAAATGGGCTCTCAG	AGCAGAAATGGAATGCGGACC
NM_001159626	*Hagh*	CCTGCCCTGACTGACAACTAC	GCAGCTTCTATAACCTTCTGTGG
NM_026897	*Haghl*	CCCGAGACGAAGGTGTTCTG	CGGACTGCATCCTCTGTCAC
NM_010401	*Hal*	CTGTGCGACGCTACATGAAGA	TCATTGTCCTCTAAGGCCACC
NM_010403	*Hao1*	CCTCGACTGGTCTGCATCAG	ATCCGTGGATAGAGCTTCCAT
NM_019545	*Hao2*	GCAGACTTTAAGGCACAAGCA	TGCCAAGTTGTCATTGTAGGTT
NM_008230	*Hdc*	CGTGAATACTACCGAGCTAGAGG	ACTCGTTCAATGTCCCCAAAG
NM_133984	*Hemk1*	TCTGGGGACAAATACTTTGGACC	CCACGATGTACTCACTGGATTC
NM_010421	*Hexa*	TGGCCCCAGTACATCCAAAC	GGTTACGGTAGCGTCGAAAGG
NM_010422	*Hexb*	CTGGTGTCGCTAGTGTCGC	CAGGGCCATGATGTCTCTTG
NM_013547	*Hgd*	ATTTGGGAATGAGTGTGCTTCA	CTCAGCGTAGAGGTTGTAGGG
NM_145567	*Hibadh*	GCAGCGGTGTGTTCTAGGTC	ACACGTCATAGAGGATGAGTGG
NM_146108	*Hibch*	GTGGAGGCGTCATAACGCTC	AGGAATGTGTCAGGGTCTTGT

NM_001146100	*Hk1*	AGGGCGCATTACTCCAGAG	CCCTGTGGGTGTCTTGTGTG
NM_013820	*Hk2*	TGATCGCCTGCTTATTCACGG	AACCGCCTAGAAATCTCCAGA
NM_001206392	*Hk3*	TGCTGCCCACATACGTGAG	GCCTGTCAGTGTTACCCACAA
NM_008254	*Hmgcl*	CAGGTGAAGATCGTGGAAGTC	GGAGCCCTGCTTCGGAAAC
NM_008255	*Hmgcr*	AGCTTGCCCGAATTGTATGTG	TCTGTTGTGAACCATGTGACTT C
NM_145942	*Hmgcs1*	AACTGGTGCAGAAATCTCTAG C	GGTTGAATAGCTCAGAACTAG CC
NM_145942	*Hmgcs1*	AACTGGTGCAGAAATCTCTAG C	GGTTGAATAGCTCAGAACTAG CC
NM_008256	*Hmgcs2*	GAAGAGAGCGATGCAGGAAA C	GTCCACATATTGGGCTGGAAA
NM_080462	*Hnmt*	TCCGGGCATAATAGCAAGGAT	TTCAGCACTTGGCTCAACAAC
NM_008277	*Hpd*	TCGGTGACCTTCTGGGTTG	CAAAGCCCATCTTGTTGCAGTA
NM_019455	*Hpgds*	GGAAGAGCCGAAATTATTCGC T	ACCACTGCATCAGCTTGACAT
NM_008288	*Hsd11b1*	CAGAAATGCTCCAGGGAAAGA A	GCAGTCAATACCACATGGGC
NM_008289	*Hsd11b2*	GGTTGTGACACTGGTTTTGGC	AGAACACGGCTGATGTCCTCT
NM_010475	*Hsd17b1*	ACTTGGCTGTTCGCCTAGC	GAGGGCATCCTTGAGTCCTG
NM_016763	*Hsd17b10*	GCTTGGTCGCGGTAGTAACTG	TGGGGCAAATATGCAGCTTTC
NM_019657	*Hsd17b12*	GGCTTCCTGTACTGGGTGG	CACGTTTTGCTAACTCTTCTGC
NM_008290	*Hsd17b2*	ATGAGCCCGTTTGCCTCTG	CCACAGGTAACAAGTCTTGGT C
NM_008291	*Hsd17b3*	AGGTTCTCGCAGCACCTTTTT	CATCGCCTGCTCCGGTAATC
NM_013786	*Hsd17b6*	GGAGCGTGTTGGAGACAGAG	GAGGTTCACTTGAAAGATAGG CA
NM_010476	*Hsd17b7*	TCTCTGCCATGTGGATAACCC	GGTCGGTAGCGTATTTGGAAG
NM_010476	*Hsd17b7*	TCTCTGCCATGTGGATAACCC	GGTCGGTAGCGTATTTGGAAG
NM_008293	*Hsd3b1*	TGGACAAAGTATTCCGACCAG A	GGCACACTTGCTTGAACACAG
NM_153193	*Hsd3b2*	GGTTTTTGGGGCAGAGGATCA	GGTACTGGGTGTCAAGAATGT CT
NM_026601	*Hyi*	TTTTCGGCCAACGTCTCCTG	CCCCTTCTCATGGTCTCCC
NM_172015	*Iars*	TCTGATGCTCAGGCTCTAGTT	ATGCGATTGATGACTTCCCGA
NM_198653	*Iars2*	CGCCAGGGCAGGACATTTT	TTCCCTCCAGGTACAAGTCTG
NM_010497	*Idh1*	ATGCAAGGAGATGAAATGACA CG	GCATCACGATTCTCTATGCCTA A
NM_173011	*Idh2*	GGAGAAGCCGGTAGTGGAGA T	GGTCTGGTCACGGTTTGGAA
NM_029573	*Idh3a*	TGGGTGTCCAAGGTCTCTC	CTCCCACTGAATAGGTGCTTTG
NM_130884	*Idh3b*	TGGAGAGGTCTCGGAACATCT	AGCCTTGAACACTTCCTTGAC
NM_008323	*Idh3g*	GGTGCTGCAAAGGCAATGC	TATGCCGCCCACCATACTTAG

NM_008324	*Ido1*	GCTTTGCTCTACCACATCCAC	CAGGCGCTGTAACCTGTGT
NM_145949	*Ido2*	TGGGGAGATACCACATTTCTG A	TGAGGAAGTCTGAGGGCAATT T
AK014297	*Il4l1*	AACACTTGTTGGTGGAAACGA	TCCTTGCGATTAGGAGTGGTC
NM_018864	*Impa1*	TCCAGAAAGCCCGAGACTTTA	GACATCAGATCGAACGGTCCA
NM_053261	*Impa2*	AGAGGGAGAGTTGGTGCAG	GTTTCTGTCACAAGATCGGCA
NM_011829	*Impdh1*	GGCTACGTTCCCGAGGATG	GGCTGATGTCAGGTCCACTT
NM_011830	*Impdh2*	ATTAGCGGAGGCACCTCTTAC	CAGTGAAGTCGATATACCCAG GA
NM_009349	*Inmt*	GCAGAGCAGGAAATCGTAAA GT	GGGGTGTAGTCAGTGACAATG AT
NM_008384	*Inpp1*	AGCAAGGTCCTTAACGGCAAC	CCCAAGACTCTCGTGAGGGAT
NM_172971	*Inpp4a*	ACTCCATCGCTAGATCGAAAA CC	AGGCAATGCTGCTTAGAAAGA T
NM_001024617	*Inpp4b*	AGAACCTCAGATGGTGGCAAA	CCCGCTCAGACTTTCTGGTG
NM_183144	*Inpp5a*	CAGGGAGAACAGTACATGAAC AC	TTGGATACACTGCTACAGGGA
NM_008385	*Inpp5b*	GGGGAAGCTGCTATGCGTTT	GGAGCGAACCTTTTCAGACAT
NM_033134	*Inpp5e*	AGCCTCCGAACACGGAAAAG	GCTGTGGGGTGATGAGATTTG
NM_008916	*Inpp5k*	CAGCACGGAGACAGGAACAC	AGGCCACATTCCACGTCAC
NM_001122739	*Inppl1*	CAGCCTGGTATCACCGTGAC	GCCACGCTCTCGCTATCTC
NM_027184	*Ipmk*	GAAGGAAGGCGTCTCCAAGTT	CGCCAAAGTTCTATCGTTTGC
NM_199056	*Ippk*	AGAGTCCCGTTGTGACAAGGA	GCTTCATCTCGTGGGTAACATC
NM_023627	*Isyna1*	CGGCCCTCAGTCTACATTCC	ATGTCCTTTCGGATTTGCTCC
NM_025922	*Itpa*	GGAGGAGGTCATTCAGATTCT CG	CTCCCGACACTTCTGTATGGA
NM_172584	*Itpk1*	GTGCAGGAAGCGAGGGATAG	GGATGACATCGGTCAGCTTGT
NM_146125	*Itpka*	GACTCGGAGGACGATCTGCT	CTTCTGCCAGTGGCTTTTCTG
NM_001081175	*Itpkb*	CGGCGCAGGCTGAATAGTAG	ATGCCCACTTTCTGGTTCACC
NM_019826	*Ivd*	GGACGGCGAGTTTCCAGTT	CTCCTCGTTTAGCCCGTTGA
NM_001114637	*Jmjd7*	GACCTCAATGTACCTCGTGTTG	CGCATAACCATCTGGCGTCA
NM_027534	*Kdsr*	TCCAGTGGCATTGGGAAGTG	CTTCTCTTGTGCCTGCTTTATG A
NM_008439	*Khk*	ATGTGGTGGACAAATACCCAG A	CAAGCAAGGAAAGGACAGTG C
NM_133809	*Kmo*	ATGGCATCGTCTGATACTCAG G	CCCTAGCTTCGTACACATCAAC T
NM_027552	*Kynu*	GTCAAGCCTGCGTTAGTGG	GGAGGGTTTGAAATTCGGAAT CC
NM_145443	*L2Hgdh*	TAGTCATCGTTGGAGGTGGAA	TCCAGTCTGGTGAAGAGCTAA AT
NM_010679	*Lalba*	ATTTCGTTCCTTTGTTCCTGGT	TGCCTTGATAGCCATCTATGTC T

NM_024434	*Lap3*	TGGGAGTGAGAGGTCTCTGG	CGCACTTGTAAATTGTGGCAG
NM_134137	*Lars*	GAGCAGCAAGGGCAAATACTT	ACTGCAAACTCACACTTGGATA A
NM_153168	*Lars2*	CATAGAGAGGAATTTGCACCC TG	GCCAGTCCTGCTTCATAGAGTT T
NM_008490	*Lcat*	GTAACCACACACGGCCTGTC	TCTTACGGTAGCACATCCAGTT
NM_001177968	*Lclat1*	TGACATCACGGTCGCATATCC	TGTACTCTGCCCAGTAAAGTG G
NM_025304	*Lcmt1*	TGTGCTGGTTTACATGACTCC	CCAGGTCACAACTTCGCCT
NM_177846	*Lcmt2*	CAGGCTCCGACTCGCTTTATT	GTCCCCGATCTTGAAAGGACC
NM_001081078	*Lct*	CTGTCATGGGCACAACTTCTC	TGTGGCATAATCAGCAAAGAG G
NM_001136069	*Ldha*	TGTCTCCAGCAAAGACTACTGT	GACTGTACTTGACAATGTTGG GA
NM_175349	*Ldhal6b*	GGGCTACAAGCATCTTGAGAG	GACACGTTGCACCTGACTG
NM_008492	*Ldhb*	CATTGCGTCCGTTGCAGATG	GGAGGAACAAGCTCCCGTG
NM_013580	*Ldhc*	TTCACGGTGTAAGATTACTGTG G	TCGTATCAGCGTCAACAAGGG
NM_027570	*Ldhd*	CTGAAGGCAGTTGTAGGGAGC	GGAACACCTTGATTGTAGCAC AG
NM_001252659	*Ldlr*	TGACTCAGACGAACAAGGCTG	ATCTAGGCAATCTCGGTCTCC
NM_145554	*Ldlrap1*	CCTCAAGTACCTTGGTATGACG C	GAGGCTGTCGGTCAGGATG
NM_029609	*Lhpp*	GACATCTCCGGGGTGCTATG	CTTTCAGCGGGGACTGTTTCA
NM_001111100	*Lipa*	TGTTCGTTTTCACCATTGGGA	CGCATGATTATCTCGGTCACA
NM_008280	*Lipc*	ATGGGAAATCCCCTCCAAATCT	GTGCTGAGGTCTGAGACGA
NM_008280	*Lipc*	ATGGGAAATCCCCTCCAAATCT	GTGCTGAGGTCTGAGACGA
NM_026334	*Lipf*	TGTGCTATCTGCATTTGGAGG	ACCCCCAGAATGTAGCCATCT
NM_010720	*Lipg*	ATGCGAAACACGGTTTTCCTG	GTAGCTGGTACTCCAGTGGG
NM_145376	*Lpcat1*	GGCTCCTGTTCGCTGCTTT	TTCACAGCTACACGGTGGAAG
NM_173014	*Lpcat2*	CCCTTCGTCCAGCAGACTAC	GCAGCAAAATTATTCCAACCA GT
NM_145130	*Lpcat3*	GACGGGGACATGGGAGAGA	GTAAAACAGAGCCAACGGGTA G
NM_207206	*Lpcat4*	CTCATCCGATACCCCAACAGT	GGGAGGAACTCTACATCCACG
NM_172266	*Lpgat1*	CTGGGCTGGATTGTAGCGAAA	GTCCTTCGATATACCAGAAGC G
NM_008509	*Lpl*	GGGAGTTTGGCTCCAGAGTTT	TGTGTCTTCAGGGGTCCTTAG
NM_008512	*Lrp1*	ACTATGGATGCCCCTAAAACTT G	GCAATCTCTTTCACCGTCACA
NM_001081088	*Lrp2*	AAAATGGAAACGGGGTGACTT	GGCTGCATACATTGGGTTTTCA
NM_013587	*Lrpap1*	CACAACCTCAACGTCATCCTG	AGCACATTGTACTCCTGGATCT T

NM_146006	*Lss*	TCGTGGGGGACCCTATAAAAC	CGTCCTCCGCTTGATAATAAGTC
NM_008517	*Lta4h*	GAGGTCGCGGATACTTGCTC	CTCCTGTGACTGGACCGTG
NM_008521	*Ltc4s*	ATGAAGGACGAAGTGGCTCTT	CCTGTAGGGAGAAGTAGGCTTG
NM_008866	*Lypla1*	TTTTCCTTCACGGATTGGGAG	GGGGACTTTTGATACCTGCAA
NM_011942	*Lypla2*	ATGTGTGGTAACACCATGTCTG	ACTCAGCCCCATCAGGTCAA
NM_173740	*Maoa*	GCCCAGTATCACAGGCCAC	CGGGCTTCCAGAACCAAGA
NM_172778	*Maob*	ATGAGCAACAAAAGCGATGTGA	TCCTAATTGTGTAAGTCCTGCCT
NM_133653	*Mat1a*	GTGCTGGATGCTCACCTCAAG	CCACCCGCTGGTAATCAACC
NM_145569	*Mat2a*	GCTTCCACGAGGCGTTCAT	AGCATCACTGATTTGGTCACAA
NM_134017	*Mat2b*	AGGGAACCTTTCACTGGTCTG	ATTTGGAGCAATCGAGCTGAG
NM_153546	*Mboat1*	AGCCTCTCTTACCGTACCACC	GGCTGGCTTTACCAGGATGTA
NM_153546	*Mboat1*	AGCCTCTCTTACCGTACCACC	GGCTGGCTTTACCAGGATGTA
NM_026037	*Mboat2*	TCAGACACGTAGTTGCTACCC	TGCAGTAGGAAATCCCACTTTG
NM_029934	*Mboat7*	TACCGCACCTACCTGGATTG	AGAAGACCGGGATCATGTAGAA
NM_023644	*Mccc1*	ACCATGAAGTATGGAACAACCC	TGCACACCCATCTTTTTGGCT
NM_030026	*Mccc2*	GCCTATCACGGGGACTCAGT	CACTCCCTCCTAGTCTCACATAC
NM_028626	*Mcee*	ATGAGGCGTGTAGTGAAGGC	TTTTCCAAATCTGGCACTGCTA
NM_008618	*Mdh1*	TTCTGGACGGTGTCCTGATG	TTTCACATTGGCTTTCAGTAGGT
NM_008617	*Mdh2*	TTGGGCAACCCCTTTCACTC	GCCTTTCACATTTGCTCTGGTC
NM_001198933	*Me1*	GTCGTGCATCTCTCACAGAAG	TGAGGGCAGTTGGTTTTATCTTT
NM_145494	*Me2*	GGCTAAGAGCTGTTACCACTCC	CGTAAACGCCATTCCCTTGTT
NM_181407	*Me3*	GGAACCCCCATCTCAACAAGG	GTTGCGGTCTTGGTGAGTCA
NM_001171003	*Mgam*	CTGACCAGTCATCTAACAAGGGC	CGTCACTCTCCATTTTGTAGCC
NM_011844	*Mgll*	CGGACTTCCAAGTTTTTGTCAGA	GCAGCCACTAGGATGGAGATG
NM_010798	*Mif*	GCCAGAGGGGTTTCTGTCG	GTTCGTGCCGCTAAAAGTCA
NM_010799	*Minpp1*	TCCCTGGTGTGATGTGTTTGA	ACCGGCTGTTAATGGTGTAGC
NM_019977	*Miox*	CCCTTCCCTGGTCTATCGAC	GTGGTAAAGACACGATCCAGC
NM_001162493	*Mpst*	TCACAGCCGCTGAAGTTACTG	CAGCATGTGGTCGTAGGGG
NM_024433	*Mtap*	ACGGCGGTGAAGATTGGAATA	ATGGCTTGCCGAATGGAGTAT
NM_138745	*Mthfd1*	GGGAATCCTGAACGGGAAACT	TGAGTGGCTTTGATCCCAATC

NM_172308	*Mthfd1l*	GCGGAGAGGATGAGATCATAGA	GTCACCCCGTCCACATCTT
NM_008638	*Mthfd2*	AGTGCGAAATGAAGCCGTTG	GACTGGCGGGATTGTCACC
NM_026788	*Mthfd2L*	AAGGACGTTGATGGATTTCACAT	GATGATTTCCCAAACGGCACT
NM_016985	*Mtmr1*	AAGAACAGCGAAAACTTGGGA	TGGCAGGATAGGTATCACAGAAC
NM_023858	*Mtmr2*	AGAACTCGGTGCATACCAAATC	GCTAACTTGTTTGCCTCCCTCA
NM_144843	*Mtmr6*	ACAGGAACACTGTATCTTACGGC	AAGTGGTCAGAGCAAGTTTCTC
NM_001040699	*Mtmr7*	GTGGAAGAATCGAGCTGACTAC	CCCCTTTTCAAAGCGGTTATACA
NM_008650	*Mut*	TTTTTGCTATCGCCCCATTACC	CCTCTGGGTTTTTGCCTTTCAG
NM_153789	*Mylip*	ATGCTGTGCTATGTGACGAGG	TCGATGATCCCTAGACGCCTG
NM_019542	*Nagk*	CACGGTCCAAAGTCCTTTTACT	GTCTGTGCCAATCAGCCAGT
NM_178053	*Nags*	CTCTAGCCAAGGCTTTGCAG	CAGCCACTCAGCGTTGGTA
NM_026086	*Nanp*	GGACAACACACTCATCGACAC	AGCACTCCTTGCTCAGTTTAAC
NM_053179	*Nans*	CAGAACCACCAAGGAGACATAGA	GGCCTTCCGGTTAAACTTGAAC
NM_019750	*Nat6*	ATTCAACCTGTCCAAGCTAACC	CCAGGACTGAGGCTCATCTCT
NM_178772	*Nceh1*	TTGAATACAGGCTAGTCCCACA	CAACGTAGGTAAACTGTTGTCCC
NM_019443	*Ndufa1*	ATGTGGTTCGAGATTCTCCCT	TGGTACTGAACACGAGCAACT
NM_024197	*Ndufa10*	ACCTTTCACTACCTGCGGATG	GTACCCAGGGGCATACTTGC
NM_027244	*Ndufa11*	GGTGAAGCGGTTCTTTGAATCT	GAGCCGATTATGCCGCAAATG
NM_010885	*Ndufa2*	TTGCGTGAGATTCGCGTTCA	ATTCGCGGATCAGAATGGGC
NM_025348	*Ndufa3*	ATGGCCGGGAGAATCTCTG	AGGGGCTAATCATGGGCATAAT
NM_010886	*Ndufa4*	TCCCAGCTTGATTCCTCTCTT	GGGTTGTTCTTTCTGTCCCAG
NM_001098789	*Ndufa4l2*	AGTCTAGGGACCCGCTTCTAC	TGTACTGGTCATTGGGACTCA
NM_026614	*Ndufa5*	ATGGCGGGCTTGCTGAAAA	GCTGCATGTTTAGGAAAGTGCTT
NM_025987	*Ndufa6*	TCGGTGAAGCCCATTTTCAGT	CTCGGACTTTATCCCGTCCTT
NM_023202	*Ndufa7*	TCCGCTACTCGCGTTATCCA	GATTGAGGGAGGCACAACTTC
NM_026703	*Ndufa8*	GGAGCTGCCAACTCTGGAAG	CCAGCGGCACAGCATAAAC
NM_025358	*Ndufa9*	GTCCGCTTTCGGGTTGTTAGA	CCTCCTTTCCCGTGAGGTA
NM_028177	*Ndufab1*	ATGGCGTCTCGTGTCCTCT	GCGGCACAAATGTGTGACT
NM_026684	*Ndufb10*	GATTCTTGGGACAAGGATGTGT	CCTTCGTCAAGTAGGTGATGGG
NM_026612	*Ndufb2*	CCCCGGTACAGGGAGTTTC	GCCAAAATCGCCAAAGAATCCA

NM_025597	*Ndufb3*	ACAGACAGTGGAAAATTGAAGGG	GCCCATGTATCTCCAAGCCT
NM_026610	*Ndufb4*	GTGTCGCACATCGTGAGTG	GAAGGCCGAAGTTGAAGGG
NM_025316	*Ndufb5*	CAAGAGACTGTTTGTCGTCAAGC	TGTTCACCAGTGTTATGCCAAT
NM_001033305	*Ndufb6*	TGGAAGAACATGGTCTTTAAGGC	TTCGAGCTAACAATGGTGTATGG
NM_025843	*Ndufb7*	CGGCGCTATCTCTGGGATG	TGGGCACAGTAGTCACGTTG
NM_026061	*Ndufb8*	TGTTGCCGGGGTCATATCCTA	AGCATCGGGTAGTCGCCATA
NM_023172	*Ndufb9*	GGTACTTTGCTTGCTTGATGAGA	TGGGAAGATATACGGCTGAGG
NM_025523	*Ndufc1*	GTAGTGCTGCGCTCGTTTTC	CCAACCAGTTAGGTTTGGCAT
NM_024220	*Ndufc2*	GGCCATGAGCCCTTAAAATTCT	CCGTGCAGTAGCCCAACAA
NM_145518	*Ndufs1*	AGGATATGTTCGCACAACTGG	TCATGGTAACAGAATCGAGGGA
NM_153064	*Ndufs2*	CAGCCAGATATTGAATGGGCA	TGTTGGTCACCGCTTTTTCCT
NM_026688	*Ndufs3*	TGGCAGCACGTAAGAAGGG	CTTGGGTAAGATTTCAGCCACAT
NM_010887	*Ndufs4*	CTGCCGTTTCCGTCTGTAGAG	TGTTATTGCGAGCAGGAACAAA
NM_001030274	*Ndufs5*	GACATACAGAAAAAGCTGGGCA	TCGCCTCATCGTTTTGTACCG
NM_010888	*Ndufs6*	ATACCGGCCAGGTAACGATG	AGGCTGTTGTGCTATCAAATCAA
NM_029272	*Ndufs7*	GTTCATCAGAGTGTAGCCACTG	CAGGCCGAAGGTCATAGGC
NM_144870	*Ndufs8*	AGTGGCGGCAACGTACAAG	TCGAAAGAGGTAACTTAGGGTCA
NM_133666	*Ndufv1*	TTTCTCGGCGGGTTGGTTC	GGTTGGTAAAGATCCGGTCTTC
NM_028388	*Ndufv2*	GCAAGGAATTTGCATAAGACAGC	TAGCCATCCATTCTGCCTTTG
NM_030087	*Ndufv3*	GAGAGGGGCAAGCTCCTAAC	ACGCTACCAAAGTCTTTCTTGAC
NM_010893	*Neu1*	GGACCGCTGAGCTATTGGG	CGGGATGCGGAAAGTGTCTA
NM_001160164	*Neu2*	CACAGGCGTCCATGCTTACA	CTGCGTGCTCATCCGTCTT
NM_016720	*Neu3*	AGCAGGAAGAACAGAGTGGG	CCTCCATCAGTAGCCGTTGG
NM_008704	*Nme1*	AGGAGCACTACACTGACCTGA	GGTTGGTCTCTCCAAGCATCA
NM_008704	*Nme1*	AGGAGCACTACACTGACCTGA	GGTTGGTCTCTCCAAGCATCA
NM_019730	*Nme3*	GAGATCGTGCGTCGCTTTGA	CGCAGCTCGACATAATGCTC
NM_019731	*Nme4*	TCCATATCTCAAGGGCCATGA	AGCTCGATCTCCCTCTGGG
NM_080637	*Nme5*	AAAACCCTAGCCCTTATCAAGC	AGGTGTAGTTTCCGTCTCTGAA
NM_018757	*Nme6*	CCTCATTGTACGAACGAGGGA	GCAAGGATATAGGCTCGGATTG

NM_178071	*Nme7*	ATGCTTTGGAATGTCAGCGAT	TGCTCTGTTGGATCTCTATTGCT
NM_008712	*Nos1*	CTGGTGAAGGAACGGGTCAG	CCGATCATTGACGGCGAGAAT
NM_010927	*Nos2*	GTTCTCAGCCCAACAATACAAGA	GTGGACGGGTCGATGTCAC
NM_008713	*Nos3*	GGCTGGGTTTAGGGCTGTG	CTGAGGGTGTCGTAGGTGATG
NM_008720	*Npc1*	TGTTTGGTATGGAGAGTGTGGA	GTCACAGCAGAGACTGACATTG
NM_023409	*Npc2*	AGGACTGCGGCTCTAAGGT	AGGCTCAGGAATAGGGAAGGG
NM_008727	*Npr1*	GCTTGTGCTCTATGCAGATCG	TCGACGAACTCCTGGTGATTTA
NM_173788	*Npr2*	TGCTGCCAGAACACAACCTG	TTCGGAGCTGACAAACCGC
NM_008739	*Nsd1*	ATTTGGGCAAAATTCAAGAGACG	GCCTCCTATTGGCAACTTTCATT
NM_010941	*Nsdhl*	TCATGGTGAATCAAAGCGAGG	CCGGGGGTTATCAAAGCCTTG
NM_015807	*Nt5c*	CGGACCTGGCGGAAAAAGT	GTTCATCTCTCGCAAAGCGTC
NM_001085502	*Nt5c1a*	ATGACAACCTCTCGTCCAAGA	AGGGCCTTCACAAAGGGGA
NM_027588	*Nt5c1b*	AGAACAGACCTATCACCCAGC	GGCATCCCTTGTCGTGAGT
NM_001164365	*Nt5c2*	CCTCCTGGAGTGACCGCTTA	AACACCCGGTGATAGGCTTCT
NM_001252374	*Nt5c3*	AAGCTCAGTTCGTATCAAGAACC	GCGCCTCCTTTAATGAGACCA
NM_011851	*Nt5e*	GGACATTTGACCTCGTCCAAT	GGGCACTCGACACTTGGTG
NM_134029	*Nt5m*	GCCTGGGCTAAGCGAGAAG	TCTGGTCAACACAATCTGCTC
NM_025539	*Nudt2*	CTGAGGGCATGTGGCTTGAT	GCAGGGCTGTTTCTAAGTCATTC
NM_016918	*Nudt5*	GGAAGAAACTGGCTACAAAGGT	TTGGCCTTACATTTCCTGCAT
NM_028794	*Nudt9*	CTCGCGTCAGTGACTGTCC	AGACATGATGTTGGCGTTAAGG
NM_016978	*Oat*	GGAGTCCACACCTCAGTCG	CCACATCCCACATATAAATGCCT
NM_177215	*Ocrl*	AGTGAGCGACCCCTTCAAGT	AGGCAATCAATGATCTGCTGTAA
NM_016967	*Olig2*	TCCCCAGAACCCGATGATCTT	CGTGGACGAGGACACAGTC
NM_001252288	*Ogdh*	AGGGCATATCAGATACGAGGG	CTGTGGATGAGATAATGTCAGCG
NM_001081130	*Ogdhl*	TCGCTTGGAGAGCACCTACT	CTCGACGGAAAACTGCATCA
NM_001199227	*Osbpl5*	TTCTGGGCTGCGAAAATGAG	GTCAGATCCATTGCATAGCCTG
NM_008769	*Otc*	AGGGTCACACTTCTGTGGTTC	CAGAGAGCCATAGCATGTACTG
NM_024188	*Oxct1*	CATAAGGGGTGTGTCTGCTACT	GCAAGGTTGCACCATTAGGAAT
NM_011030	*P4ha1*	AGCCACCATTTCAAACCCAGT	GCCAAGCACTTTTGCTAATTCTG

NM_177161	*P4ha3*	ACATGCTCAACGTGAAGGG	TGCCAACTTGGAAGCAGTCAT
NM_013625	*Pafah1b1*	CCTTGGATTTCCATAAGACGGC	ACTCCCACACCTTTACTGTTTG
NM_008775	*Pafah1b2*	GCAGCTATTCCACATGCCG	CCGCCATATCTCGTACTGCT
NM_008776	*Pafah1b3*	AACCCACTTCGAGAGAAAAACC	CCATCAGAATGCACAAACCCA
NM_133880	*Pafah2*	GGAGGGTCACAGTCTTGAGG	CCCATGCTGTACTCGTAGCG
NM_008777	*Pah*	TTGTCCTGGAGAACGGAGTC	CTGGATTCAATGTGTGTCAGGTT
NM_025939	*Paics*	ATGGCGACAGCCGTAGTAGT	CCTGGGGTGTCTAACAACTCA
NM_011863	*Papss1*	TGTGCAAGAAAGTCAAGCTGA	ACCTGCCCTCTCTTATTCCTG
NM_001201470	*Papss2*	AGGCCCATCATGTGAGCAG	CACACGGTACATCCTCGGAAT
NM_144844	*Pcca*	TTCATACCAATGCCTAGTGGTGT	GACAGCCTCATCCGCCATTTT
NM_025835	*Pccb*	GCGGCGATTAGGATTCGGG	TGCGCTCTTTAACTGAAACCG
NM_011044	*Pck1*	CTGCATAACGGTCTGGACTTC	CAGCAACTGCCCGTACTCC
NM_028994	*Pck2*	ATGGCTGCTATGTACCTCCC	GCGCCACAAAGTCTCGAAC
NM_153565	*Pcsk9*	GAGACCCAGAGGCTACAGATT	AATGTACTCCACATGGGGCAA
NM_177546	*Pcyt1b*	TTCTCACGCAAGACCCTGAC	GCAAACTCCTACCAACAAGTAGC
NM_024229	*Pcyt2*	CGATGGCTGCTATGACATGGT	GCCCCTTATGCTTGGCAATCT
NM_011866	*Pde10a*	GGACAGAGACAAGCGAGATGA	GGTGTGCTCTTGCTAGGCG
NM_001081033	*Pde11a*	AACAGGACCTACGATGAACAGG	TGAGGCAGATTCACCCTCGAT
NM_001159582	*Pde1a*	ATGGGGTCTACTGATACGGAC	TCTCAGATGGGATCAAGTTGCT
NM_008800	*Pde1b*	GATGCTGGAGTCGGATTGCC	TTCAGTGTCTAGGATTTGCCTTG
NM_001159960	*Pde1c*	CCTCCTTTGCGTCACTTTAAGC	TCATCTTGATCCGCTGATCCA
NM_001243758	*Pde2a*	TGGCGTTGTGGACGATGAG	CGCGATAGAAAAGCGGATGG
NM_018779	*Pde3a*	TCCCAGTCAGGAACCAGCAT	CAAGTTGCTTACGGCCCTC
NM_011055	*Pde3b*	AAAGCGCAGCCGGTTACTAT	CACCACTGCTTCAAGTCCCAG
NM_019798	*Pde4a*	AATGCCCTACAGACGCCTG	GACGGTGTTGGCCCATTTT
NM_019840	*Pde4b*	CAAACAAGGTAAGGACACCTCTT	TGGTAGCAAGGTACGAGCAAA
NM_201607	*Pde4c*	TCCGAGAGCCAGTGGATTCT	CCTTGAGTTCCAATCGTGAAGAC
NM_011056	*Pde4d*	TTTTGCCAGTGCAATACATGATG	CAGAGCGAGTTCCGAGTTTGT
NM_153422	*Pde5a*	CGGCCTACCTGGCATTCTG	GCAAGGTCAAGTAACACCTGATT
NM_008806	*Pde6b*	GCAGCACTTTTTGAACTGGTG	CATTGCGCTGGCGGTACATA
NM_001170959	*Pde6c*	GTGCAGAGTCAGGTAGCACTT	TCCATCATAGGAATTGGTTGCT

			G
NM_008801	*Pde6d*	CCCGTGTGCCCAAGAAAATC	CCACTCTTCTAGGCATTGTCCT T
NM_012065	*Pde6g*	AGGGTGAGATTCGGTCAGC	GCTCTTGAACTGCCTTGTTTG
NM_008802	*Pde7a*	AGTGGATCACCTCTAAGAGAC G	CGGACATCTCCTAGCATACGA AT
NM_013875	*Pde7b*	TGCTAGGAGATGTACGACTAA GG	GGGCCTGCGGTATAATCCC
NM_008803	*Pde8a*	CCGAGCATCCACACTTCCG	TCAGCTACTGATACCTTCGAGG
NM_172263	*Pde8b*	GGGAAGTTCGCTCACAGTTCA	CAAAGGCAGGGTTGACATACT
NM_008804	*Pde9a*	CCACCATCTCCCTTTTAACCAC	CAGCACGCCCTGGATAAGT
NM_008810	*Pdha1*	GAAATGTGACCTTCATCGGCT	TGATCCGCCTTTAGCTCCATC
NM_008811	*Pdha2*	CTGTCTCACGTATTTTCGGGAA	AGCCGGTACAGGTCACATTTC
NM_023523	*Pecr*	GGTCAGAGCTACCTAGCGG	GACCACGTTACACCCCAGG
NM_008819	*Pemt*	TGGCTGCTGGGTTACATGG	GCTTCCGAGTTCTCTGCTCC
NM_001159519	*Pfas*	TCTCCTTCATGGGTCTCCCC	AGGCCCTGTTTCTCCTCTATC
NM_008824	*Pfkfb1*	ATGAGCTGCCCTATCTCAAGT	GTCCCGGTGTGTGTTCACAG
NM_008825	*Pfkfb2*	GACAAGCCAACTCACAACTTCC	ACACTGTAATTTCTTGGACGCC
NM_001177758	*Pfkfb3*	CCCAGAGCCGGGTACAGAA	GGGGAGTTGGTCAGCTTCG
NM_173019	*Pfkfb4*	AGGATGTCTTGTCGCCTCTG	CGGTACTTGTCCTGATCCCG
NM_008826	*Pfkl*	GGAGGCGAGAACATCAAGCC	CGGCCTTCCCTCGTAGTGA
NM_001163487	*Pfkm*	TGTGGTCCGAGTTGGTATCTT	GCACTTCCAATCACTGTGCC
NM_019703	*Pfkp*	TGGTGCCATCATGCTATCTGA	GGTCGCACGTCTCGACAAT
NM_023418	*Pgam1*	TCTGTGCAGAAGAGAGCAATC C	CTGTCAGACCGCCATAGTGT
NM_018870	*Pgam2*	TGGAACCAAGAGAACCGTTTC	TGGCATCTTTGATAGCGGTGG
NM_008828	*Pgk1*	ATGTCGCTTTCCAACAAGCTG	GCTCCATTGTCCAAGCAGAAT
NM_031190	*Pgk2*	TTCTGCTAAGTTGACTCTGGAC A	AGCCTTGATTCTCTGGTTGTTT G
NM_025396	*Pgls*	CCAGGTCCTTACCATCAATCCT	AGGGAAGAGCGAACAGGTAT G
NM_025700	*Pgm1*	CAGAACCCTTTAACCTCTGAGT C	CGAGAAATCCCTGCTCCCATA G
NM_028132	*Pgm2*	AGTGAAGACGCAGGCATATCC	GGCTCCACGGTAGAGACGA
NM_027629	*Pgm2l1*	ATGACCTGAACTCTAACCTGCT	CCCATTCCGCAAGAGATTTTCA A
NM_028352	*Pgm3*	AGCAGTGGGATGCTATTTATG TC	TGTCTGCGCTTTCTTGTGAGT
NM_025954	*Pgp*	GCTTCGACCCACACTTCAG	AGCCGGTTGTCCATGTTGG
NM_133757	*Pgs1*	GTTCCTCAGGTCACCTCCC	CCATCACAACTCGCCTCTTGG
NM_016966	*Phgdh*	ATGGCCTTCGCAAATCTGC	AGTTCAGCTATCAGCTCCTCC
NM_153104	*Phospho1*	ATGAGCGGGTGTTTTCCAG	ATCGAAGTCGAAGGTGAGGA G

NM_029293	*Phpt1*	TGTAGACATTGATTCGGATGGC	GCACGATTTCCTTGCACTCC
NM_001001983	*Pi4ka*	CTCAGCCATGTTTTATGCTGGG	GGGAAGGATCGCAGGACTTTTA
NM_175356	*Pi4Kb*	ATGGGAGACATGGTAGTGGAG	TGATGACGCTTAGTAGGGAGC
NM_011083	*Pik3c2a*	TGACAGCCCAAGAGGCTTTG	CCTGGGTGAGCTTTTCTACATC
NM_001099276	*Pik3c2b*	TGACATCAACAGTTTCTCTTCGG	CTCTGAGTCGTAGGTGGACTT
NM_011084	*Pik3c2g*	CCATCTATCAGCTAATCGACGTG	GGAACACACTGTGAAGCTCAG
NM_181414	*Pik3c3*	CCTGGACATCAACGTGCAG	TGTCTCTTGGTATAGCCCAGAAA
NM_008839	*Pik3ca*	CCACGACCATCTTCGGGTG	ACGGAGGCATTCTAAAGTCACTA
NM_029094	*Pik3cb*	CTATGGCAGACAACCTTGACAT	CTTCCCGAGGTACTTCCAACT
NM_001164052	*Pik3cd*	GTAAACGACTTCCGCACTAAGA	GCTGACACGCAATAAGCCG
NM_020272	*Pik3cg*	CGAGAGTGTCGTCACAGTGTC	TGTTCGCTTCCACAAACACAG
NM_011086	*Pikfyve*	TCCCCGACACTGGACTCTG	GGCTGGCCCAACTTGAACT
NM_008845	*Pip4k2a*	CTGGCGAGCAAGACCAAGA	GGCTCAGTTCATTGATCGAATGG
NM_054051	*Pip4k2b*	AGCGCCAGCAAAACCAAGA	CATCCGGCATTAGCATGACAG
NM_054097	*Pip4k2c*	GCGTCGCTCACTCGATCAAT	TGCCTTCAGTTTCGCTTGGG
NM_008847	*Pip5k1a*	ACCTGAAGGGTTCAACTTACAAG	ACAGTCACGCTGTAGAGTCTT
NM_008846	*Pip5k1b*	TGCCAAAATTCTATGGGCTGT	GGATGCTCTTCGCTTGTATGT
NM_001146687	*Pip5k1c*	GGATGCGTCGGGAGAGACTA	AGGTCTTGAAGCGGAAATCCT
NM_008952	*Pipox*	TTCTGGGATGCTATTGTGATTGG	CTCTGTCCATGTGAGCTTCCT
NM_177298	*Pisd*	TCTGGGGACCTTACAGAAATTGC	GGCACAGATTTATACAGGGACAC
NM_001099779	*Pklr*	TCAAGGCAGGGATGAACATTG	CACGGGTCTGTAGCTGAGTG
NM_011987	*Pla2g10*	GTGCAGGTGTGACGAGGAG	CACTTGGGAGAGTCCTTCTCA
NM_183423	*Pla2g12a*	TGCCTTTCCTGTAGGCTGTTC	CGCAGGTCTCGTAGCATCTG
NM_023530	*Pla2g12b*	GAACCTGGCTCAGAGTGACC	GCAGCTCCATGAAGGAATCCA
NM_133792	*Pla2g15*	GGGTAACCAGTTGGAAGCAAA	TTGTCAATCCAGCAGTCAATGAT
NM_011107	*Pla2G1b*	GTGTGGCAGTTCCGCAATATG	CCTGTCTAAGTCGTCCACTGG
NM_001082531	*Pla2g2a*	TGGCTCAATACAGGACCAAGG	GTGGCATCCATAGAAGGCATAG
NM_011109	*Pla2g2d*	TGCTGGCCGGTATAACTGC	CTGTGGCATCTTTGGGTTGC
NM_012044	*Pla2g2e*	CCAGTGGACGAGACGGATTG	AGCAGCTCTCTTGTCACACTC

NM_012045	*Pla2g2f*	ACTGGACGGAAGAGCCCAA	GGATGGAGTTTCTGTGTGTGAT
NM_172791	*Pla2g3*	AGAGACCACAGGGCCATTAAG	GCTGTAGAATGACATGGTGCT
NM_008869	*Pla2g4a*	CAGCACATTATAGTGGAACACCA	AGTGTCCAGCATATCGCCAAA
NM_145378	*Pla2g4b*	TGGCCCCTAGCCAACTTTG	GTTCTGGCCTCGACTCAGG
NM_001122954	*Pla2g5*	CCAGGGGGCTTGCTAGAAC	AGCACCAATCAGTGCCATCC
NM_001199023	*Pla2g6*	GCAAGCTGATTACCAGGAAGG	GAGAGAAGAGGGGGTGAGTTG
NM_001145830	*Plcb1*	GCCCCTGGAGATTCTGGAGT	GGGAGACTTGAGGTTCACCTTT
NM_177568	*Plcb2*	TGCTGATCGAAAACGGGTGG	AGCTTTAGAGTGGTAGGAAGTGA
NM_008874	*Plcb3*	CTGCCGCTCTATCTTTGGGG	GCCGATGTCGCTTCTTATTCTTC
NM_013829	*Plcb4*	GGACAAGTGCTAGAATGTTCCC	GAAGCCGATATTCACCAGATCC
NM_019676	*Plcd1*	CAGCTCGTGGCGTAGAGAAC	CCTGAATGTCCTCGATGGAGAA
NM_152813	*Plcd3*	GGCTACGGGCACTGAAGAAG	GCTGCACGAAGAATATGTGCTT
NM_148937	*Plcd4*	GAAGGTTATGAAGTGTCCGATGT	AACTGCTTTGACAAGAGAATGGA
NM_019588	*Plce1*	GAGTGTAAAGGTCTCAGATGCG	GGAGTCTGGTGCGTTTTCCT
NM_021280	*Plcg1*	GCGGACAAAATCGAGGGGT	TCCTATCAGGTGATCCAAGACC
NM_172285	*Plcg2*	GTGGACACCCTTCCAGAATATG	ACCTGCCGAGTCTCCATGAT
NM_054066	*Plcz1*	CTCGCAGAAGCAAGATGGTTT	GGCATGGGAAATCAAGTTTCTCA
NM_001164056	*Pld1*	TCGTTTTGTGGACTGAGAACAC	GCTGCTGTTGAAACCCAAATC
NM_008876	*Pld2*	CCTCTTTCCCTATGGGGACTATC	ACCAAGGGGTGTGCTTTCAG
NM_011122	*Plod1*	GAAGGATGACGCCAAGCTAGA	TGAAGAACTGAGCTGAACGCT
NM_011961	*Plod2*	GAGAGGCGGTGATGGAATGAA	ACTCGGTAAACAAGATGACCAGA
NM_011962	*Plod3*	TGGATGGGGAAGGGCTAGTG	GCCAACCTGGAATCCTCGTG
NM_011125	*Pltp*	CGCAAAGGGCCACTTTTACTA	GCCCCCATCATATAAGAACCAG
NM_013872	*Pmm1*	GTCCTGGCGGGAATGACTTT	TGGGCTGTCTCTGGGAAGAA
NM_016881	*Pmm2*	TGGTAGGTGGGTCAGATTTTGA	CCAAGCCATTCTCTGGAAACA
NM_026925	*Pnlip*	CTGGGAGCAGTAGCTGGAAG	AGCGGGTGTTGATCTGTGC
NM_018874	*Pnliprp1*	ATGCTGATTCTCTGGACAATCC	GAAGTTTTAGGGGCCTGATAG

		C	C
NM_011128	*Pnliprp2*	ATGCCTATGGATGTCCGTGGA	TGCCCAGGGCTTGTCATTG
NM_008890	*Pnmt*	CAGACCTGAAGCACGCTACAG	TAGTTGTTGCGGAGATAGGCG
NM_013632	*Pnp*	CAACACACTGAATATCGACCTCA	GCTTTGGGGAAAGTTGGGTATCT
NM_054088	*Pnpla3*	TCACCTTCGTGTGCAGTCTC	CCTGGAGCCCGTCTCTGAT
NM_027869	*Pnpt1*	CCTTCTCCATATTTGCACCAACA	TGTCGCGGTATAAACTGCTCC
NM_008892	*Pola1*	AGCTCGGCGAGAAAAGAAATC	CCACATAGCCTATCCCATCGTC
NM_008893	*Pola2*	TTCAAGCAATGCCTGAGAACA	CCTACTTGTCCTAGAGGAACCTG
NM_011131	*Pold1*	TCGCAGTTTGAGGCGAACC	CTGAGCCCACATAGTGGTCA
NM_008894	*Pold2*	TGGGAGAGCGCAGCTTTAAC	CCTTGAACAGAGTGCCCAC
NM_133692	*Pold3*	AAGATCGTGACTTACAAGTGGC	ACACCAAGTAGGTAACATGCAG
NM_027196	*Pold4*	ATCACTGACTCCTATCCTGTTGT	TACTGCCAGGCCAGGTCAA
NM_011132	*Pole*	CTCAGCACTCAAGCGTCTGG	GCCACCTTAAATCTGCTTCCAT
NM_011133	*Pole2*	TGTCACTGAATTGGAGCTAGAGG	ACTGACTGCACTCTTGCACAG
NM_021498	*Pole3*	GAGAGGCCCGAGGACCTAAAT	TGGCATACAAAACGAAGACACTG
NM_025882	*Pole4*	ACGCCCAGAGAGGAAGAGG	CCAGCGTTACGTCAGGGTC
NM_009088	*Polr1a*	GCATGGCAGGACGAGAAGG	GATCGTACTGGATGACCAGCC
NM_009086	*Polr1b*	CAGGCCATACCTCCCTTTGAA	TCCCTCGGTAGGTGCTCTTTC
NM_009085	*Polr1c*	CGTGGTTCTCGGAGAGTTTGG	CCAGCGTGTCTTCATCCATTT
NM_009087	*Polr1d*	GAAGGCGAGAGGAAGACAGC	TGGGTCGTAGTGTAACCACAAA
NM_022811	*Polr1e*	CAGGGACAAGTTGGATTCTTGC	AGAGGGCGTCATATTGTAAGTCA
NM_009089	*Polr2a*	CTGGACCTACCGGCATGTTC	GTCATCCCGCTCCCAACAC
NM_153798	*Polr2b*	GACGACGATGAGATCACTCCG	GGTGCATCTTCCACAATTCTTTG
NM_009090	*Polr2c*	CTCACCGAAGAGAACGTCAAG	TCGATGGCTATTATGGGCACC
NM_027101	*Polr2d*	GCTGGCGACGTAGAAGAAGAC	CTGCTTTCGATGCTCCAGAAG
NM_025554	*Polr2e*	GGTGGGCATCAAGACCATCAA	TCAGGGACTAGCTCGTGCTC
NM_027231	*Polr2f*	GACAACGAGGACAATTTCGACG	GGAGAATCTCGACATTTTCCTGG
NM_026329	*Polr2g*	AAGCGACCGCAGAGAAGTG	CGTGCTCCAGGGAAATGTGATA
NM_145632	*Polr2h*	CTGCACTGTGAGAGTGAATCTT	GCTATGACCAACCGGAACTTG

NM_027259	*Polr2I*	CATCTACGTCAACAAAATCACGC	GCAGTGCAAACATAGTACAGGC
NM_011293	*Polr2J*	GCCTTCGAGTCGTTCTTGCT	GCGATTTAATGATGTTCCCCAGA
NM_001039368	*Polr2k*	GCAAAACCAGGAGAGACACTT	TGAGCGTCCATTGTTAGTGCC
NM_025593	*Polr2l*	GGGAAGATCGTCGGCAACAAA	TAGGGGTGCATAGTTCAGCAG
NM_001081247	*Polr3a*	GACAGCCATTGAGCATAACAAAG	CTGGGATGCGCTTAAACAAGT
NM_027423	*Polr3b*	CCCATCAAGAATGACGGTTGG	ACATCCTTCACTTTACTCCCTCC
NM_028925	*Polr3c*	GATACACAGATAACAAGGAGCCC	CGCTCACGATTGCTTGGTC
NM_025945	*Polr3d*	AAAAGCGTGAACGGGACAGG	CCACGGTCTTATCCCAGTTCC
NM_029763	*Polr3f*	AAGCCGTGAAGTCTGTAGCAG	TGCCTTGCTTTGTAGGAATTTGA
NM_001081176	*Polr3g*	AGAGAGGCGAGGGTGAAAGAT	GCCGTTGTCGAAGTAGGAGTT
NM_027241	*Polr3gl*	TCCTCCCTTGGAGTTCCACC	CAATAGGCCCAGACATCTGATAC
NM_030229	*Polr3h*	CATACCCCCGTGGCAGTTT	CGTAGCGGAAATGGACTTTGG
NM_025901	*Polr3k*	TGCGGGAACGGACTCATTG	CGGTTTGTTACCTTACGGGTGA
NM_026438	*Ppa1*	AGTACCGCGTCTTCCTCAAAA	GACCAGCGTGGAACCTCAAC
NM_146141	*Ppa2*	GATGCTGACGCACCTCAGAAT	AGAACCTTCATTTGCGACAGG
NM_172146	*Ppat*	GCGAGGAATGTGGTGTGTTTG	TTTAGGCACTGCACTCCCATC
NM_007453	*Prdx6*	CGCCAGAGTTTGCCAAGAG	TCCGTGGGTGTTTCACCATTG
NM_008921	*Prim1*	ATGGAGCCATTTGATCCTGCG	CACTCCGCCATAGTTGAGCC
NM_008922	*Prim2*	GGCGGGTGACCAGAGAAATG	ACATAGCTCACACCGAGGTTC
NM_011172	*Prodh*	GCACCACGAGCAGTTGTTC	CTTTGTTGTGCCGGATCAGAG
NM_019546	*Prodh2*	GTGGGGCCTTCCATGTCAAG	CCTGAAACGCTAGTCCATGAGT
NM_021463	*Prps1*	ACTTATCCCAGAAAATCGCTGAC	CCACACCCACTTTGAACAATGTA
NM_029294	*Prps1l1*	CCAAACATCAAACTCTTCAGCG	ACACTCTCACCAATTTCCACG
NM_026662	*Prps2*	ATGCCTAACATCGTGCTCTTC	GATCTCGACACTGGTCTCCTG
NM_177420	*Psat1*	CAGTGGAGCGCCAGAATAGAA	CCTGTGCCCCTTCAAGGAG
NM_133900	*Psph*	AGGAAGCTCTTCTGTTCAGCG	GAGCCTCTGGACTTGATCCC
NM_008959	*Ptdss1*	GCAGGACTCTGAGCAAGGATG	GGCGAAGTACATGAGGCTGAT
NM_013782	*Ptdss2*	GGATTGCCTTTCAGTTCACGC	AGGTAGAAGGTGTTCAGCTCTG
NM_008960	*Pten*	TGGATTCGACTTAGACTTGACCT	GCGGTGTCATAATGTCTCTCAG
NM_008963	*Ptgds*	TGCAGCCCAACTTTCAACAAG	TGGTCTCACACTGGTTTTTCCT
NM_022415	*Ptges*	GGATGCGCTGAAACGTGGA	CAGGAATGAGTACACGAAGCC
NM_133783	*Ptges2*	CCTCGACTTCCACTCCCTG	TGAGGGCACTAATGATGACAG

			AG
NM_008968	*Ptgis*	ACAGCATCAAACAATTTGTCGT C	GCATCAGACCGAAGCCATATC T
NM_008969	*Ptgs1*	ATGAGTCGAAGGAGTCTCTCG	GCACGGATAGTAACAACAGGG A
NM_011198	*Ptgs2*	TGAGCAACTATTCCAAACCAG C	GCACGTAGTCTTCGATCACTAT C
NM_144795	*Pycr1*	ATGAGCGTAGGCTTCATCGG	CGAGACCGTAGCTTGGTCC
NM_133705	*Pycr2*	ATGAGCGTGGGTTTCATCGG	GCTTCGGGTCAGATTCACACC
NM_025412	*Pycrl*	ATGGCAGCGACAATGTCTGAA	CCAGCACTTGTTTAGCTTCTAC T
NM_153781	*Pygb*	CCGCGACTACTTCTTCGCTC	CAACCCCAACTGATAAGTGGC
NM_133198	*Pygl*	GAGAAGCGACGGCAGATCAG	CTTGACCAGAGTGAAGTGCAG
NM_011224	*Pygm*	CTTAGCCGGAGTGGAAAATGT	GTAATCTCTCGGAGTAGCCAC A
NM_153196	*Rbks*	GGTTCCTGCATGACCGACC	TGCCAAAAGAATCGTTGCCAA
NM_001164704	*Renbp*	GTATTGTCGCTTATACCGCAGT	AACGCAGCAAAAATTCACCAC
NM_025683	*Rpe*	GCACCTGGATGTAATGGACGG	CCTGGCCTAGCTGCTTTCG
NM_009075	*Rpia*	AAGGCCGAGGAGGCTAAGAA	CTTTCAGCTATTCGCTGCACA
NM_009103	*Rrm1*	GCCGCCAAGAGCGAGTTAT	TCATGGTGATCTGAGCAGGAT
NM_009104	*Rrm2*	TGGCTGACAAGGAGAACACG	AGGCGCTTTACTTTCCAGCTC
NM_199476	*Rrm2b*	GAGCCACTCCTAAGAAAGAGT TC	GAGGGAGGTCCTTTGACAAGT
NM_138665	*Sardh*	CGGGTCCTACGTGTAGCTG	TGATAAGGAACACTCTTCTCG GT
NM_009121	*Sat1*	GAGAACACCCCTTCTACCACT	GCCTCTGTAATCACTCATCACG A
NM_026991	*Sat2*	AGGTTGCCCTGAATAAGGGTT	CTCGCCTTCAAATCGAAAGGA
NM_016741	*Scarb1*	AAACAGGGAAGATCGAGCCA G	GGTCTGACCAAGCTATCAGGT T
NM_023281	*Sdha*	GGAACACTCCAAAAACAGACC T	CCACCACTGGGTATTGAGTAG AA
NM_023374	*Sdhb*	AATTTGCCATTTACCGATGGGA	AGCATCCAACACCATAGGTCC
NM_025321	*Sdhc*	GCTGCGTTCTTGCTGAGACA	ATCTCCTCCTTAGCTGTGGTT
NM_025848	*Sdhd*	TGGTCAGACCCGCTTATGTG	GGTCCAGTGGAGAGATGCAG
NM_145565	*Sds*	GAAGACCCCACTTCGTGACAG	TCTTGCAGAGATGCCCAATGC
NM_178029	*Setd1a*	TGCTGTCCCTCGTAGACTGG	GGCTCTTTCCGTTTTACCTTGA
BC038367	*Setd1b*	TCCTCAAGCTCCGACAAGGAT	CGTCGATGTCTGAATCAATCTG G
NM_001081340	*Setd2*	CACCAGGTTACCCCATGCAAG	TCTGTAGAATGTCCTACCAAG GG
NM_001163642	*Setdb1*	CAACTTGCACAATCACGGAAA C	CAAAGGTGACCGATATGTCTG G

NM_001081024	*Setdb2*	GGATGGAGCTACAAGATGATG G	CCAGTGTTTGCGTGTTACTCAG
NM_178391	*Setmar*	AGATAGCAGCATCTGAGGAGG	TCCAGGTCCAGCGACATGA
NM_001168525	*Sgms1*	GAAGGAAGTGGTTTACTGGTC AC	GACTCGGTACAGTGGGGGT
NM_028943	*Sgms2*	GAGACAGCAAAACTTGAAGGT CA	CCCGTTGGATAAGGTCTTGGG
NM_009163	*Sgpl1*	CTGAAGGACTTCGAGCCTTATT T	ACTCCACGCAATGAGCTGC
NM_030750	*Sgpp1*	GATGCAGAGACCGAGGTTCG	CGGCAAGTTGCTCACTTTGAC
NM_001004173	*Sgpp2*	TTCACCCACTGGAATATCGACC	AAGTCTCACAACGGGAGGAAA
NM_009171	*Shmt1*	CAGGGCTCTGTCTGATGCAC	CGTAACGCGCTCTTGTCAC
NM_001077515	*Slc1a2*	GCCAACAATATGCCCAAGCAG	GACACCAAACACAGTCAGTGA
NM_148938	*Slc1a3*	ACCAAAAGCAACGGAGAAGA G	GGCATTCCGAAACAGGTAACT C
NM_011421	*Smpd1*	TGGGACTCCTTTGGATGGG	CGGCGCTATGGCACTGAAT
NM_009213	*Smpd2*	TGGGACATCCCCTACCTGAG	TAGGTGAGCGATAGCCTTTGC
NM_021491	*Smpd3*	ACACGACCCCCTTTCCTAATA	GGCGCTTCTCATAGGTGGTG
NM_001164611	*Smpd4*	CACACTAGCCTCTTGAAGCGA	TGTAGAACCTCCAACTTGGCAT
NM_009230	*Soat1*	GAAGGCTCACTCATTTGTCAG A	GTCTCGGTAAATAAGTGTAGG CG
NM_146064	*Soat2*	ACAAGACAGACCTCTTCCCTC	ATGGTTCGGAAATGTTGCACC
NM_146126	*Sord*	GCTAAGGGCGAGAACCTGTC	CATGCTCCCAGTAGTGAACATC
NM_019972	*Sort1*	CCCGGACTTCATCGCCAAG	AGGACGAGAATAACCCCAGTG
NM_001172472	*Sphk1*	ATGGAACCAGTAGAATGCCCT	TCCGTTCGGTGAGTATCAGTTT A
NM_020011	*Sphk2*	CACGGCGAGTTTGGTTCCTA	CTTCTGGCTTTGGGCGTAGT
NM_009269	*Sptlc1*	ACGAGGCTCCAGCATACCAT	TCAGAACGCTCCTGCAACTTG
NM_011479	*Sptlc2*	AACGGGGAAGTGAGGAACG	CAGCATGGGTGTTTCTTCAAAA G
NM_009270	*Sqle*	ATAAGAAATGCGGGGATGTCA C	ATATCCGAGAAGGCAGCGAAC
NM_175283	*Srd5a1*	GAGTTGGATGAGTTGCGCCTA	GGACCACTGCGAGGAGTAG
NM_053188	*Srd5a2*	GATCCTGTGCTTTGGGAAACC	GCATCCCTACCGACACCAC
NM_020611	*Srd5A3*	CTACGTCATCTCAGTTGTGTGG	GAGCAGAGCACTAAGCCAGT
NM_009272	*Srm*	ACATCCTCGTCTTCCGCAGTA	GGCAGGTTGGCGATCATCT
NM_011485	*Star*	ATGTTCCTCGCTACGTTCAAG	CCCAGTGCTCTCCAGTTGAG
NM_021547	*Stard3*	GTGACTTGGAGCGCAGTTTG	GCCAGTGTTGGTATTTAGCTCG
NM_009293	*Sts*	CTCGCTGACATCATCCTCGC	GCGTTTTGTTGCCGTAGCAG
NM_011506	*Sucla2*	ACCCTTTCGCTGCATGAATAC	CCTGTGCCTTTATCACAACATC C
NM_019879	*Suclg1*	TGGGATACGACACGGGTCTTA	CAGAAGCCGTTGCTCCTGTT
NM_011507	*Suclg2*	CCCCGAAGATGGCTGAACC	ACCTCCTTTCAAACCGCTATTG

NM_023135	*Sult1e1*	ATGGAGACTTCTATGCCTGAGT	ACACAACTTCACTAATCCAGGTG
NM_017465	*Sult2b1*	ACATCACGGGACTGCTGTG	GTCGCAAGGCCCTTCATTCA
NM_011514	*Suv39h1*	GCAGTGTGTGCTGTAAATCTTCT	ATACCCACGCCACTTAACCAG
NM_022724	*Suv39h2*	CTGCCCAGGATAGCATTGTTC	CAAGTCTCGGCTCCACATTTAC
NM_001164483	*Synj1*	GGAAGAATGTCTCATGTTCGAGT	CCAGATAGTGCAGCATGGTATCA
NM_001113352	*Synj2*	GCTGAGGTTACAATCCGGTGG	CAGGCGGTCCTCTTCCTTG
NM_011528	*Taldo1*	GTAAAGCGCCAGAGGATGGAG	CTCTTGGTAGGCAGGCATCT
NM_146214	*Tat*	TGCTGGATGTTCGCGTCAATA	CGGCTTCACCTTCATGTTGTC
NM_001242615	*Taz*	ATGGTGTCTACCAGAAAGGGAT	CGGTGATTTTCAACATGCCACA
NM_011539	*Tbxas1*	TACCATAGTGACTGTGACTCTGC	GGTGCCTGATGCCCAACTT
NM_001136091	*Tcirg1*	CACAGGGTCTGCTTACAACTG	CGTCTACCACGAAGCGTCTC
NM_019911	*Tdo2*	ATGAGTGGGTGCCCGTTTG	GGCTCTGTTTACACCAGTTTGAG
NM_027179	*Tecr*	AAGCACTACGAGGTGGAGATT	TGAGGCTCTACCTTGTCCAGG
NM_009387	*Tk1*	AAGTGCCTGGTCATCAAGTATG	GCTGCCACAATTACTGTCTTGC
NM_021028	*Tk2*	AGCAGTGGTTTGTATTGAGGG	ACATGAGGCTCAGAGGGTTATG
NM_009388	*Tkt*	ATGGAAGGTTACCATAAGCCAGA	TGCAGCATGATGTGGGGTG
NM_031379	*Tktl1*	CTGCGAATCCGTTCCATCAG	GTCTGGGTTTTCGGGGTCTTC
NM_028927	*Tktl2*	TGGAGTCGGACACCCTTCA	CTCTGCCACACTACAGCACG
NM_028454	*Tm7Sf2*	GTCGCGGCTTTACTGATCCT	CAGGCAGATAGGCCGGTAG
NM_138758	*Tmlhe*	GAGAGGAAACATAGCTCAAGGTC	CCAGCATATTGCAGTTCCAAATG
NM_001136084	*Tph1*	AACAAAGACCATTCCTCCGAAAG	TGTAACAGGCTCACATGATTCTC
NM_173391	*Tph2*	GGTTGTCCTTGGATTCTGCTG	GCCTGGATTCGATATGAAGCAT
NM_009415	*Tpi1*	CCAGGAAGTTCTTCGTTGGGG	CAAAGTCGATGTAAGCGGTGG
NM_009417	*Tpo*	CTTGGAGCTATGGCAATAATGCT	GAGCTGGCTCGTTTCCACA
NM_010067	*Trdmt1*	CACGCGCTGCGAAAAAGTC	CCCTGTAGGCCAATTCTTGTG
NM_021481	*Treh*	TCCAACCTCACCCCACTTTG	CCACTGCTGGCCTGTGTTA
NM_028604	*Trmt11*	GCTCCTTGAATCGCTATCTCTTG	ACTGTTAGTGAACTGCCCTCC
NM_009775	*Tspo*	GCCTACTTTGTACGTGGCGAG	ATGGCTGAATACAGTGTTGCC
NM_031201	*Tsta3*	AAGGTGGTTGCAGATGGGG	CCATTGCAGCGAGATGGATGA

NM_001042523	*Txnrd1*	CCCACTTGCCCCAACTGTT	GGGAGTGTCTTGGAGGGAC
NM_013711	*Txnrd2*	AAACCAGGACTTTGAATCTGGAG	GATGTGGGGAACAGAGGTAGC
NM_138302	*Tymp*	CGCGGTGATAGATGGAAGAGC	CACACCTCCTGTGGAGTGTT
NM_021288	*Tyms*	GGAAGGGTGTTTTGGAGGAGT	GCTGTCCAGAAAATCTCGGGA
NM_011661	*Tyr*	CTCTGGGCTTAGCAGTAGGC	GCAAGCTGTGGTAGTCGTCT
NM_133806	*Uap1*	CCATCCCCCGCTTGAAAGAT	CCCTCTCCAGCATAAGAGATGA
NM_011675	*Uck1*	ACCGCTAGTGGCAAGTCAAC	AGAACCTTGTAGAAGCAGTCCT
NM_030724	*Uck2*	CTTCCGTTTGTGCTAAGATCGT	GTGAGGACTCGGTAGAAGCTAT
NM_026765	*Uckl1*	CAGGCCGAGCAGAATGAAAC	CCTTGGACTGTGTACCATGTTC
NM_011673	*Ugcg*	GGAATGGCCTTGTTCGGCT	CGGCTGTTTGTCTGTTGCC
NM_009466	*Ugdh*	AGTAGTCGAATCCTGTCGAGG	CTCCGTGACAATTTTGTACCCA
NM_139297	*Ugp2*	AGCAAAGCTATGTCTCAAGATGG	GAGGCTGCTGTGGTAAGTATTT
NM_201645	*Ugt1a1*	GCTTCTTCCGTACCTTCTGTTG	GCTGCTGAATAACTCCAAGCAT
NM_053184	*Ugt2a1*	AGCCTTCTAGGAATGAGTCTTGG	CAAGGACAGTCACATTATGCTCT
NM_028094	*Ugt2a3*	CGTGTGGCCCTGTGATATGAG	GTGCAGTGGAATACGTTTACTCT
NM_009471	*Umps*	GTCACCGAGCTGTATGACGTG	GGTAACGCTGTATAAGGAACTCC
NM_133995	*Upb1*	GTGCCTGGAAAAACATCTGCC	GCGGGATTCTGTTCTGAACGA
NM_001159402	*Upp1*	ACAGGAACTGAAGCAAAGGAC	GTTGAAATGGTAGAGCACGTCTT
NM_029692	*Upp2*	GGGAGCGTCCAGAGTATGG	CTGGTAGGTTGTGTGTTTTGGT
NM_001081189	*Uprt*	GGAAAATAACGCTGCTATGGCT	GAGAGTCCCGCTCCGAAAG
NM_197979	*Uqcr10*	ATCCCTTCGCGCCTGTACT	GTGCTCGTAGATCGCGTCT
NM_026219	*Uqcrb*	GGCCGATCTGCTGTTTCAG	CATCTCGCATTAACCCCAGTT
NM_025407	*Uqcrc1*	AGACCCAGGTCAGCATCTTG	GCCGATTCTTTGTTCCCTTGA
NM_025899	*Uqcrc2*	AAAGTTGCCCCGAAGGTTAAA	GAGCATAGTTTTCCAGAGAAGCA
NM_025710	*Uqcrfs1*	GAGCCACCTGTTCTGGATGTG	GCACGACGATAGTCAGAGAAGTC
NM_025641	*Uqcrh*	GTGGACCCCCTAACAACAGTG	CGGGAAGACACGCGATTATCA
NM_025352	*Uqcrq*	CCTACAGCTTGTCGCCCTTT	GATCAGGTAGACCACTACAAACG
NM_144940	*Uroc1*	ACATGCTCCGGTTAGAACTCC	CCAAACTGTCTTAGCTCCTGG
NM_026430	*Uxs1*	CCAGAAATACCCGCCTGTAAA	GTTTGTCAGTTAGATGGGAGC

		G	C
NM_013933	*Vapa*	ATCCTGGTCCTCGACCCTC	GCAGCATTACTGAAACAGTCACA
NM_019806	*Vapb*	ATGTCACTTCTGACCTCAGGAT	GTGAACGAGTCCCACACCA
NM_011690	*Vars*	GTCCAGCAGTGGGTCAGTTAT	GCAGCAGTAAGGCTGTCAC
NM_175137	*Vars2*	ACATCCCTCTGTGGAGACTGG	GCAGCTAGGTCCCCCATTTC
NM_011694	*Vdac1*	CCCACATACGCCGATCTTGG	GTGGTTTCCGTGTTGGCAGA
NM_011695	*Vdac2*	CTCCACCCTATGCTGACCTC	CCCGCTAACTTTACCAGTGTCT
NM_011696	*Vdac3*	CAAAGGGTATGGGTTTGGCAT	TTGGTCTCTAGGTTGCCTGAT
NM_001164488	*Wars*	GCTGGAAATGCACCAAAAGATG	GCTTGTCCGCACTGTCCAT
NM_027462	*Wars2*	GTCCACACACGTTCCTGTCG	GGACTTGGGCAATGGAAAGAA
NM_025375	*Wbscr22*	GCATCTCGTAGCCGGAGAC	CGTGAGTTGCGAACGTATTTCC
NM_001177884	*Whsc1*	GGCCAGAACAAGCTCTTACAA	TGTGGGCTCCCATAAAAGCTC
NM_001001735	*Whsc1l1*	TCCACTGGTGTTAAGTTCCAGG	GGCACCTCTTGTGTTAATTTTGG
NM_011723	*Xdh*	ATGACGAGGACAACGGTAGAT	TCATACTTGGAGATCATCACGGT
NM_001199568	*Xylb*	TCAGCACGCAGCAGGTTAAA	CGCCCTGAGTTCCAAATTCC
NM_178691	*Yod1*	GCCAAATCGCCGCTATCAC	ATGTCCCGGTCGCTGAGAT
NM_023162	*Znrd1*	CCCCGCTCCAACTTTCAATCA	AGAAGCCACAACGCGAACA

Urea cycle genes

NM_010324	*Got1*	GCGCCTCCATCAGTCTTTG	ATTCATCTGTGCGGTACGCTC
NM_010325	*Got2*	GGACCTCCAGATCCCATCCT	GGTTTTCCGTTATCATCCCGGTA
NM_007494	*Ass1*	ACACCTCCTGCATCCTCGT	GCTCACATCCTCAATGAACACCT
NM_001080809	*Cps1*	ACATGGTGACCAAGATTCCTCG	TTCCTCAAAGGTGCGACCAAT
NM_028122	*Slc14a1*	TTAAAGTAGACCGGGGTGAAAAC	GATTCCACTGATGGGGTTGC
NM_207651.3	*Slc14a2*	AAGGAGATGTCTGACAGCAACA	GGGCTGGGTGTGTATCCTG
